# A systematical genome-wide analysis and screening of WRKY transcription factor family engaged in abiotic stress response in sweetpotato

**DOI:** 10.1186/s12870-022-03970-6

**Published:** 2022-12-28

**Authors:** Siyuan Liu, Chengbin Zhang, Fen Guo, Qing Sun, Jing Yu, Tingting Dong, Xin Wang, Weihan Song, Zongyun Li, Xiaoqing Meng, Mingku Zhu

**Affiliations:** 1 Institute of Integrative Plant Biology, School of Life Sciences, Jiangsu Normal University, 221116 Xuzhou, Jiangsu Province China; 2 Agricultural Bureau of Linyi City, 276000 Linyi, Shandong Province China; 3 Jiangsu Key laboratory of Phylogenomics & Comparative Genomics, School of Life Sciences, Jiangsu Normal University, 221116 Xuzhou, Jiangsu Province China; 4 Jiangsu Xuzhou Sweetpotato Research Center, 221131 Xuzhou, Jiangsu Province China

**Keywords:** Abiotic stress, Expression profiles, Molecular characterization, Regulatory network, Sweetpotato, WRKY transcription factor

## Abstract

**Background:**

WRKY transcription factors play pivotal roles in regulating plant multiple abiotic stress tolerance, however, a genome-wide systematical analysis of WRKY genes in sweetpotato is still missing.

**Results:**

Herein, 84 putative *IbWRKYs* with WRKY element sequence variants were identified in sweetpotato reference genomes. Fragment duplications, rather than tandem duplications, were shown to play prominent roles in *IbWRKY* gene expansion. The collinearity analysis between *IbWRKYs* and the related orthologs from other plants further depicted evolutionary insights into *IbWRKYs*. Phylogenetic relationships displayed that IbWRKYs were divided into three main groups (I, II and III), with the support of the characteristics of exon-intron structures and conserved protein motifs. The *IbWRKY* genes, mainly from the group Ib, displayed remarkable and diverse expression profiles under multiple abiotic stress (NaCl, PEG6000, cold and heat) and hormone (ABA, ACC, JA and SA) treatments, which were determined by RNA-seq and qRT-PCR assays, suggesting their potential roles in mediating particular stress responses. Moreover, IbWRKY58L could interact with IbWRKY82 as revealed by yeast two-hybrid based on the protein interaction network screening. And abiotic stress-remarkably induced IbWRKY21L and IbWRKY51 were shown to be localized in the nucleus and had no transactivation activities.

**Conclusion:**

These results provide valuable insights into sweetpotato *IbWRKYs* and will lay a foundation for further exploring functions and possible regulatory mechanisms of IbWRKYs in abiotic stress tolerance.

**Supplementary Information:**

The online version contains supplementary material available at 10.1186/s12870-022-03970-6.

## Background

Owing to sessile lifestyles, ever-changing environmental vagaries including high salinity, drought, and extreme temperatures, remarkably restrict plant distribution, negatively affect their growth and reduce crop yield [1, 2]. For instance, the adverse environment can lead to an estimated yield loss of about 70% in major crops [3]. To adapt to adverse environmental challenges, plants have evolved a series of interconnected defense mechanisms to facilitate their survival, which involve multiple processes, such as sensing, signal transduction, transcript processing, translation and post-translational modification [4, 5]. The resistance mechanisms are extremely sophisticated because plants should balance growth and the stress response under stressful conditions [3]. Substantial genetic and molecular researches have identified transcription factors (TFs) as master regulators of plant abiotic stress tolerance, such as bZIP, MYB, WRKY, NAC, and AP2/ERF [6–9]. In plant genomes, about 7% of coding sequences are assigned to TFs, and many of which are immediate-early abiotic stress response genes [10].

The WRKY TF family, originally isolated from sweetpotato as SPF1 (Sweet Potato Factor1) [11], constitutes one of the largest plant TF families with 72 genes in *Arabidopsis* and 105 genes in rice [12]. With the ever-increasing number of completed plant genome sequencing, WRKY TFs have been extensively identified at the genome-wide level, such as 61 in cucumber [13], 102 in cotton [14], 124 in wheat [15], 125 in maize [16], 164 in banana [17], and 182 in soybean [18]. Members of WRKY TFs possess a conserved WRKY domain about 60 amino acids long consisting of the N-terminal invariant WRKYGQK sequence and a C-terminal zinc finger motif formed mainly by either C_2_H_2_ (C_X4–5_C_X22–23_H_X_H) or C_2_HC (C_X7_C_X23_H_X_C) [19, 20]. The WRKYGQK sequence was involved in DNA-binding, and the zinc finger structure was related to protein interaction and helper DNA binding [21]. Based on the number of WRKY domains and the features of zinc finger motifs, WRKY TFs can be divided into three main groups. Members of group I has two WRKY domains, while only single WRKY domain exists in group II and III, which are distinguished by the zinc finger motifs [19]. Besides, in general WRKY TFs can act as activators or repressors, which are enriched in potential transcriptional activation or repression domains [22].

The roles of WRKY TFs in regulating plant response to various environmental stresses such as salt, drought, heat and cold have been widely documented in many plants [20, 23]. In *Arabidopsis*, multiple WRKY proteins, such as WRKY-8/-28/-30/-54/-70, have been shown to modulate abiotic stress tolerance [24, 25]. Regulations of the transcription of *WRKY-25*/-*26*/-*33*/-*3*9 affected heat stress tolerance through modulations of transcriptional reprogramming of heat-related genes [20, 26]. WRKY TFs have also been tested for their involvements to enhance abiotic stress tolerance in crops. In rice, overexpression of *OsWRKY11*/*30*/*45*/*72* significantly enhanced salt, drought or heat tolerance [20, 27, 28]. Genome-wide identification of *TaWRKY* genes in wheat demonstrated differential expression profiles under multiple abiotic stresses, and ectopic expression of *TaWRKY75-A* in *Arabidopsis* improved drought and salinity tolerance [15]. Recently, it was demonstrated that *Pyrus betulaefolia* PbWRKY40 positively regulated salt tolerance by binding to the promoter of *PbVHA-B1* [29]. Furthermore, recent findings revealed that WRKYs also function as central components of ABA-dependent signaling networks. For instance, WRKY57 was shown to improve drought tolerance in *Arabidopsis* by enhancing ABA levels, it could directly bind to the promoter of ABA-related *RD29A* and *NCED3* genes [30]. Mutation of *WRKY63*/*ABO3* conferred ABA hypersensitivity and impaired drought tolerance by binding to the W-box in the promoter of *ABF2* [31].

Sweetpotato (*Ipomoea batatas*) has many advantages such as wide adaptability, high starch content, strong stress resistance, high yield and low input requirements, making it a globally important food crop [32]. The warranty of sweetpotato production and safety thus is especially important, however, multiple abiotic stresses have brought serious threat to sweetpotato production. To date, few of WRKY genes have been functionally identified in sweetpotato. Previously, 79 *WRKY* genes were isolated from two sweetpotato cultivars Jishu 26 and Nonglin 54 by the transcriptome analysis under salt stress [33]. Recently, overexpression of *IbWRKY2* improved drought and salt tolerance in *Arabidopsis* by interacting with VQ4 protein [24]. However, the genome-wide systematical survey on WRKYs in sweetpotato is still not available, many details remain to be further elucidated. In this study, a systematical analysis of 84 identified sweetpotato *IbWRKYs* including chromosomal location, classification relationship, gene duplication, gene structure, conserved domain, cis-element, and the response to various abiotic stresses and hormones was accomplished. Furthermore, subcellular localization, transactivation activity and protein interaction were performed to further interpret the molecular functions and regulatory modes of IbWRKYs. These findings may pave the way for an in-depth exploration of the critical role of sweetpotato IbWRKYs in abiotic stress responses.

## Results

### Identification and analysis of *IbWRKY* genes in sweetpotato

In this study, a set of 84 putative *IbWRKY* genes were identified through the genome database of sweetpotato cultivar Taizhong6 [34]. These genes were consistently named *IbWRKY1* ~ *IbWRKY84* according to their chromosome locations (Additional file 1). Molecular characterizations were analyzed including the length of amino acid residues, molecular weight, isoelectric point, and subcellular location. The detailed data are presented in Additional file 2. The length of IbWRKYs varied from 120 (IbWRKY84) to 838 (IbWRKY36) amino acid residues, accordingly, the molecular weight ranged from 13.2 to 93.9 kDa. The pI of 48 IbWRKYs were acidic, and the remaining 36 were basic, and distributed greatly from 4.89 (IbWRKY33) to 10.74 (IbWRKY43). The subcellular location prediction suggested that almost all IbWRKYs were located in the nucleus (Additional file 2). Among them, IbWRKY34 and IbWRKY70 share exactly the same protein sequence even though they are distributed in different chromosomal position. Since sweetpotato is a hexaploid crop and there are potential differences in gene sequences among different cultivars, thus the reported sweetpotato WRKYs are not completely consistent with the corresponding IbWRKYs identified in this study.

### Chromosomal location analysis of *IbWRKY* genes

The analysis of genomic distribution shows that the 84 loci of *IbWRKY* genes are mapped to all 15 chromosomes of sweetpotato. In general, the distribution of *IbWRKYs* is relatively clustered rather than uniform, which may be due to uneven gene duplication of chromosome fragments. Chr 1 contains the largest number of *IbWRKYs* (10 genes), and the second is Chr 9 and Chr 15 (both 9 genes). However, Chr 3, Chr 8, Chr 10 and Chr 12 only contain two or three *IbWRKYs*. The data revealed that the distribution of *IbWRKYs* is uneven. In addition, the distributions of *IbWRKYs* are also not proportional to chromosome size. For instance, the two large chromosomes Chr 6 and Chr 12 only contain two *IbWRKYs* (Additional file 1).

### Phylogenetic classification of sweetpotato IbWRKY proteins

**Fig. 1:**
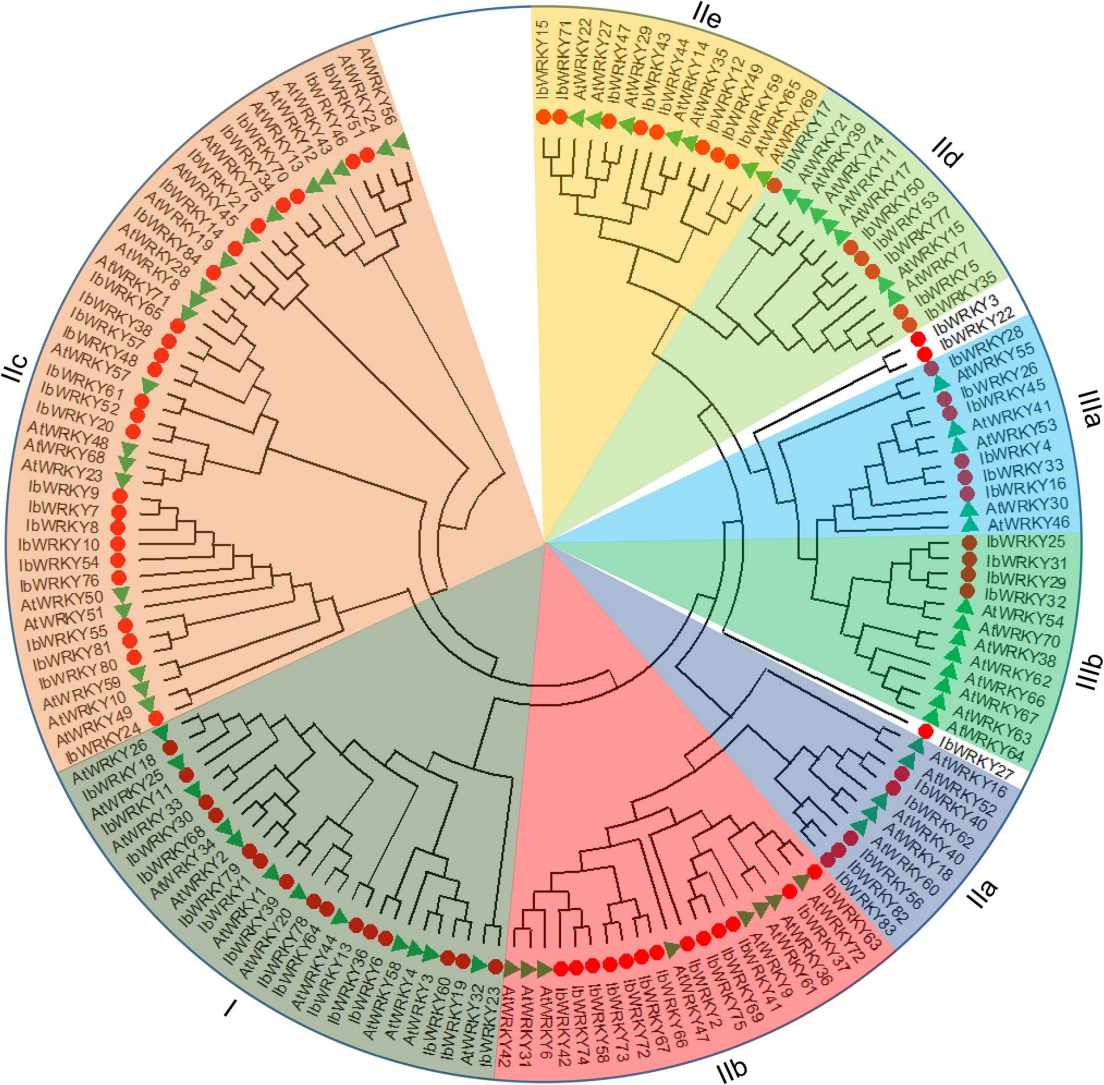
Unrooted phylogenetic tree of sweetpotato IbWRKYs and *Arabidopsis* AtWRKYs. The phylogenetic relationships were derived by the Maximum Likelihood method and the best evolutionary model JTT + G + F obtained through MEGA X was employed with the bootstrap value of 1000. Different subgroups are named based on the reports in *Arabidopsis*. The red circles and green triangles represent the sweetpotato IbWRKYs and *Arabidopsis* AtWRKYs, respectively

To explore the phylogenetic relatedness among sweetpotato IbWRKYs, 72 *Arabidopsis* AtWRKYs were detected together with 84 sweetpotato IbWRKYs identified in this study using their complete protein sequences (Additional file 3). The results depicted that 84 IbWRKYs were divided into three main groups (I; II: IIa, IIb, IIc, IId and IIe; III: IIIa and IIIb) as defined previously based on the number of WRKY domains and the structure of zinc-finger motifs [19, 35]. Differently, IbWRKY3, IbWRKY22 and IbWRKY27 did not belong to any of the groups because of their distinct sequence compositions. Among them, IbWRKY3 and IbWRKY22 were classified into the same branch. The distributions of IbWRKYs were largely diversified and uneven in different subgroups. Among them, 15 IbWRKYs belong to group I; and 5, 13, 24, 6 and 8 IbWRKYs belong to subgroups IIa ~ IIe, respectively; and 10 IbWRKYs belong to group III (Fig. 1).

Besides, the conserved WRKY domains extracted from the sweetpotato IbWRKYs were also used for phylogenetic analysis, and the tree was almost identical to the above, except that subgroups IIC and IIe differed by only one member each (Additional file 4). The results suggest that whether WRKY TFs are classified into the same subgroup mainly depends on the similarity of WRKY domains.

**Fig. 2:**
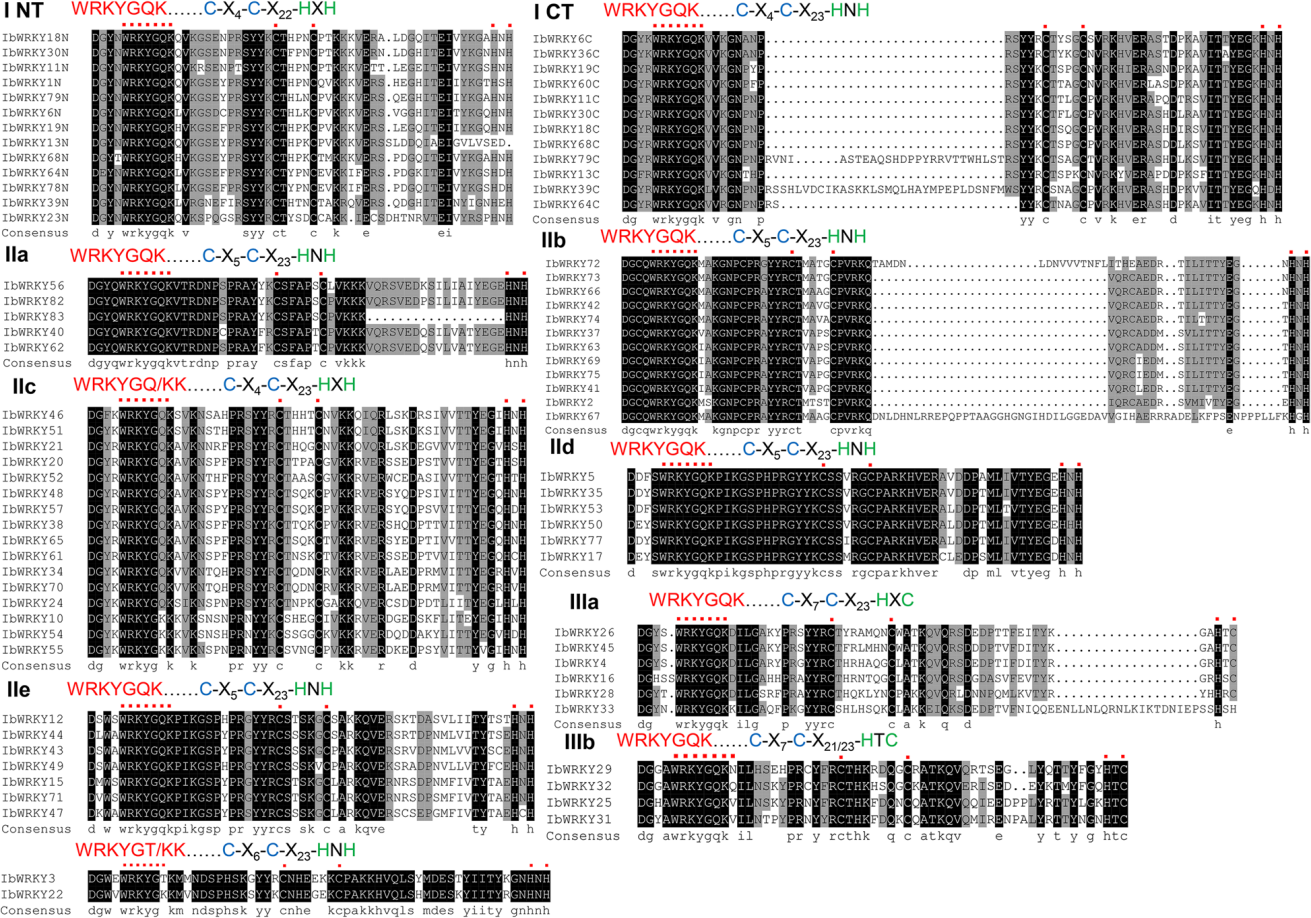
Multiple sequence alignment of WRKY domains extracted from IbWRKY proteins. Identity, similarity, and deletions among WRKY alignments are indicated by black, grey shades and hyphens, respectively. The amino acid residues in the WRKY domain and the zinc finger domain are identified by red dots

To reveal how conservative the WRKYGQK peptides and zinc-finger domains were in each subgroup, their sequence composition was also exhibited in Additional file 2, and the sequence alignment of extracted WRKY domains was further conducted using DNAMAN 9.0 (Fig. 2). The WRKY proteins have two standard motifs, first, most of the IbWRKYs contain the conserved “WRKYGQK” motif, whereas IbWRKY59 has no the typical WRKY sequence, but still has a variant WRKY domain, which was confirmed by CD-search. 15 IbWRKYs in group I contained two WRKY domains. And two variants, WRKYGTK (IbWRKY3) and WRKYGKK (IbWRKY-7–10, -22, -54, -55, -76, -80 and − 81), were also observed in IbWRKYs (Additional file 2). The other one was the zinc-finger domain with two types: C_2_H_2_ and C_2_HC. However, no or only fragmentary zinc-finger motifs were observed in 12 IbWRKY members. The zinc-finger motifs from the Group I and II members belonged to the C_2_H_2_ type, while Group III members belonged to the C_2_HC type, except IbWRKY13 containing mutated C_2_XX. In addition, unlike previously reported WRKYs from many other plants, IbWRKY3 and IbWRKY22 contained a specific C_X6_C_X23_H_X_H zinc-finger backbone, thus they did not belong to any of the reported groups (Fig. 2).

### Gene structure and conserved domain analyses of IbWRKYs

**Fig. 3:**
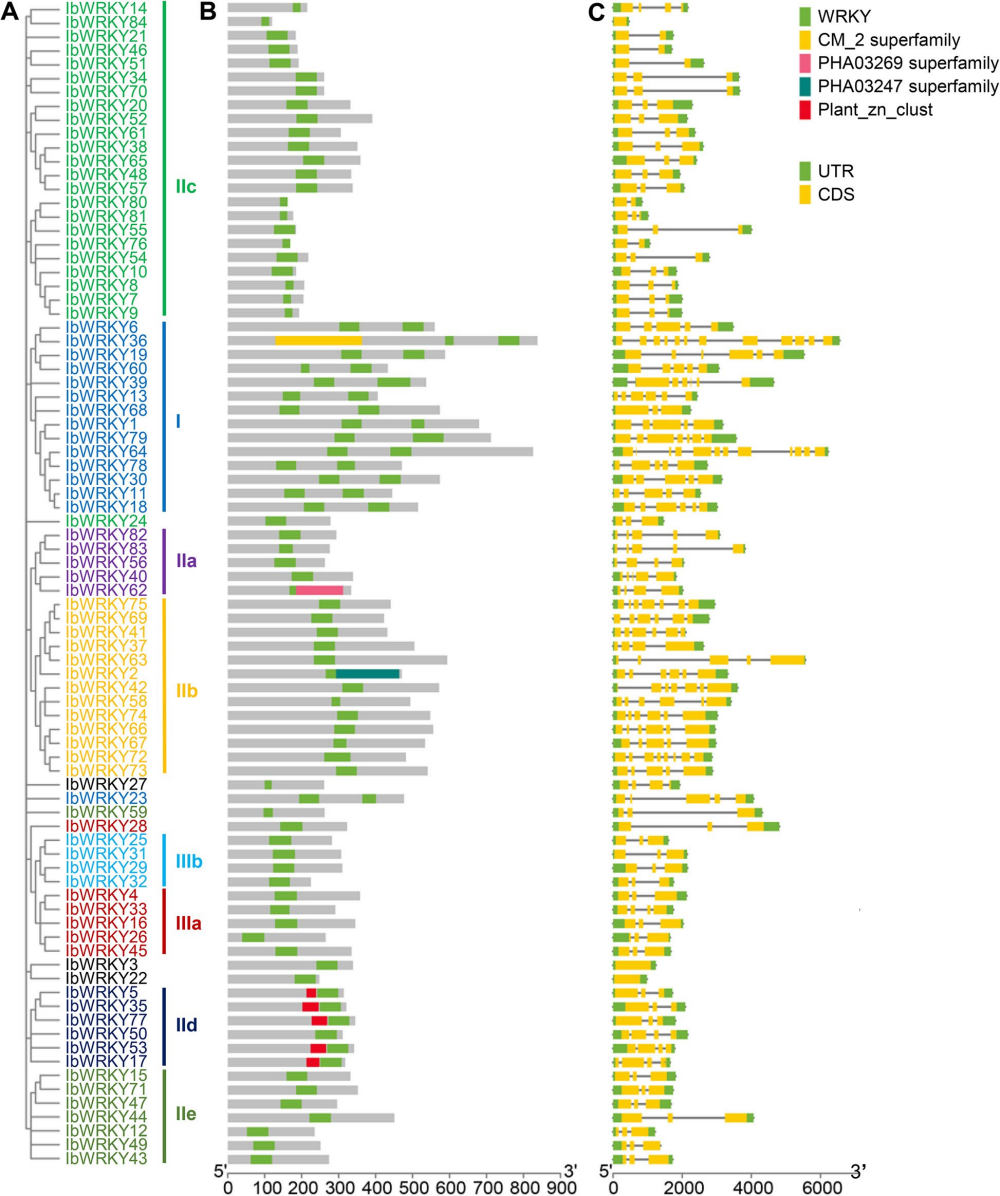
Gene structures and conserved domain distributions of 84 sweetpotato IbWRKYs. **A**. The phylogenetic tree of 84 IbWRKYs was constructed using the same parameters descripted in Fig. 1. **B**. Distributions of conserved domains detected by CD-search in IbWRKYs. **C**. Gene structures of 84 *IbWRKY* genes. Exons and UTR are represented using yellow and green bars

The diversities of gene structure are significant components in the process of gene evolution. To obtain insights on the *IbWRKY* gene evolution, the genetic structures of each *IbWRKY* gene was evaluated. The varying patterns of structural features displayed that the majority of *IbWRKYs* (43 out of 84) had two or three exons, and *IbWRKY36* and *IbWRKY64* had the biggest number of exons (12), nonetheless, *IbWRKY-3/-22/-84* had no introns. The remaining *IbWRKY* genes had four to eight exons. To better visualize gene structures and conserved motifs, a phylogenetic tree was generated using the completed IbWRKY proteins (Fig. 3 A). Previous data exhibited that the members of closely clustered WRKYs generally contained similar gene structures and sequence compositions. Similarly, our results showed that the majority of *IbWRKYs* in the same subgroups also showed similar gene organizations. For instance, most *IbWRKYs* had only three to four exons in subgroups IIIa, IIIb, IId and IIe, while most members from subgroup IIb contained over five exons (Fig. 3B and C).

Results analyzed by NCBI CD-Search revealed that all IbWRKYs contain one or two conserved WRKY domains. Notably, 13 IbWRKY members contained incomplete WRKY domain (less than 30 amino acid residues, while WRKY domains are generally about 60 amino acid residues long), including IbWRKY-7/-8/-9/-14/-27/ 36 N/-58/-59/-60 N/-76/-80/-81/-84, which were mainly due to the lack of integral C-terminal zinc-finger domain. Besides, IbWRKYs from multiple subgroups contained additional conserved domains. For instance, almost all IbWRKYs from IId subgroup contained a Plant_zn_clust (Fig. 3B and C).

#### Motif composition analyses of IbWRKY proteins in sweetpotato

**Fig. 4:**
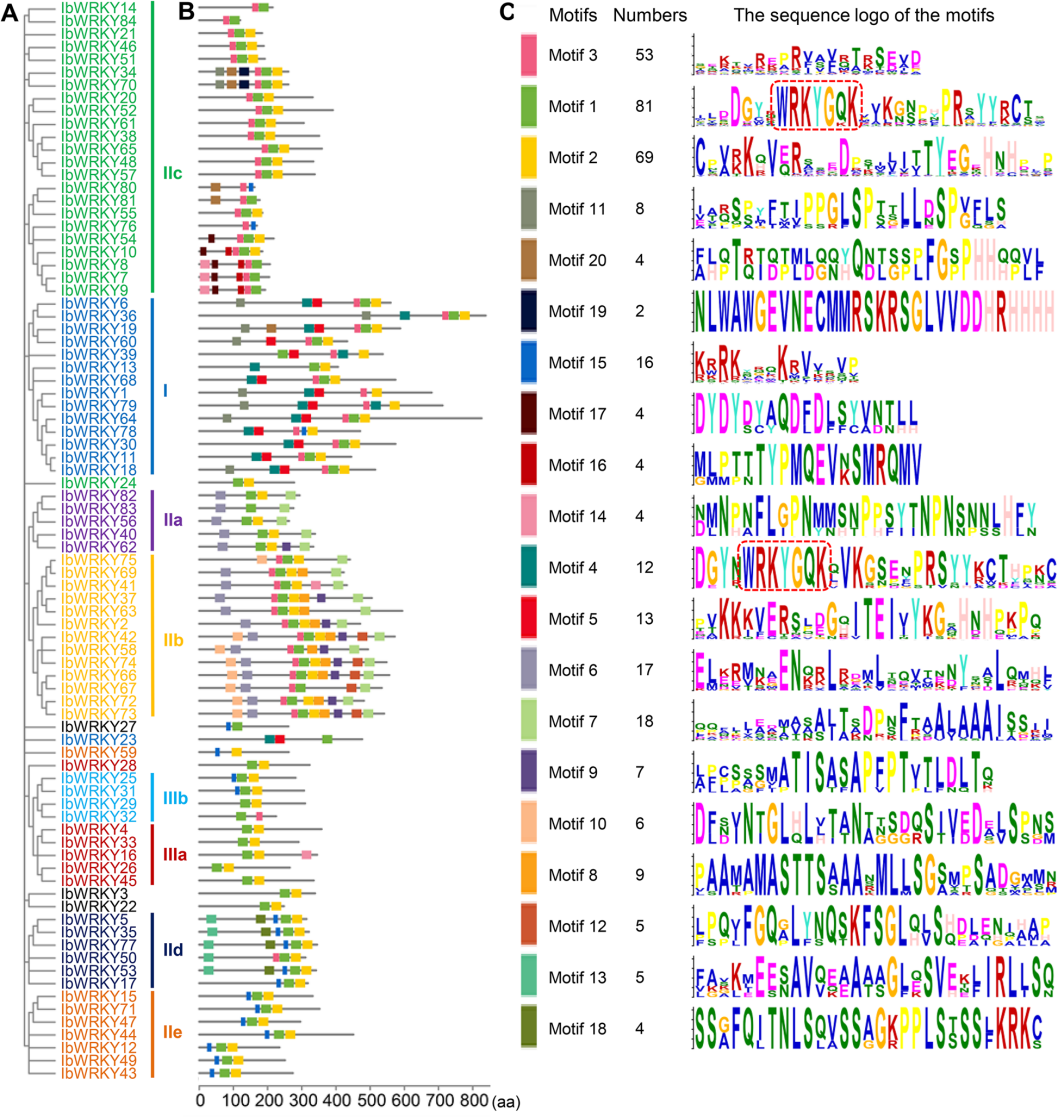
Distributions of conserved amino acid motif compositions within the IbWRKY proteins identified by MEME. **A**. The phylogenetic tree of 84 IbWRKYs was constructed using the same parameters descripted in Fig. 1. **B**. The distribution of 20 most significantly conserved motifs in IbWRKYs. Different motifs and their positions in each IbWRKY member are represented by differently colored boxes. **C**. The sequence logo of conserved motifs. The conserved heptapeptide WRKYGQK is marked with a red box

To further compare the sequence compositions of IbWRKYs, 20 common or specific motifs were identified within 84 IbWRKYs by the MEME tool. The detailed information of these motifs is listed in Additional file 5. Generally, IbWRKY members with similar motif compositions were divided into the same subgroup in the phylogenetic tree, which further consolidate the classification analysis (Fig. 4). Consistent with the homologs in other plants such as *Arabidopsis*, rice and maize, most IbWRKYs had conserved heptapeptides WRKYGQK (Motif 1 or Motif 4), and Motif 2 and Motif 5 were comprised of the C_2_H_2_ and C_2_HC motifs. And the results exhibited that multiple motifs particularly existed in different subgroups. Most IbWRKYs from group I contained five to six different conserved motifs, and most members from subgroup IIb had six to nine distinct conserved motifs. Notably, several motifs only existed in one subgroup or few IbWRKYs. For instance, Motifs 16 and 19 are only present in subgroup IIc, and Motif 13 existed only in subgroup IId. The results suggested that the structure complexity of IbWRKYs and the specific motifs in different groups might play diverse roles in evolution and function.

### Collinearity survey of *IbWRKY* genes in sweetpotato

**Fig. 5:**
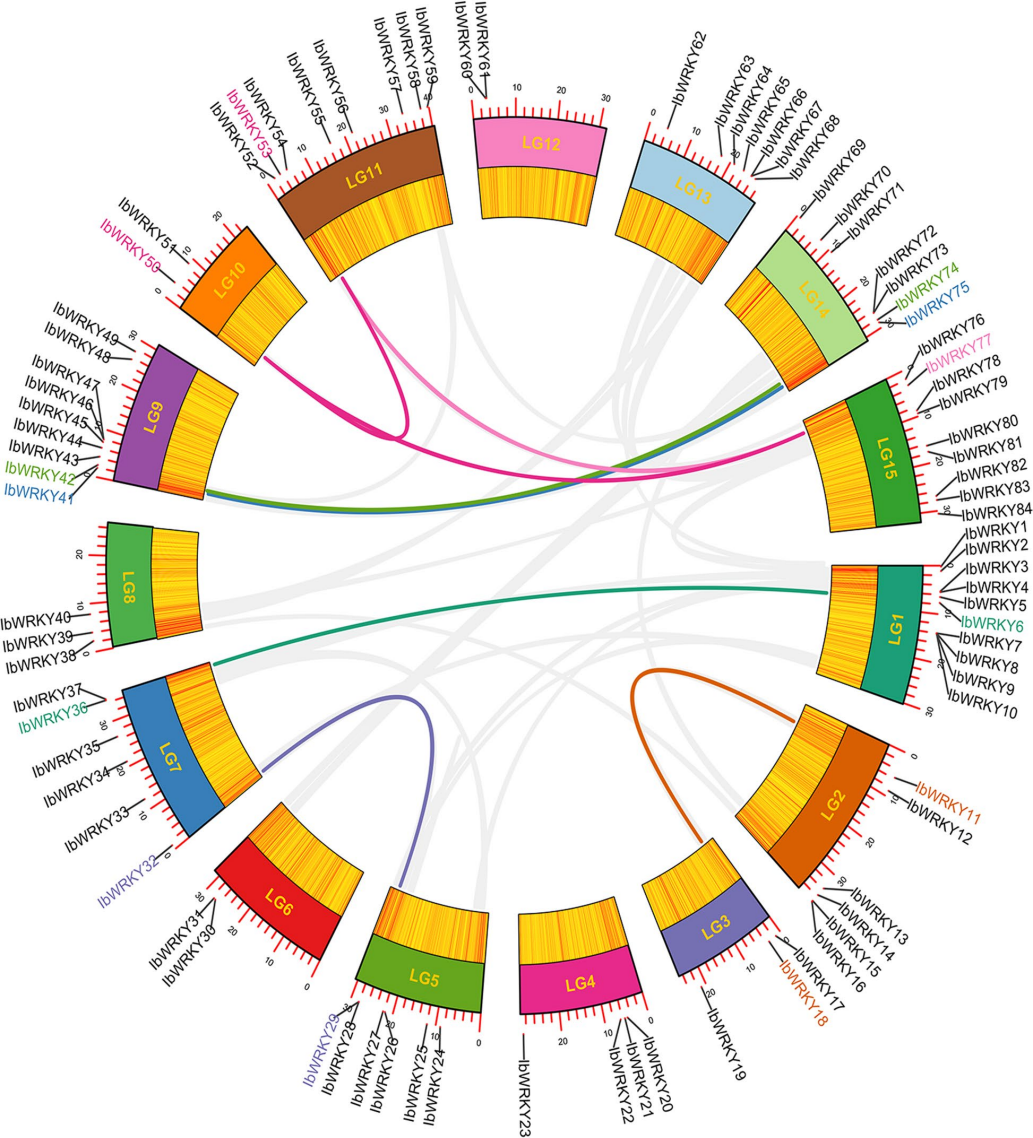
Inter-chromosomal relations of *IbWRKY* genes in sweetpotato chromosomes. 15 chromosomes are indicated by different colors. The gene density is displayed using a heatmap. The synteny *IbWRKY* gene pairs in different chromosomes are marked with colored curves

Genome duplication play an important role in promoting evolutions and expansions of gene family [36]. The gene duplications were investigated to detect the potential expansion mechanisms of *IbWRKYs*. Among 84 *IbWRKY* genes, only one pair of tandem duplication was found between *IbWRKY8* and *IbWRKY9* (Additional file 1). Further, eight gene pairs were recognized as segmental duplications on 10 of 15 chromosomes as follows: *IbWRKY6/36*, *IbWRKY11/18*, *IbWRKY29/32*, *IbWRKY41/75*, *IbWRKY42/74*, *IbWRKY50/53*, *IbWRKY50*/*77*, and *IbWRKY53/77* (Fig. 5 and Additional file 6).

**Fig. 6:**
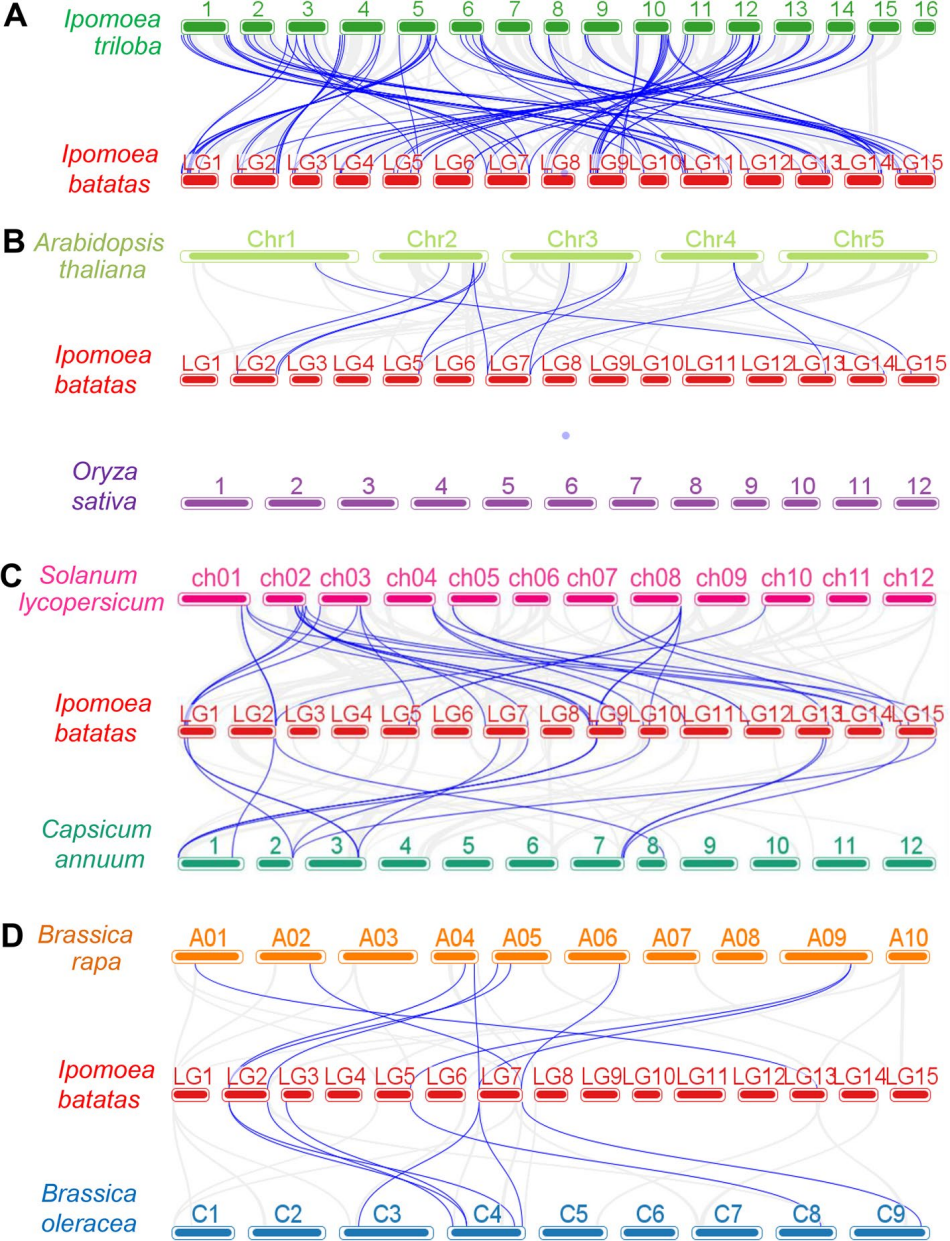
Synteny analyses of WRKYs between sweetpotato and different plant species including *Ipomoea triloba* (**A**), *Arabidopsis* and rice (**B**), tomato and pepper (**C**), cabbage and *Brassica oleracea* (**D**). The blue lines connecting the chromosomes indicate the syntenic WRKY gene pairs within the genomes of sweetpotato and other plants

To further assess the evolutionary history of *IbWRKY* genes, comparative syntenic maps were generated between sweetpotato and seven plants, including *Ipomoea triloba*, two model plants (*Arabidopsis* and rice), two Solanaceae plants (tomato and pepper), and two Brassica plants (cabbage and *Brassica oleracea*). As shown in Figs. 6 and 88 orthologous gene pairs generated by 68 *IbWRKYs* between sweetpotato and *Ipomoea triloba* were identified, followed by tomato (30 genes with 31 gene pairs), pepper (15 genes with 15 gene pairs), *Arabidopsis* (11 genes with 13 gene pairs), cabbage (6 genes with 9 gene pairs) and *Brassica oleracea* (6 genes with 8 gene pairs). Therefore, many *IbWRKY* genes had syntenic relationships with two or three genes from other plants, especially with the *Ipomoea triloba* (including 16 *IbWRKYs*) (Additional file 7). However, no orthologous gene pair was found between sweetpotato and rice. Obviously, there were far more orthologous genes between sweetpotato and *Ipomoea triloba* than that between sweetpotato and other six species. Moreover, we found that *IbWRKY-16/-74/-79* could form a collinear relationship between sweetpotato and *Ipomoea triloba*/*Arabidopsis*/tomato/pepper, but not between sweetpotato and the two Brassica species. And five *IbWRKYs*, *IbWRKY-14/-29/-32/-36/-64*, were collinear with all tested plants except rice (Additional file 8).

### Transcriptome screening of salt-responsive *IbWRKYs* and the expression profiles under abiotic stresses

To certify the response of *IbWRKYs* to abiotic stresses, their expression levels were analyzed using our previous transcriptomic data of salt-tolerant and salt-sensitive sweetpotatoes under salt stress [37]. We found that the expressions of most screened *IbWRKYs* were salt-responsive or genotype-specific (cultivar XuShu22 or XuShu32) (Additional file 9). Afterwards, qRT-PCR assays were accomplished under various abiotic stresses for ten selected *IbWRKYs* that exhibited remarkable differences in RNA-seq data (mainly screened from salt-tolerant cultivar XuShu22). Notably, the hexaploid genome of sweetpotato complicates the transcripts, and gene sequences of different cultivars are generally diverse. The sequence information of several salt-responsive WRKY transcripts screened by RNA-seq in cultivar Xushu22 is not completely consistent with that of 84 *IbWRKYs* identified in cultivar Taizhong6 in this study. Therefore, here the homologous genes with their protein sequence similarity less than 95% were named the corresponding WRKY-like genes.

**Fig. 7:**
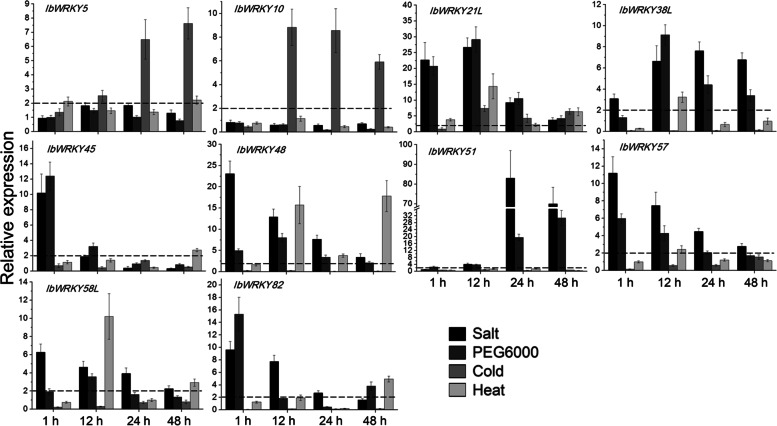
Relative gene expression of 10 *IbWRKYs* under salt, PEG6000, cold and heat stresses detected by qRT-PCR. The expression value at 0 h was normalized to 1, and the Y-axis delineates the expression fold changes comparing with 0 h. Bars represent the mean of three biological replicates ± SE. The two-fold thresholds are presented by dotted lines

As depicted in Fig. 7, the transcription of the tested *IbWRKY* genes showed marked upregulations after at least one of the four abiotic stress treatments including NaCl, PEG6000, cold or heat. Wherein, *IbWRKY21L* transcription could be upregulated by four stresses, and the expression of *IbWRKY-38 L*/-*45*/-*048*/-*57*/-*58 L/-82* was increased by three stresses. Notably, the expression of *IbWRKY21L* and *IbWRKY51* showed the highest induction after both salt and dehydration stresses with 26.7–83-fold. The upregulated transcription of other *IbWRKYs* except *IbWRKY5* and *IbWRKY10* could also be detected under both salt and dehydration stresses to relative low levels (3.6–23.1-fold), which were in good agreement with the transcriptomic data. Only the expression of *IbWRKY-5/-10/-21 L* was improved with 7.4–8.8-fold under cold, and inhibited mRNA was found in that of other *IbWRKYs*. For heat stress, *IbWRKY21L* and *IbWRKY48* exhibited high inducible levels with 14.4–17.8-fold, followed by the enhanced expression of *IbWRKY58L*, and transcriptions of five *IbWRKYs* including *IbWRKY-5/ -38 L/-45*/-*57*/*-82* could be lightly enhanced by heat with 2.2–4.9-fold (Fig. 7). These data suggested that *IbWRKYs* might participate in sweetpotato responses to various abiotic stresses.

### Expression profiles of *IbWRKYs* under different hormones

**Fig. 8:**
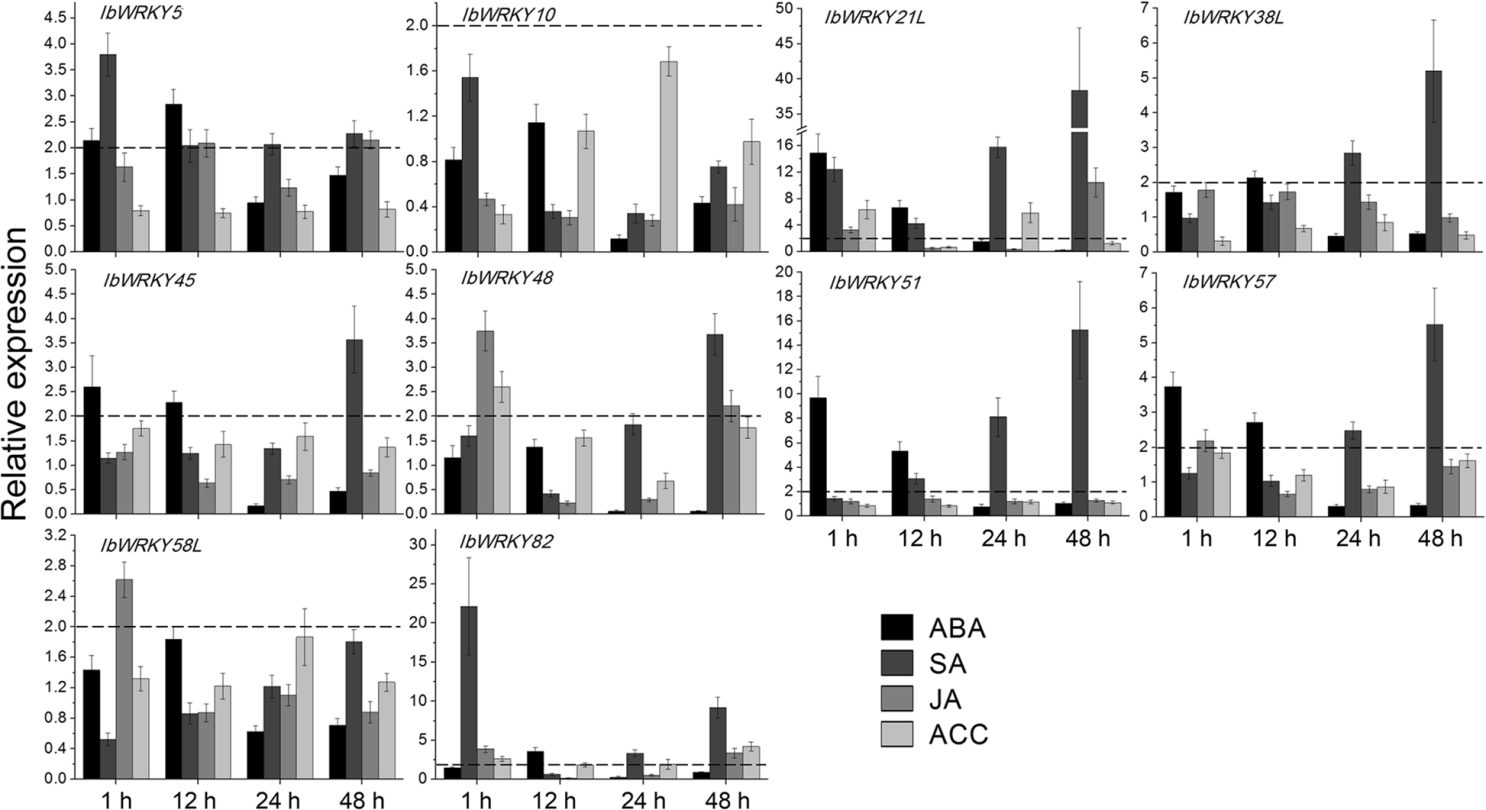
Relative gene expression of 10 *IbWRKYs* under hormone ABA, SA, JA and ACC treatments detected by qRT-PCR. The expression value at 0 h was normalized to 1, and the Y-axis delineates the expression fold changes comparing with 0 h. Bars represent the mean of three biological replicates ± SE. The two-fold thresholds are presented by dotted lines

qRT-PCR assays were also conducted to detect the transcriptions of ten *IbWRKYs* under different hormones, including ABA, SA, JA and ACC, which all play pivotal roles in plant defense responses to various stresses [38, 39]. Different degrees of up- or down-regulation were found after hormone treatments. Wherein, only the transcriptions of *IbWRKY21L* and *IbWRKY82* were induced by all hormones, while no obvious upregulation in the mRNA of *IbWRKY10* was observed when treated with these hormones. ABA and SA could enhance the expression of most detected *IbWRKYs*. Among them, *IbWRKY21L* and *IbWRKY51* were most significantly induced by both hormones at 9.7–38.3-fold under, while *IbWRKY-5/-38 L/-45/-57/-82* with a lesser extent of 2.1–5.5-fold. *IbWRKY21L* also displayed the highest induction after ACC and JA treatments with 6.3–10.4-fold. The transcript of five *IbWRKYs* (*IbWRKY-5/-48/-57/-58 L/-82*) was improved by JA treatment at a similar level (2.1–3.9-fold). Additionally, the expression of *IbWRKY48* and *IbWRKY82* also displayed an upregulated change (2.6–4.2-fold) post ACC (Fig. 8). The data suggested that multiple *IbWRKYs* might play significant roles in hormone signal transduction.

### Prediction of specific cis-elements in the promoters of *IbWRKYs*

**Fig. 9:**
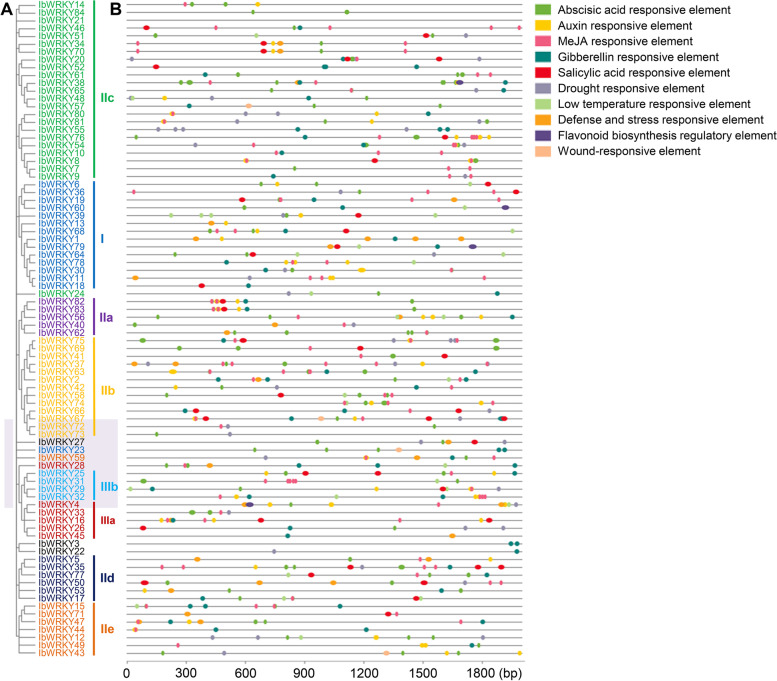
Predicted stress-, hormone- and flavonoid biosynthesis-related cis-elements in the promoters of *IbWRKY* genes. **A**. The phylogenetic tree of 84 IbWRKYs was constructed using the same parameters descripted in Fig. 1. **B**. Predicted cis-elements in the *IbWRKY* promoters. 2.0 kb promoter sequence of each *IbWRKY* gene was detected by PlantCARE database. Different colors represent specific cis-elements

To investigate the possible regulatory mechanism of *IbWRKYs* in response to stresses and hormones, the 2 kb promoter regions of each *IbWRKY* gene were scanned. As depicted in Fig. 9, totally 10 types of predicted cis-elements were discovered, including four stress-related elements, five hormone-related elements, and one flavonoid biosynthesis-related elements. The promoters of all *IbWRKYs* except *IbWRKY21* contained many potential stress- and/or hormone-related cis-elements. Among them, promoters of 64 *IbWRKYs* (76%) included stress-related ones, such as drought-, defense and stress-, low temperature-, and wound-responsive elements. And hormone-related cis-elements such as ABA-, IAA-, SA- and GA-responsive elements were discovered in the promoters of all *IbWRKYs* except *IbWRKY21* (Fig. 9, Additional file 10). Therefore, these predicted cis-element indicated that the expression of *IbWRKYs* might be associated with different abiotic stresses and hormones. For example, multiple stress-related cis-elements were found in the promoters of the detected stress-responsive genes except *IbWRKY-10/-21/-82*. Nonetheless, in-depth functional validation is necessary to confirm the roles of these predicted cis-elements.

### Analysis of protein interaction, subcellular localization, and transactivation activity of selected IbWRKYs

**Fig. 10:**
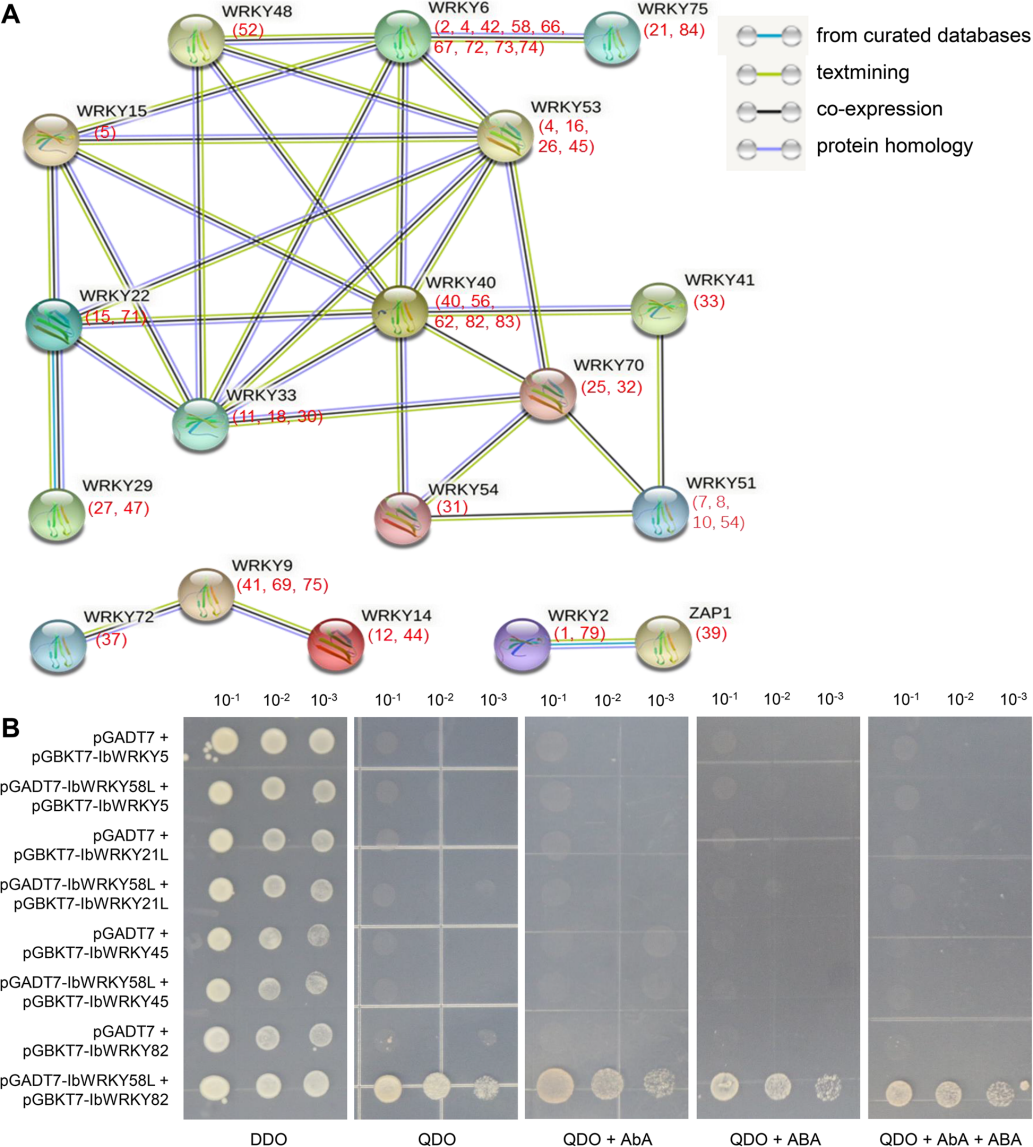
Interaction networks of IbWRKYs in sweetpotato and yeast two-hybrid experiment validation. **A**. Interaction networks of IbWRKYs in sweetpotato predicted by STRING database, network nodes represent different proteins. The numbers in brackets indicate the corresponding homologous IbWRKY protein(s) in sweetpotato. **B**. Protein interaction validation between IbWRKY58L and IbWRKY-5/-21 L/-45/-82 by yeast two-hybrid. Different interaction was detected on DDO (SD/-Trp-Leu), QDO (SD/-Trp-Leu-His-Ade), QDO with 200 ng/mL AbA, QDO with 20 µM ABA, and QDO with 200 ng/mL AbA + 20 µM ABA. The yeast cells were recorded 3 days after 30° of incubation

Multiple reports showed that WRKY TFs often function by forming homologous or heterologous protein complexes [23]. To gain insights into the potential relationship between IbWRKYs, a protein-protein interaction network was accomplished according to the orthologs of *Arabidopsis* AtWRKYs (Fig. 10 A). Complex interaction relationships suggested that multiple IbWRKY members might form protein dimer complexes. To validate the protein-protein interactions, the interaction between IbWRKY58L and IbWRKY-5/-21 L/-45/-82 identified in qRT-PCR analysis was tested by constructing the recombinant pGBKT7 or pGADT7 plasmids using yeast two-hybrid assay (Y2H) assay based on the interaction network. The results showed that all transformed yeasts could normally grow on DDO medium, while co-transformed constructs containing pGADT7-IbWRKY58L and pGBKT7 control or pGBKT7-IbWRKY-5/-21 L/-45 all could not survive on QDO and QDO with AbA medium, and only IbWRKY82 could directly interact with IbWRKY58L. Moreover, we found that all the tested interactions in yeasts were not affected by exogenous ABA (Fig. 10B).

**Fig. 11:**
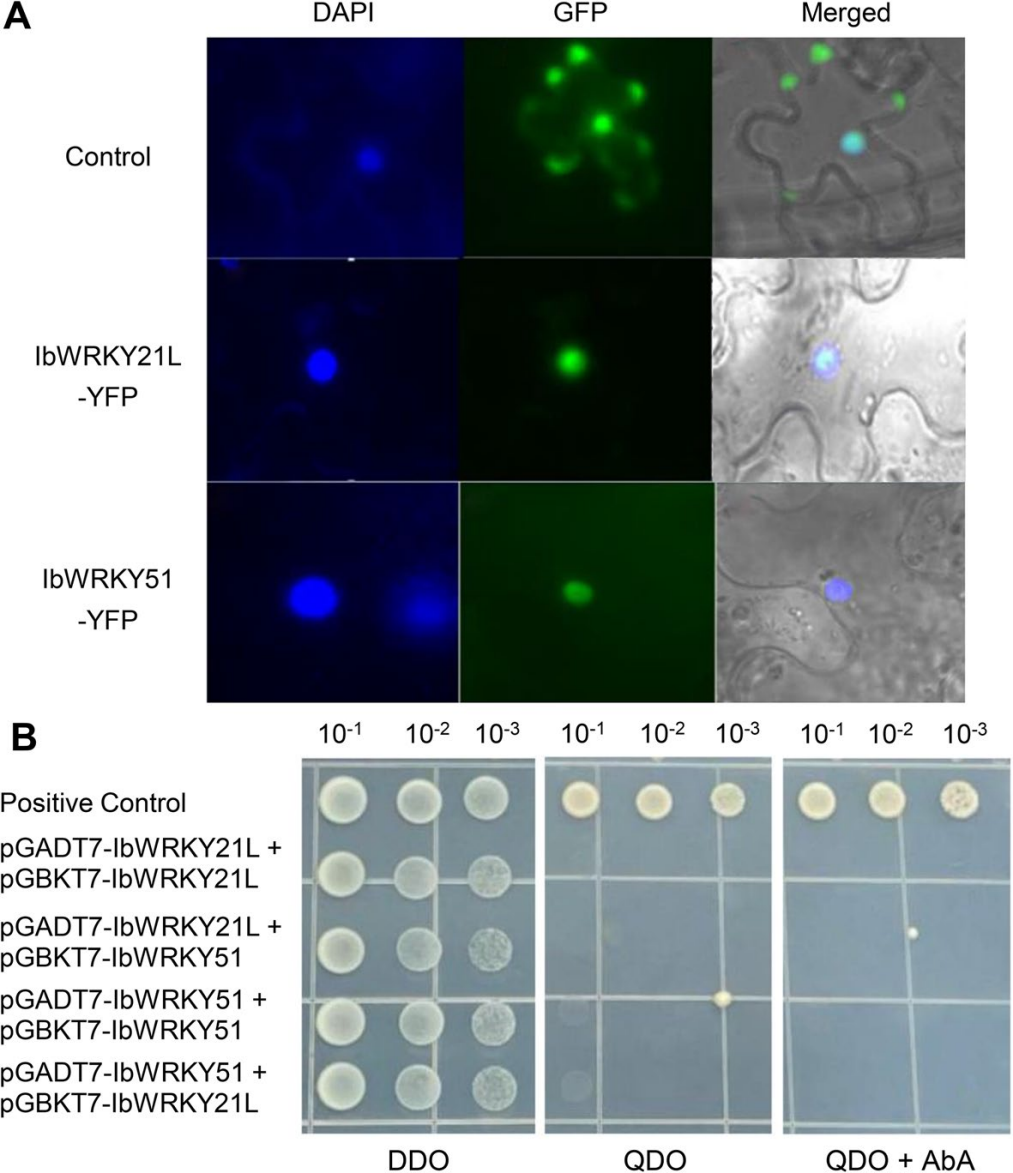
Subcellular localization, transactivation activity and protein interaction of IbWRKY21L and IbWRKY51. **A**. Localization of IbWRKY21L/IbWRKY51-YFP fusion protein in tobacco cells, the YFP signaling was visualized using a microscope. **B**. Transactivation activity and protein interaction of IbWRKY21L and IbWRKY51 in yeast cells. The activity and interaction were tested on DDO (SD/-Trp-Leu), QDO (SD/-Trp-Leu-His-Ade) and QDO with 200 ng/mL AbA. The yeast cells were recorded 3 days after 30° of incubation

Besides, considering that the transcription of *IbWRKY21L and IbWRKY51* genes was most significantly induced by multiple stresses, their molecular characteristics were further explored. Bioinformatics analysis showed that IbWRKY21L and IbWRKY51 TFs localized in the nucleus (Additional file 2), then a recombinant pEarleyGate101 vector was constructed with a translational fusion of IbWRKY-21 L/-51 to YFP to verify the results. The free YFP control displayed fluorescence in both the cytoplasm and the nucleus, while the observed IbWRKY-21 L/-51-YFP fusion proteins were exclusively located in the nucleus (Fig. 11 A). Further, their transactivation activities and interaction relationships were also identified. The results showed that all transformed yeasts could not survive on QDO and QDO with AbA medium, suggesting that IbWRKY21L and IbWRKY51 proteins have no transactivation activity in yeasts, and IbWRKY21L can not interact with IbWRKY51 and itself, nor can IbWRKY51 interact with itself (Fig. 11B). The data provide a reference for predicting potential pathways by which IbWRKY TFs regulate the response to environmental stresses.

## Discussion

WRKY TFs have broad application prospects as promising molecular diagnostics in enhancing crop improvements [20, 23]. For instance, overexpression of *OsWRKY47* not only improved drought tolerance of transgenic rice but also raised its yield [40]. However, genome-wide comprehensive investigation and expression analysis under different abiotic stresses of WRKY TFs in important sweetpotato crops are lacking. In this study, 84 *IbWRKYs* were systematically analyzed and their various molecular characterizations were further performed, which will improve the interpretation of the molecular functions and regulatory modes of IbWRKYs.

### Diverse characterizations of IbWRKY TFs

84 *IbWRKY* genes were identified in this study, which were unevenly distributed across the sweetpotato genome and varied even within chromosomes. Previous data showed that WRKY gene family was undergoing rapid expansion following polyploidization in the autopolyploid *Saccharum spontaneum* [41]. However, similar numbers of *ItfWRKY* genes (83 members) were isolated from *Ipomoea trifida*, which is the closest diploid wild ancestor of sweetpotato [42]. This might be caused by the limitation of half haplotype-resolved hexaploid genomes of sweetpotato [34]. Gene structures and conserved motifs of WRKY TFs provided crucial clues for exploring their evolutionary relationships [13, 17]. Gene structures and motifs of *IbWRKYs* were distributed in a phylogenetic relationship-specific manner for the members of close subgroup, and most *IbWRKYs* contained two/three exons, which were commonplace in many plants, such as rice and *Arabidopsis* [35], maize [16], *Liriodendron chinense* [43], and *setaria* [44].

Most IbWRKYs share the highly conserved heptapeptide WRKYGQK and C_2_H_2_ or C_2_HC type zinc finger domains. However, two variations including WRKYGTK and WRKYGKK, and no or only fragmentary zinc finger motifs were also observed. Earlier studies have also reported similar variations. For instance, 19 variants were detected in rice WRKYs, of which WRKYGEK and WRKYGKK were two common variants [45]. Phenomenon with such variation was also found in WRKYs of *Arabidopsis*, cucumber, maize, and banana [13, 16, 17, 35]. Moreover, the incomplete zinc finger motifs in IbWRKYs was consistent with earlier studies conducted on cotton [14], *setaria* [44] and soybean [18]. Differently, unlike previously reported numerous WRKYs, unique C_X6_C_X23_H_X_H zinc finger backbones were found in two IbWRKYs. Such rare variants, like C_X6_C_X28_H_X_C in rice OsWRKY68 [46] and C_X6_C_X23_H_X_C in wheat TaWRKY-101/-110/-121 [47] have also been found before. Structural classifications of IbWRKYs were supported by their evolutionary relationships, and two unique members IbWRKY3 and IbWRKY22 were divided as a specific branch, whether this implied that they exerted new functions remained to be elucidated. The data suggested that WRKY TF family might had gone through domain loss or acquisition events during the evolutionary process [16, 45].

Presence of lots of duplicated blocks indicated duplication occurred during the evolution and expansion of WRKY gene family [41]. Fragment duplications were shown to play prominent roles in sweetpotato *IbWRKY* gene expansion. Similar situations were also found in maize [16]. Contrarily, duplication events suggested that tandem duplications played major roles in *Liriodendron chinense* WRKY gene expansion [43]. In addition, 68 *IbWRKYs* had putative orthologs in *Ipomoea triloba*, which was significantly higher than in other plants tested, possibly due to the closer phylogenetic relationships between sweetpotato and *Ipomoea triloba*. Furthermore, we found five *IbWRKYs* to be syntenic with all tested plants except rice, implying that they likely derived from a common ancestor.

### The potential function and regulatory pathway of IbWRKYs in response to abiotic stress

WRKY TFs have received increasing attentions due to their extensive involvements in a variety of plant processes, especially the abiotic stress responses [20, 48]. In this study, the transcription of most tested *IbWRKY* genes was remarkably and differentially upregulated under various abiotic stresses. Many reports have revealed that stress-responsive WRKY genes are involved in the regulation of abiotic stress tolerance in a variety of plant species [20, 23]. For example, the *TaWRKY* family members exhibited diverse expression patterns under multiple abiotic stresses, including *TaWRKY75-A*, and its ectopic expression in *Arabidopsis* improved salt and drought tolerance [15]. Similarly, the expression of *IbWRKY2* (*IbWRKY79*) was upregulated by PEG6000 and NaCl, overexpression of *IbWRKY2* also enhanced the drought and salt tolerance of *Arabidopsis* [24]. Many studies demonstrated that the WRKY members of close subgroup might play similar roles in multiple abiotic stress response [16, 46, 49]. Recent findings revealed that WRKY members from multiple groups, including AtWRKY-8/-28 of subgroup IIc, AtWRKY39 of subgroup IId, and AtWRKY-30/-54/ -70 of group III were all associated with tolerance to various abiotic stresses [20, 24, 25]. Therefore, the expression of *IbWRKY-10*/-*21 L*/-*38 L*/-*48*/-*51*/-*57* genes in subgroup IIc, *IbWRKY5* in subgroup IId, and *IbWRKY45* in group III were significantly enhanced by different stresses, implying that they might also modulate stress response pathways. Therefore, studying the involvements of *IbWRKYs* in abiotic stress regulation could provide valuable clues to reveal their potential roles in stress tolerance.

Many documents reveal that WRKY TFs are key regulators connecting plant hormone signaling in response to environmental stresses, such as ABA, JA and SA [20, 50]. These hormones play key roles in the defense response of plants to various pathogens and abiotic stresses [38, 39]. For instance, *Fortunella crassifolia* FcWRKY40 participated in ABA signaling pathways and positively modulated salt tolerance through directly binding to and activating the promoters of *FcSOS2* and *FcP5CS1* [51]. *WRKY39* was induced by SA and MeJA, and was positively co-regulated by SA and JA signaling pathways in responses to heat stress [50]. The transcriptions of seven *IbWRKY*s was evidently improved simultaneously by ABA and various abiotic stresses, suggesting that these *IbWRKYs* may regulate stress responses through ABA-dependent signaling pathways, while this remains to be further elucidated. Similar results were also observed in JA-, SA- and ACC-induced *IbWRKYs*. Furthermore, many cis-elements related to stress and hormone response in the *IbWRKY* promoters were uncovered, indicating that they might play necessary roles in response to stress and/or hormone signaling. In-depth exploration of these cis-elements and the corresponding *IbWRKYs* may provide worthy information on their regulatory mechanisms of stress response.

Increasing discoveries revealed that WRKY proteins physically interact with diverse proteins to form integral parts of functional networks that regulate in signaling, transcription and chromatin remodeling [23, 52]. In *Arabidopsis*, WRKY18, WRKY40 and WRKY60 formed a homodimer or heterodimer to change pathogen resistance [53]. In rice, OsWRKY51 and OsWRKY71 functionally interact to play synergistic roles in regulating ABA and GA signaling crosstalk [54]. Prediction of potential regulatory effects for IbWRKYs displayed that multiple IbWRKYs homologous to *Arabidopsis* WRKYs were set as the core node of the interaction network. The protein sequences of IbWRKY5, IbWRKY21L, IbWRKY45, IbWRKY58L and IbWRKY82 were similar to that of WRKY15/WRKY75/WRKY53/WRKY6/WRKY40, respectively. Subsequent yeast two-hybrid experiments showed that IbWRKY58L indeed interacted with IbWRKY82. Taken together, the potential regulatory mechanisms of IbWRKY TFs in responding to abiotic stresses are complex, the data offer a foundation for forecasting the possible ways for IbWRKY TFs to modulate the response to adverse environmental conditions.

## Conclusion

84 putative IbWRKY TFs were isolated and systematically characterized in sweetpotato, including their molecular characteristics, classifications, evolutionary relationships, gene structures, and conserved protein motifs. The detection of differential and remarkable expression profiles of *IbWRKYs* under abiotic stresses and hormones will lay the foundation for figuring out the signaling pathways in sweetpotato responses to abiotic stress. Multiple stress-induced *IbWRKYs* may be closely related to the transcriptional regulation of abiotic stress responses in sweetpotato, and diverse interactions among IbWRKYs were identified, thus the complicated co-expression of *IbWRKYs* in response to abiotic stresses was predictable. Further validation of the specific functions and regulatory mechanisms of the excellent candidate *IbWRKY* genes in stress tolerance is necessary.

## Methods

### Identification of putative WRKY genes in sweetpotato

The DNA and protein sequence data, and the GFF annotation information of hexaploid sweetpotato Taizhong6 were obtained from the *Ipomoea* Genome Hub database [34]. To screen the possible WRKY-encoding genes in the genome, two approaches were used. HMM files of WRKY (PF03106) from the Pfam database (http://pfam.xfam.org/) were first employed, 225 seed sequences (Additional file 11) were applied to identify the possible WRKYs by BLASTP programs utilizing a default parameter. Afterwards, the reported protein sequences of 72 *Arabidopsis* AtWRKYs, 105 rice OsWRKYs and 79 sweetpotato WRKYs were got from the TAIR database (https://www.arabidopsis.org/), Rice Genome Annotation database (http://rice.plantbiology.msu.edu/) and published documents [33, 35] (Additional file 3). These protein sequences were then applied as inquires to search the sweetpotato protein database by BLASTP programs using a default parameter. All the candidates containing a WRKY domain were then detected by NCBI Batch CD-Search programs. In the end, overlapping or defective sequences were manually excluded, and 84 non-redundant candidates were considered as putative WRKYs and used for downstream analysis. The sequences of 84 IbWRKYs are presented in Additional file 12.

### Sequence characterization analysis of IbWRKYs

The physical and chemical characteristics of 84 IbWRKYs, including the length of amino acid residues, molecular weight (kDa) and isoelectric point (pI) of each IbWRKY were estimated by the ExPASy tool by a default parameter. Subcellular localizations were predicted through the WoLF PSORT (https://wolfpsort.hgc.jp/).

### Analysis of gene structures and conserved motifs

Exon-intron structures were visualized by Tbtools software v1.0971 [55] using the GFF annotations of sweetpotato genome information. The conserved motif compositions of IbWRKYs were analyzed by MEME 5.4.1 [56]. The parameters were set as follows: maximum motif number: 20, minimum motif width: 10, maximum motif width: 30, default for others.

### Sequence alignment and phylogenetic classification

Phylogenetic tree was constructed using complete amino acid sequences of 84 sweetpotato IbWRKYs and 72 *Arabidopsis* AtWRKYs (Additional file 3). Multiple sequence alignment of these sequences was conducted through the ClustalW program with a default parameter, the obtained result was applied to generate the un-rooted phylogenetic tree through the Maximum Likelihood method by the MEGA-X [57]. The best model JTT + G + I + F calculated by MEGA-X was adopted with a bootstrap value of 1000, and a phylogenetic analysis of 84 IbWRKYs was also conducted by same parameters. The classification of 84 IbWRKYs refers to the report in *Arabidopsis* [35]. Additionally, the conserved WRKY domains of IbWRKYs in different subgroups were extracted and aligned by DNAMAN 9 software.

### Chromosomal mapping and synteny analysis of IbWRKYs

Chromosomal locations of 84 *IbWRKYs* were accomplished using GFF annotations of sweetpotato genomes according to each starting and ending position. Tandem duplication and segmental duplication of *IbWRKYs* were identified using MCScanX by a default parameter [58]. Synteny analysis between *IbWRKYs* and associated genes from seven plants including *Ipomoea triloba*, *Arabidopsis*, rice, tomato, pepper, cabbage and *Brassica oleracea* was also carried out using MCScanX. The genome sequences of these plants were got from TAIR, Ensembl and Phytozome databases. Circos and TBtools softwares are employed to display the results, and the block sizes were set to 30 [55, 59].

### Screening of salt-responsive IbWRKYs by. transcriptome sequencing and analysis of gene expression under abiotic stress and hormone treatments by qRT-PCR

The adventitious root of salt-tolerant cultivar XuShu22 and salt-sensitive cultivar XuShu32 was treated with salt stress and used for RNA-seq detection. Differentially expressed genes were evaluated by read counts based on false discovery rate (FDR) < 0.05 and |Log2 (fold change)| > 1 as descripted in our previous report [37, 60]. Gene annotations were mainly according to the sweetpotato reference genome information, NCBI Nr and SwissProt databases. Afterwards, the key word ‘WRKY’ was used as a query to search against the annotation database, and NCBI Batch CD-Search was employed to validate each salt-responsive *IbWRKY* gene using default parameters.

For abiotic stress and hormone treatments, uniform XuShu22 plants were employed, detailed protocols for different abiotic stress and phytohormone treatments to plants are described before [61]. Total RNAs were prepared from different samples by the RNA Extraction Kit (TianGen) in accordance with the instructions. Then 1 µg RNA was reverse transcribed through TransScript one-step gDNA removal and cDNA synthesis mix (TransGen). To carry on the expression analysis, qRT-PCR was conducted on a CFX9 Real-Time detection System (Bio-Rad, USA). PCR reactions using a SYBR solution as descripted previously [62]. The sweetpotato *ARF* gene (JX177359) was used as the internal control gene [63]. All the qRT-PCR primers are presented in Additional file 13.

### Analysis of the cis-elements in *IbWRKY* promoters and protein interacting network

To survey the potential hormone- and stress-related cis-regulatory elements of the promoter sequences, the plantCARE database (http://bioinformatics.psb.ugent.be/webtools/plantcare/html/) was employed to scan the 2 kb promoter sequences of 84 *IbWRKY* genes retrieved from sweetpotato genome database. The functional protein interacting network was determined in the STRING 11.0 (https://string-db.org/).

### Transactivation activity, protein interaction, and subcellular localization of IbWRKY proteins

The open reading frame sequences of *IbWRKY21L and IbWRKY51* genes were isolated from XuShu22 cultivar and were separately inserted into the pEarleyGate101 vector using the Gateway method (Invitrogen). Agrobacterium strain (GV3101) harbouring pEarleyGate101-*IbWRKY* plasmids was cultivated and resuspended in a buffer (10 mM MES, pH 5.6; 10 mM MgCl_2_ and 100 µM acetosyringone). The strain was incubated at 28 °C for two hours, then injected into *N. benthamiana* leaves and expressed for two-three days. Subsequently, each IbWRKY*-*YFP expression was visualized using a fluorescence microscope (Olympus IX71S8F-3, Japan).

For transactivation activity and protein interaction analysis, the open reading frame sequences of *IbWRKY-5/-21 L/-45/-58 L/-82* genes were inserted into the activation domain vector pGADT7 or DNA binding vector pGBKT7, respectively, using the Gateway (Invitrogen) or homologous recombination method. The pGBKT7 control plasmid, each recombined pGBKT7-IbWRKY vector, or both recombined pGBKT7-IbWRKY and pGADT7-IbWRKY vectors were transformed into Y2HGold yeasts as descripted before [8, 64]. For self-activation detection, serial dilutions of transformed yeasts were dropped on SD/-Trp and SD/-Trp-His-Ade medium with or without AbA (200 ng/mL). For protein interactions, serial dilutions of transformed yeasts were dropped on SD/-Trp-Leu and SD/-Trp-Leu-His-Ade medium with or without AbA (200 ng/mL)/20 µM ABA. All the transformed yeasts were cultivated at 30 °C for three days to check their survival and growth. The related primers information is presented in Additional file 13.

### Data analysis

In order to strictly screen for the stress-induced *IbWRKYs*, a cut-off value of two-fold was adopted [8, 65]. OriginPro 8 (SAS Institute) was utilized to generate figures.

## Supplementary Information

**Additional file 1.**

**Additional file 2.**

**Additional file 3.**

**Additional file 4.**

**Additional file 5.**

**Additional file 6.**

**Additional file 7.**

**Additional file 8.**

**Additional file 9.**

**Additional file 10.**

**Additional file 11.**

**Additional file 12.**

**Additional file 13.**

## Data Availability

Most data analysed in this study are included in this published article and its supplementary information files. The RNA-seq data used in this study are available in the NCBI database (https://www.ncbi.nlm.nih.gov/biosample?LinkName=bioproject_biosample_all&from_uid=631585, accession numbers SAMN14884352-SAMN14884363).
