# Correction: A systematical genome-wide analysis and screening of WRKY transcription factor family engaged in abiotic stress response in sweetpotato

**DOI:** 10.1186/s12870-023-04047-8

**Published:** 2023-01-19

**Authors:** Siyuan Liu, Chengbin Zhang, Fen Guo, Qing Sun, Jing Yu, Tingting Dong, Xin Wang, Weihan Song, Zongyun Li, Xiaoqing Meng, Mingku Zhu

**Affiliations:** 1 Institute of Integrative Plant Biology, School of Life Sciences, Jiangsu Normal University, Xuzhou, 221116 Jiangsu Province China; 2 Agricultural Bureau of Linyi City, Linyi, 276000 Shandong Province China; 3 Jiangsu Key laboratory of Phylogenomics & Comparative Genomics, School of Life Sciences, Jiangsu Normal University, Xuzhou, 221116 Jiangsu Province China; 4 Jiangsu Xuzhou Sweetpotato Research Center, Xuzhou, 221131 Jiangsu Province China

**Correction:** ***BMC Plant Biol*** **22, 616 (2022)**

**https://doi.org/10.1186/s12870-022-03970-6**

Following the publication of the original article [1], the authors reported that Siyuan Liu, Chengbin Zhang, and Fen Guo were not added as equal contributors of this article. An article not has been added below:

- †Siyuan Liu, Chengbin Zhang and Fen Guo contributed equally to this work

The correction does not have any effect on the results or conclusions of the paper. The original article [1] has been corrected.
