# Supplementary material for: A systematical genome-wide analysis and screening of WRKY transcription factor family engaged in abiotic stress response in sweetpotato
Source: BMC Plant Biol. 2022 Dec 28;22:616. doi: 10.1186/s12870-022-03970-6 (PMC9795774; doi:10.1186/s12870-022-03970-6)
Supplement: Supplementary file 3 — Additional file 3. [file 12870_2022_3970_MOESM3_ESM.docx]

## **Additional file 13**. Specific primer sequences used for gene cloning, qRT-PCR analysis and vector construction.

| Primer code | Primer sequences (5' →3') | Application |
| --- | --- | --- |
| *IbARF-Q-F* | CTTTGCCAAGAAGGAGATGC | Internal standard gene for qRT-PCR analysis |
| *IbARF-Q-R* | CTTGTCCTGACCACCAACA |  |
| *IbWRKY5-Q-F* | CTTTTCTCACAGCCACTACTACCTC | qRT-PCR analysis for *IbWRKY* genes |
| *IbWRKY5-Q-R* | TAGTCTTTGTTCTATTCACCACCATC |  |
| *IbWRKY10-Q-F* | TGGAATGCTGCCCACAAATAC |  |
| *IbWRKY10-Q-R* | CAATATCTCAAGCTCAGTCTTCGTC |  |
| *IbWRKY21L-Q-F* | AATGAAGGGGTTGTTGGAGC |  |
| *IbWRKY21L-Q-R*  *IbWRKY38L-Q-F*  *IbWRKY38L-Q-R* | CTCCTGGTTTGGAACGCATAC  CCAGTTTTACCATGATCACACCG  CTGGGTGCGGGAGATTCG |  |
| *IbWRKY45-Q-F* | CGCCACCTCACAGATTCACA |  |
| *IbWRKY45-Q-R* | GGAGGACGAGAACCCGAAAC |  |
| *IbWRKY48-Q-F* | AAGGAGACCGAAGGAGAAGACG |  |
| *IbWRKY48-Q-R* | ATGAAGGCGAATCGTGGTTGT |  |
| *IbWRKY51-Q-F* | ATAGTATTGACTGGGTTGGGCTTC |  |
| *IbWRKY51-Q-R* | TCTTCCTCTTCCCTTTATTCTTCTG |  |
| *IbWRKY57-Q-F* | GAGAGAGAACGGAATCTGAATCG |  |
| *IbWRKY57-Q-R* | GCTTTCTTCCTTACTACGACCAA |  |
| *IbWRKY58L-Q-F* | TGGAGATTGAAATGGTGGCTG |  |
| *IbWRKY58L-Q-R* | GAACTTGCTGAATCGGCGTAG |  |
| *IbWRKY82-Q-F* | GCCCGTTCTTCAGCAGGTG |  |
| *IbWRKY82-Q-R* | CTATGGCGTCGGGTCAAACC |  |
| *IbWRKY5-c-F：*  *IbWRKY5-c-R：*  *IbWRKY21L-c-F* | TGGCATCATTTTGACTCTTGT  CGAGTATCACCCGAACTAAAAC  TCTTCACCAATTCACCCTCTC | Gene cloning and vector construction for *IbWRKY* genes |
| *IbWRKY21L-c-R* | TGAAATAAGGTAGCAAACAAAGC |  |
| *IbWRKY45-c-F*  *IbWRKY45-c-R*  *IbWRKY51-c-F* | ACTTGTTGAGTTTTGAATCCGA  TCATGTGAGAAACTCCAAGGTAT  TTGCTATACGTTGATGGAAGG |  |
| *IbWRKY51-c-R*  *IbWRKY58L-c-F*  *IbWRKY58L-c-R*  *IbWRKY82-c-F*  *IbWRKY82-c-R*  *IbWRKY5-BD-F：*  *IbWRKY5-BD-R:* | ATAGGGCGGATTAGAATCTTG  CACTTCAATTCATACCCGTTTC  CTTGGTTTTAAAGGATGTGGC  TTCATTAGCCCTTAGCACAAA  AACCTCCAAGTTTGTTACCCTA  TGGCCATGGAGGCCGAATTCATGGCAGTGGACCTTATGAT  CGCTGCAGGTCGACGGATCCAGAAGACTCTAAGATTAAACTGTTTG |  |
| *IbWRKY21L-F* | GGGGACAAGTTTGTACAAAAAAGCAGGCTTCATGGAGGCCTACCCAACACTG |  |
| *IbWRKY21L-R*  *bWRKY45-BD F*  *IbWRKY45-BD-R* | GGGGACCACTTTGTACAAGAAAGCTGGGTCAAAGGAGGCATAGATTTGCATCTG  TGGCCATGGAGGCCGAATTCATGGAAAGCGCTTATAACGG  CGCTGCAGGTCGACGGATCCAAGTTGCAGGGTGAAGTGAGTAG |  |
| *IbWRKY51-F* | GGGGACAAGTTTGTACAAAAAAGCAGGCTTCATGGAAGGAGAAGAGCCG |  |
| *IbWRKY51-R*  *IbWRKY58L-AD F：*  *IbWRKY58L-AD-R*  *IbWRKY82-BD-F*  *IbWRKY82-BD-R* | GGGGACCACTTTGTACAAGAAAGCTGGGTCGAATCTTGAGAGAAACTGAAGTTG  CATGGAGGCCAGTGAATTCATGGGGCTAACCCTTAAGAG  GCTCGAGCTCGATGGATCCTTTCGCCGGCAAACTAC  TGGCCATGGAGGCCGAATTCATGGGCTACTACAGTTCTAGCTTG  CGCTGCAGGTCGACGGATCCTGAGCATGAATCTGCGAGC |  |
