# Supplementary material for: A systematical genome-wide analysis and screening of WRKY transcription factor family engaged in abiotic stress response in sweetpotato
Source: BMC Plant Biol. 2022 Dec 28;22:616. doi: 10.1186/s12870-022-03970-6 (PMC9795774; doi:10.1186/s12870-022-03970-6)
Supplement: Supplementary file 4 — Additional file 4. [file 12870_2022_3970_MOESM4_ESM.docx]

**Additional file 2**. Characteristics of 84 IbWRKY proteins in sweetpotato. The asterisk indicates that the deficiency of two conserved WRKYGQKs despite two WRKY domains were identified.

| Gene name | Gene ID | Amino acids | MW (Da) | PI | Subcellular  localization | Conserved motif | Domain pattern | Zinc finger |
| --- | --- | --- | --- | --- | --- | --- | --- | --- |
| g34.t1 | IbWRKY1 | 680 | 73357.51 | 5.6 | Nucleus | 2(WRKYGQK^*^) | C-X4-C-X22-HNH/  C-X4-C-X23-HNH | C2H2 |
| g204.t1 | IbWRKY2 | 471 | 51128.56 | 6.18 | Nucleus | WRKYGQK | C-X5-C-X23-HNH | C2H2 |
| g907.t1 | IbWRKY3 | 339 | 37259.49 | 6.11 | Nucleus | WRKYGTK | C-X6-C-X23-HNH | C2H2 |
| g933.t1 | IbWRKY4 | 358 | 39322.04 | 5.48 | Nucleus | WRKYGQK | C-X7-C-X23-HTC | C2HC |
| g1093.t1 | IbWRKY5 | 314 | 33919.58 | 9.82 | Nucleus | WRKYGQK | C-X5-C-X23-HNH | C2H2 |
| g1282.t1 | IbWRKY6 | 560 | 61487.24 | 7.18 | Nucleus | 2(WRKYGQK) | C-X4-C-X22-HNH/  C-X4-C-X23-HNH | C2H2 |
| g2065.t1 | IbWRKY7 | 205 | 23146.73 | 6.58 | Nucleus | WRKYGKK | - | - |
| g2068.t1 | IbWRKY8 | 207 | 23242.46 | 9.15 | Chloroplast thylakoid membrane | WRKYGKK | - | - |
| g2069.t1 | IbWRKY9 | 193 | 21990.3 | 6.03 | Nucleus | WRKYGKK | - | - |
| g2071.t1 | IbWRKY10 | 185 | 21379.74 | 7.75 | Nucleus | WRKYGKK | C-X4-C-X23-HNH | C2H2 |
| g5064.t1 | IbWRKY11 | 445 | 48751.67 | 7.17 | Nucleus | 2(WRKYGQK) | C-X4-C-X22-HNH/  C-X4-C-X23-HNH | C2H2 |
| g5534.t1 | IbWRKY12 | 235 | 26748.57 | 6.72 | Nucleus | WRKYGQK | C-X5-C-X23-HNH | C2H2 |
| g8790.t1 | IbWRKY13 | 406 | 44344.19 | 8.96 | Nucleus | 2(WRKYGQK) | C-X4-C-X23-XXX/  C-X4-C-X23-HNH | C2XX/  C2H2 |
| g9098.t1 | IbWRKY14 | 215 | 23517.91 | 9.05 | Nucleus | WRKYGQK | - | - |
| g9355.t1 | IbWRKY15 | 332 | 36721.25 | 5.54 | Nucleus | WRKYGQK | C-X5-C-X23-HNH | C2H2 |
| g9401.t1 | IbWRKY16 | 345 | 38247.65 | 5.1 | Nucleus | WRKYGQK | C-X7-C-X23-HSC | C2HC |
| g9614.t1 | IbWRKY17 | 318 | 35411.98 | 9.49 | Nucleus | WRKYGQK | C-X5-C-X23-HNH | C2H2 |
| g9904.t1 | IbWRKY18 | 515 | 56299.67 | 6.23 | Nucleus | 2(WRKYGQK) | C-X4-C-X22-HNH/  C-X4-C-X23-HNH | C2H2 |
| g12300.t1 | IbWRKY19 | 588 | 63540.39 | 7.99 | Nucleus | 2(WRKYGQK) | C-X4-C-X22-HNH/  C-X4-C-X23-HNH | C2H2 |
| g13503.t1 | IbWRKY20 | 332 | 36548.76 | 7.71 | Nucleus | WRKYGQK | C-X4-C-X23-HNH | C2H2 |
| g13583.t1 | IbWRKY21 | 184 | 20529.22 | 9.27 | Nucleus | WRKYGQK | C-X4-C-X23-HNH | C2H2 |
| g13719.t1 | IbWRKY22 | 248 | 27948.93 | 5.28 | Chloroplast, nucleus | WRKYGKK | C-X6-C-X22-HNH | C2H2 |
| g16510.t1 | IbWRKY23 | 477 | 51559.16 | 6.62 | Nucleus | 2(WRKYGQK) | C-X4-C-X22-HNH/- | C2H2/- |
| g17895.t1 | IbWRKY24 | 278 | 31330.89 | 4.93 | Nucleus | WRKYGQK | C-X4-C-X23-HNH | C2H2 |
| g18302.t1 | IbWRKY25 | 282 | 31805.56 | 6.31 | Nucleus | WRKYGQK | C-X7-C-X23-HTC | C2HC |
| g19571.t1 | IbWRKY26 | 265 | 30097.4 | 5.58 | Nucleus | WRKYGQK | C-X7-C-X23-HTC | C2HC |
| g19636.t1 | IbWRKY27 | 261 | 28714.08 | 5.62 | Chloroplast, nucleus | WRKYGQK | - | - |
| g20451.t1 | IbWRKY28 | 323 | 34677.66 | 5.76 | Nucleus | WRKYGQK | C-X7-C-X23-HRC | C2HC |
| g20452.t1 | IbWRKY29 | 310 | 34616.86 | 5.29 | Nucleus | WRKYGQK | C-X7-C-X21-HTC | C2HC |
| g24541.t1 | IbWRKY30 | 574 | 63809.7 | 7.64 | Nucleus | 2(WRKYGQK) | C-X4-C-X22-HNH/  C-X4-C-X23-HNH | C2H2 |
| g24598.t1 | IbWRKY31 | 307 | 33970.64 | 6.08 | Nucleus | WRKYGQK | C-X7-C-X23-HTC | C2HC |
| g25439.t1 | IbWRKY32 | 225 | 25399.49 | 9.31 | Nucleus | WRKYGQK | C-X7-C-X21-HTC | C2HC |
| g26929.t1 | IbWRKY33 | 291 | 32110.35 | 4.89 | Nucleus | WRKYGQK | C-X7-C-X42-HSH | C2H2 |
| g28114.t1 | IbWRKY34 | 261 | 29336.02 | 9.32 | Chloroplast, nucleus | WRKYGQK | C-X4-C-X23-HNH | C2H2 |
| g28911.t1 | IbWRKY35 | 321 | 34696.34 | 9.51 | Nucleus | WRKYGQK | C-X5-C-X23-HNH | C2H2 |
| g30061.t1 | IbWRKY36 | 838 | 93900.94 | 6.93 | Chloroplast | 2(WRKYGQK) | -/C-X4-C-X23-HNH | -/C2H2 |
| g30102.t1 | IbWRKY37 | 505 | 54273.32 | 7.59 | Nucleus | WRKYGQK | C-X5-C-X23-HNH | C2H2 |
| g30666.t1 | IbWRKY38 | 351 | 39015.45 | 6.7 | Nucleus | WRKYGQK | C-X4-C-X23-HNH | C2H2 |
| g30984.t1 | IbWRKY39 | 537 | 59289.63 | 6.17 | Nucleus | 2(WRKYGQK) | C-X4-C-X22-HEH/  C-X4-C-X23-HDH | C2H2 |
| g31550.t1 | IbWRKY40 | 339 | 37388.71 | 9.19 | Nucleus | WRKYGQK | C-X5-C-X23-HNH | C2H2 |
| g34309.t1 | IbWRKY41 | 432 | 47492.48 | 6.26 | Nucleus | WRKYGQK | C-X5-C-X23-HNH | C2H2 |
| g34420.t1 | IbWRKY42 | 572 | 61135.6 | 8.12 | Nucleus, chloroplast | WRKYGQK | C-X5-C-X23-HNH | C2H2 |
| g34688.t1 | IbWRKY43 | 274 | 30213.89 | 10.74 | Chloroplast | WRKYGQK | C-X5-C-X23-HNH | C2H2 |
| g34938.t1 | IbWRKY44 | 451 | 48304.57 | 5.51 | Nucleus | WRKYGQK | C-X5-C-X23-HNH | C2H2 |
| g35097.t1 | IbWRKY45 | 335 | 38606.6 | 8.94 | Nucleus | WRKYGQK | C-X7-C-X23-HTC | C2HC |
| g35173.t1 | IbWRKY46 | 189 | 21263.32 | 9.32 | Nucleus | WRKYGQK | C-X4-C-X23-HNH | C2H2 |
| g35174.t1 | IbWRKY47 | 296 | 33105.14 | 5.47 | Nucleus | WRKYGQK | C-X5-C-X23-HCH | C2H2 |
| g37414.t1 | IbWRKY48 | 334 | 36413.27 | 6.56 | Nucleus | WRKYGQK | C-X4-C-X23-HNH | C2H2 |
| g37639.t1 | IbWRKY49 | 251 | 27516.18 | 5.42 | Nucleus | WRKYGQK | C-X5-C-X23-HNH | C2H2 |
| g38705.t1 | IbWRKY50 | 311 | 33279.67 | 9.31 | Nucleus | WRKYGQK | C-X5-C-X23-HHH | C2H2 |
| g39363.t1 | IbWRKY51 | 192 | 21532.63 | 9.08 | Peroxisome, nucleus | WRKYGQK | C-X4-C-X23-HNH | C2H2 |
| g41639.t1 | IbWRKY52 | 391 | 43133.01 | 6.36 | Nucleus | WRKYGQK | C-X4-C-X23-HTH | C2H2 |
| g41910.t1 | IbWRKY53 | 342 | 36489.17 | 9.49 | Nucleus | WRKYGQK | C-X5-C-X23-HNH | C2H2 |
| g42183.t1 | IbWRKY54 | 218 | 24676.46 | 7.57 | Nucleus | WRKYGKK | C-X4-C-X23-HDH | C2H2 |
| g43744.t1 | IbWRKY55 | 185 | 20387.48 | 5.96 | Cytoplasm | WRKYGKK | C-X4-C-X23-HNH | C2H2 |
| g44395.t1 | IbWRKY56 | 263 | 29678 | 5.43 | Nucleus | WRKYGQK | C-X5-C-X23-HNH | C2H2 |
| g45950.t1 | IbWRKY57 | 338 | 37355.3 | 6.37 | Nucleus | WRKYGQK | C-X4-C-X23-HDH | C2H2 |
| g46541.t1 | IbWRKY58 | 494 | 53674.4 | 9.73 | Nucleus | WRKYGQK | - | - |
| g46785.t1 | IbWRKY59 | 262 | 27838.95 | 5.58 | Nucleus | - | - | - |
| g47333.t1 | IbWRKY60 | 433 | 47020 | 6.16 | Nucleus | 2(WRKYGQK^*^) | -/C-X4-C-X23-HNH | -/C2H2 |
| g47361.t1 | IbWRKY61 | 306 | 32573.86 | 6.18 | Nucleus | WRKYGQK | C-X4-C-X23-HCH | C2H2 |
| g51746.t1 | IbWRKY62 | 334 | 36663.34 | 8.85 | Nucleus | WRKYGQK | C-X5-C-X23-HNH | C2H2 |
| g53553.t1 | IbWRKY63 | 594 | 64654.73 | 7.12 | Nucleus | WRKYGQK | C-X5-C-X23-HNH | C2H2 |
| g53997.t1 | IbWRKY64 | 826 | 90733.96 | 8.59 | Nucleus | 2(WRKYGQK) | C-X4-C-X22-HDH/  C-X4-C-X23-HNH | C2H2 |
| g54369.t1 | IbWRKY65 | 359 | 39881.87 | 6.37 | Nucleus | WRKYGQK | C-X4-C-X23-HNH | C2H2 |
| g54719.t1 | IbWRKY66 | 556 | 59861.06 | 7.23 | Nucleus | WRKYGQK | C-X5-C-X23-HNH | C2H2 |
| g54721.t1 | IbWRKY67 | 534 | 57960.83 | 6.75 | Nucleus | WRKYGQK | C-X5-C-X65-HGH | C2H2 |
| g54892.t1 | IbWRKY68 | 574 | 62619.43 | 5.33 | Nucleus | 2(WRKYGQK) | C-X4-C-X22-HNH/  C-X4-C-X23-HNH | C2H2 |
| g55476.t1 | IbWRKY69 | 423 | 47693.88 | 9.19 | Nucleus | WRKYGQK | C-X5-C-X23-HNH | C2H2 |
| g56425.t1 | IbWRKY70 | 261 | 29336.02 | 9.32 | Chloroplast, nucleus | WRKYGQK | C-X4-C-X23-HVH | C2H2 |
| g56897.t1 | IbWRKY71 | 352 | 38487.86 | 6.37 | Nucleus | WRKYGQK | C-X5-C-X23-HNH | C2H2 |
| g59084.t1 | IbWRKY72 | 482 | 51064.7 | 5.96 | Nucleus | WRKYGQK | C-X5-C-X38-HNH | C2H2 |
| g59087.t1 | IbWRKY73 | 541 | 57320.56 | 6.35 | Nucleus | WRKYGQK | C-X5-C-X23-HNH | C2H2 |
| g59354.t1 | IbWRKY74 | 548 | 59076.95 | 6.84 | Nucleus | WRKYGQK | C-X5-C-X23-HNH | C2H2 |
| g59463.t1 | IbWRKY75 | 441 | 48140.02 | 6.78 | Nucleus | WRKYGQK | C-X5-C-X23-HNH | C2H2 |
| g60157.t1 | IbWRKY76 | 169 | 18912.94 | 7.05 | Nucleus | WRKYGKK | - | - |
| g60387.t1 | IbWRKY77 | 345 | 37375.37 | 9.82 | Nucleus | WRKYGQK | C-X5-C-X23-HNH | C2H2 |
| g61234.t1 | IbWRKY78 | 471 | 51530.25 | 6.04 | Nucleus | 2(WRKYGQK) | C-X4-C-X22-HDH/- | C2H2/- |
| g61316.t1 | IbWRKY79 | 712 | 77088.72 | 5.98 | Nucleus | 2(WRKYGQK) | C-X4-C-X22-HNH/  C-X4-C-X23-HNH | C2H2 |
| g62149.t1 | IbWRKY80 | 162 | 18336.19 | 5.89 | Nucleus | WRKYGKK | - | - |
| g62313.t1 | IbWRKY81 | 177 | 19903.14 | 6.03 | Nucleus | WRKYGKK | - | - |
| g63584.t1 | IbWRKY82 | 294 | 32229.31 | 6.71 | Nucleus | WRKYGQK | C-X5-C-X23-HNH | C2H2 |
| g63694.t1 | IbWRKY83 | 276 | 30197.03 | 8.02 | Nucleus | WRKYGQK | - | - |
| g64087.t1 | IbWRKY84 | 120 | 13193.91 | 9.84 | Chloroplast | WRKYGQK | - | - |
