# Supplementary material for: A systematical genome-wide analysis and screening of WRKY transcription factor family engaged in abiotic stress response in sweetpotato
Source: BMC Plant Biol. 2022 Dec 28;22:616. doi: 10.1186/s12870-022-03970-6 (PMC9795774; doi:10.1186/s12870-022-03970-6)

**Additional file 8**. Venn diagrams among the detected species with orthologous genes of sweetpotato *IbWRKY* genes.


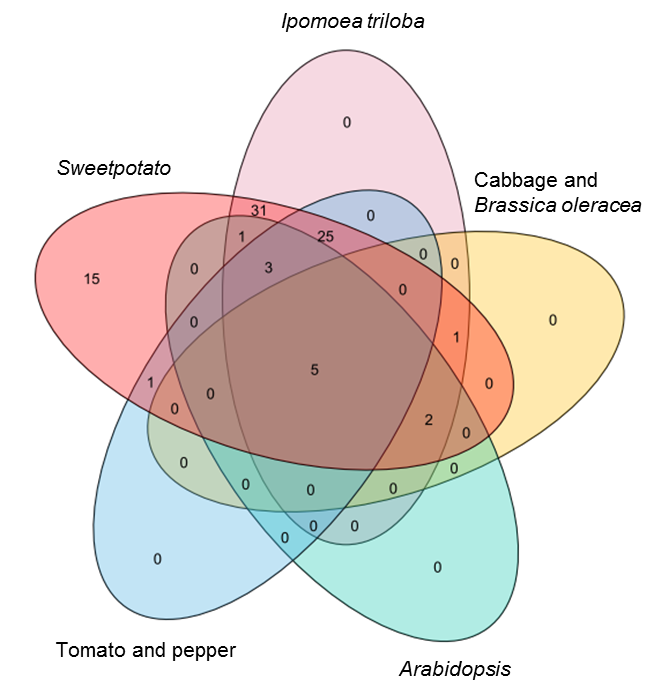

Supplement: Supplementary file 5 — Additional file 5. [file 12870_2022_3970_MOESM5_ESM.docx]
