# Supplementary material for: A systematical genome-wide analysis and screening of WRKY transcription factor family engaged in abiotic stress response in sweetpotato
Source: BMC Plant Biol. 2022 Dec 28;22:616. doi: 10.1186/s12870-022-03970-6 (PMC9795774; doi:10.1186/s12870-022-03970-6)
Supplement: Supplementary file 7 — Additional file 7. [file 12870_2022_3970_MOESM7_ESM.docx]

**Additional file 3.** A list of sequence information of reference *Arabidopsis*, rice and sweetpotato WRKYs used to access the data analyzed in this study.

*Arabidopsis* WRKYs

>AtWRKY1

MAEVGKVLASDMELDHSNETKAVDDVVATTDKAEVIPVAVTRTETVVESLESTDCKELEKLVPHTVASQSEVDVASPVSEKAPKVSESSGALSLQSGSEGNSPFIREKVMEDGYNWRKYGQKLVKGNEFVRSYYRCTHPNCKAKKQLERSAGGQVVDTVYFGEHDHPKPLAGAVPINQDKRSDVFTAVSKGEQRIDIVSLIYKLCIVSYDIMFVEKTSGSSVQTLRQTEPPKIHGGLHVSVIPPADDVKTDISQSSRITGDNTHKDYNSPTAKRRKKGGNIELSPVERSTNDSRIVVHTQTLFDIVNDGYRWRKYGQKSVKGSPYPRSYYRCSSPGCPVKKHVERSSHDTKLLITTYEGKHDHDMPPGRVVTHNNMLDSEVDDKEGDANKTPQSSTLQSITKDQHVEDHLRKKTKTNGFEKSLDQGPVLDEKLKEEIKERSDANKDHAANHAKPEAKSDDKTTVCQEKAVGTLESEEQKPKTEPAQS

>AtWRKY2

MAGFDENVAVMGEWVPRSPSPGTLFSSAIGEEKSSKRVLERELSLNHGQVIGLEEDTSSNHNKDSSQSNVFRGGLSERIAARAGFNAPRLNTENIRTNTDFSIDSNLRSPCLTISSPGLSPATLLESPVFLSNPLAQPSPTTGKFPFLPGVNGNALSSEKAKDEFFDDIGASFSFHPVSRSSSSFFQGTTEMMSVDYGNYNNRSSSHQSAEEVKPGSENIESSNLYGIETDNQNGQNKTSDVTTNTSLETVDHQEEEEEQRRGDSMAGGAPAEDGYNWRKYGQKLVKGSEYPRSYYKCTNPNCQVKKKVERSREGHITEIIYKGAHNHLKPPPNRRSGMQVDGTEQVEQQQQQRDSAATWVSCNNTQQQGGSNENNVEEGSTRFEYGNQSGSIQAQTGGQYESGDPVVVVDASSTFSNDEDEDDRGTHGSVSLGYDGGGGGGGGEGDESESKRRKLEAFAAEMSGSTRAIREPRVVVQTTSDVDILDDGYRWRKYGQKVVKGNPNPRSYYKCTAPGCTVRKHVERASHDLKSVITTYEGKHNHDVPAARNSSHGGGGDSGNGNSGGSAAVSHHYHNGHHSEPPRGRFDRQVTTNNQSPFSRPFSFQPHLGPPSGFSFGLGQTGLVNLSMPGLAYGQGKMPGLPHPYMTQPVGMSEAMMQRGMEPKVEPVSDSGQSVYNQIMSRLPQI

>AtWRKY3

MAEKEEKEPSKLKSSTGVSRPTISLPPRPFGEMFFSGGVGFSPGPMTLVSNLFSDPDEFKSFSQLLAGAMASPAAAAVAAAAVVATAHHQTPVSSVGDGGGSGGDVDPRFKQSRPTGLMITQPPGMFTVPPGLSPATLLDSPSFFGLFSPLQGTFGMTHQQALAQVTAQAVQGNNVHMQQSQQSEYPSSTQQQQQQQQQASLTEIPSFSSAPRSQIRASVQETSQGQRETSEISVFEHRSQPQNADKPADDGYNWRKYGQKQVKGSDFPRSYYKCTHPACPVKKKVERSLDGQVTEIIYKGQHNHELPQKRGNNNGSCKSSDIANQFQTSNSSLNKSKRDQETSQVTTTEQMSEASDSEEVGNAETSVGERHEDEPDPKRRNTEVRVSEPVASSHRTVTEPRIIVQTTSEVDLLDDGYRWRKYGQKVVKGNPYPRSYYKCTTPDCGVRKHVERAATDPKAVVTTYEGKHNHDVPAARTSSHQLRPNNQHNTSTVNFNHQQPVARLRLKEEQIT

>AtWRKY4

MSEKEEAPSTSKSTGAPSRPTLSLPPRPFSEMFFNGGVGFSPGPMTLVSNMFPDSDEFRSFSQLLAGAMSSPATAAAAAAAATASDYQRLGEGTNSSSGDVDPRFKQNRPTGLMISQSQSPSMFTVPPGLSPAMLLDSPSFLGLFSPVQGSYGMTHQQALAQVTAQAVQANANMQPQTEYPPPSQVQSFSSGQAQIPTSAPLPAQRETSDVTIIEHRSQQPLNVDKPADDGYNWRKYGQKQVKGSEFPRSYYKCTNPGCPVKKKVERSLDGQVTEIIYKGQHNHEPPQNTKRGNKDNTANINGSSINNNRGSSELGASQFQTNSSNKTKREQHEAVSQATTTEHLSEASDGEEVGNGETDVREKDENEPDPKRRSTEVRISEPAPAASHRTVTEPRIIVQTTSEVDLLDDGYRWRKYGQKVVKGNPYPRSYYKCTTPGCGVRKHVERAATDPKAVVTTYEGKHNHDLPAAKSSSHAAAAAQLRPDNRPGGLANLNQQQQQQPVARLRLKEEQTT

>AtWRKY6

MDRGWSGLTLDSSSLDLLNPNRISHKNHRRFSNPLAMSRIDEEDDQKTRISTNGSEFRFPVSLSGIRDREDEDFSSGVAGDNDREVPGEVDFFSDKKSRVCREDDEGFRVKKEEQDDRTDVNTGLNLRTTGNTKSDESMIDDGESSEMEDKRAKNELVKLQDELKKMTMDNQKLRELLTQVSNSYTSLQMHLVSLMQQQQQQNNKVIEAAEKPEETIVPRQFIDLGPTRAVGEAEDVSNSSSEDRTRSGGSSAAERRSNGKRLGREESPETESNKIQKVNSTTPTTFDQTAEATMRKARVSVRARSEAPMISDGCQWRKYGQKMAKGNPCPRAYYRCTMATGCPVRKQVQRCAEDRSILITTYEGNHNHPLPPAAVAMASTTTAAANMLLSGSMSSHDGMMNPTNLLARAVLPCSTSMATISASAPFPTVTLDLTHSPPPPNGSNPSSSAATNNNHNSLMQRPQQQQQQMTNLPPGMLPHVIGQALYNQSKFSGLQFSGGSPSTAAFSQSHAVADTITALTADPNFTAALAAVISSMINGTNHHDGEGNNKNQ

>AtWRKY7

MTVELMMSSYSGGGGGGDGFPAIAAAAKMEDTALREAASAGIHGVEEFLKLIGQSQQPTEKSQTEITAVTDVAVNSFKKVISLLGRSRTGHARFRRAPASTQTPFKQTPVVEEEVEVEEKKPETSSVLTKQKTEQYHGGGSAFRVYCPTPIHRRPPLSHNNNNNQNQTKNGSSSSSPPMLANGAPSTINFAPSPPVSATNSFMSSHRCDTDSTHMSSGFEFTNPSQLSGSRGKPPLSSASLKRRCNSSPSSRCHCSKKRKSRVKRVIRVPAVSSKMADIPSDEFSWRKYGQKPIKGSPHPRGYYKCSSVRGCPARKHVERALDDAMMLIVTYEGDHNHALVLETTTMNHDKTL

>AtWRKY8

MSHEIKDLNNYHYTSSYNHYNINNQNMINLPYVSGPSAYNANMISSSQVGFDLPSKNLSPQGAFELGFELSPSSSDFFNPSLDQENGLYNAYNYNSSQKSHEVVGDGCATIKSEVRVSASPSSSEADHHPGEDSGKIRKKREVRDGGEDDQRSQKVVKTKKKEEKKKEPRVSFMTKTEVDHLEDGYRWRKYGQKAVKNSPYPRSYYRCTTQKCNVKKRVERSYQDPTVVITTYESQHNHPIPTNRRTAMFSGTTASDYNPSSSPIFSDLIINTPRSFSNDDLFRVPYASVNVNPSYHQQQHGFHQQESEFELLKEMFPSVFFKQEP

>AtWRKY9

MGFDFSTSKSKAKRQKRIEVRFASPLMGIDLSLKLEAEEKKKEIEGSKHSRENKEDEEHDASGDEDEQMVKEDEDDSSSLGLRTREEENEREELLQLQIQMESVKEENTRLRKLVEQTLEDYRHLEMKFPVIDKTKKMDLEMFLGVQGKRCVDITSKARKRGAERSPSMEREIGLSLSLEKKQKQEESKEAVQSHHQRYNSSSLDMNMPRIISSSQGNRKARVSVRARCETATMNDGCQWRKYGQKTAKGNPCPRAYYRCTVAPGCPVRKQVQRCLEDMSILITTYEGTHNHPLPVGATAMASTASTSPFLLLDSSDNLSHPSYYQTPQAIDSSLITYPQNSSYNNRTIRSLNFDGPSRGDHVSSSQNRLNWMM

>AtWRKY10

MSDFDENFIEMTSYWAPPSSPSPRTILAMLEQTDNGLNPISEIFPQESLPRDHTDQSGQRSGLRERLAARVGFNLPTLNTEENMSPLDAFFRSSNVPNSPVVAISPGFSPSALLHTPNMVSDSSQIIPPSSATNYGPLEMVETSGEDNAAMMMFNNDLPYQPYNVDLPSLEVFDDIATEESFYIPSYEPHVDPIGTPLVTSFESELVDDAHTDIISIEDSESEDGNKDDDDEDFQYEDEDEDQYDQDQDVDEDEEEEKDEDNVALDDPQPPPPKRRRYEVSNMIGATRTSKTQRIILQMESDEDNPNDGYRWRKYGQKVVKGNPNPRSYFKCTNIECRVKKHVERGADNIKLVVTTYDGIHNHPSPPARRSNSSSRNRSAGATIPQNQNDRTSRLGRAPPTPTPPTPPPSSYTPEEMRPFSSLATEIDLTEVYMTGISMLPNIPVYENSGFMYQNDEPTMNAMPDGSDVYDGIMERLYFKFGVDM

>AtWRKY11

MAVDLMRFPKIDDQTAIQEAASQGLQSMEHLIRVLSNRPEQQHNVDCSEITDFTVSKFKTVISLLNRTGHARFRRGPVHSTSSAASQKLQSQIVKNTQPEAPIVRTTTNHPQIVPPPSSVTLDFSKPSIFGTKAKSAELEFSKENFSVSLNSSFMSSAITGDGSVSNGKIFLASAPLQPVNSSGKPPLAGHPYRKRCLEHEHSESFSGKVSGSAYGKCHCKKSRKNRMKRTVRVPAISAKIADIPPDEYSWRKYGQKPIKGSPHPRGYYKCSTFRGCPARKHVERALDDPAMLIVTYEGEHRHNQSAMQENISSSGINDLVFASA

>AtWRKY12

MEGGGRRVFSNYDLQQVTSSSTTIQENMNFLVPFEETNVLTFFSSSSSSSLSSPSFPIHNSSSTTTTHAPLGFSNNLQGGGPLGSKVVNDDQENFGGGTNNDAHSNSWWRSNSGSGDMKNKVKIRRKLREPRFCFQTKSDVDVLDDGYKWRKYGQKVVKNSLHPRSYYRCTHNNCRVKKRVERLSEDCRMVITTYEGRHNHIPSDDSTSPDHDCLSSF

>AtWRKY13

MGAINQGISLFDESQTVINPINTNHLGFFFSFPSHSTLSSSSSSSSSSPSSLVSPFLGHNSLNSFLHNNPSSFISHPQDSINLMTNLPETLISSLSSSKQRDDHDGFLNLDHHRLTGSISSQRPLSNPWAWSCQAGYGSSQKNNHGSEIDVDDNDDEVGDGGGINDDDNGRHHHHDTPSRHDKHNTASLGVVSSLKMKKLKTRRKVREPRFCFKTLSEVDVLDDGYRWRKYGQKVVKNTQHPRSYYRCTQDKCRVKKRVERLADDPRMVITTYEGRHLHSPSNHLDDDSLSTSHLHPPLSNFFW

>AtWRKY14

MCSVSELLDMENFQGDLTDVVRGIGGHVLSPETPPSNIWPLPLSHPTPSPSDLNINPFGDPFVSMDDPLLQELNSITNSGYFSTVGDNNNNIHNNNGFLVPKVFEEDHIKSQCSIFPRIRISHSNIIHDSSPCNSPAMSAHVVAAAAAASPRGIINVDTNSPRNCLLVDGTTFSSQIQISSPRNLGLKRRKSQAKKVVCIPAPAAMNSRSSGEVVPSDLWAWRKYGQKPIKGSPFPRGYYRCSSSKGCSARKQVERSRTDPNMLVITYTSEHNHPWPIQRNALAGSTRSSTSSSSNPNPSKPSTANVNSSSIGSQNTIYLPSSTTPPPTLSSSAIKDERGDDMELENVDDDDDNQIAPYRPELHDHQHQPDDFFADLEELEGDSLSMLLSHGCGGDGKDKTTASDGISNFFGWSGDNNYNNYDDQDSRSL

>AtWRKY15

MAVELMTRNYISGVGADSFAVQEAAASGLKSIENFIGLMSRDSFNSDQPSSSSASASASAAADLESARNTTADAAVSKFKRVISLLDRTRTGHARFRRAPVHVISPVLLQEEPKTTPFQSPLPPPPQMIRKGSFSSSMKTIDFSSLSSVTTESDNQKKIHHHQRPSETAPFASQTQSLSTTVSSFSKSTKRKCNSENLLTGKCASASSSGRCHCSKKRKIKQRRIIRVPAISAKMSDVPPDDYSWRKYGQKPIKGSPHPRGYYKCSSVRGCPARKHVERAADDSSMLIVTYEGDHNHSLSAADLAGAAVADLILESS

>AtWRKY16

MTESEQIVYISCIEEVRYSFVSHLSKALQRKGVNDVFIDSDDSLSNESQSMVERARVSVMILPGNRTVSLDKLVKVLDCQKNKDQVVVPVLYGVRSSETEWLSALDSKGFSSVHHSRKECSDSQLVKETVRDVYEKLFYMERIGIYSKLLEIEKMINKQPLDIRCVGIWGMPGIGKTTLAKAVFDQMSGEFDAHCFIEDYTKAIQEKGVYCLLEEQFLKENAGASGTVTKLSLLRDRLNNKRVLVVLDDVRSPLVVESFLGGFDWFGPKSLIIITSKDKSVFRLCRVNQIYEVQGLNEKEALQLFSLCASIDDMAEQNLHEVSMKVIKYANGHPLALNLYGRELMGKKRPPEMEIAFLKLKECPPAIFVDAIKSSYDTLNDREKNIFLDIACFFQGENVDYVMQLLEGCGFFPHVGIDVLVEKSLVTISENRVRMHNLIQDVGRQIINRETRQTKRRSRLWEPCSIKYLLEDKEQNENEEQKTTFERAQVPEEIEGMFLDTSNLSFDIKHVAFDNMLNLRLFKIYSSNPEVHHVNNFLKGSLSSLPNVLRLLHWENYPLQFLPQNFDPIHLVEINMPYSQLKKLWGGTKDLEMLKTIRLCHSQQLVDIDDLLKAQNLEVVDLQGCTRLQSFPATGQLLHLRVVNLSGCTEIKSFPEIPPNIETLNLQGTGIIELPLSIVKPNYRELLNLLAEIPGLSGVSNLEQSDLKPLTSLMKISTSYQNPGKLSCLELNDCSRLRSLPNMVNLELLKALDLSGCSELETIQGFPRNLKELYLVGTAVRQVPQLPQSLEFFNAHGCVSLKSIRLDFKKLPVHYTFSNCFDLSPQVVNDFLVQAMANVIAKHIPRERHVTGFSQKTVQRSSRDSQQELNKTLAFSFCAPSHANQNSKLDLQPGSSSMTRLDPSWRNTLVGFAMLVQVAFSEGYCDDTDFGISCVCKWKNKEGHSHRREINLHCWALGKAVERDHTFVFFDVNMRPDTDEGNDPDIWADLVVFEFFPVNKQRKPLNDSCTVTRCGVRLITAVNCNTSIENISPVLSLDPMEVSGNEDEEVLRVRYAGLQEIYKALFLYIAGLFNDEDVGLVAPLIANIIDMDVSYGLKVLAYRSLIRVSSNGEIVMHYLLRQMGKEILHTESKKTDKLVDNIQSSMIATKEIEITRSKSRRKNNKEKRVVCVVDRGSRSSDLWVWRKYGQKPIKSSPYPRSYYRCASSKGCFARKQVERSRTDPNVSVITYISEHNHPFPTLRNTLAGSTRSSSSKCSDVTTSASSTVSQDKEGPDKSHLPSSPASPPYAAMVVKEEDMEQWDNMEFDVDVEEDTFIPELFPEDTFADMDKLEENSQTMFLSRRSSGGNMEAQGKNSSDDREVNLPSKILNR

>AtWRKY17

MTVDIMRLPKMEDQTAIQEAASQGLKSMEHLIRVLSNRPEERNVDCSEITDFTVSKFKKVISLLNRSGHARFRRGPVHSPPSSSVPPPVKVTTPAPTQISAPAPVSFVQANQQSVTLDFTRPSVFGAKTKSSEVVEFAKESFSVSSNSSFMSSAITGDGSVSKGSSIFLAPAPAVPVTSSGKPPLSGLPYRKRCFEHDHSEGFSGKISGSGNGKCHCKKSRKNRMKRTVRVPAVSAKIADIPPDEYSWRKYGQKPIKGSPHPRGYYKCSTFRGCPARKHVERALDDSTMLIVTYEGEHRHHQSTMQEHVTPSVSGLVFGSA

>AtWRKY18

MDGSSFLDISLDLNTNPFSAKLPKKEVSVLASTHLKRKWLEQDESASELREELNRVNSENKKLTEMLARVCESYNELHNHLEKLQSRQSPEIEQTDIPIKKRKQDPDEFLGFPIGLSSGKTENSSSNEDHHHHHQQHEQKNQLLSCKRPVTDSFNKAKVSTVYVPTETSDTSLTVKDGFQWRKYGQKVTRDNPSPRAYFRCSFAPSCPVKKKVQRSAEDPSLLVATYEGTHNHLGPNASEGDATSQGGSSTVTLDLVNGCHRLALEKNERDNTMQEVLIQQMASSLTKDSKFTAALAAAISGRLMEQSRT

>AtWRKY19

MSEKEELPLTLTSIGAATATSDYHQRVGSSGEGISSSSSDVDPRFMQNSPTGLMISQSSSMCTVPPGMAATPPISSGSGLSQQLNNSSSSKLCQVEGCQKGARDASGRCISHGGGRRCQKPDCQKGAEGKTVYCKAHGGGRRCEYLGCTKGAEGSTDFCIAHGGGRRCNHEDCTRSAWGRTEFCVKHGGGARCKTYGCGKSASGPLPFCRAHGGGKKCSHEDCTGFARGRSGLCLMHGGGKRCQRENCTKSAEGLSGLCISHGGGRRCQSIGCTKGAKGSKMFCKACITKRPLTIDGGGNMGGVTTGDALNYLKAVKDKFEDSEKYDTFLEVLNDCKHQGVDTSGVIARLKDLFKGHDDLLLGFNTYLSKEYQITILPEDDFPIDFLDKVEGPYEMTYQQAQTVQANANMQPQTEYPSSSAVQSFSSGQPQIPTSAPDSSLLAKSNTSGITIIEHMSQQPLNVDKQVNDGYNWQKYGQKKVKGSKFPLSYYKCTYLGCPSKRKVERSLDGQVAEIVYKDRHNHEPPNQGKDGSTTYLSGSSTHINCMSSELTASQFSSNKTKIEQQEAASLATTIEYMSEASDNEEDSNGETSEGEKDEDEPEPKRRITEVQVSELADASDRTVREPRVIFQTTSEVDNLDDGYRWRKYGQKVVKGNPYPRFSSSKDYDVVIRYGRADISNEDFISHLRASLCRRGISVYEKFNEVDALPKCRVLIIVLTSTYVPSNLLNILEHQHTEDRVVYPIFYRLSPYDFVCNSKNYERFYLQDEPKKWQAALKEITQMPGYTLTDKSESELIDEIVRDALKVLCSADKVNMIGMDMQVEEILSLLCIESLDVRSIGIWGTVGIGKTTIAEEIFRKISVQYETCVVLKDLHKEVEVKGHDAVRENFLSEVLEVEPHVIRISDIKTSFLRSRLQRKRILVILDDVNDYRDVDTFLGTLNYFGPGSRIIMTSRNRRVFVLCKIDHVYEVKPLDIPKSLLLLDRGTCQIVLSPEVYKTLSLELVKFSNGNPQVLQFLSSIDREWNKLSQEVKTTSPIYIPGIFEKSCCGLDDNERGIFLDIACFFNRIDKDNVAMLLDGCGFSAHVGFRGLVDKSLLTISQHNLVDMLSFIQATGREIVRQESADRPGDRSRLWNADYIRHVFINDTGTSAIEGIFLDMLNLKFDANPNVFEKMCNLRLLKLYCSKAEEKHGVSFPQGLEYLPSKLRLLHWEYYPLSSLPKSFNPENLVELNLPSSCAKKLWKGKKARFCTTNSSLEKLKKMRLSYSDQLTKIPRLSSATNLEHIDLEGCNSLLSLSQSISYLKKLVFLNLKGCSKLENIPSMVDLESLEVLNLSGCSKLGNFPEISPNVKELYMGGTMIQEIPSSIKNLVLLEKLDLENSRHLKNLPTSIYKLKHLETLNLSGCISLERFPDSSRRMKCLRFLDLSRTDIKELPSSISYLTALDELLFVDSRRNSPVVTNPNANSTELMPSESSKLEILGTPADNEVVVGGTVEKTRGIERTPTILVKSREYLIPDDVVAVGGDIKGLRPPVLQLQPAMKLSHIPRGSTWDFVTHFAPPETVAPPSSSSEAREEEVETEETGAMFIPLGDKETCSFTVNKGDSSRTISNTSPIYASEGSFITCWQKGQLLGRGSLGSVYEGISADGDFFAFKEVSLLDQGSQAHEWIQQVEGGIALLSQLQHQNIVRYRGTTKDESNLYIFLELVTQGSLRKLYQRNQLGDSVVSLYTRQILDGLKYLHDKGFIHRNIKCANVLVDANGTVKLADFGLAKVMSLWRTPYWNWMAPEVIVLKSFPLF

>AtWRKY20

MILLPEPSPTTGSLFKPRPVHISASSSSYTGRGFHQNTFTEQKSSEFEFRPPASNMVYAELGKIRSEPPVHFQGQGHGSSHSPSSISDAAGSSSELSRPTPPCQMTPTSSDIPAGSDQEESIQTSQNDSRGSTPSILADDGYNWRKYGQKHVKGSEFPRSYYKCTHPNCEVKKLFERSHDGQITDIIYKGTHDHPKPQPGRRNSGGMAAQEERLDKYPSSTGRDEKGSGVYNLSNPNEQTGNPEVPPISASDDGGEAAASNRNKDEPDDDDPFSKRRRMEGAMEITPLVKPIREPRVVVQTLSEVDILDDGYRWRKYGQKVVRGNPNPRSYYKCTAHGCPVRKHVERASHDPKAVITTYEGKHDHDVPTSKSSSNHEIQPRFRPDETDTISLNLGVGISSDGPNHASNEHQHQNQQLVNQTHPNGVNFRFVHASPMSSYYASLNSGMNQYGQRETKNETQNGDISSLNNSSYPYPPNMGRVQSGP

>AtWRKY21

MEEIEGTNRAAVESCHRVLNLLHRSQQQDHVGFEKNLVSETREAVIRFKRVGSLLSSSVGHARFRRAKKLQSHVSQSLLLDPCQQRTTEVPSSSSQKTPVLRSGFQELSLRQPSDSLTLGTRSFSLNSNAKAPLLQLNQQTMPPSNYPTLFPVQQQQQQQQQQQQQEQQQQQQQQQQQFHERLQAHHLHQQQQLQKHQAELMLRKCNGGISLSFDNSSCTPTMSSTRSFVSSLSIDGSVANIEGKNSFHFGVPSSTDQNSLHSKRKCPLKGDEHGSLKCGSSSRCHCAKKRKHRVRRSIRVPAISNKVADIPPDDYSWRKYGQKPIKGSPYPRGYYKCSSMRGCPARKHVERCLEDPAMLIVTYEAEHNHPKLPSQAITT

>AtWRKY22

MADDWDLHAVVRGCSAVSSSATTTVYSPGVSSHTNPIFTVGRQSNAVSFGEIRDLYTPFTQESVVSSFSCINYPEEPRKPQNQKRPLSLSASSGSVTSKPSGSNTSRSKRRKIQHKKVCHVAAEALNSDVWAWRKYGQKPIKGSPYPRGYYRCSTSKGCLARKQVERNRSDPKMFIVTYTAEHNHPAPTHRNSLAGSTRQKPSDQQTSKSPTTTIATYSSSPVTSADEFVLPVEDHLAVGDLDGEEDLLSLSDTVVSDDFFDGLEEFAAGDSFSGNSAPASFDLSWVVNSAATTTGGI

>AtWRKY23

MEFTDFSKTSFYYPSSQSVWDFGDLAAAERHSLGFMELLSSQQHQDFATVSPHSFLLQTSQPQTQTQPSAKLSSSIIQAPPSEQLVTSKVESLCSDHLLINPPATPNSSSISSASSEALNEEKPKTEDNEEEGGEDQQEKSHTKKQLKAKKNNQKRQREARVAFMTKSEVDHLEDGYRWRKYGQKAVKNSPFPRSYYRCTTASCNVKKRVERSFRDPSTVVTTYEGQHTHISPLTSRPISTGGFFGSSGAASSLGNGCFGFPIDGSTLISPQFQQLVQYHHQQQQQELMSCFGGVNEYLNSHANEYGDDNRVKKSRVLVKDNGLLQDVVPSHMLKEE

>AtWRKY24

MDREDINPMLSRLDVENNNTFSSFVDKTLMMMPPSTFSGEVEPSSSSSWYPESFHVHAPPLPPENDQIGEKGKELKEKRSRKVPRIAFHTRSDDDVLDDGYRWRKYGQKSVKHNAHPRSYYRCTYHTCNVKKQVQRLAKDPNVVVTTYEGVHNHPCEKLMETLNPLLRQLQFLSSFSNL

>AtWRKY25

MSSTSFTDLLGSSGVDCYEDDEDLRVSGSSFGGYYPERTGSGLPKFKTAQPPPLPISQSSHNFTFSDYLDSPLLLSSSHSLISPTTGTFPLQGFNGTTNNHSDFPWQLQSQPSNASSALQETYGVQDHEKKQEMIPNEIATQNNNQSFGTERQIKIPAYMVSRNSNDGYGWRKYGQKQVKKSENPRSYFKCTYPDCVSKKIVETASDGQITEIIYKGGHNHPKPEFTKRPSQSSLPSSVNGRRLFNPASVVSEPHDQSENSSISFDYSDLEQKSFKSEYGEIDEEEEQPEMKRMKREGEDEGMSIEVSKGVKEPRVVVQTISDIDVLIDGFRWRKYGQKVVKGNTNPRSYYKCTFQGCGVKKQVERSAADERAVLTTYEGRHNHDIPTALRRS

>AtWRKY26

MGSFDRQRAVPKFKTATPSPLPLSPSPYFTMPPGLTPADFLDSPLLFTSSNILPSPTTGTFPAQSLNYNNNGLLIDKNEIKYEDTTPPLFLPSMVTQPLPQLDLFKSEIMSSNKTSDDGYNWRKYGQKQVKGSENPRSYFKCTYPNCLTKKKVETSLVKGQMIEIVYKGSHNHPKPQSTKRSSSTAIAAHQNSSNGDGKDIGEDETEAKRWKREENVKEPRVVVQTTSDIDILDDGYRWRKYGQKVVKGNPNPRSYYKCTFTGCFVRKHVERAFQDPKSVITTYEGKHKHQIPTPRRGPVLRLLGKTET

>AtWRKY27

MSSEDWDLFAVVRSCSSSVSTTNSCAGHEDDIGNCKQQQDPPPPPLFQASSSCNELQDSCKPFLPVTTTTTTTWSPPPLLPPPKASSPSPNILLKQEQVLLESQDQKPPLSVRVFPPSTSSSVFVFRGQRDQLLQQQSQPPLRSRKRKNQQKRTICHVTQENLSSDLWAWRKYGQKPIKGSPYPRNYYRCSSSKGCLARKQVERSNLDPNIFIVTYTGEHTHPRPTHRNSLAGSTRNKSQPVNPVPKPDTSPLSDTVKEEIHLSPTTPLKGNDDVQETNGDEDMVGQEVNMEEEEEEEEVEEDDEEEEDDDDVDDLLIPNLAVRDRDDLFFAGSFPSWSAGSAGDGGG

>AtWRKY28

MSNETRDLYNYQYPSSFSLHEMMNLPTSNPSSYGNLPSQNGFNPSTYSFTDCLQSSPAAYESLLQKTFGLSPSSSEVFNSSIDQEPNRDVTNDVINGGACNETETRVSPSNSSSSEADHPGEDSGKSRRKRELVGEEDQISKKVGKTKKTEVKKQREPRVSFMTKSEVDHLEDGYRWRKYGQKAVKNSPYPRSYYRCTTQKCNVKKRVERSFQDPTVVITTYEGQHNHPIPTNLRGSSAAAAMFSADLMTPRSFAHDMFRTAAYTNGGSVAAALDYGYGQSGYGSVNSNPSSHQVYHQGGEYELLREIFPSIFFKQEP

>AtWRKY29

MGEVAYMDEGDLEAIVRGYSGSGDAFSGESSGTFSPSFCLPMETSSFYEPEMETSGLDELGELYKPFYPFSTQTILTSSVSLPEDSKPFRDDKKQRSHGCLLSNGSRADHIRISESKSKKSKKNQQKRVVEQVKEENLLSDAWAWRKYGQKPIKGSPYPRSYYRCSSSKGCLARKQVERNPQNPEKFTITYTNEHNHELPTRRNSLAGSTRAKTSQPKPTLTKKSEKEVVSSPTSNPMIPSADESSVAVQEMSVAETSTHQAAGAIEGRRLSNGLPSDLMSGSGTFPSFTGDFDELLNSQEFFSGYLWNY

>AtWRKY30

MEKNHSSGEWEKMKNEINELMIEGRDYAHQFGSASSQETREHLAKKILQSYHKSLTIMNYSGELDQVSQGGGSPKSDDSDQEPLVIKSSKKSMPRWSSKVRIAPGAGVDRTLDDGFSWRKYGQKDILGAKFPRGYYRCTYRKSQGCEATKQVQRSDENQMLLEISYRGIHSCSQAANVGTTMPIQNLEPNQTQEHGNLDMVKESVDNYNHQAHLHHNLHYPLSSTPNLENNNAYMLQMRDQNIEYFGSTSFSSDLGTSINYNFPASGSASHSASNSPSTVPLESPFESYDPNHPYGGFGGFYS

>AtWRKY31

MFRFPVSLGGSRDEDRHDQITPLDDHRVVVDEVDFFSEKRDRVSRENINDDDDEGNKVLIKMEGSRVEENDRSRDVNIGLNLLTANTGSDESTVDDGLSMDMEDKRAKIENAQLQEELKKMKIENQRLRDMLSQATTNFNALQMQLVAVMRQQEQRNSSQDHLLAQESKAEGRKRQELQIMVPRQFMDLGPSSGAAEHGAEVSSEERTTVRSGSPPSLLESSNPRENGKRLLGREESSEESESNAWGNPNKVPKHNPSSSNSNGNRNGNVIDQSAAEATMRKARVSVRARSEAAMISDGCQWRKYGQKMAKGNPCPRAYYRCTMAGGCPVRKQVQRCAEDRSILITTYEGNHNHPLPPAATAMASTTTAAASMLLSGSMSSQDGLMNPTNLLARAILPCSSSMATISASAPFPTITLDLTNSPNGNNPNMTTNNPLMQFAQRPGFNPAVLPQVVGQAMYNNQQQSKFSGLQLPAQPLQIAATSSVAESVSAASAAIASDPNFAAALAAAITSIMNGSSHQNNNTNNNNVATSNNDSRQ

>AtWRKY32

MEEDTGIDEAKTYTVEKSEKVEPEKDGLSQFRDEEKSLGADMEDLHDETVRETLGKDQVQGVRENSSVEPNVEDVLEVNETDSVKETVVSAIVPVDEVEENRQVETSPSLAASSDSLTVTPCLSLDPATASTAQDLPLVSVPTKQEQRSDSPVVNRLSVTPVPRTPARDGYNWRKYGQKQVKSPKGSRSYYRCTYTECCAKKIECSNDSGNVVEIVNKGLHTHEPPRKTSFSPREIRVTTAIRPVSEDDTVVEELSIVPSGSDPSASTKEYICESQTLVDRKRHCENEAVEEPEPKRRLKKDNSQSSDSVSKPGKKNKFVVHAAGDVGICGDGYRWRKYGQKMVKGNPHPRNYYRCTSAGCPVRKHIETAVENTKAVIITYKGVHNHDMPVPKKRHGPPSSMLVAAAAPTSMRTRTDDQVNIPTSSQCSVGRESEKQSKEALDVGGEKVMESARTLLSIGFEIKQC

>AtWRKY33

MAASFLTMDNSRTRQNMNGSANWSQQSGRTSTSSLEDLEIPKFRSFAPSSISISPSLVSPSTCFSPSLFLDSPAFVSSSANVLASPTTGALITNVTNQKGINEGDKSNNNNFNLFDFSFHTQSSGVSAPTTTTTTTTTTTTTNSSIFQSQEQQKKNQSEQWSQTETRPNNQAVSYNGREQRKGEDGYNWRKYGQKQVKGSENPRSYYKCTFPNCPTKKKVERSLEGQITEIVYKGSHNHPKPQSTRRSSSSSSTFHSAVYNASLDHNRQASSDQPNSNNSFHQSDSFGMQQEDNTTSDSVGDDEFEQGSSIVSRDEEDCGSEPEAKRWKGDNETNGGNGGGSKTVREPRIVVQTTSDIDILDDGYRWRKYGQKVVKGNPNPRSYYKCTTIGCPVRKHVERASHDMRAVITTYEGKHNHDVPAARGSGYATNRAPQDSSSVPIRPAAIAGHSNYTTSSQAPYTLQMLHNNNTNTGPFGYAMNNNNNNSNLQTQQNFVGGGFSRAKEEPNEETSFFDSFMP

>AtWRKY34

MAGIDNKAAVMGEWFDCSTTNHRKRSKAELGREFSLNYIKNEDSLQTTFQESSRGALRERIAARSGFNAPWLNTEDILQSKSLTISSPGLSPATLLESPVFLSNPLLSPTTGKLSSVPSDKAKAELFDDITTSLAFQTISGSGLDPTNIALEPDDSQDYEERQLGGLGDSMACCAPADDGYNWRKYGQKLVKGSEYPRSYYKCTHPNCEAKKKVERSREGHIIEIIYTGDHIHSKPPPNRRSGIGSSGTGQDMQIDATEYEGFAGTNENIEWTSPVSAELEYGSHSGSMQVQNGTHQFGYGDAAADALYRDENEDDRTSHMSVSLTYDGEVEESESKRRKLEAYATETSGSTRASREPRVVVQTTSDIDILDDGYRWRKYGQKVVKGNPNPRSYYKCTANGCTVTKHVERASDDFKSVLTTYIGKHTHVVPAARNSSHVGAGSSGTLQGSLATQTHNHNVHYPMPHSRSEGLATANSSLFDFQSHLRHPTGFSVYIGQSELSDLSMPGLTIGQEKLTSLQAPDIGDPTGLMLQLAAQPKVEPVSPQQGLDLSASSLICREMLSRLRQI

>AtWRKY35

MDNFQGDLTDVVRGIGSGHVSPSPGPPEGPSPSSMSPPPTSDLHVEFPSAATSASCLANPFGDPFVSMKDPLIHLPASYISGAGDNKSNKSFAIFPKIFEDDHIKSQCSVFPRIKISQSNNIHDASTCNSPAITVSSAAVAASPWGMINVNTTNSPRNCLLVDNNNNTSSCSQVQISSSPRNLGIKRRKSQAKKVVCIPAPAAMNSRSSGEVVPSDLWAWRKYGQKPIKGSPYPRGYYRCSSSKGCSARKQVERSRTDPNMLVITYTSEHNHPWPTQRNALAGSTRSSSSSSLNPSSKSSTAAATTSPSSRVFQNNSSKDEPNNSNLPSSSTHPPFDAAAIKEENVEERQEKMEFDYNDVENTYRPELLQEFQHQPEDFFADLDELEGDSLTMLLSHSSGGGNMENKTTIPDVFSDFFDDDESSRSL

>AtWRKY36

MIKEETVSYFQTFDGVMAESDKEEELDATKAKVEKVREENEKLKLLLSTILNNYNSLQMQVSKVLGQQQGASSMELDHIDRQDENNDYDVDISLRLGRSEQKISKKEENKVDKISTKNVEESKDKRSALGFGFQIQSYEASKLDDLCRQVKLANAENKCVSSRKDVKSVRNENHQDVLEEHEQTGLKKTRVCVKASCEDPSINDGCQWRKYGQKTAKTNPLPRAYYRCSMSSNCPVRKQVQRCGEEETSAFMTTYEGNHDHPLPMEASHMAAGTSAAASLLQSGSSSSSSSTSASLSYFFPFHHFSISTTNSHPTVTLDLTRPNYPNQLPDDYPLSSSSFSLNFSSPDPPPPSSHDHTLNFSGLRTQAPLSTDSLLARYRTRLSGQQ

>AtWRKY38

MEMNSPHEKAVQAIRYGHSCAMRLKRRLNHPMADGGPLSSYDLAKSIVESFSNAISILSAKPETEDDQFSDLSSRDSSPPPQGSPSKKRKIDSTNSSENWRDDSPDPIYYDGYLWRKYGQKSIKKSNHQRSYYRCSYNKDHNCEARKHEQKIKDNPPVYRTTYFGHHTCKTEHNLDAIFIAGQDPLDDFKSTQMIRFGKDQDQEKESRSNGFSLSVKHEEDIIKEQAIDQYREITSNDQDCQDVIEEYLSSPSGSYPPSSSSGSESADFNSDLLFDNPDSWDRYDQFYF

>AtWRKY39

MEEVEAANRSAIESCHGVLNLLSQRTSDPKSLTVETGEVVSKFKRVASLLTRGLGHGKFRSTNKFRSSFPQHIFLESPICCGNDLSGDYTQVLAPEPLQMVPASAVYNEMEPKHQLGHPSLMLSHKMCVDKSFLELKPPPFRAPYQLIHNHQQIAYSRSNSGVNLKFDGSGSSCYTPSVSNGSRSFVSSLSMDASVTDYDRNSFHLTGLSRGSDQQHTRKMCSGSLKCGSRSKCHCSKKRKLRVKRSIKVPAISNKIADIPPDEYSWRKYGQKPIKGSPHPRGYYKCSSVRGCPARKHVERCIDETSMLIVTYEGEHNHSRILSSQSAHT

>AtWRKY40

MDQYSSSLVDTSLDLTIGVTRMRVEEDPPTSALVEELNRVSAENKKLSEMLTLMCDNYNVLRKQLMEYVNKSNITERDQISPPKKRKSPAREDAFSCAVIGGVSESSSTDQDEYLCKKQREETVVKEKVSRVYYKTEASDTTLVVKDGYQWRKYGQKVTRDNPSPRAYFKCACAPSCSVKKKVQRSVEDQSVLVATYEGEHNHPMPSQIDSNNGLNRHISHGGSASTPVAANRRSSLTVPVTTVDMIESKKVTSPTSRIDFPQVQKLLVEQMASSLTKDPNFTAALAAAVTGKLYQQNHTEK

>AtWRKY41

MEMMNWERRSLLNELIHGLKAAKQLQGSSSPSLSASSSYLTTEIKENLLHNIVSSFKKAILMLNGSTTQHNPTIELAPDPLAHPGKVPGSPASITGNPRSEEFFNVRSKEFNLSSKKRKMLPKWTEQVRISPERGLEGPHDDIFSWRKYGQKDILGAKFPRSYYRCTFRNTQYCWATKQVQRSDGDPTIFEVTYRGTHTCSQGIPLPEKRETKPKHTVAVNYQNLRASLTVRTGGLGSEAFSFPVTSPLYTYESINGGGTFYHHVGSSGPSDFTGLISTNTSTGSSPIFDVNFQFDPTAEINTGFPTFFHNSI

>AtWRKY42

MFRFPVSLGGGPRENLKPSDEQHQRAVVNEVDFFRSAEKRDRVSREEQNIIADETHRVHVKRENSRVDDHDDRSTDHINIGLNLLTANTGSDESMVDDGLSVDMEEKRTKCENAQLREELKKASEDNQRLKQMLSQTTNNFNSLQMQLVAVMRQQEDHHHLATTENNDNVKNRHEVPEMVPRQFIDLGPHSDEVSSEERTTVRSGSPPSLLEKSSSRQNGKRVLVREESPETESNGWRNPNKVPKHHASSSICGGNGSENASSKVIEQAAAEATMRKARVSVRARSEAPMLSDGCQWRKYGQKMAKGNPCPRAYYRCTMAVGCPVRKQVQRCAEDRTILITTYEGNHNHPLPPAAMNMASTTTAAASMLLSGSTMSNQDGLMNPTNLLARTILPCSSSMATISASAPFPTITLDLTESPNGNNPTNNPLMQFSQRSGLVELNQSVLPHMMGQALYYNQQSKFSGLHMPSQPLNAGESVSAATAAIASNPNFAAALAAAITSIINGSNNQQNGNNNNSNVTTSNVDNRQ

>AtWRKY43

MNGLVDSSRDKKMKNPRFSFRTKSDADILDDGYRWRKYGQKSVKNSLYPRSYYRCTQHMCNVKKQVQRLSKETSIVETTYEGIHNHPCEELMQTLTPLLHQLQFLSKFT

>AtWRKY44

MEVNDGERVVIAKPVASRPSSSSGFRTFTELLTDSVTVSPQTTCHEIVDAAIRPKTLRFNQPVAASVSCPRAEVKGIGNGMSCDDDSDSRNYVVYKPKAKLVSKATVSALANMLQGNRQQTWRQSEAVSYGKSVSQGTHRAGPNLVQKVPSFTESETSTGDRSSVDGYNWRKYGQKQVKGSECPRSYYKCTHPKCPVKKKVERSVEGQVSEIVYQGEHNHSKPSCPLPRRASSSISSGFQKPPKSIASEGSMGQDPNNNLYSPLWNNQSNDSTQNRTEKMSEGCVITPFEFAVPRSTNSNPGTSDSGCKSSQCDEGELDDPSRSKRRKNEKQSSEAGVSQGSVESDSLEDGFRWRKYGQKVVGGNAYPRSYYRCTSANCRARKHVERASDDPRAFITTYEGKHNHHLLLSPPSSSTLPFNSPQLSKQTI

>AtWRKY45

MEDRRCDVLFPCSSSVDPRLTEFHGVDNSAQPTTSSEEKPRSKKKKKEREARYAFQTRSQVDILDDGYRWRKYGQKAVKNNPFPRSYYKCTEEGCRVKKQVQRQWGDEGVVVTTYQGVHTHAVDKPSDNFHHILTQMHIFPPFCLKE

>AtWRKY46

MMMEEKLVINELELGKELANRLMNNLKHTSSVDSNKTLISDILRIYQNAIFMLSFNQDKNILKRSLEIDGKDSKNVFKKRKVSEKNTEKVKVFVATEQENGSIDDGHCWRKYGQKEIHGSKNPRAYYRCTHRFTQDCLAVKQVQKSDTDPSLFEVKYLGNHTCNNITSPKTTTNFSVSLTNTNIFEGNRVHVTEQSEDMKPTKSEEVMISLEDLENKKNIFRTFSFSNHEIENGVWKSNLFLGNFVEDLSPATSGSAITSEVLSAPAAVENSETADSYFSSLDNIIDFGQDWLWS

>AtWRKY47

MEEHIQDRREIAFLHSGEFLHGDSDSKDHQPNESPVERHHESSIKEVDFFAAKSQPFDLGHVRTTTIVGSSGFNDGLGLVNSCHGTSSNDGDDKTKTQISRLKLELERLHEENHKLKHLLDEVSESYNDLQRRVLLARQTQVEGLHHKQHEDVPQAGSSQALENRRPKDMNHETPATTLKRRSPDDVDGRDMHRGSPKTPRIDQNKSTNHEEQQNPHDQLPYRKARVSVRARSDATTVNDGCQWRKYGQKMAKGNPCPRAYYRCTMAVGCPVRKQVQRCAEDTTILTTTYEGNHNHPLPPSATAMAATTSAAAAMLLSGSSSSNLHQTLSSPSATSSSSFYHNFPYTSTIATLSASAPFPTITLDLTNPPRPLQPPPQFLSQYGPAAFLPNANQIRSMNNNNQQLLIPNLFGPQAPPREMVDSVRAAIAMDPNFTAALAAAISNIIGGGNNDNNNNTDINDNKVDAKSGGSSNGDSPQLPQSCTTFSTN

>AtWRKY48

MEKKKEEDHHHQQQQQQQKEIKNTETKIEQEQEQEQKQEISQASSSSNMANLVTSSDHHPLELAGNLSSIFDTSSLPFPYSYFEDHSSNNPNSFLDLLRQDHQFASSSNSSSFSFDAFPLPNNNNNTSFFTDLPLPQAESSEVVNTTPTSPNSTSVSSSSNEAANDNNSGKEVTVKDQEEGDQQQEQKGTKPQLKAKKKNQKKAREARFAFLTKSDIDNLDDGYRWRKYGQKAVKNSPYPRSYYRCTTVGCGVKKRVERSSDDPSIVMTTYEGQHTHPFPMTPRGHIGMLTSPILDHGATTASSSSFSIPQPRYLLTQHHQPYNMYNNNSLSMINRRSSDGTFVNPGPSSSFPGFGYDMSQASTSTSSSIRDHGLLQDILPSQIRSDTINTQTNEENKK

>AtWRKY49

MEEEGYQWARRCGNNAVEDPFVYEPPLFFLPQDQHHMHGLMPNEDFIANKFVTSTLYSGPRIQDIANALALVEPLTHPVREISKSTVPLLERSTLSKVDRYTLKVKNNSNGMCDDGYKWRKYGQKSIKNSPNPRSYYKCTNPICNAKKQVERSIDESNTYIITYEGFHFHYTYPFFLPDKTRQWPNKKTKIHKHNAQDMNKKSQTQEESKEAQLGELTNQNHPVNKAQENTPANLEEGLFFPVDQCRPQQGLLEDVVAPAMKNIPTRDSVLTAS

>AtWRKY50

MNDADTNLGSSFSDDTHSVFEFPELDLSDEWMDDDLVSAVSGMNQSYGYQTSDVAGALFSGSSSCFSHPESPSTKTYVAATATASADNQNKKEKKKIKGRVAFKTRSEVEVLDDGFKWRKYGKKMVKNSPHPRNYYKCSVDGCPVKKRVERDRDDPSFVITTYEGSHNHSSMN

>AtWRKY51

MNISQNPSPNFTYFSDENFINPFMDNNDFSNLMFFDIDEGGNNGLIEEEISSPTSIVSSETFTGESGGSGSATTLSKKESTNRGSKESDQTKETGHRVAFRTRSKIDVMDDGFKWRKYGKKSVKNNINKRNYYKCSSEGCSVKKRVERDGDDAAYVITTYEGVHNHESLSNVYYNEMVLSYDHDNWNQHSLLRS

>AtWRKY52

MTNCEKDEEFVCISCVEEVRYSFVSHLSEALRRKGINNVVVDVDIDDLLFKESQAKIEKAGVSVMVLPGNCDPSEVWLDKFAKVLECQRNNKDQAVVSVLYGDSLLRDQWLSELDFRGLSRIHQSRKECSDSILVEEIVRDVYETHFYVGRIGIYSKLLEIENMVNKQPIGIRCVGIWGMPGIGKTTLAKAVFDQMSSAFDASCFIEDYDKSIHEKGLYCLLEEQLLPGNDATIMKLSSLRDRLNSKRVLVVLDDVRNALVGESFLEGFDWLGPGSLIIITSRDKQVFCLCGINQIYEVQGLNEKEARQLFLLSASIKEDMGEQNLQELSVRVINYANGNPLAISVYGRELKGKKKLSEMETAFLKLKRRPPFKIVDAFKSTYDTLSDNEKNIFLDIACFFQGENVNYVIQLLEGCGFFPHVEIDVLVDKCLVTISENRVWLHKLTQDIGREIINGETVQIERRRRLWEPWSIKYLLEYNEHKANGEPKTTFKRAQGSEEIEGLFLDTSNLRFDLQPSAFKNMLNLRLLKIYCSNPEVHPVINFPTGSLHSLPNELRLLHWENYPLKSLPQNFDPRHLVEINMPYSQLQKLWGGTKNLEMLRTIRLCHSHHLVDIDDLLKAENLEVIDLQGCTRLQNFPAAGRLLRLRVVNLSGCIKIKSVLEIPPNIEKLHLQGTGILALPVSTVKPNHRELVNFLTEIPGLSEELERLTSLLESNSSCQDLGKLICLELKDCSCLQSLPNMANLDLNVLDLSGCSSLNSIQGFPRFLKQLYLGGTAIREVPQLPQSLEILNAHGSCLRSLPNMANLEFLKVLDLSGCSELETIQGFPRNLKELYFAGTTLREVPQLPLSLEVLNAHGSDSEKLPMHYKFNNFFDLSQQVVNDFLLKTLTYVKHIPRGYTQELINKAPTFSFSAPSHTNQNATFDLQSGSSVMTRLNHSWRNTLVGFGMLVEVAFPEDYCDATDVGISCVCRWSNKEGRSCRIERKFHCWAPWQVVPKVRKDHTFVFSDVNMRPSTGEGNDPDIWAGLVVFEFFPINQQTKCLNDRFTVRRCGVRVINVATGNTSLENIALVLSLDPVEVSGYEVLRVSYDDLQEMDKVLFLYIASLFNDEDVDFVAPLIAGIDLDVSSGLKVLADVSLISVSSNGEIVMHSLQRQMGKEILHGQSMLLSDCESSMTENLSDVPKKKKKHSESRVKKVVSIPAIDEGDLWTWRKYGQKDILGSRFPRGYYRCAYKFTHGCKATKQVQRSETDSNMLAITYLSEHNHPRPTKRKALADSTRSTSSSIC

>AtWRKY53

MEGRDMLSWEQKTLLSELINGFDAAKKLQARLREAPSPSSSFSSPATAVAETNEILVKQIVSSYERSLLLLNWSSSPSVQLIPTPVTVVPVANPGSVPESPASINGSPRSEEFADGGGSSESHHRQDYIFNSKKRKMLPKWSEKVRISPERGLEGPQDDVFSWRKYGQKDILGAKFPRSYYRCTHRSTQNCWATKQVQRSDGDATVFEVTYRGTHTCSQAITRTPPLASPEKRQDTRVKPAITQKPKDILESLKSNLTVRTDGLDDGKDVFSFPDTPPFYNYGTINGEFGHVESSPIFDVVDWFNPTVEIDTTFPAFLHESIYY

>AtWRKY54

MDSNSNNTKSIKRKVVDQLVEGYEFATQLQLLLSHQHSNQYHIDETRLVSGSGSVSGGPDPVDELMSKILGSFHKTISVLDSFDPVAVSVPIAVEGSWNASCGDDSATPVSCNGGDSGESKKKRLGVGKGKRGCYTRKTRSHTRIVEAKSSEDRYAWRKYGQKEILNTTFPRSYFRCTHKPTQGCKATKQVQKQDQDSEMFQITYIGYHTCTANDQTHAKTEPFDQEIIMDSEKTLAASTAQNHVNAMVQEQENNTSSVTAIDAGMVKEEQNNNGDQSKDYYEGSSTGEDLSLVWQETMMFDDHQNHYYCGETSTTSHQFGFIDNDDQFSSFFDSYCADYERTSAM

>AtWRKY55

MYSYKKISYQMEEVMSMIFHGMKLVKSLESSLPEKPPESLLTSLDEIVKTFSDANERLKMLLEIKNSETALNKTKPVIVSVANQMLMQMEPGLMQEYWLRYGGSTSSQGTEAMFQTQLMAVDGGGERNLTAAVERSGASGSSTPRQRRRKDEGEEQTVLVAALRTGNTDLPPDDNHTWRKYGQKEILGSRFPRAYYRCTHQKLYNCPAKKQVQRLNDDPFTFRVTYRGSHTCYNSTAPTASSATPSTIPISSVTTGHSVDYGLAVVDMADVMFGSGGVGTNMDFIFPKNDPS

>AtWRKY56

MEGVDNTNPMLTLEEGENNNPFSSLDDKTLMMMAPSLIFSGDVGPSSSSCTPAGYHLSAQLENFRGGGGEMGGLVSNNSNNSDHNKNCNKGKGKRTLAMQRIAFHTRSDDDVLDDGYRWRKYGQKSVKNNAHPRSYYRCTYHTCNVKKQVQRLAKDPNVVVTTYEGVHNHPCEKLMETLSPLLRQLQFLSRVSDL

>AtWRKY57

MNDPDNPDLSNDDSAWRELTLTAQDSDFFDRDTSNILSDFGWNLHHSSDHPHSLRFDSDLTQTTGVKPTTVTSSCSSSAAVSVAVTSTNNNPSATSSSSEDPAENSTASAEKTPPPETPVKEKKKAQKRIRQPRFAFMTKSDVDNLEDGYRWRKYGQKAVKNSPFPRSYYRCTNSRCTVKKRVERSSDDPSIVITTYEGQHCHQTIGFPRGGILTAHDPHSFTSHHHLPPPLPNPYYYQELLHQLHRDNNAPSPRLPRPTTEDTPAVSTPSEEGLLGDIVPQTMRNP

>AtWRKY58

MAVEDDVSLIRTTTLVAPTRPTITVPHRPPAIETAAYFFGGGDGLSLSPGPLSFVSSLFVDNFPDVLTPDNQRTTSFTHLLTSPMFFPPQSSAHTGFIQPRQQSQPQPQRPDTFPHHMPPSTSVAVHGRQSLDVSQVDQRARNHYNNPGNNNNNRSYNVVNVDKPADDGYNWRKYGQKPIKGCEYPRSYYKCTHVNCPVKKKVERSSDGQITQIIYKGQHDHERPQNRRGGGGRDSTEVGGAGQMMESSDDSGYRKDHDDDDDDDEDDEDLPASKIRRIDGVSTTHRTVTEPKIIVQTKSEVDLLDDGYRWRKYGQKVVKGNPHPRSYYKCTTPNCTVRKHVERASTDAKAVITTYEGKHNHDVPAARNGTAAATAAAVGPSDHHRMRSMSGNNMQQHMSFGNNNNTGQSPVLLRLKEEKITI

>AtWRKY59

MNYPSNPNPSSTDFTEFFKFDDFDDTFEKIMEEIGREDHSSSPTLSWSSSEKLVAAEITSPLQTSLATSPMSFEIGDKDEIKKRKRHKEDPIIHVFKTKSSIDEKVALDDGYKWRKYGKKPITGSPFPRHYHKCSSPDCNVKKKIERDTNNPDYILTTYEGRHNHPSPSVVYCDSDDFDLNSLNNWSFQTANTYSFSHSAPY

>AtWRKY60

MDYDPNTNPFDLHFSGKLPKREVSASASKVVEKKWLVKDEKRNMLQDEINRVNSENKKLTEMLARVCEKYYALNNLMEELQSRKSPESVNFQNKQLTGKRKQELDEFVSSPIGLSLGPIENITNDKATVSTAYFAAEKSDTSLTVKDGYQWRKYGQKITRDNPSPRAYFRCSFSPSCLVKKKVQRSAEDPSFLVATYEGTHNHTGPHASVSRTVKLDLVQGGLEPVEEKKERGTIQEVLVQQMASSLTKDPKFTAALATAISGRLIEHSRT

>AtWRKY61

MEKDDFLRSGHGREESHDEMRKLDSSHDDSHQEHDHIIRSKLDSTKVEMDEAKEENRRLKSSLSKIKKDFDILQTQYNQLMAKHNEPTKFQSKGHHQDKGEDEDREKVNEREELVSLSLGRRLNSEVPSGSNKEEKNKDVEEAEGDRNYDDNEKSSIQGLSMGIEYKALSNPNEKLEIDHNQETMSLEISNNNKIRSQNSFGFKNDGDDHEDEDEILPQNLVKKTRVSVRSRCETPTMNDGCQWRKYGQKIAKGNPCPRAYYRCTIAASCPVRKQVQRCSEDMSILISTYEGTHNHPLPMSATAMASATSAAASMLLSGASSSSSAAADLHGLNFSLSGNNITPKPKTHFLQSPSSSGHPTVTLDLTTSSSSQQPFLSMLNRFSSPPSNVSRSNSYPSTNLNFSNNTNTLMNWGGGGNPSDQYRAAYGNINTHQQSPYHKIIQTRTAGSSFDPFGRSSSSHSPQINLDHIGIKNIISHQVPSLPAETIKAITTDPSFQSALATALSSIMGGDLKIDHNVTRNEAEKSP

>AtWRKY62

MNSCQQKAMEKLLHGHGCANQLLIMDQTESDSSMEREDLAKSVLHCFSDALSILIDTNDHQDDQSNNSSPQDSSPVLESSRKPLHKRGRKTSMAESSDYHRHESSTPIYHDGFLWRKYGQKQIKESEYQRSYYKCAYTKDQNCEAKKQVQKIQHNPPLYSTTYFGQHICQLHQAYATFPIDTSDFEEHEGSHMIRFGHPNISFSSSTSNLRQHQNHQDRIKDEYMKPVIAEDWSPSQWMSSEVALAVEAFEFNPFWTSHDLSS

>AtWRKY63

MFSNIDHKAVAALLHGQGCANILKTVLDNCKVSSVSTEPLINTILDSFSLALSSVNSPNRQPHHESSSRDMAGLVPQRSSKKKICGVKGLEIYRDDSPNPRLDDGFTWRKYGQKTIKTSLYQRCYYRCAYAKDQNCYATKRVQMIQDSPPVYRTTYLGQHTCKAFGVHDNTYGSEMINFDQVVSESVMRQLATIGEQAVLMEDEANHIMNQEYDINDYLVDDEVFWGNEFPLFSSEDLMLF

>AtWRKY64

MFSNIDQTAVAALLRGQGCANSLKRLLENHKLSSDSTEPLIYTILNSFSLALSFVDPPSLLPHNESSLQNMTSHVLQRSSKKKYYGAEDLEYYRDESPTPRPDDGFTWRKYGQKTIKTSPYQRCYYRCTYAKDQNCNARKRVQMIQDNPPVYRTTYLGKHVCKAVAVHDDTYGSEMIKFDQVVSESVMPQLATIDEQAITMEDEAIDHIMNQECDINDFSVDDDPFWASQFPPFSSEDIMFFDNIANLD

>AtWRKY65

MKRGLDMARSYNDHESSQETGPESPNSSTFNGMKALISSHSPKRSRRSVEKRVVNVPMKEMEGSRHKGDTTPPSDSWAWRKYGQKPIKGSPYPRGYYRCSSTKGCPARKQVERSRDDPTMILITYTSEHNHPWPLTSSTRNGPKPKPEPKPEPEPEVEPEAEEEDNKFMVLGRGIETTPSCVDEFAWFTEMETTSSTILESPIFSSEKKTAVSGADDVAVFFPMGEEDESLFADLGELPECSVVFRHRSSVVGSQVEIF

>AtWRKY66

MSLEIDAKAVSALLLGQGCANNLKTLLKNHETGSVSTEPLINSILDSFSFALSSQNIPRHVSQRSSKKKMCGIQGMEDSPTPAHIDGFIWRKYGQKTIKTSPHQRWYYRCAYAKDQNCDATKRVQKIQDNPPVYRNTYVGQHACEAPAYAVNNGGTYGSKMIKFDYVIPESVMPQPLSIDSQEITMEDKDTDDHILNYINEHLMEDEAYDVFPDVLGERCCFGLEPFPGLNINKS

>AtWRKY67

MVSNIDHKAMEALLRGQGCANNLKILLENGEISSVSTEPLIHTILDSFSLALSFMDSPNHPPYHESSSHNMASHMSRRSSKQVQHRRKLCVAEGLVNYNHDSRTMCPNDGFTWRKYGQKTIKASAHKRCYYRCTYAKDQNCNATKRVQKIKDNPPVYRTTYLGKHVCKAFAVHDDTYSSTMIRFDQVVPEPIMPQLTTIDHQVITVEENSAEHIMNQECDINDYLVDDDPFWASQFPPFPSSDTMFLENISAFD

>AtWRKY68

MENVGVGMPFYDLGQTRVYPLLSDFHDLSAERYPVGFMDLLGVHRHTPTHTPLMHFPTTPNSSSSEAVNGDDEEEEDGEEQQHKTKKRFKFTKMSRKQTKKKVPKVSFITRSEVLHLDDGYKWRKYGQKPVKDSPFPRNYYRCTTTWCDVKKRVERSFSDPSSVITTYEGQHTHPRPLLIMPKEGSSPSNGSASRAHIGLPTLPPQLLDYNNQQQQAPSSFGTEYINRQEKGINHDDDDDHVVKKSRTRDLLDGAGLVKDHGLLQDVVPSHIIKEEY

>AtWRKY69

MHRRAAIQESDDEEDETYNDVVPESPSSCEDSKISKPTPKKSRRNVEKRVVSVPIADVEGSKSRGEVYPPSDSWAWRKYGQKPIKGSPYPRGYYRCSSSKGCPARKQVERSRVDPSKLMITYACDHNHPFPSSSANTKSHHRSSVVLKTAKKEEEYEEEEEELTVTAAEEPPAGLDLSHVDSPLLLGGCYSEIGEFGWFYDASISSSSGSSNFLDVTLERGFSVGQEEDESLFGDLGDLPDCASVFRRGTVATEEQHRRCDFGAIPFCDSSR

>AtWRKY70

MDTNKAKKLKVMNQLVEGHDLTTQLQQLLSQPGSGLEDLVAKILVCFNNTISVLDTFEPISSSSSLAAVEGSQNASCDNDGKFEDSGDSRKRLGPVKGKRGCYKRKKRSETCTIESTILEDAFSWRKYGQKEILNAKFPRSYFRCTHKYTQGCKATKQVQKVELEPKMFSITYIGNHTCNTNAETPKSKTCDHHDEIFMDSEDHKSPSLSTSMKEEDNPHRHHGSSTENDLSLVWPEMVFEEDYHHQASYVNGKTSTSIDVLGSQDLMVFGGGGDFEFSENEHFSIFSSCSNLS

>AtWRKY71

MDDHVEHNYNTSLEEVHFKSLSDCLQSSLVMDYNSLEKVFKFSPYSSPFQSVSPSVNNPYLNLTSNSPVVSSSSNEGEPKENTNDKSDQMEDNEGDLHGVGESSKQLTKQGKKKGEKKEREVRVAFMTKSEIDHLEDGYRWRKYGQKAVKNSPYPRSYYRCTTQKCNVKKRVERSFQDPSIVITTYEGKHNHPIPSTLRGTVAAEHLLVHRGGGGSLLHSFPRHHQDFLMMKHSPANYQSVGSLSYEHGHGTSSYNFNNNQPVVDYGLLQDIVPSMFSKNES

>AtWRKY72

MEVLLKLPSSESPLKDKFGSVQIHEANKGDGDHQELESAKAEMSEVKEENEKLKGMLERIESDYKSLKLRFFDIIQQEPSNTATKNQNMVDHPKPTTTDLSSFDQERELVSLSLGRRSSSPSDSVPKKEEKTDAISAEVNADEELTKAGLTLGINNGNGGEPKEGLSMENRANSGSEEAWAPGKVTGKRSSPAPASGGDADGEAGQQNHVKRARVCVRARCDTPTMNDGCQWRKYGQKIAKGNPCPRAYYRCTVAPGCPVRKQVQRCADDMSILITTYEGTHSHSLPLSATTMASTTSAAASMLLSGSSSSPAAEMIGNNLYDNSRFNNNNKSFYSPTLHSPLHPTVTLDLTAPQHSSSSSSSLLSLNFNKFSNSFQRFPSTSLNFSSTSSTSSNPSTLNLPAIWGNGYSSYTPYPYNNVQFGTSNLGKTVQNSQSLTETLTKALTSDPSFHSVIAAAISTMVGSNGEQQIVGPRHSISNNIQQTNTTNNNKGCGGYFSSLLMSNIMASNQTGASLDQPSSQLPPFSMFKNSSSSSSTTNFVNKEEKS

>AtWRKY74

MEEVEAANKAAVESCHGVLNLLSQQTNDSKSIMVETREAVCKFKRVSSLLSRGLGQRKIKKLNNNNYKFSSSLLPQHMFLESPVCSNNAISGCIPILAPKPLQIVPAGPPPLMLFNQNMCLDKSFLELKPPSSRAVDPKPYQFIHTHQQGVYSRSKSGLNLKFDGSIGASCYSPSISNGSRSFVSSLSMDGSVTDYDRNSFHLIGLPQGSDHISQHSRRTSCSGSLKCGSKSKCHCSKKRKLRVKRSIKVPAISNKIADIPPDEYSWRKYGQKPIKGSPHPRGYYKCSSVRGCPARKHVERCVEETSMLIVTYEGEHNHSRILSSQSAHT

>AtWRKY75

MEGYDNGSLYAPFLSLKSHSKPELHQGEEESSKVRSEGCSKSVESSKKKGKKQRYAFQTRSQVDILDDGYRWRKYGQKAVKNNKFPRSYYRCTYGGCNVKKQVQRLTVDQEVVVTTYEGVHSHPIEKSTENFEHILTQMQIYSSF

Rice WRKYs

>OsWRKY1

MEADDSGGGGGRARRSVEVDFFSDEKKNMKKSRVSGGVAAEADDAKGPAAAGLAIKKEDLTINVSRRRLLRSSIARHLFNSRRSNACARPCSLQLLPAGNNARSDRSMVVDDDAASRPDHEEKSRSSNELAAMQAELGRMNEENQRLRGMLTQVTTSYQALQMHLVALMQQRPQMMQPPTQPEPPPPHQDGKAEGAVVPRQFLDLGPSSGAGGEAAEEPSNSSTEAGSPRRSSSTGNKDQERGDSPDAPSTAAAWLPGRAMAPQMGAAGAAGKSHDQQAQDANMRKARVSVRARSEAPIIADGCQWRKYGQKMAKGNPCPRAYYRCTMATGCPVRKQVQRCAEDRSILITTYEGTHNHPLPPAAMAMASTTSAAASMLLSGSMPSADGAAGLMSSNFLARTVLPCSSSMATISASAPFPTVTLDLTHAPPGAPNAVPLNAARPGAPAPQFQVPLPGGGMAPAFAVPPQVLYNQSKFSGLQMSSDSAEAAAAAAAAAQFAQPRPPIGQLPGPLSDTVSAAAAAITADPNFTVALAAAITSIIGGQHAAAAGNSNANNTNTNTTSNTNNTSSNNTTSNNTNSETQ

>OsWRKY2

MCDSLFWQSSADQGDLSDVVRASLQLQTAPRHQAASPPYVHLLGGGGGGGEDQLAAVSQHAEQQQQSMVDASAACDLLHALLPPPPVVQVQQQGASRTRTTIEEDTTGDGEELFAGAHYVVPPIKRRKSQTKKVVCIPAGASGGGGGEVVPSDLWAWRKYGQKPIKGSPYPRGYYRCSSSKGCSARKQVERSRADPTMLVVTYTSDHNHPWPTHRNALAGSTRPSSSNSSNIRLQDSTPVHHQSQTGHDRLTTTHLKQEDVIISPSLLQPDHHQLCTIIDTKHHLLFHQDYPHSFGLFD

>OsWRKY3

MAGAEWSPFDGDAAFAEYSSAVLAELGGWAAPGEEGGAGMMVPAALDLPVDVVGAAAREEEEEEEEAPARSGDGAAAAASSSSSGEPAAPDKRPAAAEAAPAAAATATATAKKGQKRARQPRFAFMTKSEIDHLEDGYRWRKYGQKAVKNSPFPRSYYRCTNSKCTVKKRVERSSDDPSVVITTYEGQHCHHTASFQRGVGGAAVAAHIHGAAAVALAEQMSAFVSPPPQPHMLYGLPRLHPPSSETAVSCSMPTTTSLQELNNSEGLQRPGYNNSPQAAVTIAQRPPSPSVPPAVSFDKGLLDDIVPPGVRLG

>OsWRKY4

MAEALVAVLRLAASAAATARPQSRSGRHGSCAARVPCPGPSPFRRGRLCARAAVAGPPEVDDDDAMTIDNLRRFFDVNVGKWNGAFYFFFFFFFFFFFFFSGFVVCKTETLSVLVRVPVCLCSSRQQFDAHGRVLQGISTRLSVSTYGEDDLISLLQSLYIKQASSQISFVDEEDSEEWVEYKIKETNMFTVDKYQQVGFFQEEKAFALRYQTAGMLETVLRAGVLGEDDTGEESPKNLKIPSRKPSIVCENCLYSREGNGRVRAFHIMDPKGVLDMLIIFHEKQGSEVPLMYSSDDADITNSDRIAPLLGRWEGRSVTKRSGVYGATLSEADTVVLLEKDRNGQLILDNMSTKSGSSTTTTVHWTGSANNNLLQFDGGYEMTLLPGGMYMGYPTDIGKIVNDMDSFHLEFCWMESPGKRQRLVRTYDSAGLAVSSTYFFETKRLSSLKPHHHLRRRVGDHGRTPDLSNANRVAGEGEGGDRFRRRRVAAVTAAAIDRAKSPEEGDSFRWFWKYSAQAVGASQSNPNTSRSDPLSWSAAVVVIDREALPLHKQRKLTRAAAVAMADRRRSDGGGGMQQQPFTSPGQERVFDGGGVPGQVAAPYGSDFDQSSYMALLAAGAVGVGVGVQPTAAPWAVEEDVAAAPPGISLAPQFSMANYAPPPSYQHPATLVSPPLAAGLHPYPPYLHGVDAPPPQWPPRPAPPPSFSVLDLAAAAAPHEQRHSMQQLLLRAAAFGGGMHAAAAPAPAAAAAIEQPAKDGYNWRKYGQKQLKDAESPRSYYKCTRDGCPVKKIVERSSDGCIKEITYKGRHSHPRPVEPRRGGAASSSSSAMAAGTDHNAGAAADDAAAADEDDPSDDDDTLLHEDDDDGEEGHDRGVDGEVGQRVVRKPKIILQTRSEVDLLDDGYRWRKYGQKVVKGNPRPRSYYKCTADGCNVRKQIERASADPKCVLTTYTGRHNHDPPGRPPAAANLQMPGPAAMRLAGGGTAHQQPSGGAHQMKEET

>OsWRKY5

MEMMVQKQRHEEGEEERGGLCAREIKELDFFSAAGAGAGRRDDDDVLRADGISSSHAGFMVSTALDLLTAVNDGDHHEEKKGQSNIHQSKQMDAAATTVEGELRQAGEENRRLRRRLEELTSSYGALYHQLVQAQQLHTKHQQQAPIAGVQLLDALAAASPASHRRRAAAAVDGDRTADSDGGEGDENVSPSLGSKRPAAAATLTRLTPESGSGGENNGGGEQAPAAEMAPCRKARVSVRARSEAPMISDGCQWRKYGQKMAKGNPCPRAYYRCTMASQCPVRKQVQRCAEDKSILITTYEGTHSHPLPPAAAAMAKTTSAAAAMLLSGPAVSRDALFAAHHHVVAPPPFFHHPYAGSTMATLSASAPFPTITLDLTQPPPTTTTTAAAAMLQLHRPYAFSSLPFSMYGAGGGSHRPPVVLPPPSSVVETMTAAITRDPNFTTAVAAALSSIMAGGGAQARTPPRGGSDAAGDINGGGGADHATAGARAAAAATQPCGTSPT

>OsWRKY6

MDGVEEANMAAVESSKKLVAILSKSGDPFRLMAAVAETDEAVSRFGKVVTILSNRVGHARARLGKRRSSPPVDPGCLMDHPLAAAASSPAPSNGRLHFSSSAATASPSPATAAAASSAANVTPAVVDRSLFLETTLLDLNSRGAPAPAASMAAAAKNSSKLAPAPMVNSSSSANHIQFQQPMKSFQFEQTPISDKFHIEMPRGVGGGGGKEVISFSFDNSVCTSSAATSFFTSISSQLISMSDAATNSAAAAAAPTTKKPSSCARKATADDDAGGKCHCPKKKKPREKKVVTVPAISDKVADIPSDNYSWRKYGQKPIKGSPHPRGYYRCSSKKDCPARKHVERCRSDPAMLLVTYENEHNHAQPLDLSVVQQATANPQT

>OsWRKY7

MAAVGAHAAVYHHPVSGLSAPAGDAAYSMSSYFSHGGSSTSSSASSFSAALAAATTPPLPDPSGSQFDISEFFFDDAPPAAVFNGAPTAALPDGAAANATRSAAEAVPAPAPAAVERPRTERIAFRTKSEIEILDDGYKWRKYGKKSVKNSPNPRNYYRCSTEGCNVKKRVERDKDDPSYVVTTYEGTHNHVSPSTVYYASQDAASGRFFVAGTQPPGSLN

>OsWRKY8

MSGPGGGGHGLYEDHPAAAGFLPFDHDDDVVASFFFGRSAASGGGAGAGAGAGDDDGVGLITPYSSITDYLQGFLQDPVYASSPLGGDAAVKHETVVDHPSQAGGVAAAPATPNSSVLSSSSEAAGGDDLRRCKKGRRPEDEEEEEIDDEGSAVQSCKTNKMKNKKGAKKEREPRVAFMTKSEVDHLEDGYRWRKYGQKAVKNSSYPSYYRCTAPRCGVKKRVERSEQDPSMVITTYEGQHTHPSPVSYHMHRQQGLMHVSARGVMPGAAGAYQFGAPPPPLLGFDEALAARSQDTVLVSQEDRLSQQQRSLYYRVDVQSCWAADRSRAHNCITKGKRKPGLGPNNTGATWELGRNPKTHRSRLAPGRTPHQQDPLRLLQRLGGDYLYRARRGVHFTHTTLLTRITQPVGISHVGRPPSFHDKAFPKPIQVYHMPTRGVPDQQQESVLKCESGKTVHPAQDVQRWLALLEIWSLILEILALRVFGLRNYTRNGTTQQTAKMKP

>OsWRKY9

MELRPPPPKHHHHRRRRGGGGEDGGEEEEEETGRLSLRGGGFWRRHDGEEEEEKGGGRRGEIKEVDFFLGASGRDVVVASRRHDDGFRGTTHGGGGGGDVNIGLDLLTTTTAGAAAGGAAAGAGEEDTGKNHRKEATTAAVDVELRRVVEENRRLRGMLDELNRSYSALYHQYLQVTQQQNHRHPDHHLIMNNNNNRPSLAQTHRTAATTTATTQQFLEPRASSTAQATADADMAASDDEAGRGGGDGDASSPSLSNAAGGGGGGNKMRRVGGQDETAAAAPARENGEQQAAAAAELPCRKPRVSVRARSEAPMISDGCQWRKYGQKMAKGNPCPRAYYRCTMAIGCPVRKQVQRCAEDKTVLITTYEGNHNHQLPPAATTMANTTSAAAAMLLSGPAASRDGAAAALLGHHHHHHPAAMFHQSFPYASTMATLSASAPFPTITLDLTQTPAGGAGAASLLHALHRPPVIHPGAAAQAMPFAVPPQLAMYLPQQRAAAAGLGGAGAARQPSVMETVTAALAADPNFTTALAAAISSVVAGGAHHQALSTTPRGSAAGAGDGNGNGSSAAAVATGAASPAATAEAPAASGSPPRLATQSCTTSN

>OsWRKY10

MAASLGLCHETSYAYSYPASNTSSSLCFPPLMADHIVDGGGGGGCSFGEFLELGHSVYSLPLPPPPSQPVVVAGGNNDQYGVSSSSSAAATTSRIGFRTRSEVEVLDDGFKWRKYGKKAVKSSPNPRNYYRCSAAGCGVKKRVERDGDDPRYVVTTYDGVHNHATPGCVGGGGHLPYPTSAAPPWSVPAAAASPPPAHAQAWGAPLHAAAAAHSSESSF

>OsWRKY11

MSSGGGGGGGGDRHGPYHQHGHLGRGEGADYVYSSSDMESFFFSQPGGVGIGGGGGGVVGAGGADEIMPYSSITDYLQGLLDPSGLARHLDVACPSSQDTVVKQELSVDVTSHDSQGTGGVAGEGVAQATPNSSASFSSSDGEAEGGKSSRRCKKGQAKAEEEDDKDEEDGENSKKPNKPKKKAEKRQRQPRVAFLTKSEVDHLEDGYRWRKYGQKAVKNSPYPRSYYRCTTPKCGVKKRVERSYQDPSTVITTYEGQHTHHSPASLRGGGGGVGIVGGHHHHHLFMPGVHGLPPSHLMPAGFHPELMGLMHHHPAMAAAAANPSMYFPGVAASAPPPPAVAGGGAMPPNDHPPLQQHHFTDYALLQDLFPSTMPSSNP

>OsWRKY12

MRGPLLLRAVVVVAMEHFNDWDLQAVVRSCSFPQSEPPRVGVGVPAAPGAGGAPVVVAPPARAPDGPDQMARASASALYDLEYLDLDHKPFLLPGSSSSSSSSRAVARARGEDDGKGRHEVMISFPAAAAASTSGAQPRSPSGRKPGIRTPRPKRRSSKKSQLKKVVYEVPVADGGVSSDLWAWRKYGQKPIKGSPYPRGYYKCSSMKGCMARKMVERSPAKPGMLVVTYMAEHCHPVPTQLNALAGTTRHKSAPTGDDDKPTSPGPAAGRAAAGEGVVKCEDVDGNELSAMAADGGAEDTAAAADDDGELWPEGMGLELDEFLGPMDDDVFEFDHVLEDDGVLGRRLSL

>OsWRKY13

MAAGEEVMDRSTSAEDGYCSAGTDSPRAESVDEQGAAEESSPRGGQKRELPSPSASPSSPLPPAAKRSRRSVEKRVVSVPIAECGDRPKGAGEGPPPSDSWAWRKYGQKPIKGSPYPRGYYRCSSSKGCPARKQVERSRADPTVLLVTYSFEHNHPWPQPKSSSCHASKSSPRSTAPKPEPVADGQHPEPAENESSASAELEVPEPEPEQESEPVVKQEEEQKEEQKAVVEPAAVTTTVAPAPAVEEEDENFDFGWIDQYHPTWHRSYAPLLPPEEWERELQGDDALFAGLGELPECAVVFGRRRELGLAATAPCS

>OsWRKY14

MDGEWSDGAAVSSPTMSGGGGREQMKGGEDVAAADCPGSPVSPSPAAAQRSAAGAAASPSGRSRRSAQKRVVTVPLADVTGPRPKGVGEGNTPTDSWAWRKYGQKPIKGSPFPRAYYRCSSSKGCPARKQVERSRNDPDTVIVTYSFEHNHSATVPRAQNRQAAPQKPKAQACSPPEPVVEVEPEETHQYGVTAGPATGGGGGAAAIEVRDEFRWLYDVVSVPATSTSPSDIDAADEMQLYDQPMFFGGAVVGTAALLPDEFGDVGGLGGEGLGEEEALFEGLGELPECAMVFRRRAGDGLEMGGGVKIEQPAESTAMT

>OsWRKY15

MAAGADERCRALVSGLLSSIDRSISIARSCCTEAAAAGRLTQQAGAAPESPPSADGSAGSDLGADSRCRANAAGPCKKRKTLPKWSKQVKVRSVQDVGPLDDGFSWRKYGQKDILGAKYPRAYFRCTHRHTQGCHASKQVQRADGDPLLFDVVYHGDHTCAHGVRSAAAAIDGQAAASAEQKHQPTPPQEQNAVSVAFTSMAVVNASTSSPFVSPAMSDCQISYELGGGSMAGVRNVPDVELASKTNSSMGDDMEFMFSLDSDFLDTYKYSSYF

>OsWRKY16

MGVAVHWRRAGDSLHMGGEPRARAEAAWAAALPAALVALVRDTATYTRMMHRLRMETPRGPTCQLDPCCCCCTAHVIPPPPPVSRTHATRRDAEASVPPPPASAAVSSRSDGTGQMAAGVTLACAAPPPLRAPRASDGGRRRGVVKGGAGTDTCRSPQRLNVRPRERERVRACVRARAKNHEHGQRRREAAVDPAMSGEYQFQDELAPLFARPGGGAGEMQMLPSSWFADYLQAGTPMQMDYDLMCRALELPVGEDVKREVGVVDVVAAGGGGAPPLTPNTTSSMSTSSSEGVGGGGGGGAGAGAGEEESPARCKKEEDENKEEGKGEEDEGHKNKKGSAAKGGKAGKGEKRARQPRFAFMTKSEVDHLEDGYRWRKYGQKAVKNSPYPRSYYRCTTQKCPVKKRVERSYQDPAVVITTYEGKHTHPIPATLRGSTHLLAAHAQAAAAAAAAHQLHHHHGHHGHHGMAPPLPLGSGAAAQFGRSSGIDVLSSFLPRAAAAHHGMTTMGGAAATTTTSHGLNSAISGGGGVSSETTSAVTVAASAQPSSPAALQMQHFMAQDLGLLQDMLLPSFIHGTNQP

>OsWRKY17

MDGAMQESREYWRDGGDVVGEELLREILDETAAVHSNSNSNSNSNSNSKEAEEEDEREYFAAAAADEQLQVEAPCGRRRRESMVNKLISTVYSGPTISDIESALSFTAAGDHQLLADGHNFAASSCSPVVFSPEKTLSKTMENKYTLKMKSCGNNGGLADDGYKWRKYGQKSIKNSPNPRSYYRCTNPRCNAKKQVERAVDEPDTLIVTYEGLHLHYTYSHFLHSTSSSSSSTTTQQQLQPQPQMMTNCKKKPKLHLHPLLHDDPPPPPPPPEMTTMMIMQSFSIQQQQHDDDQLLQPAADDHLMVQAPPDDCYNINGSSSSGLMMSLDDDEQAAGAGGLLEDVVPLLVRRPPPPICNNNNYYYSPATTCTSDNEYGSSASASPSSSVSVSSWTTPMSPCIDMAILSNIF

>OsWRKY18

MASRHQLQTMQFTDPASRSPRPVGGGVHGQPPPTPMSSPFSSRKPRMQEGHPTCVNLTPIPHTDGHLWRKYGEKKIKNSSFPRLYYRCSYRDDRNCMATKVVQQENDADPPLYRVTYIHPHTCNPSPPAPTPAHVFTEPPPAKAEVHHAVLFRFSSTAGGHTANNAVHRQQWQPAAATMAAGAQAQLSMTMSDDEREQPPAAIRSAPPARRLSMFRAVVDGLRQMRSSAPPTPSSSMVVDDGWDTFSSFDLDTCEFSVDDELLCGDHMYFPDSMQQ

>OsWRKY19

MVELCGGEGEGQIMLATELAQLRAMARELEAKMDPDRVAARELCRALASSVDRSIRLAASCFPPPEHPPPAAGNAGRDAAFKKRKGMAKVRRQVRVTSVQDTASLDDGLSWRKYGQKDILGAKYPRAYFRCTHRHTQGCNATKQVQRADGDPLLFDVVYLGDHTCGQAAVAAAAQSAPPEHAGQEQQRQSSLLAAGTEGIHQQVVAEPMAAPFLFTSTAAGGVDDGYFSFISPANSDCQFSSDFSAGSVGVDMDHEARFEDLFSSTLEFFQSEIQNL

>OsWRKY20

MAFGQDTIEQLYRELAGGRRLSAKLQALLEGPLDSRGQKEAVDVSRELGRVFMVSLYMLKPCSNSSRRPEGVTRTAPETRTDDSICLHTPARVKRVRSEEVLVRNGREEVVTRTEIITPSPYKDGYQWRKYGQKNIQDSNYLRLYFKCTFSRERSCAAKKQVQQRDAGEPPMFLVTYLNEHTCQQPQAVPGTPNTAGSSPTTTSRQRQSSSSPPAEMLDLTMNGAGLFSRLLLPHAVGGGGSAAEEEAAIVTCLAAVISGGGAAAAPPPLIWPTSAPEAAFVASAAGHSPSAADESVADEAAAAQMADMDYCFGQYDQSTFGAAAAADHRVLIGDDGDVQRIVAARIADTVWPRYTRDTSAWETAGTSSMRGSID

>OsWRKY21

MAMLGSSSAVVLELMTMGYQSAAYLGELLRAASPAQAGDEQQELAAEILRCCDRVIAKLNRGGATGATTGKKRKAAESAAAAAVTSPSLPVTPTKRRARGAEAVREVRSGTTTDGFIWRKYGQKEINGCKHPRLYYRCAFRGQGCLATRRVQQSQSQDDPAAAFVIAYYGEHTCGGDAAAAAACRDGELMPPAVINSGASSFAAAWNMASREPASSLAVERRSCDGDAPSETSQGWSPSFSSEVELDVVGFDLAGADSSASPVWEFLNGSFDWEFVINSL

>OsWRKY22

MVKRSDNMDSSSECSRGAHKRLLQDSRSYDQENAMKKVCIGTRTEYTYAPYHDGYQWRKYGQKMIRGNSFPRCYYRCTYHQDHGCPASKHVEQHNSEDPPLFRVIYTNEHTCGTSNSASDYMASSMQIQQIADASLRKAQAAERLRKAEVETPRLMHSPPPRCSGGYNMAMKEEKDVIVSSLLTVIRGCHIAESAGNNSAAALPVNRPPPAVARSDHYSCSYAISPELLPASDDLTLDFMLDSVLDPHWVEPLDLAWLKESTHTG

>OsWRKY23

MENLQLQGDDHDDEALPHFPYFAVPSPPPLAVAPAASATTSDGHQHGPLEVLEQPPCSNNLHPDGLVDGPQLAATTAVPMMLPAMTSLDWQSLLQTCLQVPPPVLEQQQPAAAAQADQYSGENDHGDLQAAESSGAGNKEKQVMAKGGAGRPSGTKKKASRPRFAFQTRSDNDILDDGYRWRKYGQKAVKNSKHPRSYYRCTHHTCNVKKQVQRLAKDTSIVVTTYEGVHNHPCEKLMEALTPILKQLQFLSQF

>OsWRKY24

MTTSSSGSVETSANSRLGTFSFASASFTDLLGGNAGAGGGGVSRYKAMTPPSLPLSPPPVSPSSFFNSPIGMNQADFLGSPVLLTSSIFPSPTTGAFASQHFDWRPEVAAAQSADQGGKDEQRNSYSDFSFQTAPASEEAVRTTTFQPPVPPAPLGDEAYRSQQQQQPWGYQQQPAGMDAGANAASFGAAPFQATSSEMAPQVQGGGGYSQPQSQRRSSDDGYNWRKYGQKQVKGSENPRSYYKCTFPNCPTKKKVERSLDGQITEIVYKGTHNHAKPQNTRRNSGSSAAQVLQSGGDMSEHSFGGMSGTAATPENSSASFGDDEIRVGSPRAGNGGGDEFDDDEPDSKRWRKDGDGEGISMAGNRTVREPRVVVQTMSDIDILDDGYRWRKYGQKVVKGNPNPRSYYKCTTAGCPVRKHVERASHDLRAVITTYEGKHNHDVPAARGSAALYRPAPPAAAATSSHPYLPNQPPPMSYQPTGPQPYALRPDGFGGQGPFGGVVGGSSFGGFSGFDDARGSYMSQHQQQQRQNDAMHASRAKEEPGDDMFFQNSLY

>OsWRKY25

MAVDLMGFSPRGGCRPSVETEQLAFQEAAAAGLRSLELLVSSLSAGGEHHHRRRPQEKQSSPPLGEIADQAVSRFRKVISILDRTGHARFRRGPVVGAAAAEAAAAAASASPSSSPVSPPLPPVTTQPAAAVKSLTLDFTNPAKVAAASVTSTSFFSSVTAGGDGSVSKGRSLVSSGKPPLAGGVKRKHPHPPCAAAGDGHGHGAGHAHAHGGCHCSKKRKQRVRRTVRVAAASARVADIPADEYSWRKYGQKPIKGSPYPRGYYRCSTVKGCPARKHVERAADDPATLVVTYEGDHRHSPPPPPLV

>OsWRKY26

MYMAAAAAGASTPFNFCRHGSHAEYDAVFSGSWMARRPSAAPHGGGASGSGSGSGYGAASYVAPTFGAAFRQQHLDLLDYLSDDQGVPAPPPAAVPSASYVTPAPAMAPAEPVVPDAVAAAGGYPRSVAAAAAAVAGEGRDRTTTDKIAFRTRSDDEILDDGYKWRKYGKKSVKNSPNPRNYYRCSTEGCNVKKRVERDKNDPRYVVTMYEGIHNHVCPGTVYYAAQDAASGRFFVAGISHPDLN

>OsWRKY27

MNTFTLFRDEVQKASEKVHRHHANDDEAGLFLSLGLSLGSSPDACQCHASKKDEADAGNGGGDGYLALALRCAPAAGEPMVHPKRQRATTNSSSSSSICGEYGGGAAAAAVPAGHDDDDRSCMITAASTANRPGRVVLRTRCSAPTVKDGCQWRKYGQKTAKGNPWPRGYYRCTGAPGCPVKKQVQRCNHDTSVLVTTYDGVHNHPITPYAAALPPSSSSSSSAAVAMLASSSSSSTWSELQRAMPAAQSSWSQRNYPIQADVVAKAIWDPKFQATVAAAVASYVRDREQSARVAGGKGAGELFNLAPPC

>OsWRKY28

MAKMLPPPSQSVPSRPPSWLYIPPRRRHGTFTSSCAFRLSPSSPSSPPPPVLDFQYIQFMDSWIEQTSLSLDLNVGLPSTARRSSAPAAPIKVLVEENFLSFKKDHEVEALEAELRRASEENKKLTEMLRAVVAKYTELQGQVNDMMSAAAAAAVNAGNHQSSTSEGGSVSPSRKRIRSVDSLDDAAHHRKPSPPFVAAAAAAAYASPDQMECTSAAAAAAAKRVVREDCKPKVSKRFVHADPSDLSLVVKDGYQWRKYGQKVTKDNPCPRAYFRCSFAPACPVKKKVQRSADDNTVLVATYEGEHNHAQPPHHDAGSKTAAAAKHSQHQPPPSAAAAVVRQQQEQAAAAGPSTEVAARKNLAEQMAATLTRDPGFKAALVTALSGRILELSPTKN

>OsWRKY29

MAMAGAGDWPFAADEAYADSSAIFAELGWANGLAVVDAVGELLPPLDPPGELATPPPPPLDLPETPAGSSADGAASSCSTDDADGGKPAAASTEAASKSLTPGKKRARQPRFAFMTKSEIDHLEDGYRWRKYGQKAVKNSPFPRSYYRCTNSKCTVKKRVERSSDDPSVVITTYEGQHSHHTVTFPRAAATAAGFSHIHAMAALAAAPFSAHQQLYSNLQPPPPTMPLAATTPASSSSLLQLPLHCNHELQVVASCGGYPSSSSSPPASVLPVDKGLLDDMVPRAMRHDG

>OsWRKY30

MDGTNNHGALMDDWMLPSPSPRTLMSSFLNEEFSSGPFSDIFCDNGSNKHQDGLGKSKAFIDSSREETAQLAKKFESNLFGANQKSSSNGCLSERMAARTGFGVLKIDTSRVGYSTPIRSPVTIPPGVSPRELLESPVFLPNAIAQPSPTTGKLPFLMHSNVKPSIPKKTEDETRHDRVFFFQPILGSKPPTCPVAEKGFSVNHQNQPSVTDNHQELSLQSSSTAAKDFTSATIVKPKTSDSMLDNDDHPSPANDQEENATNKNEEYSSDLIITPAEDGYNWRKYGQKQVKNSEHPRSYYKCTFTNCAVKKVERSQDGQITEIVYKGSHNHPLPPSNRRPNVPFSHFNDLRDDHSEKFGSKSGQATATSWENAANGHLQDVGSEVLTKLSASLTTTEHAEKSVMDKQEAVDISSTLSNEEDDRVTHRAPLSLGFDANDDYVEHKRRKMDVYAATSTSTNAIDIGAVASRAIREPRVVVQTTSEVDILDDGYRWRKYGQKVVKGNPNPRSYYKCTHPGCSVRKHVERSSHDLKSVITTYEGKHNHEVPAARNSGHPSSGSAAAPQATNGLLHRRPEPAQGGGGGSLAQFGYGSAGHRPAEQFGAAAAGFSFGMLPRSIATPAPSPAIAVPAMQGYPGLVLPRGEMKVNLLPQSGNAGAAASQQLMGRLPKQHPQM

>OsWRKY31

MDGDAWWYPGGGGGGGSNNWDLGAVVRFGCGGGRVSPAAALLGEAWEYDDDPFSSFLAPPMTAQQAALPAVWEEGDDGDAAWMAPLPGLQTGGGWGDQAPMVVDELCGALVVAPPPPPKQQEVLQVQQQPPPADNTQPTTYQQGSGGDGESTRAGGSRKKQTRKEVVRVAASGPAPDLWAWRKYGQKPIKGSPYPRGYYRCSSNKNCAARKQVERCRFDPSFLLLTYTGAHSGHDVPLHRNSLAGTTRHKPPPPPPLPSAADKSPATAAEAATASQSPGLSPTTPLRASSMELHGEDDAEAELQVEEDDMAIDDEDDDDVADETISTVPWGTPISDAIIAASYEWR

>OsWRKY32

MSSKKKRAAIDLSLEAERRRPEERGGGSDREASDGAAAAAEEDGDVKQREGPKEETGGEEEKVVEVVVDQGEDGSNEEIKYRTQQAEMIEEDKQPAAAANVDDDGGDSDGVGASAEEKHMVTEATGGEGDDGGDSRTPMAQDELSEMQEEMERMKEENRMLRRVVDKTVRDYYELQMKLAAYQQQPAAADEPKETEVFLSLGATAAASAGCGGGFPEAKSKEQAAWRRRSVGSDDSDCGKEDLGLSLSLGASSSYDDDQKAVEARPHDVDGAAAAAMIGGDGSRPAPRGYALLESSKVQGGAAPAAGELAAAGGITSQSVNPANRKTRVSVRVRCQGPTMNDGCQWRKYGQKVAKGNPCPRAYYRCTVAPGCPVRKQVQRCLEDMSILVTTYEGTHNHPLPVGATAMASTTSAAATFMLLSSTTSSSSVSDASAAPSSSYLSPYLLNSASPLLMPGATGGGGGMQHLNLFGNSPSSSSLLAPQAPGSSKYPWSPNHPPLAGAGGNKRPFWSAGGDGDKPAPAALAENVGAVMSDPNKFSAAIAAAINNFMGKDGESSSGKSSTFAWTRSMDCTATPASVPAPARPRREPVHGPGPRRGEQAAKL

>OsWRKY34

MHTCMEGGGQLGTCLPNFYLLPDHHGMPLPPPLQLPCHPKLLQMPFDQEDQPGIHGVMLSSDHCGLYPLPALPLSNSAAAAAATVALGKHSAAAGSMPNIGGAEEVATTVTKAGNESTTCNGSTTWWRGSTMAAMGEKGKMKIRRKMREPRFCFQTRSEVDVLDDGYKWRKYGQKVVKNSLHPRYICST

>OsWRKY35

MTMTTATLDPPPPPLLIAGSLLDDDRDAGSAASSSPRWLPRARGSTVGSPPRRGGEERGGEAATRRKGNSSCPDDDDDCRNDEDDCRNDDDYRDDDVGATTDGGGDAAALLPMAMMTATTMAPMPVEAAVDGGGELSSAGEGGQRRVNLLPTKILSAYENHLFSQALACREDEWIIEKLAVIAARRSSFVSEESLCSVPAAALVSLAIAHLAFSPMLVYGVLDRLCSVCHLGEMEGDIAMEEWKDSNHRGADYLMTMPMQNFLADAFPPPELLEGEGGFEKHGLSVAVGSPPPTPPPPEDGCSPLPLTPQFGQKFGSGGGGGGSLADRRARGGFSNVARISVPYNQPAADVSSAGAPSPYVTIPPGLSPTTLLESPVFSNAMGQASPTTGKLHMLGGANDSNPIRFESPRIEEGSGAFSFKPLNLASSHYAAEEKTKSLPNNQHQSLPISVKTEATSIQTAQDEAAANQLMQPQFNGGKRSRAAPDNGGDGEGQPAEGDAKADSSSGAAAVAVVAAAAAAVAEDGYSWRKYGQKQVKHSEYPRSYYKCTHASCAVKKKVERSHEGHVTEIIYKGTHNHPKPAASRRPPVHPPPPSPATTTTTPLPPGDAQADHAPDGGGGSTPVGAGQAGAEWHNGGVVGGEGLVDATSSPSVPGELCESTASMQVHEGAAAAQLGESPEGVDVTSAVSDEVDRDDKATHVLPLAAAAADGESDELERKRRKLDSCATMDMSTASRAVREPRVVIQTTSEVDILDDGYRWRKYGQKVVKGNPNPRSYYKCTHPGCLVRKHVERASHDLKSVITTYEGKHNHEVPAARNSGHPAGSASPGGGAGSSSQPHGVGVGGRRPEVPSVQESLMRLGGGCGAAPFPPHFGLHLPPPPPRDPLAPMSNFPYSLGHAPSPALRGLPPPPPPPPSASALAVAGLGGVVEGLKYPMLAPPSVHSLLRHRQGGGMEAVVVPKAEVKQEAMRPAAAVAGAGRGAAVYQQAMSRVSLGNQL

>OsWRKY36

MYACMEGSQLETACLPAALYAPLCPYTPPSPPSFLAPLPSLQHKLPQLPQLVHDHAAATGTNHGVMFSSDHGCLYPLLPGIPFCLDSGCGAAACDDDKPAGFAHLGSAEADTSAAAARVDSEIAAAATATTCHGPNSWWKGTEKGKMKVRRKMREPRFCFQTRSDVDVLDDGYKWRKYGQKVVKNSLHPRSYYRCTHNNCRVKKRVERLSEDCRMVITTYEGRHTHTPCSDDATTGAAGDHTASCAFTSF

>OsWRKY37

MPPCVWGSHTPLSLLSPPAAWAGERMEGDQAGGDLTDIVRAGGGAMPGSVVVDLPSTAAEWQLPAEPMLFPPPPSLSSTTDGCGAGGAAGADIFGGGGGDLFSGLVDPFSSDYSSGADFLDAMPDAMAKVGFDTAVGGGCGGGGGGGGGSGGHLLDMSRKPLLPRGMPMAAVGGLAAPRVMPSPLSPRAIRPYPPISAGDMMKLGITAGQAAGCAIDAAVAGMQMSSPRSGGIKRRKNQARKVVCIPAPTAAGGRPSGEVVPSDLWAWRKYGQKPIKGSPYPRGYYRCSSSKGCSARKQVERSRTDPNMLVITYTSEHNHPWPTQRNALAGSTRSHHSKNSGGGGGSGSKGSQNDKSQQQPSVKEEQKDQATTATTTTTSTITTTNSASPVVVKEEEAALAGSSEALELERVMDTTAAGVVDHSELMDHVFSESYKPMIPETGQPDDFFADLAELESDPMSLIFSKEYMEAKPSGGDHAQEKAMAKELDPFDMLDWSTTTNSSAGSSFEQGKRG

>OsWRKY39

MEEELCGNNWDLDAVVRLGCCRRRISPAAVAQQVDPFASFLQQGVAMEVAAEKEVGVEAAWSFPELTVRDGGGGGLGRDADELLKAFCAAFPSSSSSKSSPLPTPPPPPPTQPQPEQQKPVTVQENLPAPTTAPARASQPAAARQVPAGGVPRSKRRKNQQKKVVRHVPADGVSADVWAWRKYGQKPIKGSPYPRGYYRCSSSKGCPARKQVERSRSDPNTFILTYTGEHNHSAPTHRNSLAGTTRNKLPSSSAASAASAQPQPPPPSVVVVGAGGGGAEAAGLSPTTPLRTPSMEEDEEEEEEEELLVEDMEMAGEDELLFLNGGDDNAALDGTPMSSLFDIADEPFLPSPWTEPTAAGS

>OsWRKY40

MKNSSNKRSLVADQWHPSSVCCDHRAALREIAKGQSLVTQLRAIVLPALHSDERCDLAAQMLEGILDCSRKAVSQLQLLLSSPHDDDDHHHVDDKRRVRKIISSSDDDDHCSSKAAEDDNAKPLRQHKRRRFGDSVSLETPVPHYDGHQWRKYGQKHINNSKHPRSYYRCTYRQEEKCKATKTVQQREDLHHANSYNGDHPIMYTVVYYGQHTCCKGPAALADDHVVVEASQISTDSHCQSPSSSSDLQAAEVHAGNSSQCSNISVTCSPSVVVEDCNKLLDMMPAADELTADVLLFDMTAYAPLDLDINWEMDTNALWV

>OsWRKY41

MESTCHVGIGAAMNKLENLLDLCGEEKLKVVSIVGVGGVGKTTLANKLYRKLRWQFECRAFVRTSQKTDMRRLLINILLQIRSHQSPDNWKVHSLISSIRTYLQDKRGCVCCLLSLISALATIRPHLAKDIPPIKGFLIVIDDLWATSTWDIIKCALPEGNKSSRILTTTEIEDLALQSCSYDLKFIFKMIPFGEDDSRKLLFSIVFGSHSKCPPEVSETLYDIVRKCGGLPLAIVTVASLLASQLDKLEQWDYINKSLGYSLMANPTLEGMKQLLNLCYNNLPQHLKACMLYLSMYQGDHIIWKDDLVNQWIAEGFICATEEHDKEEISRGYFDELVGRKIIQPVHIDDSGEVLSCVVHHIVLNFVTYKSIEENFIIAIDHSQATIRFADKVRRLSIHFNNVEDAPPPTNMRLFQVRTIAFFGVLKYMPFIMEFRLIKVLFLHFLGDEDSTGIVDLTKISELVRLRYLKVTSNATVKLPTRLQGLPYLETLKIDGKISEVPTDIYLPAVTQRRIYGALVS

>OsWRKY42

MADPFPAAARGGEQGGGTAGQLVSTPSRLRTAVASMLNRTGHARFRRAAPVVVQEEEDEAAAAARDAVVRCDGLSASASSSFPSSVTGVTGDGSVSNARAVLPAAGDGDKPPPMQSASDYASDGRLKRSSDDDGERCHCSKKKRKASWRARRRIRVPAISSRNADIPADDYSWRKYGQKPIKGSPYPRGYYKCSTVRGCPARKHVERDPGEPAMLIVTYDGDHRHGEPGHRRPDEAATTTEHRTTDQTTGRLL

>OsWRKY43

MESYVGVKGKNVVGGGDVGREMPVAPPSSSSAAVGMVEFPAAAAGLGYAGMTAKEAGGGYQERRVVVGEMDFFKTAEKRGERKEPPPATATAAASGHAGASPDDLSLNKDDLTINMGLLVGRRRNSGSEESIVDDGGVSSNDEEHREAKAALAVTKAEIGRLSEENKRLKNMLSNVTTKYNSLQMQFVTLMQQRRSVLAAPIHQQELLDPEKKEQEGSQQQQQQLIPRQFISLGSASLQPDVEAPHSVVVVGGDVCAPSSSNPDAAVPAMMPLPHFDHHNHHHPIHGGRERGSSPAEADHHRHHQQEQPPPPPQQQQQLPPSWLPADKVPRFLPGKGPEPVPEAATMRKARVSVRARSDAPMISDGCQWRKYGQKMAKGNPCPRAYYRCTMAAGCPVRKQVQRCAEDRTVLITTYEGNHNHPLPPAAMAMASTTAAAASMLLSGSMPSADGSLMAGSNFLARAVLPCSSTVATISASAPFPTVTLDLTQTAPPPPPASSTQPQPPRPEPAQLQAALAEAARPVALPQLFGQKLYDQSKLSAVQAVAGTKGSDGGALADTVNAATAAIASDPNFTAVLAAALTSYIGSRSGSGGAGAGGSSGTVQPLMSGGGDSCSRDDKIGEQNS

>OsWRKY45

MTSSMSPAPAPAYAQVMEDMEKGKELAAQLQGLLRDSPEAGRFVDQILHTFSRAMRALDKAAVSAAGGEGSEVQSEVTCGGGASAGGKRKAPAADRKANCRRRTQQSSGNSVVVKNLDDGQAWRKYGQKEIQNSKHPKAYFRCTHKYDQLCTAQRQVQRCDDDPASYRVTYIGEHTCRDPATAPIIAAHVIHQVAAGDNDDGCGGLQAGSRLISFVAAPAAPVDAAAAPTTSTITTVTAPGPLLQPLKVEGGVGSSDQEEVLSSLTPGSSAARGGGGGGGVAGPFGPDQGDVTSSLHWSYDAVAGMEFFKNDEVVFDLDDIMGLSF

>OsWRKY46

MALDSVPSYPSDLGSSRARTPQQQRVSPRKEERTWTTDTYAPYDDGHQWRKYGEKKLSNSNFPRFYYRCTYKNDMKCPATKQVQQKDTNDPPLFSVTYFNHHTCNSSSKIVGSTPDSTVQSRKAISICFNSHGQTGEQPTFLSSSASLLSPSMQSYSSNQQPDMNTYSRQFQWADTSSSTSNAPVKMEADDYAEASASPSTTGALSRTLLPIGQSRCIEYFHFL

>OsWRKY47

MASPDGGVGDGGAEPHEVMDDLLEMREQAAMLHSMLHGTSPSSCAAAASTRQLNQLIDGVMSRLQSSSLSVMSPGGGGGRRGSGGRKKKGAKAVAGPHRRSSSGRRRSKSPFVRMVTTKELEDGRQWRKYGQKHIQDSPNNPRSYYRCTHRPDQGCMATKQVQTSESNSSEFVISYYGEHTCSDPSTIPFVVEAEAPAADYANLISFGSSGGASTSRVDPLRQSRHRLMAEAVDPTPSCSFANCHSPVLSSECASEAAALSSSLPLSAVVGSAVTTPSTSIVGSAPADYDWPSGLAGGDMAGSFPSSPSSLGFMTGSFGNLPGDDDDMFGFDP

>OsWRKY48

MALIATGATATATAAPVASPAASSMASELMAQGRESAAVLEALLHGASLPPAHGGAHALAAEILRCCDRALAALRAGGDAESSSADTKRKPATAQPSTRRRRRATASGGGAAAAAEPARVEKARTSEDGFLWRKYGQKEIKNSKHPRLYYRCSYKDDHGCTATKQVQQSEEDPSLYVITYFGDHTCSCQTAAAAAMDDDDDDENSQHFVINFGPATASRSGSPPLLYDDGDDGDVWRETAATPPSSRQSRCSPEGDGEESGVKMSKEEPVDSCPGPSAVSSPADVVSCSSPAMEPDLLGCLNWDDDFGDSSFVDADEFMNFDEIDLFQIYS

>OsWRKY49

MSGGGGGGEGFPFHDELASLFAERPPNGAMPGMLQQQQPWSFIDYHHHLMQESAPTTPPLDYEAFAGEFDDDVAPLEEVKRELVVDGVGLFPGGGASAAAAAAAVAGPMTPNSMSVSSTSSEACGVGGGAGGDEESAGKCKKEEEGDGGDDDGKEGSSTTKGDGDGEDKNKKGGKGKGKGEKRPRQPRFAFMTKSEVDHLEDGYRWRKYGQKAVKNSPFPRSYYRCTTQKCPVKKRVERSYQDAAVVITTYEGKHTHPIPATLRGTAHLLGAAAAAHHHGGLQYHHPGHFAAAVGHRLPPQPHDALGGGLLAPPHAQHLHAMQHQMQLAAAAAASGGSLHAAAMQQMPQPDHAGLVAIIASTTGASTTPPPPPATGSAAAATTPLRMQHFMAQDYGLLQDMFIPSPFLHNDDANNNNHR

>OsWRKY50

MEEAYCMMMVGRERELVAELRHLLFPSPSPTPTTPASHSTTALAGDGECCLPPGLTTTTTVSGGGRRRGRKRVNRDNDNVKLLLQADDDQEAVIADHGDANAKPLPNFTKTRRRKQQATTSTMVTTVPDFDGYQWRKYGQKQIEGALYPRSYYRCTNSTNQGCLAKKTVQRNGGGGAAGYTVAYISEHTCKSIEPSLPPVILDTTVRTTNNHQQPAAAESPAATSSSSSNMVMTSSETGNWSGQHGAYACRQMIAADEEYCCWDTPATTTTTSGSNGGNSTCAEDIELLSRPIRSPMHIAAEGNWMDDLLLVTDGLIVISNSSITHFLT

>OsWRKY51

MITMDLMGGYGRVDEQVAIQEAAAAGLRGMEHLILQLSQTGTSERSPAPAQEQQQQVDCREITDMTVSKFKKVISMLNRTGHARFRRGPVVAQSSGPAASEPAPVRSSPSAVSRPMTLDFTKAASGYGKDAGFSVSGISAASSSFLSSVTGDGSVSNGRGGGSSSLMLPPPPATSCGKPPLSSAAAAMSAGAGHKRKCHDHAHSENVAGGKYGSTGGRCHCSKRRKHRVKRTIRVPAISSKVADIPADDFSWRKYGQKPIKGSPFPRGYYKCSTLRGCPARKHVERDPTDPSMLIVTYEGEHRHSPSAAGQDHPPAPPPPLALPLA

>OsWRKY52

MAVTESACLSYEQEAVAVREVAQVYELIKTQQPLLLVHQQPQQLAHGLLNHALRALNVALSVMNQPHASSSAAAAAVPVMSLIKAEAATPANSSSPAADVAADNHVVGKPRRSSSAAKRRRINGEEYKSSSWSQFTPVPHEDGFQWRKYGEKKIQGTHFTRSYFRCTYRDDRGCQATKQIQQKDKNDPPMFQVTYSNEHTCTTTRLINNINNPAALHNLTANPNGHHDSDDDDTIFTKMIKQEEQAAWLPPPPPADLATISNNFDETPGLHVCQEVPPSSSNSSVISHYADEFDHHQMLETTVMEEALGLGADLDDPYFYDPNLLLIYESLMNCY

>OsWRKY53

MASSTGGLDHGFTFTPPPFITSFTELLSGGGGDLLGAGGEERSPRGFSRGGARVGGGVPKFKSAQPPSLPLSPPPVSPSSYFAIPPGLSPTELLDSPVLLSSSHILASPTTGAIPAQRYDWKASADLIASQQDDSRGDFSFHTNSDAMAAQPASFPSFKEQEQQVVESSKNGAAAASSNKSGGGGNNKLEDGYNWRKYGQKQVKGSENPRSYYKCTYNGCSMKKKVERSLADGRITQIVYKGAHNHPKPLSTRRNASSCATAAACADDLAAPGAGADQYSAATPENSSVTFGDDEADNASHRSEGDEPEAKRWKEDADNEGSSGGMGGGAGGKPVREPRLVVQTLSDIDILDDGFRWRKYGQKVVKGNPNPRSYYKCTTVGCPVRKHVERASHDTRAVITTYEGKHNHDVPVGRGGGGGRAPAPAPPTSGAIRPSAVAAAQQGPYTLEMLPNPAGLYGGYGAGAGGAAFPRTKDERRDDLFVESLLC

>OsWRKY54

MALTMVSLAAIAGKPTAASAQLVAEGRESAARLYALLVGSSALHGPVGLAEQILLCFDRALAKLHGVNLAGAEDDDDAAGNGNGRKRKPGRGRGLTAASAAASSKRMRVSNAGGNGARIERKATMDDKFLWRKYGQKEIKNSKHPRFYYRCSYKDDHGCTATKQVQQSETADDDTASPVYIITYFGEHTCRHGDDAAAMVVDGGEEEDQLSPAQMVISFASSNGGDASVSWPCSGDDAQNNSETSHESSPPEAPAGEEERLRPCTAAGVSDEPIMESTPPAPELLADLKPMDGCLLDGESLFGMDELVYFHELSAALGLLDRDWGAPV

>OsWRKY55

MSPVPSPHQSHHLGHGSRKEKRMRKVDTFAPHNDGHQWRKYGEKKINNCNFPRYYYRCTYKDNMNCPATKQIQQKDYSDPPLYSVTYYNEHTCNSAFLPLSPSEFQLQTASGKAVSICFESSGAQEPMTNASSPSSSAARRSTPSENKNQPLPRHSEAYSWGVGVVEQKPSCTELQSCSTECQDAFSAGTIPEETVDAGRFGSIRFFHFL

>OsWRKY56

MYGQSPHKQRRRFPGPYLMGDVLRAQATASAEEVAGGVWPCELDDHLIGELLGDDGLFVPAAEHPTLYYSFGAGSSAAAAAAPCNGGGSADHERRPRPAPAVSRDLCSVYSGPTIRDIEKALSSSASPRPPYPSGRRYSSLYFRRVEAESKYTSKVRSCGGKMPADGYKWRKYGQKSIKNNPHPRCATRSIIDPI

>OsWRKY57

MAHGGGEEEEERVLSHGDVVLLRCDLTILRGPHFLNDRIIAFYLAHLAADHHDDDLLLLPPSVPYLLSNLPDPASVAAVADPLRLASRRLVLLPVNDNPDVSHAEGGSHWTLLVLDNSNAVSGPRFVHHDSLPPTNLPSARRLAAVLRPLLPASAIPLIEGPTPRQTNGYDCGVFVLAVARAICNWWPTRARHSNSDSDWLEAVKREVNADSVKAMRTQLLQLIHTLIQNNTTTNQHSPSTQLPSHPSIASTSLPVTAQDFGTWLEDSGTHTAYDQKKADTGKGACWDNLTVSQSVRKPNVSAKNSLSYDGYSWRKYGQKQVKGSEFPRSYYKCTHPTCPVKRKVEMTPDGRIAEIVYNGEHNHPKPHPPRKPTLSTSVETLVATNDAGLENKLEGCDQAIGSDAVVEALRGGCHCLDGFRNGNEISDCKKRYAYAVIFIQNLLMFFCAKNVQVVA

>OsWRKY58

MDGLEAAAGDQQHGRLLIPQLPAAYLASSSMAALSPAGDDWAASLILPDGGSAAAGVGEDDLGGGVMAAAAAESSCGGSSTVTSSGVTEAAAAAATTTRRGRGNGKKAGGGGRTPRFAFHTRSENDILDDGYRWRKYGQKAVKNSDFPSDDELLLFSDVDNTQTATENLRFIPLGRVYITG

>OsWRKY60

MASNSQTTTTGAGGGRGQGDDEEPTPTPPPAPPPETAPSTVGGGGDGVQLVMPEDGYEWKKYGQKFIKNIQKNRSYFRCRDQRCGAKKKVEWHPHDPGLNLRVVYDGAHHHGSPSSAAGEGGTSAAAAANQYDLSTQYFGGAGGPRSQ

>OsWRKY61

MGVLKVGAYINLCILHNRKEPQMERRKVIRMSYTEDDGFSWRKYGQKDVEGAMHPTTQSNYFRCAHKMTTGCKARKKVQRTDGDPLMVDVVYKGVHSCAGVHSDSQRSSAASSKSNLRPTKSMQVRASSKDVGPPDDGYSWKRYGQKNIFGANYPRCYYRCIHKTTTGCTATKNAQATDGDPLLFDVVYHGEHTCDLQSTHSNDVEPIRPQSGLDDDMCTDDTTTVSTRHDSNTDASSISFQLDWTNCKDESDGPPTTL

>OsWRKY62

MDDDGDGSSSPTDDSAAAGLLPLFSRSPAEDLEEKLRRAMEENARLTRALDAILAGHHAHQRALLAPSLSPPPPSATARAPSVSTSCAAREDAAPAVAAAAASTACPSRQQPPTAEPRPKVRTVRVRADAADATDANSMAETVKDGYQWRKYGQKVTRDNPYPRAYFRCAFAPSCPVKKKLQRCAEDRSMLVATYEGEHNHALSTQTTEFVASGCTTSQHAGGSSSSPLPCSISINSSGRTITLDLTNQAGSGSIASCGVEAAAVSGELVTVLSPELRRHLVEEVVQVLKNDAEFVEAVTNAVAARVVDQIPHIPVHL

>OsWRKY63

MWHVRSNTEDDGLSWSKYEQKEILGAKFPRAYFRCTHWNTKKGCMATKEVQRDDGDPLMFDIVYHGEHTCTQTAESNVDEQIRLTRTRDKKVKRTKRKRQVRVTSVPADDGYSWRKYGQKNVLGFSYLRGYYRCATKGCQASKQVQRHDDGLLFDVTYFGEHTCADQPQAAHSSDQSTILERPTFKTRLQGQVDAMEKQHEGFRKMLSSIQEAIGAMSVKQGEVQETMNKMEKSISSWRPQVDAAVQSLQRDMELQRNQVGAVERHQAEADKSSNTSQALEEREEIARRTSQLPTSPTMSLPVSQALEEREEIARRAPLLPTPPTASMPASTGEIGLDGHRISTQFRGRASGVVTTLVPPPGKGFVTVTSVKTTDSMDGEDVASRPGPGAGRWHWAPGS

>OsWRKY64

MKNSSNKRPLVADQWHPSSVCCDHRAALREIARGQSLVTQLRAIVLPALHSDERGDLAAQMLEGILDCSRKAISELQLQLSSDSPHDDDGHLDDKRRVRKIVSSSSDDDDHSSSKAAEDHNAKPLRQHKRRRFGDSVSLETPVPHYDGHQWRKYGQKHINNSKHPRSYYRCTYRQEEKCKATKTVQQREDLHHANSYNGDHPVMYTVVYYGQHTCCKGPAASADDHVVVEASQISTDSHCQSPGSSSSELQAAAHAGDSSQCSNISVTCSSSVVVEDCNKLLDMLPAADELTTDVLLFDMTAYAPLDLDINWEMDTNALWA

>OsWRKY65

MEEAYCMMMVGRERELVAELRHLLFPSPSPTTPASHSTTALTGDDECLPPGLTTTTTVSGGGGRRRGRKRVRRDNDNLKLLQADDDQEVLAAAVHGDANAKPLPNFTKTSRRKLQTTTSTMVTTVPDFDGYQWRKYGQKQIEGAMYPRSYYRCTNSTNQGCLAKKTVQRNGGGGAAGYTVAYISEHTCKSIEPSLPPVILDTTVRATNNHHPPAASSSCAAQSPAAAATSSSSDMMMTSTSSTSSETGNWSGQHGAYACRRQMIAADEEYCCWDTPATTTTSGSDGGNSSTCADQVIDLMSGPIRSPLHIAADGNWVDDLLLVGDGLIDISSANITHLFSF

>OsWRKY66

MCDYFLQRMEGEQAAGDLADIVLRAGGAAAAAVAGGGIPSTEWQLPPAEEEEEEPGLFPLPPSSSDGSGMSGADAFGDPFAGLPDPFGGDYPSSGGAAAAADFFDAVVAKAGFVDVGVLGGGGGGGCDGGGVDGGGGGSSLLGMSKPILPRAAMQLPSVSPRAIRPYPVMAGDTVKLGAPMAGGPCAFDGAAAAGLHMSSSPRGAVGGIKRRKNQARKVVCIPAPAAAGGRTSGEVVPSDLWAWRKYGQKPIKGSPYPRGYYRCSSSKGCSARKQVERSRTDPNMLVITYTSEHNHPWPTQRNALAGSTRSHHAKNSSSNSSSSGASSASKNNSSHSGYHHHHHQKPLVKAEPNDQSAAATTAATVPVKEEAAMVGTSSEALAKTTQKSMEDAAAAASATAAAVEHSDLMQQMFSQSYRPMIPEAAAGGHHDDFFADLAELESDPMSLIFSKEYMATNYKPAGDPAGKEMNAVDKGLDPAYMLDWSSTTVVTRAGGSSFMQGEGGL

>OsWRKY67

MAASVGLNPEAFFFSNSYSYSSSPFMASYTPEFSAAAIDANLFSGELDFDCSLPAPAQEYPENENTMMRYESEEKMRARVNGRIGFRTRSEVEILDDGFKWRKYGKKAVKNSPNPRNYYRCSTEGCNVKKRVERDREDHRYVITTYDGVHNHASPAAAAAALQYAAAAGDYYSPPLSSAGSPPAAYSAGGSLLF

>OsWRKY68

MAVDLMGCYAPRRADDQLAIQEAATAGLRSLEMLVSSLSSSSQAAGAHKASPQQQPFGEIADQAVSKFRKVISILDRTGHARFRRGPVESSAPAAPVAAAPPPPPPPPAPVAAALAPTSSQPQTLTLDFTKPNLTMSAATSVTSTSFFSSVTAGEGSVSKGRSLLSSGKPPLSGHKRKPCAGGHSEATANGGRCHCSKRRKNRVKRTIRVPAISSKIADIPPDEYSWRKYGQKPIKGSPYPRGYYKCSTVRGCPARKHVERATDDPAMLVVTYEGEHRHTPGPLPAPPAAAAVAAMPVSVAVSTGNGHV

>OsWRKY69

MEGVVDGSGAQLVVAELVRVQGLLRQLEAHLSAPCSVELCRGLVAQIVALTDRSIGIATRSFSSASGGGAHFADTAPPMPALTSCTPSPLSDGSDHQPFRTTNAKKRKTTARWTSQVRVSAAGGAEGPADDGHSWRKYGQKDILGAKHPRGYYRCTHRNTQGCTATKQVQRTDDDASLFDVVYHGEHTCRPGAASAAAAKRPHAQTLLQSLSASLTVNTDTNTPLTPENRAPAPPLQQQQQQQSVSASPVASDSYGLGGAGYGDWRCCDGDLQEVVSALATVTSAPDHAAMDAADFMSYCFDFDPAVYGGIVGTPSFFL

>OsWRKY70

MTAAPGSLPLVNSRPVSLSLAASRSSFSSLLSGGAGSSLNLMTPPSSLPPSSPSSYFGGVSSSGFLDSPILLTPSLFPSPTTTGALFSWITTATATAAIAPESQVQGGVKDEQQQYSDFTFLPTASTAPATTMAGATATTSNSFMQDSMLMAPLGGDPYNGEQQQPWSYQEPTMDADTRPAEFTSSAAAGDVAGNGSYSQVAAPAAAGGFRQQSRRSSDDGYNWRKYGQKQMKGSENPRSYYKCTFPGCPTKKKVEQSPDGQVTEIVYKGAHSHPKPPQNGRGRGGSGYALHGGAASDAYSSADALSGTPVATPENSSASFGDDEAVNGVSSSLRVASSVGGGEDLDDDEPDSKRWRRDGGDGEGVSLVAGNRTVREPRVVVQTMSDIDILDDGYRWRKYGQKVVKGNPNPRSYYKCTTAGCPVRKHVERASNDLRAVITTYEGKHNHDVPAARGSAAAALYRATPPPQASNAGMMPTTAQPSSYLQGGGGVLPAGGYGASYGGAPTTTQPANGGGFAALSGRFDDDATGASYSYTSQQQQQPNDAVYYASRAKDEPRDDGIMSFFEQPLLF

>OsWRKY71

MDPWISTQPSLSLDLRVGLPATAAVAMVKPKVLVEEDFFHQQPLKKDPEVAALEAELKRMGAENRQLSEMLAAVAAKYEALQSQFSDMVTASANNGGGGGNNPSSTSEGGSVSPSRKRKSESLDDSPPPPPPPHPHAAPHHMHVMPGAAAAGYADQTECTSGEPCKRIREECKPKISKLYVHADPSDLSLVVKDGYQWRKYGQKVTKDNPCPRAYFRCSFAPACPVKKKVQRSAEDNTILVATYEGEHNHGQPPPPLQSAAQNSDGSGKSAGKPPHAPAAAPPAPVVPHRQHEPVVVNGEQQAAAASEMIRRNLAEQMAMTLTRDPSFKAALVTALSGRILELSPTKD

>OsWRKY72

MENFPILFATQPTSSSTSSSYHFMSSSSGSHDHRHHHGLQAGGNGGGGGGSLSHGLFMGSSSSSIRMEELSNSKQAGDVVVDGGATRSPHGGDGDGAAGDDGGDAQAAAAGGRKKGEKKERRPRFAFQTRSQVDILDDGYRWRKYGQKAVKNNKFPRSYYRCTHQGCNVKKQVQRLSRDETVVVTTYEGTHTHPIEKSNDNFEHILTQMHIYSGLTPSSAAHASSSSPLFPSAAAAASHMFQ

>OsWRKY73

MGEVREENERLKTLLSRISHDYRSLQTHFYDVLQQGRAKKLPDSPATDIEEPELVSLRLGTSTSKCKKEDKSTTSSEVKGSTEDFLKIKGGLSLGLSDCRVDANNSEKVQPDVMTLSPEGSFEDARDDTAETTEQWPPSKMLKNLRSVGAEAEDDIAPQPQVKKARVSVRARCDAPTMNDGCQWRKYGQKIAKGNPCPRAYYRCTVAAGCPVRKQVQRCADDMSILITTYEGTHNHPLSVSATAMASTTSAAASMLISGSSSTSLAAYPAAAASPALAFDASSKPPLIGGRPFFLPTAAAAAITSTPSYPTITLDLTSPAAAATSSHAAFSLSNRFSHTRYPSTGFTFSGSGPSSAPWPGYLSYGASLSAHPYNAGGGKSSSSFEAALSSINGSRQQGGGGGGGSAPPLYQMQQKAAAAAPPPPSVITDTIAKAITADPSFHTALAAAITSYVGKKGSPPASGGEDSKVGLKWGEHLGLGLTHSSLSTAAAAAASSSSQMFLQPSLGLSGSTTSASTSPVANREQAH

>OsWRKY74

MESMEGNGGGRLVVTELSHIKELVRQLEGHLGGSGSPDLCKHLASQIFSVTERSIGMIRSGHFDGHRKRSAAAVAAGDLDSATPSPLSDVSDLPFKATKKRKTSTEKKRHQIRVSSTGGVENPPVDDGHSWRKYGQKEILGAKHPRGYYRCTHRHSQGCMATKQVQRTDEDATVFDVIYHGEHTCVHKAVAAGAGKPETETDTNAAAESRLHDLSSGLTVKIEGLTAPPQQQQGGGGWNAMPPFCLSSPVSGLAPPDQHNPFSAPSTPENRLAAAASSAASPATSDSMAAAPFHQAAAGGGDEAWRDAELQEVVSALVAATTTTATAQPAPATAMVDADLSALDAFEFDPGFTIDITSFFA

>OsWRKY76

MDAAWRGGVGCSPVCLDLCVGLSPVREPSAARHELLDRPAGCRGGGDSKSMTNDEAKIVEAKVTQMSEENRRLTEVIARLYGGQIPRLGLDGSASPPRPVSPLSGKKRSRESMETANSCDANSNRHQGGDADHAESFAADDGTCRRIKVSRVCRRIDPSDTSLVVKDGYQWRKYGQKVTRDNPSPRAYFRCAFAPSCPVKKKVQRSAEDSSLLVATYEGEHNHPHPSPRAGELPAAAGGAGGSLPCSISINSSGPTITLDLTKNGGAVQVVEAAHPPPPPDLKEVCREVASPEFRTALVEQMASALTSDPKFTGALAAAILQKLPEF

>OsWRKY77

MSSLYPSLLSLSESPAEYRQVGGGRYAGEDVVDDDDDMAAVADAVSSYLSFDMDDVEYYTPEVGFHSKQHNPPPVAAAPLEAGGGREQSRREAAVNLGKMDRGPAPVSGGAATGGVPRSKNGSKIAFKTRSEVDVLDDGYRWRKYGKKMVKNSPNPRNYYRCSSEGCRVKKRVERARDDARFVVTTYDGVHNHPAPLHLRPQLPPPGGYSIAGAPAVVAPHGRLGLEEAEVIALFRGTTATSLLLP

>OsWRKY79

MAFSSEGGVPAERVAAAVNDLVEVRDGLVRLRGFLPPPPQAEQSSSRPPCAAELMDATMSKLMSAMATLGGSGDIAGEVDAAGRWTSVAESADPMVVRREGESSAGRTRRRRGGGSRSGRGRSSNKRVAATLEDGHVWRKYGQKDIQNSPYPRSYYRCTHKLDQGCGARRQTQRCEADPSNYDITYYGEHTCRDPSTIIPTAIANAAGAASDGPNNNIISFATGGVVVANSSRLAREGTTATTTSAATQLSSSWGTSGGGGGGDDVFSSSGERFMQWDELAAAVGHVSSVGVTSSTVGSAPAAENDGGNGDTAAGGGGDGGGAGSFPSSPSAGSLGFVVGPLGSIEDVDDFFPFDP

>OsWRKY80

MDMMEEEAANAATAQAAAAGDLADVVARANARAFLVSTPHHHPSPLHPLPPPPMPQAPHQYYPAPQITIPYHHHHHGELRRPTTIAYTDAPVPFETAGPPSTVVDSYHHLTPGDAGYGMPRPLALQISQHALCGGGDVVMGGGGAGAADDGEEAIRISPLTPSAHHQMMKRKNEVKKVVCIPAPPATSSRGGGGEVIPSDLWAWRKYGQKPIKGSPYPRGYYRCSSSKGCMARKQVERSRSDPNMLVITYAAEHNHPWPMQRNVLAGYARSHHSTHATASSSRHKQQQQQQTNQLQPALITSSSSSSSSPFNLYADVVLGGQQANMMMTTEGAGAGLGIQPSAADEVFAELEELEPDNPTMINANMQVYSTTSRPGVSSYDHQWHKF

>OsWRKY81

MAAHEGGGNGARRPPAPPLLPTLSLPPRSAAGSLFSAESSPGALTLAASLFPDAPSPAFQGSFTQLLVGAMGYPAASAPAPPSPFPVPHGLSPTAFLGGSPGLFSPTGNFEMSHQQALAQVTAEAVHSPYSMINQSDFSLPFSSTTTSVLASQHVNSSANVSSPREIPTLPSHTDNSNIESTEVSHGFQTTALTEDKPADDGYNWRKYGQKAVKGGEYPRSYYKCTHLSCPVKKKVERSSDGQITQILYRGQHNHQRPPKRRSKDGGALLNEADVSPEKEDASTRSEQGSQDYSGKFKASNDGGPSSSRRGDRGEQISGSSDSNDQGEEEVKVEGRATSDGNANKRHVPAPAQRIIVQTTSEVDLLDDGYRWRKYGQKVVKGNPHPRSYYKCTYQGCDVKKHIERSSQDPKAVITTYEGKHSHDVPAARNSSHSSANANVSSSSNLPHKDRGQRSSCRDGLRNASSVSSLQLKEESG

>OsWRKY82

MKNKGSSCRYLPHSSAPCATDGARCFRCDHRPAIDEIVREQSLVTQLRAVVLPALETKADDGRAEIVAQLFGSILDCSRKVISALNSRYVGESPPDDDEIVDKRRAKRKNSEGKKGDDQVKVKPHEHKRSRRYTNSTSQITAVPHYDGHQWRKYGQKNINNSNHQRSYYRCSYKHEQNCKATKTVQQLDSAGETIMYTVVYYGQHTCKTNMSNAPLHVVETSTTQSISTTCCSDDLGDYSQKMENMHTPELAEVCSDELGSYHAIIGAEHSALGLEDEHMHKLLDTFACGALDLDSWEIDAIVRSGFC

>OsWRKY83

MSPWRPGTPPRRAKHRLPPPPTTPISRGPRSMAVSGVGAGAARGLLFTSPSTAPAPPAPALSAPRSCHLRCAGQGREGCHQEQGSRWEAAVAVVQLLVLHRHRRRAPAVQQQRRGGRRGGGDEVVDALLLPQHLLRLHLRFLQQHRRRVKAPPQEPTSPRASPRAAERRQCRRREAARRQQGRREESRRQARRRRRRRGRGGQHGGGEAVAQPVRRLPELDGGDGGGAAHLWRRRHGRPPDVVPVAQLKAPPPGHPRRLRGRLGSRLRHALIDRSIDRSPSL

>OsWRKY84

MARRLPKSERSPSPPPPPPGDQRDAAIQELSKGSELATQLMAQLELIPERELDGRRDDALANVRSLSMSLSSSLYALRSERREHYYCGSSSSSGGAGPAAVTSVSGAGGERKTKRRRGKHGEELIETVFITTTPENDGFHWRKYGEKNILNSEFRKLYYRCGYSDERKCQAKKYVQQENNKHPPEFRVTLTNEHTCNTVFQDQPSSSSTNSQVLDFTKASISSSLMDSHVGAPILKEEEEEEVPSIDESTRIMSTIMRNYGSYGDYDESSPQPWNGAGWK

>OsWRKY87

MADSPNPSSGDHPAGVGGSPEKQPPVDRRVAALAAGAAGAGARYKAMSPARLPISREPCLTIPAGFSPSALLESPVLLTNFKVEPSPTTGTLSMAAIMNKSANPDILPSPRDKTSGSTHEDGGSRDFEFKPHLNSSSQSTASAINDPKKHETSMKNESLNTALSSDDMMIDNIPLCSRESTLAVNISSAPSQLVGMVGLTDSSPAEVGTSELHQMNSSGNAMQESQPESVAEKSAEDGYNWRKYGQKHVKGSENPRSYYKCTHPNCDVKKLLERSLDGQITEVVYKGRHNHPKPQPNRRLSAGAVPPIQGEERYDGVATTDDKSSNVLSILGNAVHTAGMIEPVPGSASDDDNDAGGGRPYPGDDAVEDDDLESKRRKMESAAIDAALMGKPNREPRVVVQTVSEVDILDDGYRWRKYGQKVVKGNPNPRSYYKCTNTGCPVRKHVERASHDPKSVITTYEGKHNHEVPASRNASHEMSTPPMKPVVHPINSNMQGLGGMMRACEPRTFPNQYSQAAESDTISLDLGVGISPNHSDATNQLQSSVSDQMQYQMQPMGSVYSNMGLPAMAMPTMAGNAASNIYGSREEKPSEGFTFKATPMDHSANLCYSTAGNLVMGP

>OsWRKY88

MAAARRVAGGGGGSLWGPPQPPPSTGGGIPQLPAAAAAPVEGLLDAPFSSSSGGGGGGWPPPPPPLSGTAVLIGYPQGNFETFPQQDLVPLTAQEVHSKCITFGRAENLPFIPLATSALVSQHTGSSSVNVTPLQEILTSPSQISNVNTESIGVLQGLPASSIVLDRPTDDGYNWRKYGQKAVKGGEYPKSYYKCTHLNCLVRKNVEHSADGRIVQIIYRGQHTHERPSKRRFKDCGGISDDLDDFSGTTGTSVRSQPDYDDYCRKPIIPSGTMVAPLVKKIEDGDDQLSGSSDNQDEHDDEVRTSDGASGDASANERNVPAPGQKIIVSTTSEIDLLDDGYRWRKYGQKVVKGNPYPRSYYKCTYLGCDVKKQVERSVEEPNAVITTYEGKHIHDVPAARNKSHVVANASLLQNTKSNTYCTEQSYTTITC

>OsWRKY89

MPDGYPAAPPGMERPAPEAAAAAAEPPGAKAEEEEEGPKDVGVKGTGKEKETEKVEAEKEMEGKGKGKESMEVEEGKEKEGKGKAKEKEKETKVKVKEEGGEEKEKGKVEVVEAKRRPAGVGAETPILAVPMVAVPCFLASPAFAGHFSMSHQAALASVTAQAQIQLQSPTTPYSEGLPSPFPITPKAVMPLQRSPSGTEGSVRRSVLEKSASFQSRPHNHVSVNMVGDGFNWRKYGQKQVKSSENSRSYYRCTNSNCLAKKKVEHCPDGRVVEIIYRGTHNHEPPQKTRFVKERVAHITASSGDDETLGLVNNEIIESPSPGCKLEPGAVSEASEQQLFCSSDCEGDAGNKSEDDHPSTEPQPKRSRIIETSTPLTPVLRTVREQKIIVQAGKTSDGYRWRKYGQKIVKGNPNPRSYYRCTHDGCPVRKHVEKAPDDDNNIVVTYEGKHNHDQPFRNNSESKDGPVPMIIPAETTSEQPSTMTSTSEQKQPISLLKDGGDEPMKGKTSEIGGEKAVESAQTLISIKTNPDDMKNTLLKDTSAVVPVQNN

>OsWRKY90

MASSSDHGSLMEDWMPPPTPSPRTLMSSFLNEDFVSGSFSNIFSDHESNKPQDQFERNRELVDLSKEVPSQFARPAFQRDASLDHSLVSPTQRSNSHGGLAERRAARAGFSVPKIDTSRGGSSTVIRSPVAIPPGLSPTTLLESPVFLYNAMAQPSPTTGTLPFLMASNAKSTIPSATKMDEDCTFGNDTFSFQPHVGSRRPNFSAAEKGPNACHQNQSLSNIHQRESSLQSSFTAVKDITDEKNIKTKTSDSMFGDNHSSDEQDDETNQNGENSMPPPNHRSGVPLSHTNDPEVNVLENRGSQTCHNSASLWDNAKNDCLQDVQSEVIETRTAACLPVSTNCDTSIMESQDAVDVSSTLSNEEDDRATHGTASIECNGDGDETDSKRRKLDALTAATAAITTTSNIDMGAAASRGVREPRVVVQTTSEVDILDDGYRWRKYGQKVVKGNPNPRSYYKCTHQGCSVRKHVERASHDLKSVITTYEGKHNHEVPAARNSGHGSSGSGNAPSAPQSNGSQRRQEQGQASFSQFGGAAPFSSFVLPPRNQFGPSASNFPFGMVPPGMAIPMPSLGSLAPAKMAGHPSTMQGYQGLMIPEGEMKTEPMSQLGFPAVNQSSSSFQQMMNRPPSFGPQM

>OsWRKY94

MEEEVEAANRAAVESCHRVLALLSQQQDPALLRSIASETGEACAKFRKVVSLLGNGGGGGGGGGGGGHARGRMAGRSRPSAVLREKGFLESSSGGGQLGMMMSGAATPSTSSAAHLRNRIGGGSGVPPDSLRGLDLVSSSSKGGAHQFDPPKLVQPLSVQFQFGATAHRYPFQQHQHQQKLQAEMFKRSNSGISLKFDSPSATGTMSSAFMSSLSMDGSVASLEGKPPFHLISGPVASDPVNAHHVPKRRCTGRGEDGSGKCATTGRCHCSKRRKLRIKRSIKVPAISNKIADIPPDEYSWRKYGQKPIKGSPHPRGYYKCSSVRGCPARKHVERCVDDPAMLIVTYEENAEAPSFCSGTVGPVM

>OsWRKY95

MARRRVSAGAASTPIHQIGIQGRTTKQALHGCSVVIKSSTSVVGPYAWVRSGGGRNKLSVLTIGKRKGDKHIMDNHNLEEEAKESVSKRRKNAEHTGSTVAQAPHNDGHQWRKYGQKWISRAKHSRSYYRCANSKVQGCPATKTVQQMDSSGNGTSKLFNVDYYGQHTCRGDGIANPYVVDTAHHSMEPINQNECNSPTLEHEAHEVQDERFENLCMVQNMPEYLIDFELERAFEFIVNSPLGSEHWTFDDSIRCEHSPICIWG

>OsWRKY96

MSARPPPPPRPRLALPPRSAAESLFTGAGDASPGPLTLASALFPSDPDGGGGGGGVMTSSSSSAAGATSFTQLLIGNLSAPPPPPPPPQQQQQREAARGGGVARAGPALSVAPPPTAGSVFTVPPGLSPSGLLDSPGLLFSPAMFDAFDVLDMFIPDKILPKRATRIKLDIYFVKTSPEGGFGMSHREALAQVTAQASHSPLRMFDHTEQPSFSAAPTSSEAMQHMNAAVNMTGISDMVMGPTNNENVAFQPAEASQRYQVNAPVDKPADDGYNWRKYGQKVVKGSDCPRSYYKCTHPNCPVKKKVEHAEDGQISEIIYKGKHNHQRPPNKRAKDGSSSAADQNEQSNDTVSGLSGIKRDQEAIYGMSEQLSGLSEGDDMDDGESRPHEADDKESDSKKRNIQISSQRTSAEAKIIVQTTSEVDLLDDGYRWRKYGQKVVKGNPHPRSYYKCTYAGCNVRKHIERASSDPKAVITTYEGKHNHEPPVGRGNNQNAGNAAPSSSAQQNMQNLSSNQASLTMADFNNINQRPIGVLQFKSEE

>OsWRKY97

MALDSVPSYPSDLGSSRARTPQQQRVSPRKEERTWTTDTYAPYDDGHQWRKYGEKKLSNSNFPRFYYRCTYKNDMKCPATKQVQQKDTNDPPLFSVTYFNHHTCNSSPKIVGSTPDSTVQSRKAISICFNSHGKTGELPTFLSPSASLLSPSMQPYSSNQQPDMNTYSRQFHWADTSSSTSYAPVKMEADDYAEASASPSTTGALSRTLLPIGQSRCIEYFHFL

>OsWRKY102

MALGHHGAARQPPTTMAAAASSSTTSAAAAPATATTTVAFSFQHPTPTPSCEEGDHRGRPQMGNKGEAAAAMGAMGINDAGNNTAAAAAAQHHLGVGAVRMKKVGGGGGGGGKARRKVREPRFCFKTMSDVDVLDDGYKWRKYGQKVVKNTQHPRSYYRCTQDNCRVKKRVERLAEDPRMVITTYEGRHVHSPSRDDDDAARASAEMTFICPLNRRNVPYGALLNSYGMPHFMIIVQIAFSTIYVVLVLLVAVTHVDSSLQGVYFGGNSSSAAAAAASQMSNQILIEFNQIYYFRFEFLKNHLNSSLMLTNFELARNQKTSSQISMGFPRNCISWLDTLLQPSPSTHASHLPPRGQLVASVDACGPSDADRIFPLRSTARETRSRGNINSSDLMDLSAIAIAHCISTKQPNHKVGASRERKKER

>OsWRKY104

MKILESFGHSDCQVVINMIEHQKALMVELRGMVMPLLPSDNEQAKLALQLLGDILSCSDKAISMLELGGDTKKLTNLVGGKRKGDKHSMDNHNLEEEAKESVSKRRKNAEHTGSTVAQAPHNDGHQWRKYGQKWISRAKHSRSYYRCANSKVQGCPATKTVQQMDSSGNGTSKLFNVDYYGQHTCRGDGIADPYVVDTAHHSMEPINQNECNSPTLEHEAHEVQDERFENLCMVQNMPEYLIDFELERAFEFIVNSPLGSEHWTFDDSIRCEHSPICIWG

>OsWRKY105

MENQSGQAQDGMADQRFRSWLTEQFNTQPYAGSSSNLAVMQMQVLPSHTTSQIIDTHPPEYDGYNWRICGQKVVQGGCHQKFYYECSQANCGAEKSVTRSADGQIKKTVCKGSHNHPLSSERVFGEGSATLDAIPVGEILQAAGVIRPSVAMPMNEEEDELQSGLGDSEEDDANEARVDGDGAAADANAIERHGAAQDITAQTATEVDVTGNGCQQRKNYCRSENRRSKSKVWKEFTAVLSVGKIQSAECKHCKKCLSGKSSGGTSHLRRHLKICPGQCRDTRIQQKWSSSRLDSSDANNWEFDQETSLELLTRALVSNLCPFSVTTSANFRKFFAGICPTYNIVPQAAIEEKFLSIFQNEKMKLKEEIALKPGGVFLSVTRWAPECKQFLCFTVHFIDKEWKLNRKIIRFQFSGDEALEAEHYVSILSNWKSFSNIRNASYEYGTEKTNKALIKAAVQDWNLEKKLLGIALPTNIGNEVILDLEETMTAAGQNFLLAKYKLLIVPCMINALHGLFGYTLERYVLEASREWFEYMTCSAIRLEKYKEILLRLHLSQPSFGSQKWHLTYYLFEAALQFVKEFPNPDAAHLKMFLRKPFPERLEATKNFCDLARPIYHAIDVLSRQNVAFNSHFHVIWSLGTVLKESSKKINIKRIIDIDDMLKKFDNLWRKCYVWLSLAVVLDPRFKLRYLEQCFKQAFGTGAKLCILEVRGKIYELFLQYSCNADQQSGELVNHWNNDLQMDRDGNDSLHGTDQNDIGQSALGEFRELTLYLEGGLCPQNEQFDILKWWKDNALTYPTLARLARDILAIPGSAVSAESAFDETDERVSLFNRKLSPEIVEALICTQDWIKSSETGDENGGS

>OsWRKY106

MENNQSGQAQDGMADQRFRSWLTEQLNMQQQNAGCSSNPAPAVMQMPALPSHTDYGGIYPNPTEYDGYNWRMCGQKLVQGGCHQKFYYECSQANCGAEKSVTRSADGQIKKTVCKGSHNHPRSSERVFGDGSATLDAILVGEILQAAGVIRPSVAMPRNEEEDGLQSGSSDSGDDDASEARAAGDDNAIRHVPAAAAQDTTAHNTIDVDVLGNSSQQLMGSFLTASQPLDSFLSSEKRKSKSKVWEDFTAVFSGGKVQSAECKHCKKCLSGKTSGGTSHLRRHLKICPAQFRTTRLQQEGSSSILDSSAANNRKFDQETSLELLIRGLVSNHCSYLVPSSANFRQFLVAICPDYNMVPQAAFEEKFLSFFHNEKMKLKEKIELTPGGVFLSVTKQYVEFKTFVCITVHFIDNEWKMNRKIISYGYGGYPDGADYYVGILTNWKSYLDIRDSLDYNFEEIDSSLIKEAVQDWNLEHKLLGLALHKNFRNNVTSDLEECMAGEVQNYLLAKYKLLTVPCMIDALHDFFGYDVGNFVKEISKEWFEYMTCSALCLEKYKEILSRMHLNKPSLGSQKWHLTFYLFEAALQFNKEFPNPEEMDSQMDIRKPSPQRLEATKNFCDLVRPIYHAIDLLSRQYVTSNSHFHALWRVGIALGESSRKLNMKCIINVDYMKKRFDILWRKCYVWLSLAVFLDPRFKLRYLEQCFTQVSSSGCAKLFVLEVRAKIYELFLQYSCNVDWQTGELLNHRSNDLQMDRHGNDSLHGTDKNDIEQGSNGEFRELTSYIEGELYPQNDQFDILKWWKDNASTYPTLARLARDILAIPGSAVSAEYAFNKTGERVILFNQKMSPEIVEALICTQDWIKSSETGDKNGGS

>OsWRKY107

MDVVVESPPVRDEKKVDVAAIGGAPPIVFESFAPSTQRDSTIIKKEEKMEAAKAEMGEVREENERLKTMLTRIVSDYKSLHTHFLDVVKVKEQTAAELSGDDDDDEPDDLVSLSLCTRPNAAATRRKGHERTPSSGGGGDDGRLSLGLSCARGGVASDDDDDKQASRRALPPMPVLNLSSDSSGDAAGAGAGEPTQPNKASRSSSGGGDGADDEVLQQQQAKKARVSVRVKCDTPTMNDGCQWRKYGQKISKGNPCPRAYYRCTVAPNCPVRKQVQRCADDMSILITTYEGTHSHPLPPAAAAMASTTSAAAAMLTSGSTNSTMHGSGGVHHHLPFASAVGGGGGVGLLGPTTISTATSCPTVTLDLTAPHSLLHPSSASPYAAAAAGYESSRALPAAWSSGYLAYGGAAAAQPYYAKGVAPSPFGHHFGMMGMAAAAARPAPEQLFGGQTTSPYLQRAIGGGGVAPAAVTDTIAKAITSDPSFQSVLAAAITSYMGRGGGAAAPNK

>OsWRKY108

MQAQSRLAAAASGGSGSGISGSGGISRLGGGAGEEHEAVVRELTRGHELTARLRAEALRALRGQGQAEATATFILGEVSRAFTVCLSIMASASPSASPPQPDETPPADSAVSPPPPRAAREDNVPRKRLLTASPYDDGYQWRKYGQKKINNTNFPRSYYRCSYHRERRCPAQKHVQQRDGDDVPALHVVVYTHEHTCLQGAPAELPDAATNGGAAAAASPDYFPAGGETPSSLRRLRGVGGGGLQPQFVDHRAAMEERERQVLVSSLARVLQGRQCYDDDDDDDTDVASLGAVHARAPAAAAPVAASSSSSGPVDAAGEELDVMDYDMTDALFWGPFGTDSNSYDGNLTSTRCFDLIN

>OsWRKY109

MTKMSSMKADGSVPKRRRQDVQKVVVSLKDHKVEQGPPADSWSWRKYGQKPIKGSPHPRYHHLALIPHTYTAPNVLVISQSFACALILYITDNFTRIRLSDQLLVRFFSLYQSIIFALFVDHAVSRGYYKCSSYRGCPARKQVDKCRNDASLLIITYTSDHNHDNYAATTTNSVQEQAHNPDTSDPLSNGMSLAEVVTVASSKLSGEEESCDFFDELEELPVSASPLPSLSFMVQECSFSDARTLL

>OsWRKY111

MTITHASSLSRFHPLLKKLIMEYSNDWDLQALVRSCGTAVADSEPEPPAAPSTTRRAEAETVFVGRAGGVPEFVGQPVRSSAASFYDLEYLDLYHERPRAPFLVTAPSTSRERGEGGEHEVLISFPAIASTSGQGRKQPGRKPGVRTARPKRSKKSQLKKVVCEVPVADGGVSTDLWAWRKYGQKPIKGSPYPRGYYKCSSLKACMARKMVERSPEKPGVLVITYIAEHCHAVPTQLNSLAGTTRNNKPASPDQQQQQQPSPGGASTDEAAAAAAKTEDSADTTCSMADDENDLWAPVEMDMNDFFGPFDDDLDHFLDDDAVLGRRLSL

>OsWRKY112

MSANIESFCCIDHRMIIQEMKREHGLLIDLKNHIIPILRFNNVQADHIVHAFDDILCCSNGIISKIQAEVCDGGNSDPGIDKGNGRNALDNMKVFIEDGTVTKNKRRKNAQHTGSVVTATPDYDGYEWRKYGQKSISKTKHSRSYYRCTNQKGQGCMATKTVQQIENDNSSNSVVKLYNVDYFGKHTCKFGNDMVCPDIVETDSPKYSSINDKYASTRLTNHSDDHQPKNDMKPENLFAVPDMSLFSENMWDIIFEDVTMNSTFSLEQEAKDSWIKHQQESTIHLWADELC

>OsWRKY113

MDGGDIHLLLSILADGEEQARQLGEPAAAADDEYHGGGRGEEYYRGVARQLQGTLARAMGIARAIEAAAFAGGGGGGGASGSRGTTGDRSDSPRSADESSGRTARDAAVAQQERHHDTIKRRKGLPRWTEKFRVPDASLEATPDDGFSWRKYGQKDILGAKFPRGYYRCTYRNAQGCPATKQVQRSDADLAVFDVTYQGAHTCHQKQRRAAAAGDQPPPPPPQADPSVELLVNFRHGLKVETNGLAPPPPPPPTTTTNFHDDQHFCFPSMPPFHAGVGPPPPPDDALGGGCNNFSSPPFVSPAGSAAGESYFSMEHSYEPRGGGGHFVMSRGDSSELHEVVSAAASSSAVVDPAAAAGGFDYPLYHGEVDPHLPFPPLFGHASMYGQYRDA

>OsWRKY114

MAVTESVCLSDEQQAVAVREVAQVYELIKTQQPLLLVHQQPQQLAHGLLNHAMRALNVALSVMNQPHASSSSSAAAAAGGHHFPVMTMIKAESTPANSPAADVSDNHVAGKARRSSPAKRRRINCEDKSSWVYHTVVPHEDGYQWRKYGEKKIQGTHFTRSYFRCTYRDDRGCQATKQIQQEDKNDPPMFQVTYSNEHTCTTTRLINNTNNNPAALHSLTANPNGHPDDDSDDTILTKMIKQEQQAAWLPSPPPDLTTISNNFDETPGLHVSQEVPPCSSNSSAISHYADEFDHHQMGQQLETTVMEEALGLGADLDDPYFYDPNLLLIYENLMNCY

>OsWRKY115

MTYLEFLQKNQWGQEASVGADGVQVDDVCAGSLGGHATAGVWPGRWREIGGRACAWTAAEGLENGGQESRADGSSRRIILELGDRDDSYPWRKYGQKDILGARFARSYYRCAQMLGCTARKQVQQSDDDPSRLEITYIGLHTCGGDRPSSPAPTNPADGPRCDAATSSHRLLPSALQQKLEEHVPAASDDMMMACTPSWLFIPSPACSQSELLSEGEVPELRVVRQEPYDPVELVEEHKKPSDADEDSLALHNSVVPDFM

>OsWRKY116

MASPRLKREQSFDFEEASAQEAVGSASASYSPPGGGGVFGISPPESSPRDGRKRRKDRPSLVKHTFTPHFDGHLWRKYGQKNIKDSAFPRLYYRCSYREDRQCLASKLVQQENDDDPPLYRVTYTYEHTCNTTPVPTPDVVAEQPPPGAAGDAYLLRFGSSAGGGGGGAHQQQTERERQQQNTARRRPFMMLSFDSSSSHQLHEQPHAFPPDGQLPATAAAASPSSFTAAEALAAPPLTTTMNDGGDLFSTWDALRYGLDYDHGHLGNHVYLPDDCNGGDDNY

>OsWRKY117

MADQGFRPFSALMSAPSTAQQHVGSSFSTAVVQVAAAQQSHTDHGNICLADDDGYYWRMTGQSTTQGESSPTILSHYQCAQANCVVQKTVAYTADVETFYRGRHNHLRQSDRLEPMSQVGVLVEASDAAGAAAGPSVPETENGDDQSSGSSDRNEDDAGDVEMDEDAAAGDPNAMQRRKLKSKVWKEFKLVFKDGKLHTAICNHCKLRLVAETRNGTSHLRRHLKICPEKAGTSRVQKKRRSSTSQSQPDLPVSENLENGQENPSQNLENGQENPLEEFMRATVLKLCPFPAMYRASFASFLAGRNPAPNMVPQTTVEDKFISVYEKEKLKLKEKIIATPGGVFLSVNKWYSGSYETGIVCLTVHFIDEEWKINRKTIRCCLSESDGLDLNLFPHWQSEIANYEDDDKMVLKKVVRDWCLEPKLLGVTLEGSVDKKATISLEDDLTTGKNYLVAKCKLLTIPCMVDGLDDLMQYTVGREVRSMWSRYMTNTPERKLKCQEVVSQLQLDRPSFGSKYRYLTFYWCEAALQFIKSFPLSNGSERPSLDDLEATENFCKIARAIYHATKAFYEPYNLTFNSYFHVIWSLRATLQELPRIKNIERVIKVKNMQEKFDNHWKKWYLWLSIAVVLDPRYKLAFIELRFREAFSQDAGTYISEVRAKFYELYIQYSHVNEQSNEILNQGNNGSGTQISAPLHKQRTNYTIAQAALEEFKELFEYLGGGLCPQNDSFDILKWWKDNSAAYPSLAKMARDILAIPGCAVSAESAFNDDSDHRAELFNGKLGPETTEALICAQSWIIKSSGTADADNGNNITLS

>OsWRKY118

MENQSGQPQYAMADQGFHPFSPFMLAPSTTMQQHVGSSSSTPVIQVAALPSHAYYGNIDVADDGFHWRMCGQSTIQGGLCPTVFSYQCALPNCGVRKSITRSADGQTIETVCKGCHNHPRQSLRWLGDGSERLEPISQEIVLLEASDASGAAGGPSVPGTGNGHGQSSGSSDSCRDDDGDLGIDGNASVGDANAVKSGQVPAPAKEITVHSACEVDILNNSVRHENPQPRKKVRSKSTVWEEFEVVLIDGKVQTAECKHCKKGLSAKTSGGTSHLIRHLKICPAQHGTSRVQKKCSSLADLPIVKSWKDDQESSLDEIIRSIVSNLCPFSAMYSASFAQFLAGRNPVLNMVQQATVEEKFLSVFHNEKMKLKEKITATPGGVFLSLGEWQRLFYIQVRVACLTVHFIDEDWKINRKTIRCSLSVFGKSDILSLYPHWQSDIVLAEKVLKEVVQDWGLLDKLLGVTLQRSVDKKAPLHLEDDITGRNYLLSKCRLLSIPCMVDALHELMDSTVLDMESTWSHYMTSSPERKQKYQEILSQLHLDRPSLGSKGWYFTFYFSEAALQFIKSFPLPDAKPNCQSGPWEPSFDDLEATENYCKIARSAYRVIKVVSGPHNMTFNSYFHVIWSLRAAIQELPSIKNIGRVFDVAYMQKKFDRNWKKWYLWLSIAVVLDPRYKLGFIELCFRQAFSHVAGMYFSEVRAKLHELYIQYSYVNEQSKEILDHKNNCSDIQISAPLHNKGQNSTTAQAAVEEFKELYEYLGGGLCTQDDSFDILKWWRGNSSAYPTLAMMARDILAIPGCAVSTESAFDQCDQRAELFDGKLRPETTEALICAQSWIKSSGTADADDGNKNTSF

>OsWRKY119

MEEDDGLALMTGANQSLWSYYKCTSSRCSAKKHVEKSTDDPEMLIVTYEGSHHHGPQPLFPPHIAQPPPPTSVVGFSAASGAGPPPSSPAAAARKRKNYVRAAFSPTTSEDDGDGAGRLRPEWPQDDGTSCDVAELRRRGDAEHAAPRRVATDRSCDDGGGGGSTSASSSVARADAATALSSDSPPTIWSCLDWPWSQETLFL

>OsWRKY121

MEGMEEANREAVQSCHRVLTLLSSPHSQLVPNKDLAAATGEAVAKFCSVASRLNNGNGLQGHARVRKIKKPLPIFDSNLFLESPALAVAMAAKTPNSSPITSLQLFPRYHQMEGSSSKDPVRIPAQFPKRLLLDNPAVDSDGPSRGPPLQLIQPVSVAPPAGTPHPALPSAHLHFIQQHQSYQRFQLMQQMKMQSEMIKRSGLGEQGGSNGGGKGVNLKFDSSNCTASSSRSFLSSLSMEGSIASLDGSRSSRPFQLVSGSQTSSTPELGLMQRRRCTGREDGSGRCTTGSRCHCAKKRKLRIRRSIKVPAISNKVADIPADEFSWRKYGQKPIKGSPHPRGYYKCSSVRGCPARKHVERCVDDPSMLIVTYEGDHNHNRVLAAQPA

>OsWRKY123

MEPGSSSREGEQQEPANERLTATGGGGGGGGGSPVSDQELSDGEEVSDGEYQAGDDFSGYAVRGRGFVEKEHIFDKVVTPSDVGNLGRLVIPWQHAECYFPRDVPANEREGVVLRFEDDAGNSWRFLYRGSSLTLGWSHFFRKNRLDAGDMVSFYRGASEATRDRLFIHSKRRMHILPTLGYSDPQVHINRLFQLLVRVRTMVSSFGKSGSPSPPNSPLPTVQNFTYKALKDCLQDLHYYSLLSMMGRNHKSQSTSAKIRDTIPTLDALIQLRSLSDPVFRIPAAATSHCILLCRGVLGIMGFLCSDENMKEDHRMLQVPAFDDLNYSAQDKITRMKEQTMPSSLADPIYLLPTAIRNLLYLDLSNCSDIVQLPPSLGSSLHMLSALNLSCCYSLRALPDSLVCLYDLQILLLSFCHNLQNLPVSFGDLSNLRLLDLSGCRSLRLFPSSFVNLGSLENLNLSDCIRLMGIPQNFEDLQKLEYLNFAGCYRVDLPVYCLTNLVNLKCLTLSNHTDIKDFPYSFTDLKRHLYLSRWWKYNWVHTQCNLKSYRCHQQRIINSLLSDGSDEGDITSEQSLTSICIFGERGTGKTELLHEIYNDQKILEGFHLRIWINMCDKKRLLEKIIEFTACAYCYDAPSSILEETVREELNGKRFLLVLNDADIENQCFWTDVWKVSNVGAAGSALIVTTRSKEVASLFGAMKPYYMNPLSKEECFMVFQEHADCGFDINNDHELTKVGWKIVEKCGGNLLCMKALSGLLWHSKTALSEIDSLVGGIVPALRLCYDLLPSHLKQCFKFCSLFPKDYVFVKHHIIQLWISQGFVYPEEDSQPEDTGLQYFNEFLCRSFFQHCPFSNDHEDKFVMHELFHDLARSVSKDESFSSEEPFFSLPENICHLSLVISDSNTVVLTKEHRHLQSLMVVRRSATEYSSSFVPLLKILGLNDLLMKCGFLRALNLSCTTIVDLPGSIGRMKHLRFLAMNNTKIKSLPTEIGQLNTLQTLELKDCCCLIELPESTKNLMKLRHLDVQKEPGNIHVGMPSGLGQLTDLQTLTVFNIGDDLSHCSIRDLKNLSGLRGHVHITGLQNITAGDDAKEANLVGKQFLQALTLEWCCSSEEMEDESDKEIANQVLQNLQPNTSIQELAIQNYPGNSFPNWIKDSGLCMLVSITIDNSQDCNEIPYLGDLPCLKFLFIQKMYAVENFGQRSNSLTTDGKHAPGFPSLEILNLWEMYSLQFWNGTRYGDFPQLRGLSISRCPKLSNLPPLISLLYLSFHCGDQLPALSEFPSLKSLKIEGFQKLKSVSFCPEMPLLQKLEISDCKELVSIDAPLLSVSNLKVVRCPKLHFGGSWLEGCLMWEEFKR

>OsWRKY125

MEGSMFNLPGRLDRLLRRHGSMLPKGAEEEIPLIKQDLEEIISVLHGHCSKPKLEDHAMVVRCWMKEVRELSYDIEDCIDQYEHAATATRSHTGPNICRRKFNQRHGKMIPWVPWKLKQRLWMANKIREFSLRTQEALQRHTMYNNLGGITIASTTGGDVCSATPWHPTQFREHTDNVCSVGIDADGMEAALNDLNKLKNLLASIPTASLVQFREHANKVRHIHPDVEAILNKLKNIPTGITTTSTTTRGDVSSTSSRQPTRFMESAGLVGINAAVNKLENLLDVCGEEKLKVVSIVGVGGVGKTTLANKLYCKLQRQFECWAFVQTSQKTDMRRLLINILSQVQPHQSPDNWKVHSLISSIRTHLQDKRYLIIIDGLWATSTWDVIKCALPDGNSSSRILTTTEIEDLALQSCSYDLKFIFKMKPFGEGDSRKLFFSIVFGSHSKCPPEVSETLYDIVRKCGGLPLAIVTVASLLASQLEKQEQLDYINKSLGYGLMANPTLEGMKQLLNICYNNLPQHLKVCMLYLSMYQEDHIIWKDDLVSQWIAEGFICATEGHDKEEISRAYFDELVGRKIIQPVHIDDSGEVLSCVVHHMVLNFVTYKSIEENFIIAIDHSQATIRFADKVRRLSIHFSNVEDATPPTSMRLSQVRTVAFFGVLKYMPFVMEFRLIKVLVLHILGDEDSIGIFDLTKISELVRLRYLKVTSNVTVKLPTQMQGLPYLETLKIDGTISEVPTDIYLPRLLHLTLPAKTNLPSGIVHMTSLRTIGYFDLSCNSAENLWSLGELSNLRDLQLTYSEIHSDNLKDNMKYLGSILGKLRNLTSITLSPPGSSCPDTLHIDRTRINVDGWSSVSSPPALLQRFELLPCVCIFSNLPNWIGQLGNLCILKIGIREVTSNSIDVLGVLPKLTVLSLYVHTKPAERIVFDNAGFSILKYFEFICSVAWMKFEMGAMPSLRKLKLGFDVHIADQHDIIPVGIEHLSGLEEISAKIRVACTAHDHCRRFAESALTNAFMMHPGRPSVNIRCVDWTFHDKDNDCVGTREEECRTPMKQEHFVKEDLSEKSAVLQNEHDEEAHKFVDRRYYPIMDAAEIRRCPWSVNEEQEQPVLIYDARTKISQSSSMHSEFWAAVQRLTGPAATPAKTKRHLHLTTSPELEDGFLPVRSLVFPSAPDPRCNMKKKKMRAGPGGGRAVRSNWAPKS

Sweetpotato WRKYs

>IbSPF1

MAASSGTIDAPTASSSFSFSTASSFMSSFTDLLASDAYSGGSVSRGLGDRIAERTGSGVPKFKSLPPPSLPLSSPAVSPSSYFAFPPGLSPSELLDSPVLLSSSNILPSPTTGTFPAQTFNWKNDSNASQEDVKQEEKGYPDFSFQTNSASMTLNYEDSKRKDELNSLQSLPPVTTSTQMSSQNNGGSYSEYNNQCCPPSQTLREQRRSDDGYNWRKYGQKQVKGSENPRSYYKCTHPNCPTKKKVERALDGQITEIVYKGAHNHPKPQSTRRSSSSTASSASTLAAQSYNAPASDVPDQSYWSNGNGQMDSVATPENSSISVGDDEFEQSSQKRESGGDEFDEDEPDAKRWKVENESEGVSAQGSRTVREPRVVVQTTSDIDILDDGYRWRKYGQKVVKGNPNPRSYYKCTSQGCPVRKHVERASHDIRSVITTYEGKHNHDVPAARGSGSHGLNRGANPNNNAAMAMAIRPSTMSLQSNYPIPIPSTRPMQQGEGQAPYEMLQGSGGFGYSGFGNPMNAYANQIQDNAFSRAKEEPRDDLFLDTLLA

>IbWRKY1

MAVELLSSYRNSGFAAKMEENAVQEAAAAGLQSVEKLIRLLSQSQSQSSGFSSGSLPPPPATAAGEGSADYQAVADVAVSKFKKFISLLDRTRTGHARFRRGPICNPPHAPQPQRKMDQESEPVASGQTRVVENSENPHTGASKMYSPPPIQRLPPLPHNHHHMLKNVPAPPAPDRKESSTTINFSASQATSSPGSFISSLTGDTESLQPSLSSGFQITNLSQVSSAGRPPLSTSSFKRKCNSMDDSSLKCSSAGGSASGRCHCPKKRKSRVKRVVRIPAISMKMADIPPDDFSWRKYGQKPIKGSPHPRGYYKCSSVRGCPARKHVERALDDPTMLTVTYEGEHNHSQSIGDTPASLILESS

>IbWRKY2

MGGFDDHVAIMGDWMPPSPSPRAFFSSILGDDLGTRSAAEPTRENKTGTLVSEPEGYASSRNSDGKGGDQAGSMSSLSEQKIGSRGGGLLERMAARAGFNAPRLNTESIRTAADLSQNQEVRSPYLTIPPGLSPTTLLDSPVFLSNSLAQPSPTTGKFPFPSSGDIQNSTMFMEASHKSKESSFDNNDSSSFAFQPVFQTGPSIFPGTFGKVPPSNLARQSFPSVEPTQVYAQNGTIQRPDFSRSSTEKDNGSGNITSEHSPPPDELPDEETDQRGSGDPNFLGAPADDGYNWRKYGQKQVKGSEYPRSYYKCTHLNCPVKKKVERSQEGHITEIIYKGAHNHPKPTPNRRSALGSANPLGDMQIENTEQAGTGGDGDPIWANMQKGSGAGDPDWRNDNLEVTTSAPLGSEYCNGSSLQAQNVTQFESGDAVDRSCTFSNDEDEDDRGTHGSVSLGYDGEGDESESKRRKIETYAADMSGATRAIREPRVVVQTTSEVDILDDGYRWRKYGQKVVKGNPNPRSYYKCTSAGCTVRKHVERASHDLKSVITTYEGKHNHDVPAARNSSHVNSGVSNSGPSQATVPNQTHLHRPEPSQLQNTMARFEGPLSLGSFGLAGRPPQLGGTPGFGFGMNPQGLTNLAMAGLGTNPGKLPVLPVHPYLGQQRPVNHMSFMMSKGEPKMEPVSDPGLNISNSSSVYQQVMSRLPLGPHM

>IbWRKY3

MAENHAGRASKSSSLRLASARPSITLPPPSPIFTGGVSPGPMTLVSSLFGENDQDSECRSFSQLLAGAMDSPGARPARRVDPNSKEEEVFRHNGSAITQPSIFTIPLGLSPASLLDSPGQGHFGISHQQALAQLTAQPYIQSDHPSSSLPPAPQFFQLQMPLPGRDHNSIKESSSNVSHSDNVSEPCSFPVDKPADDGYNWRKYGQKPIKGGEFPRSYYKCTHPNCPVKKKVERSLDGQITEIIYKGKHNHQPPQPRKGAKDTGNQNGLQGSSELNLMDGVPFQSLPMKDQESSLATQEHMSGSSESEKVGDAEARMDRRDDDDERESKRRATEGQIPEPVTSHRTVTEPKIVVQTTSEVDLLEDGYRWRKYGQKVVKGNPYPRSYYKCTTAGCNVRKHVERLASDPKAVITTYEGKHNHDVPAARNSSHNTANNGPALQLRQHNPAAVDNQAALLQFKEEQIT

>IbWRKY4

MEDENFVFNLVIDDDNSPDEMKSSIGESFPSTSANETHSSSPMQIIPFDFPMESLDCGVDMSSAVDTNPNSNSNLMLQSPVNGECINNNVNGETCSPFNNPEPELDHRRSAAAAPAAKSLANNNNNSAGSSSDDGYTWRKYGQKHVKGSEYPRSYYKCTHPKCTMKKKVERSPDGQITEIVYKGAHNHPKAPATSLRRSPPSLGAESSSSEMMSQGSGSCFRSQAPIWANIHHYGSMPERSALASSSDLTAEICDPLSSLTTRSAAAMSGFESATTPEPSSTLASQDCDDNEDAVTQGISPCQFGEDGESEPKRRRKDGWSIEANLSTRSIREPRVVLQIESEIDILDDGYRWRKYGQKVVKGNPNPRSYYKCTSPGCPVRKHVERASDDLKSVITTYEGKHNHEVPPNKAAVVNYNSYSAASGTTASSAMPRAPALGGVGVQDHHPSFPFERKPMIAGAAGGDELLRPEMLDCYAAGDFRFVPSSIYPLKFPPPPLQGPLTAAAATFNYSRPPGMVLPEFPMPLLPMSLPPFHELTNLPPLADFLHFNDPSTKEEHKENDPHTSLLYE

>IbWRKY5

MGTPMEEGVDEGSLGKLQPKREPNTGSLESETEHKVSDKLVPAEGVSGELQKRLSPNVKAEASESSESKVAVPDKCETVPSDMQRKQGVVNGSSASQSEKEKSSHSGVQVKDGEEVQQKQGVGSDTDASQFSQVSIVPKKESDGTGHEQSNDEKIHGTETLALAVIPEKNSDNPQQLQIQSMEVLATHSNPARVTYVKPHEKGLDKLQPRRNPEIGAHTPQFDQRSPPSKAPEKPSEDGYNWRKYGQKLVRGNEFIRSYYKCTHTNCTAKRQVERSQDGHITEINYIGNHEHPKPQNSPQINAPTILPIQMRRPDLPIMTPSEGTQSVTLGEKCETPEPKQITSPVGVVSADIGARDSVLQSHNLRDEDDHCGGPDSKKQKKCLSSPDDNKPHGEPRHVVQTMSEVDIVNDGYRWRKYGQKLVKGNPNPRSYYRCSNAGCPVKKHVERASHDPKVVITTYEGQHDHDMPASRTITQNSGEGDATSGESRPESGENKHVGLDMVVHIGAN

>IbWRKY6

MAHSSPENSSANRKRAVDGLILGRNLTCQLREVLKNSSDEHGAPSKVVAEDLVAKILESFNEGISVIGSMDSDEVSQPPSDGRKSEDSSGSCKTTSALKDGRGCYKRRKTCETLIKDSQTLVDDGYAWRKYGQKVILNTPYPRNYYRCTHKFDQKCQATKQVQMIRENPALYRTTYNGNHTCLNFQKYPQIIVDSTAHGDSSFLLCFGQNGQSNKEVHNPTLIKQELNKQEFAENLYHSHIQSSSSGCCLPSSDDRPMSSARWGPAASSGSEYGDVNSSGCTHDDLGMQMMGNVDVDDFTLGFLADF

>IbWRKY7

MAVELMSGFRSDLGGKMEENAVQEAATAGLQSVEKLIRLISQSQQQNSGFSAAPPLPAFSSADYQVEADAAVTKFKKFISLLDRSRTGHARFRRGPVVNQKREVDPPPVNQNSSSRIRVSEEQPEKPEKKIYHPKPIQCLPPLPHHHHQAAKTAAAAANTIDRKEPSTTISFAAMAAPSPAGSFISSLTGDTDSVQPSLSSGFQITNLSQVSSAGKPPLSTSSFKRKSSSIDDAAVKCHSAGGSASGRCHCPKKRKTRVKRVVRVPAISLKMADIPPDDYSWRKYGQKPIKGSPHPRGYYKCSSIRGCPARKHVERALDDPTMLIVTYEGDHNHSRSITEAPSALILESS

>IbWRKY8

MAEELRDFYYHQPFQDDRHGGGFLYSAAQASSMADSSLLHHHHNLDPTSPYISFTDYLQGSSDFEASAGLGFSSSPSFSSAKDGERRSVNVTTTDAAVGGGSSETPVVMTPNSSISSSSTEAAGGDNDDSKHKREKLAKETEGEDDGEDNNSSKKENKGKKKGEKKQRQPRFAFMTKSEVDHLEDGYRWRKYGQKAVKNSPYPRSYYRCTSQKCPVKKRVERSYQDPSVVITTYEGQHDHHIPTNLRGSLAGMLPPSMLATSSLLGGPPPQGVTLPPELLMAQINPLAHHFYGGGHNAASMMFQPHQNNLTQMQQLHPDFGLLQDMVPSMIFKQEP

>IbWRKY9

MNTCSNNNNNESPHDHMDIDLSLKLNESPQDSPQPVGESSSQPNKGDSPSSSKNSKTEEITVLQTEMKRMKEENKVLREAVEHTMKDFLDLQTKLALFVQQNDHKKDVISNFLWVNGEEEKISQELNRTSSSSSPTAQLEHDKNNNNNSISDTELGLSLTLQTAAAADDHEKGRDQKSPPSPPPSIHGNIHHGSSFAAGLSAKNPPISQHNVRKPRVSVRARCESATMNDGCQWRKYGQKIAKGNPCPRAYYRCTVAPGCPVRKQVQRCIEDMSILITTYEGTHNHPLPVGATAMAASTASTAASFMFLDSSNPISNNNLGIPQNQPFLNSQNYHHMIPTLTRNLNPNSAMATPYHHDPAASSKLGLVLDLTKDGVSSSASSSTSLPKLQGQMGQYSSWMMQRLAGNFDGHLQPGNTAENDKQKLVENVSAIAADPKFRVAVAAAISSFINKDKPPS

>IbWRKY10

MGESSREGGASSAVSGPSRPTITIPPRAPFDTTLFSLGSIPGFSPGPMTLMSTFFSDSDACSFSQLLAGAMASPLANPALLPDKDGDSGPGCEKLSGYKQKQPMSLLVAQSPLFMVSPRFSPSGLLNSPGFLSPLQSPFGMSHQQALAHVTAQAAFNQSYKQMQVEYQHSSSVEGAGHQTSSSMPNQAVQGEAANVALDTESLKVETSELSQVDSKVSSGAIEKPASDGYNWRKYGQKLVKGSECPRSYYRCTHLNCAVRKKVERSPDGHIIEITYKGKHNHEVPKSNKRKQDDCDQESREDNSREKPQSASHRWTETNRSNKVVCSQPEMVSTKLQSEQLTVASKRDEMEETATVLDEKDDGERNAERRISEAGSSVLPSSHKTVMEPKIIVQTRSEVDLLDDGYKWRKYGQKVVKGNANPRSYYRCTYKGCNVRKHVERASTDPKAVITAYEGKHNHDTPNAPNNNHTETKNSSIQQLKQQKLVAA

>IbWRKY11

MAVELMMGYGNDGFAVKIEESAVQEAATAGLKSVENLIRLLSNSPECGGGFSASVSETPAAEAEFQAAALANAAVTKFKKFISLLDRTRTGHARFRRGPVGNQAGLDGKKIDQEPEPEPAASAADASEKPLSGSKIYCPTPIQRLPPLPHHHFVKNGSAANDRKESSTTISFSGAAAAAAAASPAGSFISSLTGDTDSLQPSLSSGFQITNLSQVSSAGGRPPLSTSSLKRKCNSMDDVTLKCGSAGGSASGRCHCPKKRKSKVKRVVRVPAISLKMADIPPDEYSWRKYGQKPIKGSPHPRGYYKCSSVRGCPARKHVERALDDPTMLIVTYEGEHHHSHSITDPPAAMILESS

>IbWRKY12

MEGDEGRSGLPNYGLQVSFSTTPHHHHPHHHHPHHHHPHAAMHHEMGFVHFEDHNQAVMSFLTPLSSSSQPLDGGAASSCSNAASTTPAAAKSSSHAATASLGFSHSEPPQLSNRPSWNNNDQVGTMDPKGANDENCSGNAAEGNNSWWRSSSSSSLVDKGKVKVRRKLREPRFCFQTRSDVDVLDDGYKWRKYGQKVVKNSLHPRSYYRCTHSNCRVKKRVERLSEDCRMVITTYEGRHNHSPCDDSNSSEHDCFTSF

>IbWRKY13

MSSSSTTSQAMLNQILFQDHQDVASSSSSLFCVSSNNNNITGTGTGTALPFESLKTLITVPMPTSLASLLPPLVESSSPNSTSAFHQTQTLLQQHQDLSPLFGPPHHHQLLSLHRSAPNLWAWGEVNECMMRSKRSGLVVDDHRHHHLGGLGVSAVKMKKMSKSRRKVREPRFSFKTMSDVDVLDDGYKWRKYGQKVVKNTQHPRSYYRCTQDNCRVKKRVERLAEDPRMVITTYEGRHVHSPSHDDDDSQASSQANDLLW

>IbWRKY14

MVGRFDQTHPSPPEQDDSDTSPENSADSPVSGADHDTKITKVSFPKRRKSAQKRVTSVTINDGEISIPPADSWAWRKYGQKPIKGSPHPRGYYRCSSSKVCPARKQVEKSRADPNVLLVTYFCEHNHSRPAARNAAVMAPDAAVSDESKRQTATSDHSSQSEGDSEERVAAKCHESPLAAGNDADWLLNFEPATSFAILDESPMMTQTKVADADDATPAVFPDRVEDESLFADLGELPEFSRGFRRGWGER

>IbWRKY15

MGSCLEHNSPSMDIDLNADVNEIPMRDGEHDTMLEEELKETRAENKRLSAALSAMCENYGSLQSQLLDLMQERSWKRMKSDRDTTTCGSSEIGYDEASVSKRPREIRTNISRVHVQTDPSDTSLIVKDGYQWRKYGQKVTRDNPSPRAYYKCSFAPSCLVKKKVQRSVEDKSILIAIYEGEHNHPHPSETTQTFTLASQTSNLGLGFHNNIQQRSLCDSTIMDTKEIQQLLVEKMASSLTNDHSFTEALAAAISERILDNPLD

>IbWRKY16

MDNTKSPASELKNSRTLRQASSSKKRSMAKKVVVRVKLGEGDGRKRKSEGPPSDSWSWRKYGQKPIKGSPHPRGYYRCSTSKGCSAKKQVERSKTDASVLIITYTSTHNHPGPPEDNPTTPVPEEEGEEAQMEQHRTRLKDNEEDSFHYSQSPFNTSQDIIINNYLFTQTLGTSSSLLLDGEEPLSYPHLMDFNSPPPNHKKENYDFYDELEELPHTSSFITHSTLSSRTQSL

>IbWRKY17

MGYYSSSLGDKSLAIDLNTSINMDCEAADTCGNSNSSVGDQKHGLLMEELKVTKMENKKLTAKLTEVCENYCTLQNHFLGLLKTHGADDDLLGKRKSADDDGDCYGAAAASSPKRPRETRTPVSRVRVKTDPSDMSLVVKDGYQWRKYGQKVTRDNPSPRAYYKCSFAPSCPVKKKVQRSVEDPSILIAIYEGEHNHPHPTQPEVLSVPLPQGFTPQSICSPVSDVDNSSSPARLDIRAKLQRSLTSIDSVELQHFLAEKMASSLTKNRSFTDALAAAISDRILLDHALADSCS

>IbWRKY18

MEFTSLVDTSLGLNAKPIRVVSGKPKQEVESNFIGLRMNIGNKDEAGELMEELNRVSAENRKLSEMLTVVCGNYNALREQVREYMNKQQQQSGSINDHNSSQVIMGSRKRKSPSNNNNNNANSESSSSDEDSAKKPRRELEHQHHIKANTSKIYVKTEASDTSLIVKDGYQWRKYGQKVTRDNPCPRAYFRCSFAPTCPVKKKVQRSVEDQSILVATYEGEHNHDLPSKLEQPSATATATAAARSLPPAAALNAQPRDLSPPKTALSVPNANGAKTASTPAGSSLPVDRPDFQQFFIEQMASSLTKDPTFKAAIAAAISGKFSPHNNNREKW

>IbWRKY19

MAKNDGGSSSSSAASRSAPERPTITLPPRSSVESLFTGGFMSGISPGPMTLVSNFFSEGDSYSECPSFSQLLAGAMASPAAFGGVRPPPPPPQPVEAKEESGGAGSGGDSDFRLKHNRPAGLAITQQSMFTIPPGLSPTTLLDSPGFSALFSPGQGAFGMSHQQALAQVTTQAAQAQVQMHIQPDYSSSSAAPATSYSQLQTIASNATINQQVPSQASDHNIMKETSEVSHSDQRIEPASFPVDKPADDGYNWRKYGQKHVKGSEFPRSYYKCTHPKCPVKKKVERSLEGQITEIIYKGQHNHPPPQNRKGAKDPGNSNGPYALQGGSELSSEGLTTNFNKPKDQESSQATHEHASGSSESEEVGDAETRADGGDDDERESKRRAIEMQVPDPATSHRTVTEPRIIVQTTSEVDLLDDGYRWRKYGQKVVKGNPYPRSYYKCTSPGCNVRKHIERAANDPKAVITTYEGKHNHDVPAARNSSHNTSNNNTGPQLRPHNVAGQQQAALRTDYSSNEQQVALLRFKEEQIT

>IbWRKY20

MEDSHSHSLYSNSHSQQQLAAPRADADAHAPDRVDDGCSDASDAAVFSRSAAGPNGGAKYKLMSPAKLPISRSACITIPPGLSPTSFLESPVLLSNVKAEPSPTTGSFSKLQTMQGFGSTGGFSFPRSNIYIESKSNNFEFEFPVGSYSTSESSSIGPKQNEPLKQAQDQCLPQSLAPSSLLESSIIPTSKELRISAPVAVHTSSVSTAPIESDELNQRGQSNPGIQTLNGDQPAAGAERSSEDGYNWRKYGQKLVKGSEFPRSYYKCTYPNCEVKKIFERSPDGQITEIVYKGSHDHPKPQPSRRFTPGSLTSIQEDKFEKESSFNVKEDKLNSNDFNGYPVLSPRRVDDDGHEGAASQLLVTNDDTDDDDPFSKRRKLDGCVDITPVVKPIREPRVVVQTVSEVDILDDGYRWRKYGQKVVRGNPNPRSYYKCTNAGCSVRKHVERASHDPKAVITTYEGKHNHDVPTARTSANHETGATPLSGASRVRPEENDAISLDLGVGIGYGTEHRPNGQLHSLAPETLQGQVHVSSSGMMTVQPPPMVCYGTIHGGINRFGSTRQNMVQAPGFDTLPLQQANQCPQTLGRVLLGP

>IbWRKY21

MEEIEEANKAAVHSCHRIISLLSQPHDENHYGKLMIESGDAVHKFKRVVSLLNSKLGHARVRKGNMTHTPLSPKFLLENPVTGNDGRPKALHLLPINSLEILNQERSSTVNSALTLGNPLHGKSPIQLPHYTSMTNYRFPQQQQRYELQQQQHRHGEMICRQSSNSGISLNFDSTTCTPTLSSTRSFISSLSVDGSVANMDGSAFHLIGASCSGDQSTFQRKKRCSGRGEDGSAKCGSSGRCHCSKKRKHRVKRSIKVPAISNKLADIPQDEYCWRKYGQKPIKGSPHPRGYYKCSSMRGCPARKHVERCLEDPSMLIVTYEGEHNHPRLASQSTNT

>IbWRKY22

MEDDWDLHAVVRGCAAAASSSAATATATTASPLGSAFQPRQDQDLLCLQDLLEFGAKRNESRRRFNDDLHDLYRPFFRPPPPPSQPPQPSLQRTQAAPPLSPQNTPISPLSVLGGLPDLSPSHQILKPHISPAATIIHPKKQPFPVNISSATTPTTPHTQSPRSKRRKNQLKRVCQVPAEALSSDVWSWRKYGQKPIKGSPYPRGYYRCSTSKGCLARKQVERNRSDPSMFIVTYTAEHNHPMPTHRNSLAGSTRQKPAAAHQPETSGDTMKSGASPPAPEKQESSRDEREDMFEDEDDEFGVANMAIDSLGPDDDFFEGLEDLGGDCFPDSLPGTTLQFPWLTTTTAAGGG

>IbWRKY23

MLLITKSMENKAAELSKTENNNNNNNNPMVTPSFSDQIPATFSLQTLFDIPSSDHNKNPSSSFFDYLFPSQDLSTAVFDLLQTPQPPLLIPPPPSQSLPESSEAVNTPVTPNSSSISSSSNEAAIDDQLPKTPAEEVEQESDRSKKQLKPKRKNQKREREPRVAFMTKSEVDHLDDGYRWRKYGQKAVKNSPFPRSYYRCTTPACGVKKRVERSSEDPSTVVTTYEGTHSHPCPVTPRATTVGIMPEPSNFGSVSAAPGGTGSPPPSSFLIPHHHFHYPMQQQQPFFAITSPLPPPLTYTPSTGNLERRFPPPLSSSPAARDDGLLQDMLPFPLRKEPRPEE

>IbWRKY24

MDNNNNNIDLSLNLNESRRRPRSSELESSEPDKMLQSNAADGEISTGSSLSENQKIEELSVLQREMKRMKEENKALRDAVEQTMKDFHDLHQKFSSIQQKNNHEDKEFAKDFLTLSGTDETRNHRELQERSHQITSDPSPEDGGDEDGDGDGELGLSLTLKSSSSSSSLIGRRMHGEGEERGEKSKAEEMNSTTGFTPTPPPPPPAAMIQNNPPPGFTATSPPNKKTRVSVRARCDAATMNDGCQWRKYGQKIAKGNPCPRAYYRCTVAPGCPVRKQVQRCLEDRSILITTYEGTHNHPLPVGATAMASTASAASFMSHLDSTNPISNLNHAFLIPNYNHNPHFIINPNNPSSHLNIPNLVRNNIPASNPSSSSPSPHFWGPKLPDHPHHRQIELRRSNGGDRTGDNTNNKNDNNKVVVADHQNMSAAIAADPKFRVAVAAALSSLISTKDQTHASS

>IbWRKY25

MSEITERGSIVAGPSRPTALAIAPRPPLESFFNDGFIPGFSPGPMTLVSGFFADSDGCSFSQLLAGAMASPLAKPSVLEDSSAKKGSYGAGSEKQSGYKQNRPVSLAVAALSPLLVVPPGLSPSGLLNSPGFLSPIQSPFGMSHQQALAHVTAQAAINQSYRQMQTEYQQQSSPADAFEHESSLMPNDSFQFQVDDMLDMESLKSEPVEVSQSLRKPAPGVLERPARDGYNWRKYGQKLVKGSDCPRSYYRCTHLKCPVKKKVERSVGGHITEITYKGQHNHELPNPNKRRKDECDLDSGENIQVNSEIASHSWTEMNTSNEAEFSESAQLPTKLPSEQLDVGCDLDEMEETAMALDEDDGQNPKKRSLEAVSSVMPSSHKTVTEPRIIVQTRSDVDLLDDGYKWRKYGQKVVKGNANPRSYYRCTYSGCSVRKHVERASTDPKAVITTYEGKHNHDIPNGRNSNRSQTNANVLQLKQQSTLAVNS

>IbWRKY26

MAASSGTVYSTAFTELLAGDVYPAGVKSLSPPSLPLSPSTQLLDSPLLLSASNLLPSPTTGTFPAQPFSWNSTANVTREALKQENNGGFSDFSFLTNPASAGELNWAYQEGNEVEDAASSEKAIGELNTVVNSQSRNCSDYNHHKNQALKRSDDGYNWRKYGQKQVKRSENPTSYYKCTHPNCPTKKKVETTLEGEITEIVYKGSHNHPMPQTTRRSAASPPSSYVSNGTGQLDSSVATPENSSVSYGDDDFEQSSRKRELGGEEFHEYDSNAKRWKAETGNENEGISALGSRTVREPRVVVQTRSDIDILDDGYRWRKYGQKVVKGNPNPRSYYKCTTLGCPVRKHVERAPQDTRSVITTYEGKHNHDVPAARGGGHSLNRPIPSNSNNSPAIAIRPSAMSHQSSLLAAILPTQGSGNFGANQGTLFSGSREDRLRDDMLLQMLL

>IbWRKY27

MGECIGFEDNWGLQAIVQPPLMASEFSCHDLFDFSQENVLNFDGFVGSAMVSNDALLEELYKPFYQPAPSISFAGEVKQAEGAVHQILQQDGDVKKGGLDKSSPVLETPNYVPKFKRRKNEHKRVVVQVPAEELCEDKWAWRKYGQKPIKGSPYPRSYYRCSSSKGCLARKQVEQSNTAAGMFIVTYTAEHSHSQPTRRNSLAGTIRNKFPPSTASKAVKAAEDSSTAHHVPELVAAASALSSPTDNNWRACSEEETKIKDEDDDEEELETGEKISLSDQIQSGMPQVTMDNNEDFFAGLEDLDGLISQFSYSSQYFPSMFSS

>IbWRKY28

MSEDLRDLYYHHPFQEDERSHAGFLFSGAAATSQIHNATTTNNPHHAFLDPPSSYMSFTDHLIGATEFGRPGGFGFSSSSLHSSADAAAFSAVKDEQKPSTMNDGGGGGGGGSNNNANETPVTPNSSISSSSTEAAGDEDSNKASKRDKQAVDASEDGEDKKETKGKKKAEKKQRQPRFAFMTKSEVDHLEDGYRWRKYGQKAVKNSPYPRSYYRCTSQKCPVKKRVERSYQDPSIVITTYEGQHNHHIPTNLRGTIAGMLPPSLLTPSPLLGAGPPPQISFPPELLAQMTPPHHLFAAHANPFAGAGAAAFHPQNLAQLQLPPDFGLLQDMVPPIFFKQEP

>IbWRKY29

MDAHHLLHHPTVILNSLDDPTLTAPAPAFRPEKRAVNELDFFKRENSDLDSAMDEALVSKGNGRRVGDEAVNHPPVLDTGLDLLGSSKKSMVFHGASPPATMEHKATVEEERSLYLTALREELERMNSENQHLKSMLNQVHEKYNALKMHYAYILEHQHTLKPEIHKMNDGFVEGNERKRKVTDDMKEEHSHSSPEGAGASPPCPEDNAREESPDKVQQKLGRSNGEHSDHLLPAAADQHAPAAKKARVSVRIPCDTPYCSDGCQWRKYGQKMSKGNPCPRAYYRCTMTSTCPVRKQIQRCAEDRSVMIVTYEGEHNHPLPPAARPMASTTSAAATMLLSGAARSADGAGRPANLDALPANFLPTISTFAPVPTITLDLTNPMATQPQTPPPFHSPNPPLPPGIVSAAAAALTRNPSFTAALVSAIASIIGGNNIAAQPPPPPNSAELSDQDVKPSPSIESNVQAKVL

>IbWRKY30

MEKGGWGLTLKSSESVGIFIEKGAKTLNEVDFFSEKRPPPPAAVLVKKEMPDLNVNTGLELVMANNGGKENQSTADDCAAPELERRPAKSEVGRLQVELERMKDENQRLKGMLSQATDTYGALHMYFLTLVQQQQQQQTSTTYQVADGKGTVTARQFLDTAAGAAAAGGQTDEASHNSRTSSGERTESESAGNKVEAAVSRSHEGSIGRSKEESADSETWRPNKVPRLLNPPSKPVDDEDQAAAAASAAATMRKARVSVRARSEASMISDGCQWRKYGQKMAKGNPCPRAYYRCTMAVGCPVRKQVQRCSDDRAVLKTTYEGTHNHPLPPAAMAMASTTSAAANMLLSGAMPSGDVMMNPNFYGRAIFPSGSIATISASAPFPTITLDLTHQPLNSLPNYPRPPIAQFPFSNALQNPQHYVSTPQVFGQAGLYNNHSKFSGLQVSNTLQHPSFAHGGATTAAAITADPNFTAALAAAISSIINGSQHIPAHNNPSSNSNQTSSLPAK

>IbWRKY31

MASRSGLSFDPDPIRHLPYKPPSSEPTQNHRHRLHEPPHRHKFIKLEPFLSMESPVNNRSPPPTIQFPVNRNCSDHRRHAHDQEDHDHKTTTVLGEMDFFAYTKDNNNGDSRASAVDDDVKDLRTSTELDFSINTGLHLLTANTNSDQSIVEDELSPNSEDKRAKGELAVVQAELERMNGENRRLRDTLNQVTNNYTSLQMHVMTLMQQQQQQQSHGKTEESKQNPRQNNGGQMLPRQFIDLGLAAGGGGPTEADEASLSSSEGRSGREGSQSPTNNLDEASRADSPEKGSGWRSNKVARSGHASKSGNIDQATEATMRKARVSVRARSEAPMITDGCQWRKYGQKMAKGNPCPRAYYRCTMAAGCPVRKQVQRCAEDRTILITTYEGNHNHPLPPAAMAMASTTSSAAKMLLSGSMPSADGLMNSNFLARTLLPCSSSMATISASAPFPTVTLDLTQNQNPNQTQFPRPPNPFQFPFPNPSHNPAAALLPQIFGQALYNQSKFSGLQLSQDLENIHAPPSSMPPHQSSQQNPLADTVNALASDPNFTAALAAAITSLIGNPSHSGNASNASNANNNGSVITSSNGNDKVNS

>IbWRKY32

MDDTGEASKPSLQLQNTCADIGGGGGGGDEPSGATTGTETSEEAQVGGSDSEETLDTVDSPSIQLDKSASRPDSLATSSSHVLSEVPIEYSLHPSEFLKEIKDEVGISNQKASTVQAQRRNQLQSADDPSVLELSPTSVTQSISSIPSPTPGERRLSPLENRNGACIQEVDNQNSSNSKALSLVPVLKIQAPDGYNWRKYGQKQVKSPQGSRSYYRCTYSDCCAKKIECSDHTNRVTEIVYRSPHNHEPPRKVNTPKVNKLAISSMPRSQDSKVARLNSNADETVPSTSKKHVKETIPISETKQQDFSGLDDNAETNVKREDCDEPTQKKRLKKCSSSPESLPKPGKKAKLVVHAGGDVGISSDGYRWRKYGQKMVKGNPHPRNYYRCTSAGCPVRKHIERAVDNTTAVIITYKGVHDHGMPVPKKRYGQPSAPLVAATASASMTDSQTKKSEPTTQWSVDKEGALTGETLEHEGEKTVESAKTLLSIGFEIKPC

>IbWRKY33

MSSSGGSLNTCVDNSHHHNSYSSFSSFSFTDLLSNNEESKNPEKGLGSSSSSFNWGISDTHEIPKFKSFPPATLPISPSPVSPSSFLNIPPSLSPSVLLDSPVFFSTSNVLPLSPTTGAFAGLNNNPKEEERKSNDFSFQSRAASSSSMFQSSLGRNSMEEQMSRQQQQPNMGSADFSTMKTDIKPELPQTHSFSQENPAMQQQPAMVHYSQPSQYARAQKAEDGYNWRKYGQKQVKGSENPRSYYKCTFPNCPTKKKVERNLDGHITEIVYKGNHNHPKPQSTRRSSSSSQSVQINPESFNIDVANQSNMMLGSTQRDSFITPENSSASFGDEDLEQGSPSRDDDENEPEAKRWKGDNENEAISSASRTVREPRIVVQTTSDIDILDDGYRWRKYGQKVVKGNPNPRSYYKCTFLGCPVRKHVERASHDLRAVITTYEGKHNHDVPAARGSGGYSLNKPQQPQPAGNMGSSAAPVALRPSTMPNHSLNYQNAIFNPRPQTTQSQQPITLQMLQRPAGNLGYSRLGNSTGSYMPTGKDEPKDDFFSTFLN

>IbWRKY34

MAGFGDHMPIMGDWVPPSPSPRAFFSSVLGNDIESRSNANQPLLPYPKEYASSGNSDSKNGAQGSDQTPKLSSLSERNMNSHGGLLERMAARAGFSAPKLKTDSIRPPALVQNQELRSPYFTIPPGLSPTTLLDSPVFLSNSLVQSSPTTGKFAFPSIGDSRNSALFMGASDNNKETSFNNNDASSFAFKPVIETGPSLFPETISKVPSSSLSWQSVPGIEVSVHSENPRVHQHAEPTLVHTQSGTLEQSVFSRSYTEGVSNIVSEPRTFQTVAGSMEHSPPPDEQQDEDIDQRGGGDPNAVGAPADDGYNWRKYGQKQVKGSEYPRSYYKCTHPNCQVKKKVERSHEGHITEIIYKGTHNHQKPPPNRRAAFGSENAEQDGTIGIGDPIWENVQNGSGAGGPDWGNDNLEVTSSGTQFEPRDPVEGSSPFSNEEDEDDRGTHGSVSLPEGEGDESDSKRRKIETYATDMSGATRAIREPRVVVQTTSEVDILDDGYRWRKYGQKVVKGNPNPRSYYKCTSAGCTVRKHVERASHDLKSVITTYEGKHNHDVPAARNSSHPNSGASNGLSSQTTATQGHVHRPEPSQLQNTMSQFARPPSLASFGFPGGPQLGHTLGFGFGMNQQGLANLAMAGLGPNQGKFPVPPVHSYLGQQRPMNDMRPKAEPKMEPSSDPGLNLSNDSSVYQQFTSRLPLGPQM

>IbWRKY35

MDGRLNSNPFVSEQEEQENSPENSGDSPPSSMFSDAKISSSTSSPKRSRRAMQKRVVSVPIKDVEGSRLKGECAPPSDSWAWRKYGQKPIKGSPYPRGYYRCSSSKGCPARKQVERSRVDPSMLVVTYSCEHNHPWPASRNHHNTTTPPTTAAAIPTPTGAPATPPSPKPAIFSSQAEPDPDEKFADLGEESLIRTEDFTWFSDFESSPSAMLESPIFAENNVRDADVAMFFPMREEDESLFADLGELPECALVFRRGVLEMEEERRRCTAPMCGTTS

>IbWRKY36

MDVPENLLVRQRRAITVLVNGKATTVELQTLLQNPPPDGASSSLPAELVQQIVRSFNQAIFELTSGDAAAQICQIPSASACSGGLTFEDSGETTGKNKKKGRRGRYKKSKNSETWNKVSETQEDGGAWRKYGQKNILHSEHPRCYFRCTHKRDQGCRATKQVQRTSEGLYQTTYFGYHTCKDPQRFPRRKPAADHVFSGDNAPNDHQTVLKAEKQMNLQQEEEDEDGGAIDAVKKEEETAQSEISVSKSPDNNNNNDDNNDKFYDDDNFIWGDIIGESSNYESSFYACSSTSFNDLDMGGVADFGTFFPPH

>IbWRKY37

MDGNYQNTGSPFGSPHHQPVFEPSEFLELSDWAEEEEPAAMHVSGGHYYPLLNPPHHQVPPPPEGVHGGYLQGGPRNNGGSYGGGREKFAFKTKSEVEILDDGYKWRKYGKKMVKNSPNPRNYYRCSVDGCQVKKRVERDKDDPSYVITTYEGIHNHQGPLS

>IbWRKY38

MMSGSDFLQANINPPSKDHPTFNHESFDNLPSIIEDYHRHYYPISEVSSYISYVNHFLNDESPAPAPPNSTGHGFGPSPSLSPPSAAVVTQERSCTTTTTTGSSCSSFDGMPPTSSPHSQMIGYSSNEMRRKLKVNKEEQTIAFRTKTELPVLDDGYKWRKYGKKMVKSNTNPRHYYKCVNEGCNVKKKIERDPEDLDYLITTYEGIHNHESPFVCQLPEDVGSLCHPSSFTLDF

>IbWRKY39

MAVDLMMDYRHGGNGRSVDLTFAKKLEESAVVQEAASGLESVHTFIRLLSQQKHKAAESRGKSTVEIEMVADVAVNKFQKVINLLGRTRTGHARFRRAPVVSSLPVPAKVDTKVYNPTPIQQVPPPVSAAAAAGKTISFSYSPEVSRANSFNISSLTGETESKQASSSSAFQITNLSLASSGGKPPLSSSSLKRKCSSSENNLSGKCSGGSSGRCHCSKRKKLRQKTVTRVPAISMKMADIPPDDFSWRKYGQKPIKGSPHPRGYYKCSSVRGCPARKHVERAVDDPAMLIVTYEGEHNHSLSVAETNSLILESS

>IbWRKY40

MEFTSLVDTSLDLNIKPLRPAGDAGLPPKQEVESNFIGLGINMAIKNEADGLVEELNRVSAENKRLTEMLTIMCEKYNDLREKLKGYMMKNNGCEDNSSPVGVLGSRKRKSESNNVNNGGQHSESSSSDEDSVKKPREEQQQQPQHIKSKTSKVYVRTESSDTGLIVKDGYQWRKYGQKVTRDNPSPRAYFKCSFAPTCPVKKKVQRSVEDQSVLVATYKGEHNHPHPSKMDQSSTPPARSAPAPSTTSALTTLNTSAGPTITLDLTDPKPKPSLPITATAAAARVLPSPADRPDFHQFLIEQMASSLTKDPSFKAALAAAISGKLIIPHNQTEKW

>IbWRKY42

MDAAFGWEFKALINELTQGVECTKRLRASFNSETSFETQNSLLQQIVASYEKSLMILNCGSGRQSQQVPPLSSAPESSISVDGSPRSDDMNKGSKDQEYRDMSKKRKLMPTWTEQVKVGTDNGLEGPPEDGYSWRKYGQKDILGAKYPRSYYRCTYRAMQNCWATKQVQRSDEDPTTFEITYKGAHTCSQAPKSVPPLASPKKQDLKQSIHCKDSLSMQPNQMLMELRSNLRVNTSDMERKETTYPFSFPPTFSGLTDEKPMFQISQVDDNLLGTYSPSFVSPTTPESNYFSVSHQQTSSFGGVQNLHHSESDLTDIFSANTSSTNSPIVGLDYTLDPADFDPNFLFDTSEFFT

>IbWRKY43

MEGEEPPAPPSLPPLLANGDINIQDLFSFPSTSVVEHNNPSMMLSHLPIQSSVVGSDSIDWVGLLSGCMDQMAAPAPASRGGENCVQKNKGKRKKYVPPRVAFHTRSTEDILDDGYKWRKYGQKSVKNSTHPRSYYRCTHHTCNVKKQIQRLSKDKSIVVTTYEGIHNHPCEKLMETLTPLLKQLQFLSRF

>IbWRKY44

MEIKESERIVVAKPVASRPTCSSFRSFSELLAGAINASPPTPCPETSFAAIRPKTVRFKPMVNRAPIGVVSSQAQISGTEVCNSSDKVLKSDSKPTVVYKPLAKLVSKTTVSLLANLGNSTMSHQQALAQVEARVQPPNQDRQHSRPHLSSSLHQTFPSQEETDRTIEPSKTTSQNLEEDQKPLLPTSNGDRPSYDGYNWRKYGQKQVKGSEYPRSYYKCTHPSCPVKKKVERSLDGQIAEIVYKGEHNHSKPQPPKRNSSGTMGQGFVSDGTGQDTNTNNPAWGTHLNERNEGSEGRIENQNEVGLSAHSTYPGKAPLNYDSGTTGAFNAGGGTPDNSCGLSGDCEEGSKGLEPEEDEPRSKRRKSENQSSETGIVGEAAQEPRIVVQNSTDSEILGDGFRWRKYGQKVVKGNPYPRSYYRCTSIKCNVRKHVERASDDPRSFITTYEGKHNHDMPTRNTNAATSEPDMQAHMNKEKP

>IbWRKY45

MDAYPSMFRCSSASQPYLSLMNMMNNNNNSGMESGFLGMKMSSAGGVEVPSSEEIMKSESICTENNNNNGLTEIGAEKSPPSSSAGKKKGEKKMKKARFAFQTRSQVDILDDGYRWRKYGQKAVKNNRFPRSYYRCTHQGCNVKKQVQRLSKDEGVVVTTYEGVHSHPIEKSTDNFENILSQMQIYAAF

>IbWRKY46

MQSPSPENMAEDWKRAIGELIRGQKLTNQLRDSLKDPKVADDLLRQILGTFNKTLWILNKSSIDTDEVSQGGAGDPSSPCYDGRRSEDSSGSCKAFVKDRRGCYKRRKTCETQIKESPNLVDDGHAWRKYGQKVILNSKYPRNYFRCTHKFDQNCQATKQVQQIEEDPPLYRTTYLGKHTCRNFQKCPQILLQPDDASVLLCFGQNSQSDMYTCLPTFSSSIKHESNPHSSRIGSSTSDCFVPSDAAAGHVAPLSSASDYGDVISSGGTMDMTQFIDSDVVDMDDFLIY

>IbWRKY47

MDKGWGLTLENPDQRAAGFFTNKPVFGFNLSPRLNPINAGGSGGMIPINAAAEKRGPPNEVDFFSDKKLPPPQAAADIVVKKEITLHGEPVTKSDLNVNTGLQLVIANAGSDQSTVDDSVSSDMEERRAKNDLSVLQVELEKMNAENQRLRGMLSQVSTNYSALKQHLENLMQSQNQQSSRIGSTQDREVVDRKSEEKKPEKEETTVPRQFLELVPGGGAAAGPTDEPSQSHTSSEERTLSAGSPRNNTELSRHKGIAREDSPDSESWAPNKLPKLNSSKPVDQAAEATMRKARVSVRARSEAPMISDGCQWRKYGQKMAKGNPCPRAYYRCTMAVGCPVRKQVQRCAEDRTILITTYEGTHNHPLPPAAMAMASTTSAAANMLLSGAMPSADGMMNTNFLARAILPCSSSMATISASAPFPTVTLDLTQTPNSLPNYQRPPTQFQAPFAGAPQIPQNYPQLPQVFGQGLYNQSKFSGLHVSHQDIGAAAAQAAQLAQQPRVQPPPPQHPLFADTLSAATAAITSDPNFTAALAAAISSIMGGGSQPNNATNAAAAATSNTNKSSFPGN

>IbWRKY48

MENKFDDLIIKRDSMGIPVFSDEIPSTSPAALQQALLGEADKTTYSLGFLDAQHNNYYYYNTTPIPNTIFDLIIHTHTHTPPPQHQSIPPPPSHSPSPLASTSLLESSEVVNATPPTPNSSSLSSSSNEATPAANDDQHQTSKTVEEDDEEDKTTKKQVKPKKKKKNAQKRQREARFAFMTKSEVDQLDDGYRWRKYGQKAVKNTHFPRSYYRCTAASCGVKKRVERWCEDASIVVTTYEGTHTHPCPIKHTAGSLGIGIMPPNTSSFFPTTTTTAAAQEGGITSSSSSYCFEELDNNNVILIPSRPSSQQLHYPLQHTSTYFQTPTYSNNISNGAAALLWGRTDSISTTTNLLPALPQGLPPSLIPDHGLLQDMVPFQMVKQEERSSPAKKDI

>IbWRKY49

MAAHTEFMYPESMMDAEELIQELLDDESPLFLAPQETIMESSSFAGVSNYSLLNSLIYGHANQPLHDARSCMLERGLMVSRDHHESKYTLRIKTNCGNAMADDGYKWRKYGQKSIKNSPNPRSYYKCTNPKCGAKKQVERCSDDPDTLIITYEGLHLHFAYPFFTLDNEPNKTIDLVPTKKQKKTIAEELVESQEQEQTNHVVYENNPGREDTNNPTPIDEWDSQGLLEDVVPLVIRRPSTICNATTSYSSSSSFLSPPTSPASLSCSTNNCFLSDFDA

>IbWRKY50

MDGNNHHHHYYYTTHHNDDSTFARQDSGFELSEFFDLDVWPPEEDPVFAVAGHPQNPGQATAVDAVMIPSGAGGAVTYAGPSSSITDSGGGMERTAAGVTEKFAFKTKSDIEILDDGYKWRKYGKKKVKNSPNPRNYYRCSVNGCPVKKRVERDKEDPSYVITTYVGIHNHQGP

>IbWRKY51

MENSQFQNPDNGGTSIYDAGVLFVDAPEYYLSDYLLPENIPPPAEQQTMVPMESVGSGSSNPIPTSSNIEYKNRTKKKMERAKSRIAFRIKTELETLDGGFKWRKYGKKMVKTSPNPRNYYKCSSGGCSVKKRVERDREDSSYIIATYEGVHNHESPCVLYYTHEMYPTLPIGAHGWALQPSSLPSSSL

>IbWRKY52

MKNQTALFLGFTPPLAASSEDAKMGSGGTKVVGYPTAEEANDVTNINNSDEEISLLSQKVSSGGVVSKKKGEKKTRMPRFAFQTRSQVDILDDGYRWRKYGQKAVKNNKFPRSYYKCTHEGCKVKKQVQRLNRDETIVVTTYEGMHMHPVHKPADNFEQILNNMHIFPPLLL

>IbWRKY53

MDSAYNGEYKALLNELIQGMECAKQLRVHLNSAASSETQYFFLQRILSSYEKALLILKWRLVGQSHPVATPLPGAPEPSISLDGSLDINNNNNSFKEQQDYNVSKKRKAMPTWTEQVRVGAENGLEGPTEDGYSWRKYGQKDILGAKYPRSYYRCTFRLMHNCWATKQVQRSDDDPTVFDITYKGAHTCNLAPTTSVPPLRSPENQELKQIHHQNENFQAMQSNQMLMNLRASLRVNTDGLDTKETAFPFSFPPTFSGLTDENQHFQSSQVDDNAVALGTYSPSFVSPTTPESNYFSASQQHHTNAFKGVHISSANTSSTNSPIVGLDLDYSLHPATLDPNFPFDTLEFLT

>IbWRKY54

MERHTDTTMTTMECNWPENLSGDRKRAIDELARGRYFTNQLRSVLGKPVGGDQQVSAQDLLAKILRSFTDTLSILSSGKSDEAASQIPASAQLYSPGWDGRKSEESGESSKSSTKDRRGCYKRRKNSQSWMKITPNFYDDGHAWRKYGQKMILNAKHQRSYYRCTHKHDQGCMATKQVQMTEDEPPMYKTTYHGHHTCKSMLKPSQIMLDTRPARDSSILLSFESNNQENSNPFFSSFPLIKQEEEIPSDHEVTHNDNTNNNNSSSSDYLLSPELTTFESNMGSDHGDVLSGINSSSTDSTHSLDDMMMASVDFDDVFLGFDC

>IbWRKY55

MDEDKGNKVDRGVDDFATDSSWALGGGGDSDGVYFFGGGGVEKDESNILSDFGWSFQEEEEHGGGFSSRIDSDLAGNSSNDRFSVLESATTAAATPSDATEPVAVQAEQTSSSSSDDAPEKSTASGGSTSRQPADTASKVKKKGQKRIRQPRFAFMTKSEVDHLEDGYRWRKYGQKAVKNSPFPRSYYRCTNTKCTVKKRVERSSEDPTIVITTYEGQHCHHTVGFPRGGFIGEAAAYMSQSYPLTSQFYLPALQFPQEGSLGIPQPHHQSQGNNRGPNAAMADPCSHPAPQLPPDEGLLGDIVPPGIRNK

>IbWRKY56

MEEIEEANRAAVESCHRVISLLSQPHDQSQYAKLALETGEAVHKFKRVVSKLNSTLGHARVRKVKKIQTPSLPPSILLENPMCRGDDHHPKALQLLPAISLEASNQEKGSSGVIKSGLALGSPSFELNLHGKTPVPLSHQTPIPSYHFLQQQQQRYQQQQQQQLKQQAEMIYRRSNSGISLNFDSSTCTPTMSSTRSFISSLSIDGSVANMDGNSFHLIGASRSADLSSYQHKKRCSGRGEDGSTKCGSSSRCHCSKKRKHRVKRSIKVPAISNKLADIPQDEYSWRKYGQKPIKGSPHPRGYYKCSSMRGCPARKHVERCLEDPSMLIVTYEGDHNHPRVPSQSANT

>IbWRKY57

MDDKGKFDHHEFTADSSGWALRGDADGAYFFGAGGVDKEESSILSDFGWNFQPLDGISGGGGGGGGVGAFDLIHADLAGNGCVSGSGGADESPSGDGEAAATQAEPATSSSSEEAAAAEKPSSSSASRPPPPPADTTSSNKVKKKGPKRIKQPRFAFMTKSEVDHLEDGYRWRKYGQKAVKNSPFPRSYYRCTNSKCTVKKRVERSSEDPTVVITTYEGQHCHHAVGFRGGFIGHEASAYMARLNPLAVQSYLPGLTAQSHTLPIQSHQNLTHNIESHDPSRRPTPPQLPPDEGLLGDMVPPGMWNK

>IbWRKY58

MEDSQSHSHSHSQSQAQHHSGEDLDALQSSHVIHAADGSSDASDAVFSRGVAGSNCGARYKLMSPAKLPIARSAGITIPPGLSPTSFLESPVLLSNIKAEPSPTTGSFSNLHLMQGSGGSAAFSFPDSRAFSQRKSSNFEFKFPIGSSSTSASTSIEPMTSAGLNQQQSETQRQVQNQFISQSLATSSVVESSIPTSNELNLSSAVALHTSSVGTDAIESDDLNQRGKAVNADQSSVTAERSSDDEYNWRKYGQKLVKGSEFPRSYYKCTYPNCEVKKIFERSPDGKITEIVYKGSHDHPKPQPSRRFTPGAMMPIQEDKFEKESFFNGQEDKFNSIAQTGRPEPSGIPILSPQRVGDDIHEGATLLLQGTNDDTDEDDPFSKRRKMDGCVDITPVVKPIREPRVVVQTVSEVDILDDGYRWRKYGQKVVRGNPNPRSYYKCTNAGCPVRKHVERASHDPKAVITTYEGKHNHDLPAARTSSHEMASTAPESGISRVRPEENDVISLDLGVGIGYGTENRTNDQLHSLAPETVPTQVLASGGGMMAVQAPAIVRYGIVNGGINRFGSRENHVQAPGFETLPLQSSNQCPQTIGRILMGP

>IbWRKY59

MEIKEVKKIAVAKPVATRPTCSAYRPFSELLSGAINGSSTGGCSQTAVIAAIRPKTVRLRSAGNQTLGGKVEMPGISARSPPANVLKSDDKPTIIYKPMAKLLSKTTFPQNLNMRSSTSSQQNEAAEETNQVRPSEIRLEAHQNLSLKSGTEKKPVENSKMALQNIEEVQEDERSLFQASGMDCLSGDGYNWRKYGQKQVKGSEYPRSYYKCTHPKCPVKKKVERSSLDDQIAEIVYKGEHNHPKPQLPRSNLRDGQPKGVLVSEDTCNETNNPVRSEQLTLQNEPCGPSTEHKNNTMLSTRSTYSSGPPPPCYPVTSAAAFHGAVSTPENSCTPSGIHREGLEAEGDELKGKRRKCGSQTNNGATLGNGAMETQTVVGSTTDSETTGDGFRWRKYGQKVVKGNTYPRSYYRCTSPKCNVRKYVERAPDDPKSFITTYEGKHNHDIPTRTPNPEASRSSTRAAATKEKS

>IbWRKY60

MEFTSLVDTSLDLNIKPLRPAGDAGLPPKQEVESNFIGLGINMAIKNEADGLVEELNRVSAENKRLTEMLTIMCEKYNDLREKLKGYMMKNNGCEDNSSPVGVLGSRKRKSESNNVNNGGQHSESSSSDEDSVKKPREEQQQQPQHIKSKTSKVYVRTESSDTGLIVKDGYQWRKYGQKVTRDNPCPRAYFRCSFAPTCPVKKKVQRSVEDQSILVATYEGEHNHDLPSKLEQPSATATAAARSLPPAAALNAQPRDLSPPKTALSVPNANGAKTASTPAGSSLPVDRPDFQQFFIEQMASSLTKDPTFKAAIAAAISGKFSPHNNNREKW

>IbWRKY61

MVHYYKNTFKISFLHTDLSSFLKTKMADSLDISGDGAAKQKAGDSLAGRQEGFMTAVLKKDEGRAAATVKAEMKEVKEENARLKTLLAKIEKDYSSLQMRFFDVFSNQPAEIEKKSCKISSPMSSHHHDEETQISLRLGRSPSPDRRQSRVIDDINAAAAAKSTDEDDDEHNQTLKLGLDYGGDNKSTEPNLELSSGRQSPDNSASETKEEDAAAAGETWPPSKALKATRSGDDELSQPSVKRARVSVMARCDTPTMNDGCQWRKYGQKVAKGNPCPRAYYRCTVAPSCPVRKQVQRCADDMSVLITTYEGTHNHPLPVAATAMASTTSAAASMLLSGSTTSQTGLRSSPSPATNFFPGLNFSLPADTSRTTRPLYFPNSSSPPFPTITLDLTTSSNNVSSMFSSNVMKSTHRFPSTNLNFSSSESNISPAIWSTGGYTNYSTIYNRNNNNILGTSQPGKSSQEQQFYGQAAAASQQALTETLTKAITSDPSLRSVIAAAITSMVGNNAPMQHKRVKVNDEVIGTHQAIS

>IbWRKY62

MENGWGLTLENSIFKNGLGSSRPRFDTANMFPVKKDDGERRQQAVLNEVDFFSEKKKPVDSGFVVKKETSNDEPPIRTDLNINTGLQLVTANTGSDQSTIDDGVSSGMEERRVKDEVAGLQAELERMNGENQRLKGMLTQVTNNYTALEMHLAALMQQQKKNSMAESAHEVVDRKSEEKKYEKEGSTVPRQFLDLGPSGLTGAEQTDEPTHSPTTSSEEKTISASPRNNVDSSKHKRSAREESPDLESWNPNKAPKSIISSSSSRPVDDQQASTDATMRKARVSVRARSEAPMISDGCQWRKYGQKMAKGNPCPRAYYRCTMAVACPVRKQVQRCAEDRTILTTTYEGTHNHPLPPAAMAMASTTSAAANMLLSGSMPSADMMMNPNFLARAILPCSSSVATISASAPFPTVTLDLTQTPTSLPNYPRLPPSQFPGAGAPHSLPGFAVTPPQVFGQGLYSQSKFSGLQVSHEAQHPLLPPAHPSLSDTLSAATAAITADPNFTAALAAAISSILNGSQPNIVNSTNNNTTATANVPNSNPTNNCNKTH

>IbWRKY63

MEPDWDLHAVVRGCAVTSTTAAAATTTSPFCSFNPRQQDENLDFSHEPFDFSSSTDNSATWFNEELHDLYLPFLHRREPPSLPPQSPPPPFPVLRGLEGSVLHNQLNITTTRIQANSQSLSSNASSTTSSNSRPQTPAGKRRKNQMKRLCQVPAEDLASDMWSWRKYGQKPIKGSPYPRGYYRCSTSKGCLARKQVERNRSDPNMFIVTYTAEHNHPMPTHRNSLAGSTRRKGASQQTTTSGSETNRPSTSSPPPEKQESSRDEKGGIFYDEFGVSNMEIMDGNAPEEDEDFFEGMAELGESLKSDDAGDSFSDNFQDAMQFQCWLPTTAGGGG

>IbWRKY64

MMMAQSVGGCRLEDGALQAVVHGSSTRDSAAAAGFGVFDGRGSPDEDFGVFEDVVGSEMTRIWNSDELEELYKPFYNPVVCASSPVCFPKEVNEQQAVELQDDYQMQPPAAPPAATAVYVPKYKRRKSEHKRVVLQVPLEELSDDKWAWRKYGQKPIKGSPYPRSYYRCSSSKGCLARKQVERSCSEPGMFIVTYTAEHCHSQPTRRNSLAGTIRNKFPTTAASKKPQHNYSSEDQDHPPASFMSPALSSSVVSPAMVKEEEKNEYNINNVKYDVEDDFFAGLDDLDGLISHFSSACDQSFLYS

>IbWRKY65

MDGRFSNQPPFINDQDDSENSPENSADSPRSGMFHDTKMASINSPKRSRRAIQKRVVSVPINDVEGSKLKGESSFPPSDSWAWRKYGQKPIKGSPYPRGYYRCSSSKGCPAKKQVERSRVDPNMLVVTYSCEHNHPWPAARNNHAHRNAVSLTAATAAAAPTTRSSSKAAAADAEDAREAEAEAEASEFSAQPKPETSEKFVGLGDSPSVIYSDEFGWFSSFEPTTSSSTVIESTSILTEARVTHADMSVIFSMREEEEGEESPFAGLGELPECSREFGIGMMERDEARRRHNLTPWCGTA

>IbWRKY66

MSDNPFYFHNHMGSGRINTFPFFGDDNSDHHNPSSSIYSSSDHHPPPSAPTQNLLHQEFLPSPFMSFTESLQGSMDYHTLSNAFGMSCSSSEVVCTPTDHHHHHHQQQQQQNQESSRKSSVSAGEAAGENIPFVAANSSVSSSSSEAAVGDGEEDSSKSNKDLLLPKGCEDGDDKSTKINKGAAKKKGEKKQREPRFAFMTKSEIDNLEDGYRWRKYGQKAVKNSPFPRSYYRCTSQKCTVKKRVERSYEDPTIVVTTYEGQHNHHCPATLRGNAVALLSPASFLSPSPAALMPNFHQDLLLNPMLSGAPNFQPSSMYGGYHHHHLGLNPHHYDHHQITQSPVDQYTLFQDMVVSSLGHKQEHP

>IbWRKY67

MSDMNPNFLGPNMMSNPPSYTNPNSNNLHFYPEMLQDYDYDYAQDFDLSYVNTLFNDDHHHYSNVVSNNPFASSSTSSSSSLFSHPPPPPTIVQDKSSTTSASSGSSGFDGMLPTTTYPMQEVKSMRQMVNTTKAKERHSIAFRTKTELEILDDGYKWRKYGKKKVKSNSNPRNYYKCSHEGCIVKKRVERDGEDSKFLITEYEGIHNHESPYVIFYY

>IbWRKY68

MSAPTFLEPNPNYLNYSTAPFSSNIIPDYDYSCYQDLDFFCADNHHLFSDDFTSSNDNNTFGYPSSDLPQTPLVLPEKSNNSTTGSSSCSSDGMPTSTNYMPVKCKRETMKGPKMKEKRAIAFRTRTDLEVLDDGYKWRKYGKKKVKSNSHPRNYYKCSSGGCKVKKKVERDQDDAKYLITTYEGVHNHENLYVIYYQGMPATLTSNGLSLPAASQPY

>IbWRKY69

MMETRLHHNSSSCFHKQEELSAGTPPENGVESPLSGDEAPEVSTPSPRKRRGAQKRVVSVPAGDGDGSRSKSEVYPPPDSWSWRKYGQKPIKGSPYPRGYYRCSSSKGCPARKQVERSRLDPTMLLITYSSEHNHPHPTKIHHHYAAGTGTGTGAATSSSSAATTPTDSAADPGSPPKPAPKEELPIFADPDPENDTFPELAGGEMGWFSDVGPCCFMESATAVAGPTLCHDTDVALTLPIREEDQSLFGDLGELPECSVVFRRCRVETPCYAGTG

>IbWRKY70

MPKPTMETPNPFHTHRKRVISVLLKGKKSATQLQTLLRNFSHGSQEKSHLVLEILGSFSEAVSQLKNGLPPPDSELSGGRNSGDNPAKARRGCNNRRKCSDTWINVSNTKEDGGAWRKYGQKQILNSKYPRCYFRCTHKHSQGCKATKQVERISEDEYKTMYFGQHTCQDSFRAPVLVIKSISTVDSTQSPSFECITQDGNGGGDHYDDVVVNQENHQEFKDQYCMTSGYSGTNNLDVDESCFADLSNLFKSEEMRLLDY

>IbWRKY71

MSENQFYHDHTGFNPLFIGDDGDDHRNPSLYSSNLPHPEFDPSSPFVNYNVVSSSGFPTLLWSSSTRSEVVGPPSSDHHVQECSRKSSGSVGQPPASSSSSEAAGGGGGGEEDSSKSGKNLEGVECEDGEDKSKKMNKGKKKEEKKQREPRFAFITKSEIDNLEDGYRWRKYGQKAVKNSPFPRSYYKCTTQKCPVKKRVERSHQDPTTVITTYEGQHNHHCPATLRGNAAAAAMFSSPPSFFPTSSSAPPPPRDLFAHQMYPAAAPPHFQPPIMYDYQQSLIGGHAPPQPQFDYGMFQEMVASLVHKQEHNNL

>IbWRKY72

MEEDKKKAADSSGDDEGYCTHEIGDGNKENERDTLKASSSPNHKTLSSDKEENELLESAKAEMGEVMEENQRLKMYLERIMKDYRTLQMQFQGMVEKEGEKAAKSDDNTPQITEESELVSLSLGRASAEMKREEQNRPVIVCAGKDKVDNEDNDQKEGLTLGLDCKFKSLQPNNPSTDNSSDEVKEENGETWPPSKALKTMRSGEDEVSQQNPAKRARVSVRVRCDAPTMNDGCQWRKYGQKIAKGNPCPRAYYRCTVAPSCPVRKQVQRCADDMSILITTYEGTHNHPLPLSATAMASTTSAAASMLMSGSSTSASTSMPPGTTTTTATSTSTTNLNGLNFYLSDTSKPKPFYIPTSSITPTLGHPTIVLDLTSTAPSSSSNLSRIGSLANFPPRFSSTNLNFSSLESNPLPISWNLGTTQPYNKPHITQSLTFARQQQQQPQDNQILFQSYLSKNNLNNNNNNPSQSLPQETIAAATKAITSDPNFQSALAAALSSIMGTNNGNNGASLNLGIHGLAEKLSHNLKAAAEPFPILSSFSPSPNPNKCSSSLLSRPTTTSSANPHPGNLMYHLQQTSKSKSSSPGDSRDQII

>IbWRKY73

MDGEKNDWDLGAVVRGCKNLNGNSSSQDVHEHFNGGFASQTYASIPLLPQPSHYYSSVSPSIGTERRYFGLEEVIHRFTNGKVREPALVPQATITPPTSLDKADGSGGMGKDTPALQSPDPYFGLEEVIDRFTGGKMREPILDLKATITRPTSLDKAGGSGLGGIGKDTRAPQSPNPSPTLEPQQPLPSSQTSSPEKDGGLEDENLPHQVEEVTEVEMSVVKVKVPVEKVQVPAEKVDEWDGWEWRKYGTKMMNDSPHSKGYYRCNHEKKCPAKKHVQLSYMDESTYIITYKGNHNHPPPVQTTTSKRKKRSWARARPPAPPASKGEGSFSTPSTNTAT

>IbWRKY74

MAVELMMSYRNVDVNGGGGGRIGFVKSLEESSAVVKEAASGFESVEEFIRLLSQGKKKQQGDREKAAMEIDVVADVAVNKFKKVIDLLGRTRTGHARFRRAPVASSSPVMEAPADNKVYSPTPIQQVPPPASYDYSAAAHRYSAAAAAPMTISFTCSPEISRANSFNISSLTGETDSKPMLSSSSAFQLTNLSQVSSAGKPPLSTSSLKRKCSSSENNLSGKCSGSSSRCHCSKRKKLRLKRVTRVPAISMKLSDIPPDDYSWRKYGQKPIKGSPHPRGYYKCSSVRGCPARKHVERAVDDPTMLIVTYEGEHNHSVSVAETNGLILESS

>IbWRKY75

MEAYPTLFGSSSSSPLRLSPHLSLASMGNHHEFIKDDGAQGDLQFHGGGKGMKGLLEQLKRTEAAAAAPTSDMKSHDSSISTNSLTAAESEAKSSSPGYKIKKGEKKIRKPRYAFQTRSQVDILDDGYRWRKYGQKSVKNNKFPRSYYKCTHQGCNVKKQVQRLSKDETVVVTTYEGMHSHPIEKSTDNFEHILSQMQIYASF

>IbWRKY76

MEKVGVLEGNSLIINELTQGRELAAQLKGQFDSFTSPEICEPLVEKILSSYEKALTLLNFKLFFGGDPNAMDSPLPLLANNSNVSPTGEASDGDSSKEQCHVFKKRKTSPQWSKQVRVCSATGLGANLDDGHSWRKYGQKDILGAHHPRAYYRCTHRNTQGCLATKQVQRSDGDASVFEVTYKGRHSCKASHPALISGENERPKPQFQVQQPEGKQLQAQSLVLDYGLNQKLETTEEDHVLPPFSFPPTIKCEVADKENNLLKCCSPSPFTAPSTSEECMYLSFLPGQNDDDDFGLSQILDRSESDLTDHMISTPTSVTNSPFQDWDFLADQPNLDATDISKYFS

>IbWRKY77

MDNIGGDREFLQSLISELANGRDAATHLQMILNAPSSFSPETRELLVHNVLASYDRALGMLNYSPESAAVQPPAAAGPALGIESPSSFTGSPHSEDSDREYDGSRRRNAPRWTQKVQVCPGSGLEGHLDDGYSWRKYGQKDILGARYPRGYYRCTHRHAQGCLATKQVQRSDEDPTIFDITYRGRHTCNQAGGNPNPPQNQEPNMPESQRNIIPFTQQQGQPSETLLSFRRQLRVETSDLDNTHQDNHQQQFTYLYTFPSSSDQSYTFRPPSAPDASNFSWSISPSFGSPASTTASSDFGRRPRQTTEAPAASTANAGGMIPAVSAGNSPAVDPGFAFGTMGFDSNFTFDSHGFFPNP

>IbWRKY78

MESLVNNGSSPPPTIQFPVSRFINPADHRHRRHEHGQEDPVDKRKVMDEMDFFADKSKDENAGRKDLHAPTELDFSINTCLRLLTSNTSSDDQSMVEDRLPSDCEKNGNKTELVAVKAELERMSAENRVLRDMLSQVSDNYTNLQTQMISLMQRQKDNHHNHGKIDEEDNSNNGGIKFPRQFMDLGLATRRSNDGAVDEANLSSSPEGRNVRERSSRSPTSREESPEKGSSSGWGPNKVPRLDHGPESSSVDQATEATMRKARVSVRARSDAPMITDGCQWRKYGQKMAKGNPCPRAYYRCTMATGCPVRKQVQRCAEDRTILITTYEGNHNHPLPPAAMAMASTTSSAAKMLLSGSIPSADGLMNPNILAKTLFPCSSSMATISASAPFPTVTLDLTQTPNPNPLQFPPRQPNNNPQFPLPCSTNPAATLPQMFNQALYNQSKFSGLQLSQDLENHQSSQNPQLIDTLNSLTGDPNFTTAAIAAAITSIIGNSSNPATVTNGASNNANNGGSKSTNSSVPAN

>IbWRKY79

MATSPPEKLSTDQKSVVGELVQGRDLATQLQILLREPFSDQRLESAQDLVVKILRSFTEPLAVLRCYDQSGDDGGGSPAESGNCKGVKNRRGCYKRRKNSETWTVVSSTMEDGHAWRKYGQKDILNAKFPRSYYRCTRKYEQGCRAMKQVQTMKEDPITYHTTYIGHHTCRDLLKAPQFIGGSTYPGYDSNYMAGSESKIPEEVQEMKPELIKEETVLSDLTTENVSSLDSINLWSGLEALDSSMPAMVSPRGGSDDQDVDSTMYSRNATCTITSHNSDMDFVLGCLDFESDDEFHFDESYVKYF
