# Supplementary material for: A systematical genome-wide analysis and screening of WRKY transcription factor family engaged in abiotic stress response in sweetpotato
Source: BMC Plant Biol. 2022 Dec 28;22:616. doi: 10.1186/s12870-022-03970-6 (PMC9795774; doi:10.1186/s12870-022-03970-6)
Supplement: Supplementary file 10 — Additional file 10. [file 12870_2022_3970_MOESM10_ESM.docx]

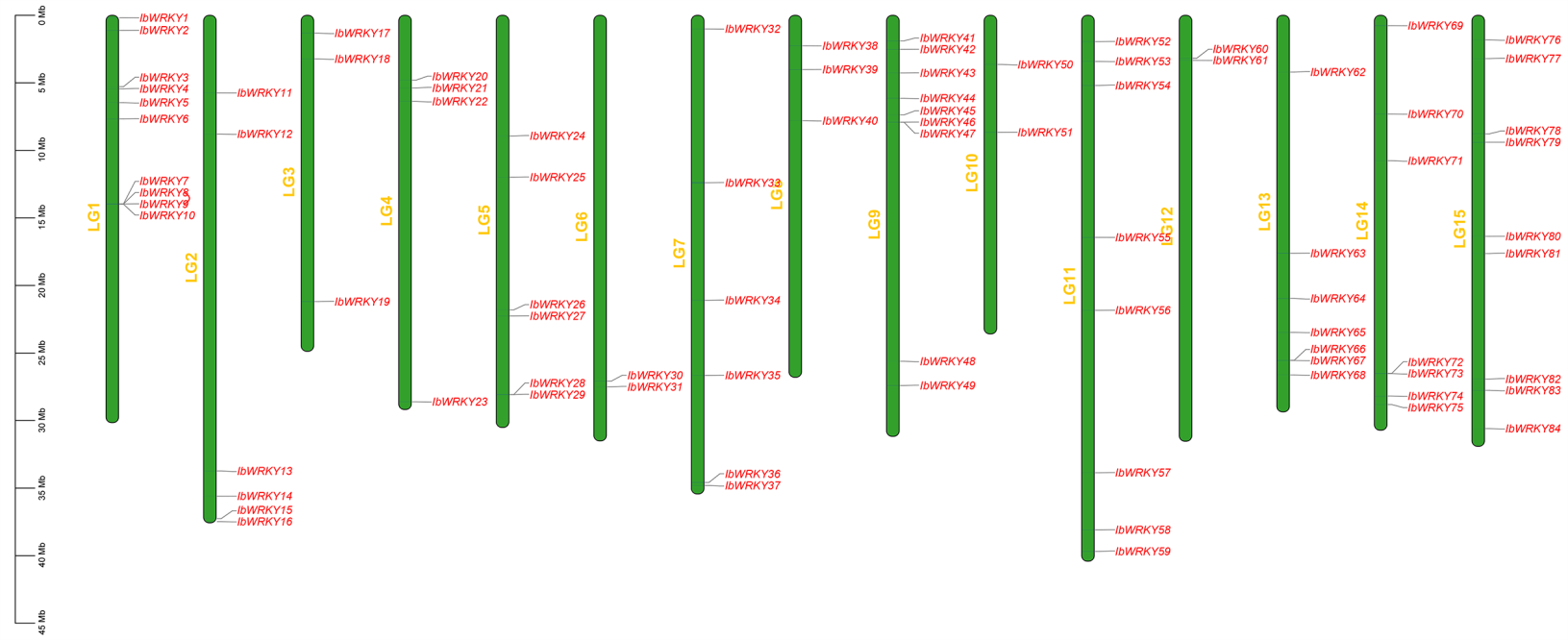
**Additional file 1**. Chromosomal map showing the uneven distribution of 84 *IbWRKY* genes on 15 sweetpotato chromosomes. The chromosome numbers 1-15 are indicated to the left of each chromosome as LG1-LG15, and tandem duplicated genes are indicated as red arcs. The scales were indicated the genome size of sweetpotato genome (Mb).
