# Supplementary material for: A systematical genome-wide analysis and screening of WRKY transcription factor family engaged in abiotic stress response in sweetpotato
Source: BMC Plant Biol. 2022 Dec 28;22:616. doi: 10.1186/s12870-022-03970-6 (PMC9795774; doi:10.1186/s12870-022-03970-6)

**Additional file 4**. Unrooted phylogenetic tree of conserved WRKY domains retrieved from sweetpotato IbWRKYs. The phylogenetic relationships were derived by the Maximum Likelihood method and the best evolutionary model JTT + G obtained through MEGA X was employed with the bootstrap value of 1000. Different subgroups are named based on the reports in *Arabidopsis*.


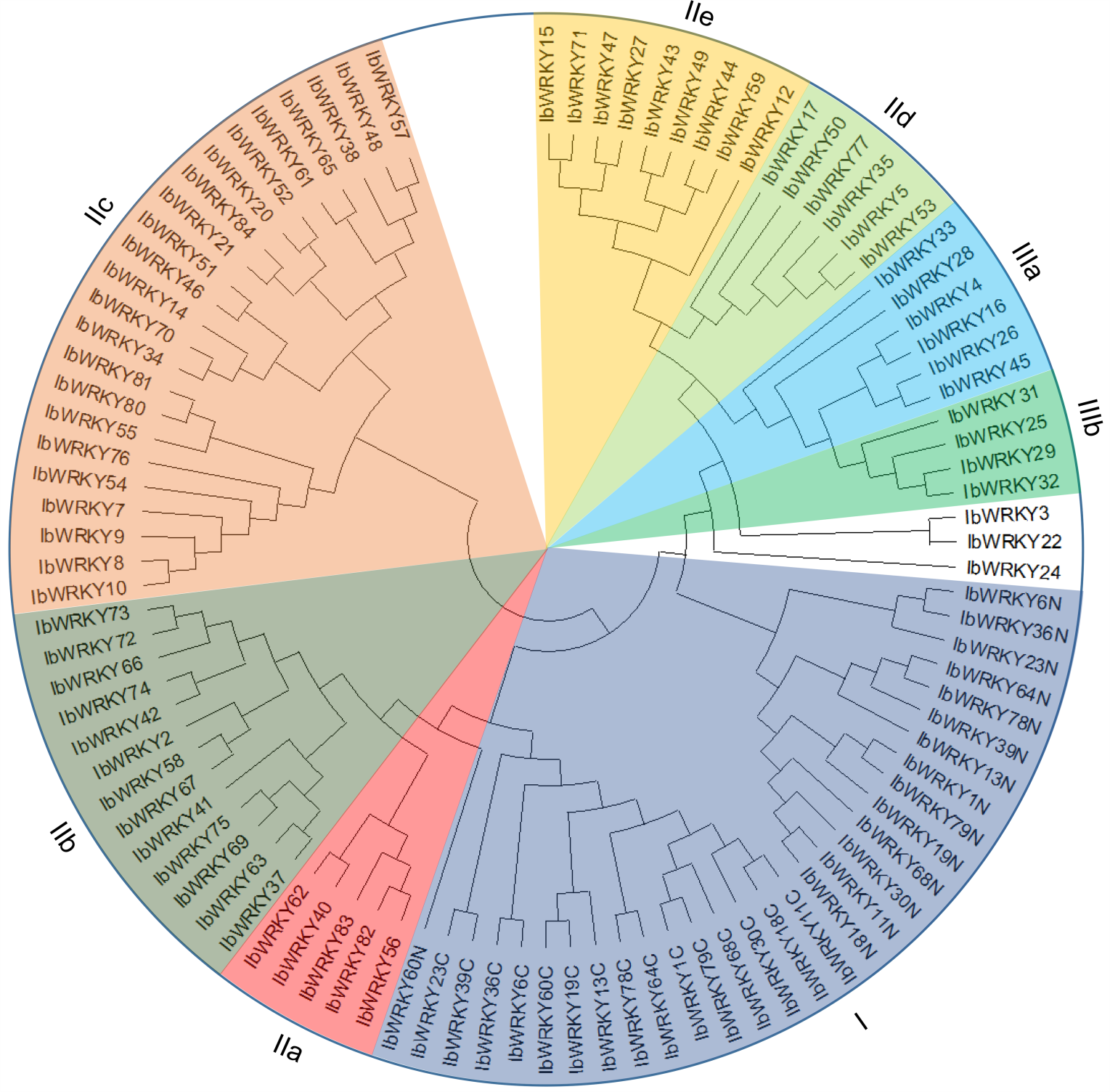

Supplement: Supplementary file 11 — Additional file 11. [file 12870_2022_3970_MOESM11_ESM.docx]
