# Supplementary material for: A systematical genome-wide analysis and screening of WRKY transcription factor family engaged in abiotic stress response in sweetpotato
Source: BMC Plant Biol. 2022 Dec 28;22:616. doi: 10.1186/s12870-022-03970-6 (PMC9795774; doi:10.1186/s12870-022-03970-6)
Supplement: Supplementary file 12 — Additional file 12. [file 12870_2022_3970_MOESM12_ESM.docx]

**Additional file 11**. The 225 seed sequences of WRKY (PF03106) from the Pfam database (<http://pfam.xfam.org/>).

>D8RSA8_SELML/111-168

DDGYRWRKYGQKIVKGNPYPRSYYKCTNVGCPVRKHVERASNDPKSVITTYEGKHNHD

>F6GX25_VITVI/254-311

NDGYRWRKYGQKVVKGNLHPRNYYKCTSTGCSVRRHVERASNNQKSIIATYEGKHNHE

>K7LJY1_SOYBN/143-200

DDGYNWRKYGQKVIKGHTYPRAYYKCISAGCYVRKHVERDSCNRKIVINTYEGKHNHE

>K7LJY0_SOYBN/131-188

DDGYQWHQYGLKTMKGNLFPRVYYKCASAGCCARKEVDRNSVNTKHVITTYVGKHNHE

>A0A3Q7IAT7_SOLLC/509-566

EDGYRWRKYGQKLVKGNPNPRSYYKCTNSGCSVRKHVERSPLDQMSVITSYDGKHNHD

>M4EPX6_BRARP/284-341

DDGYQWRKYGRKIVNGNSNPRSYYKCTYGGCNVKKHVERGADDVKLLVVTYDGKHNHP

>M4F7P5_BRARP/264-321

DDGFRWRKYGQKVVKGNPNPRSYYKCTYTACDVKKHVERGAEDVKFLLVTYDGIHEHD

>M4FA92_BRARP/266-323

NDGHRWRKYGQKIVKGNPNPRSYYKCSHKGCIMKKHVERAADDLRMLLVTYYGKHGHA

>M4EPX7_BRARP/108-165

DDGYSWKKYGQRLIMGNQNTRSYYKCTFAGCDVKKHVERRADNVKLLVITYYGNHEHD

>D7KMZ8_ARALL/325-382

NDGYRWRKYGQKVVKGNPNPRSYYKCTNNECKVKKHVERGADNNKLVVTTYDGIHNHP

>D7MF96_ARALL/373-429

DDGYRWRKYGQKVVKGNPNPRSYYKCTANGCTVTKHVERASDDFKSVLTSYIGKHTH

>I1GQR9_BRADI/375-432

DDGYRWRKYGQKVVKGNPHPRSYYKCTFQGCDVKKHIERCSQDSTDVITTYEGKHSHD

>WRK25_ARATH/329-385

DGFRWRKYGQKVVKGNTNPRSYYKCTFQGCGVKKQVERSAADERAVLTTYEGRHNHD

>J3MMM2_ORYBR/273-330

DDGYRWRKYGQKVVKGNPFPRSYYKCTYLGCDVKKQVERSVEEPNAVITTYEGKHIHD

>A0A3Q7FWV5_SOLLC/387-444

DDGYKWRKYGQKVVKGTQHPRSYYRCTYPGCNVRKQVERASTDPKAVITTYEGKHNHD

>K7LGJ6_SOYBN/248-305

DDGFRWRKYGQKVVKGNSNARSYYKCTAPGCSVRKHVERAAHDIKAVITTYEGKHNHD

>E1ZGV1_CHLVA/113-170

DDGYRWRKYGQKIVKGNPHPRSYYKCTHPGCNVRKQVERSGRNARMLVTTYEGTHTHD

>F6H336_VITVI/353-410

SDGYRWRKYGQKMVKGNPHPRSYYRCTSAGCPVRKHVERDTDDKTTIIVTYEGKHDHD

>D7U1A8_VITVI/362-418

DGYRWRKYGQKMVKGNPHPRNYYRCTSAGCPVRKHIETAIDNTSAVIITYKGIHDHD

>B9RNV8_RICCO/233-290

SDGFRWRKYGQKMVKANSYLRSYYRCTSAGCPSRKHVEMAIDDARTTTIKYEGKHDHD

>M0U1R5_MUSAM/391-448

TDGYRWRKYGQKFVKGNRNPRSYYRCTHSGCPVRKHVERVPHDAKALLITYEGEHNHE

>F6HHL8_VITVI/281-338

NDGYRWRKYGQKLVKGNTNPRSYYRCSNAGCPVKKHVERASHDPKMVITTYEGQHDHD

>M4D9M7_BRARP/278-335

NDGYRWRKYGQKSVKGSPYPRSYYRCSSSGCPVKKHVERSSRDTKMLIMTYEGNHDHD

>A4S4Z2_OSTLU/45-105

ADGYRWRKYGQKNIKGSSFPRSYYRCTERGCPARKKTELRRASEDGEMETVVCYEGEHTH

A

>Q013H7_OSTTA/23-83

NDGFRWRKYGCKLIAGNMVPHPTERSYYRCKHFGCPARKRVEVEKVTGATRTVYEFEHTC

R

>A4S1F5_OSTLU/27-88

DDAHRWRKYGHKKIGKRDKTSNDLSERSYYRCSFEGCPARKRVGWCPETGHTRVWYEFEH

TC

>E1ZGV1_CHLVA/4-61

DDGYNWRKYGEKQVKGSPFPRSYYKCSHPGCPAKKMIEREPKTGRISQAELKNEHNHA

>M0TLT1_MUSAM/216-272

DDGCHWRKYGEKQVKGSDNPRSYYKCTYPSCPRKKQVERSSDGQITEIVYKGTHSHS

>D8T390_SELML/1-56

DGYNWRKYGQKQVKGCDNPRSYYRCTHPDCSAKKLVERSVSGETTQIVYKGDHSHS

>M1C548_SOLTU/54-110

DDGYTWRKYGQKQVKGSNFPRSYYKCTQQNCLVRKKVECAPNGQVIEIVYNGAHNHP

>B3H4T8_ARATH/468-524

NDGYNWQKYGQKKVKGSKFPLSYYKCTYLGCPSKRKVERSLDGQVAEIVYKDRHNHE

>I1IVB4_BRADI/118-173

DGYNWRKYGQKQVKGSEFPRSYYKCTYPTCPVKRKVETTLDGQIAEIVYNGEHNHP

>M0U8S7_MUSAM/172-227

DIYHWRKYGQKQVKNTENFRSYYRCADSNCLAKKKVECYPDGKICDVIYRGKHNHD

>M0U1R5_MUSAM/220-275

DVYNWRKYGQKQVKSSENARSYYRCTDSNCSAKKKVERCPDGTIIEVIYRGKHNHD

>M4D1B7_BRARP/162-218

HDGYHWRKYGQKQVKSPKGSRSYYRCTYSDCCAKKIECSNDSGNVIEIVNKGSHSHE

>D7U1A8_VITVI/189-245

ADGYNWRKYGQKQVKSPKGSRSYYKCTYSDCYAKKIECCDDSGQVIEIIYKSRHNHD

>I1LT43_SOYBN/160-216

SDGYNWRKYGQKQVKNPMGSRSYYKCTHSNCCAKKIKFCDHSGHVIEIVYKSQHNHD

>F6H336_VITVI/139-196

NDGYNWRKYGQKQVKSTESSRSYYRCTYSDCDAKKKVQQCHQSGFVTGVIYKGFHNHD

>B9RNV8_RICCO/68-125

TDGYSWRKYGQKQVKSSRSFRSYYRCSHSNCHAKKKVQRCDQSGQVIDTVYIGQHNHD

>M0SDB1_MUSAM/144-200

DDGYNWRKYGQKQIKGSENPVGYYKCTYPDCPTKKKVEMSIDGEITEIVYKGSHNHP

>WRK25_ARATH/166-222

NDGYGWRKYGQKQVKKSENPRSYFKCTYPDCVSKKIVETASDGQITEIIYKGGHNHP

>Q0V871_ARATH/24-81

DDGYNWRKYGQKQVKGSENPRSYFKCTYPNCLTKKKVETSLVKGQMIEIVYKGSHNHP

>M0YT11_HORVV/178-234

DDGYNWRKYGQKQMKGSENPRSYYKCSFAGCPTKKKVEQAPDGQVTEIVYKGTHNHP

>F6GX25_VITVI/36-92

EDGYNWRKYGQKSMKGSEHTRSYYKCTHLDCPMRKKVQQSHDGQITEIIYKGGHNHP

>J3M640_ORYBR/195-252

EDGYNWRKYGQKQVKGSENPRSYYKCTYNSCSMKKKVERSLADGRITQIVYKGAHNHP

>J3MYJ7_ORYBR/269-325

EDGYSWRKYGQKQVKNNENPRSYYKCTQTACTVRKKVEYAQDGQITQIIYKGAHNHP

>F6HHL8_VITVI/106-162

EDGYNWRKYGQKHVKGNEFIRSYYRCTHPNCQVKKQLERSHDGQITDIIYFGKHDHP

>I1JJR0_SOYBN/113-169

KDGYNWRKYGQKHVKGNEFIRSYYKCTHPNCLAKKQLQQSNNGHITDSICIGQHNHP

>M1BJ77_SOLTU/62-118

ENGYNWGKYGQKLVKGTEFPRSYYKYTYPNCEVKKTFERSPDGQITEIVYKGFHDHP

>W1NWG1_AMBTC/259-315

EDGFNWRKYGQKQVKGSEFPRSYYKCTYPNCQVKKIMERSLDGRITDINYKGQHDHP

>A0A3Q7IAT7_SOLLC/297-353

EDGHNWIKYGENQVKGSEYPRSFYKCTHPNCLVKKEIARYHQGHVTEVIYNGAHKHP

>I1IR53_BRADI/286-342

EDGYNWKKYGPKQVKSTEYPRSYFKCTHPNCPVKKKVERSQVGQITEIIYKGTHNHP

>D8RSA8_SELML/4-60

EDGFNWRKYGQKQVKGSEFPRSYYKCTSSGCPVKKKVERSQDGQVTEIVYKGEHNHP

>I1J5V4_SOYBN/175-231

DDGYNWRKYGQKQVKGSEFPRSYYKCTHPNCSVKKKVERSLEGHVTAIIYKGEHNHQ

>C5WVP7_SORBI/215-271

DDGYNWRKYGQKAVKGGEYPRSYYKCTHASCPVKKKVERSAEGYITQIIYRGQHNHQ

>J3MMM2_ORYBR/104-160

DDGYSWRKYGQKAVKGGEYPKSYYKCTHLNCLVRKTVEHSADGRIVQIIYRGQHTHE

>I1MBG9_SOYBN/221-276

GDGYNWRKYEDKVVKGSANQLSYYKCTQPTCYVKKKVERTIEGEIVDIHYQGTHTH

>WRK34_ARATH/178-234

DDGYNWRKYGQKLVKGSEYPRSYYKCTHPNCEAKKKVERSREGHIIEIIYTGDHIHS

>M1A3W0_SOLTU/224-280

SDGYNWRKYGQKMVKASECPRSYYKCTHVKCPVKKKVERSIDGYITEITYKGHHNHE

>M7ZE84_TRIUA/87-143

DDGYNWRKYGQKVVKGSDCPRSYYKCTHPSCPVKKKVEHAEDGQISEIIYKGKHNHQ

>M4E2W1_BRARP/156-211

DGYNWRKYGQKQVKGSDCPRSYYKCTHPKCPVKKKVERSMGGLVSEIVYQGEHNHS

>D3BE88_POLPP/755-813

SDGYQWRKYGQKNVKGTQFPRSYYKCTYPGCTVKKQMERRSSSDDTLNHVVYKGEHNHE

>F4PLI3_CAVFA/633-691

ADGYQWRKYGQKNVKGTQFPRSYYKCTVPGCTVKKQVEKLSETDETKNRVVYKGTHNHD

>WRKY1_DICDI/814-869

SDGYQWRKYGQKNVKGSLHPRHYYKCTFQGCNVRKQVERIGDTNQNSTVYKGEHCH

>J3NAU6_ORYBR/110-173

DGYQWRKYGQKQIEGAKYPRSYYRCTNSTDQGCGAKKTVQRNDDGGGGGAARYTVAYISE

HTCK

>K3Y9C1_SETIT/76-139

DGYRWRKYGQKQIEGAMYARSYYRCTRSAEQGCPAKRTVQRNDDGDNGGAAPKYTVVCMG

EHTC

>C5YRA1_SORBI/118-182

DGYQWRKYGQKQIEGAMYPRSYYRCTRSAEQGCAAKRTVQRNDDDGGGAAAAPEYTVVYV

SEHTC

>C0PKA1_MAIZE/112-175

DGYQWRKYGQKQIEGAMYPRSYYRCIQSAKQGCQAKRTVQRNDDDGATAPPEYTVVYVAE

HTCT

>C5XNA2_SORBI/124-184

DGYQWRKYGQKRITKTQFPRCYFKCSFHRERNCRATKQVQQCSNDDPPQYVVIYFNEHTC

D

>K3XS92_SETIT/45-106

HDGYQWRKYGQKMIRGNTYPRCYYRCTFHQDHGCPATKHVEQTNSQDPPLFRVIYTNEHT

CS

>K3XPF8_SETIT/105-165

KDGYQWRKYGQKKIQNCNFSRYYYRCNRHRRCLAKKKVQQQDGSLLPPMFEVTYVNEHTC

H

>M0VMN8_HORVV/120-183

KDGYKWKKYGQKNIKNRKFARLYFRCMHSHERGCWAKKQLQQEDSSIGSPPLYKVTYLND

HTCH

>T1NKL9_TRIUA/123-186

SDGYQWRKYGQKNIHKRKFARSYYKCMFSHDRGCRAKKTVQQHDSSSGHRPMFQITYMHE

HKCQ

>T1MEI9_TRIUA/119-182

SDGYQWRKYGQKNIQNRKFARCYYKCMFSHDRGCRAKKTVQQQDTSGGRRPMFQVTYVNE

HKCQ

>K7V4I1_MAIZE/134-195

NDRFHWRKYGEKNILYSEYPRLYYKCGYSDDHKCPAKKYVQQQSNSYPPIFLVTLINEHT

CD

>J3M860_ORYBR/133-194

NDGYHWRKYGEKTILNTEFPKLYYRCGYSDERKCQAKKYVQQANRKHPPEFTVTLTNEHT

CN

>J3L5A9_ORYBR/122-183

KDGFLWRKYGQKNIQDSNYLRLYYKCTFSRERRCAAKKQVQQKDDGEPPMFLVTYLGEHT

CQ

>K3ZJB7_SETIT/126-188

EDGYEWRKYGEKKINGTSYTRSYFRCTYKDDTGCLATKHVQQKDCNSDPPMFQVTYNNGH

TCK

>C5Y3H4_SORBI/132-194

DDGYEWRKYGEKKINGTLFTRSYFRCTYKDDAGCLATKHVQQRDDNSDLPMFHVTYNNDH

TCN

>B4FCE6_MAIZE/44-105

DDGHQWRKYGEKKLSNSHFPRFYYRCTYKNDMKCPATKQVQQKDSSDPPLFSVTYFNQHT

CS

>K4AKU1_SETIT/30-91

NDGHQWRKYGEKKINNTNFPRYYYRCTYKDNMNCPATKQVQQKDHSDPPLYAVTYYNEHS

CN

>M0SWT7_MUSAM/131-193

DDGHSWRKYGQKDINSAKYPRSYYRCIHRKEQGCPATKTVQQEDGDAYPPQFIVAYSMQH

TCR

>M0TJN8_MUSAM/70-132

EDGYQWRKYGQKTINDAMYPRCYYKCTYRDDQGCPATKTVQQQDSYADPPTFMVEYSMKH

TCK

>M0TTG4_MUSAM/70-131

DGYQWRKYGQKTINNAKYPRSYYRCTYKEGQGCLAKKTVQQEDSCADPPSFKVEYSIQHT

CK

>I1IV03_BRADI/121-188

YDGHQWRKYGQKNINGMQHPRSYYRCTYKERSCSATKTVQKQDHNGSSFSYGDEAVNYTV

MYYGNHTC

>T1M9Q3_TRIUA/111-176

DGHHWRKYGQKKINGRQHVRSYYKCAYTERNCSATKTIQQQEHNRTLNCEDETPKYIVVY

YGHHSC

>T1LAC7_TRIUA/117-183

DGHHWRKYGQKNINGRENARSYYRCAYTERNCSATKTIQQQDQNGSLNCEEEAAKYNVVY

YGHHTCS

>A0A0D3G4T7_9ORYZ/147-208

DGHQWRKYGQKNINNSNHQRSYYRCTYKHEQNCKATKTVQQLDSAGETIMYTVVYYGQHT

CK

>K3Y972_SETIT/138-202

DGRQWRKYGQKHINKAKHPRSYYRCTYRQEQDCKATKTVQQQDDSAGTDHPVMYTVVYHG

QHTCK

>K3ZNM2_SETIT/136-202

DGHQWRKYGQKQINGAKYPRSYYRCTYGKEQGCKATKTVQQYDPVTNTASDHSIMYKAIY

YGRHTCN

>J3N5J0_ORYBR/148-214

DGHQWRKYGQKHINNSKHPRSYYRCTYRQEEKCKATKTVQQREDLNYANNGDHPIMYTMV

YYGQHTC

>T1N7Y3_TRIUA/130-195

DGHQWRKYGQKIINHTKHPRSYYKCTYKQEQDCRATKTVQQQQQDAGVDDDPAMYAVVYY

GQHTCK

>K3XKH8_SETIT/112-171

DGFIWRKYGQKEINGHKHPRLYYRCAHKQQQGCNATRRVQRTRDHPAAYEIAYYGEHTCR

>M7ZW64_TRIUA/111-170

EDGFIWRKYGQKEIHNNRHPRLYFRCTYKHDSGCPATRQVQQAEDDPSLYVITYFGDHTC

>M0YJN3_HORVV/140-204

DDGFLWTKYGQKDIRGSGHPRHYFRCAYKLDAGGCPARRQVQRSEEEEEDDPYLYVITYF

GDHTC

>K7UWT7_MAIZE/125-185

EDGFIWRKYGQKDIHGSNYPRLYFRCTYKDDRGCEARRQVQRSDTDPCAAYLITYFGEHT

C

>D7LDZ6_ARALL/104-164

DDGHCWRKYGQKEIHGSKNPRAYYRCTHRFTQDCLAVKQVQKSDTDPLVYEVKYLGNHTC

D

>M4C895_BRARP/99-159

DDGYCWRKYGQKEIHGSSNPRGYFRCTHRFTQKCPAQKQVQRSDKDPSIFEVKYVGNHTC

N

>K7KQN0_SOYBN/1276-1336

DDGYIWRKYGKRQILGAKYPREYYRCAHKFVHWCPARKRVQRLDENPATIEIIYEHRHTC

A

>M1BIZ3_SOLTU/122-182

EDGYSWRKYGQKNILGAKYPREYYRCARNRSSNCAATKMVQRSETEESLAFEVSYGENHS

C

>M1D7L1_SOLTU/128-184

EDGLNWRKYGQKLILGAKYPREYFQCVCRHCDATKMVQRIQTEPLTFEVTYGESHSC

>M1D7L8_SOLTU/129-186

EDGLSWRKYGQRLVLGEKYPRKYYRCAPCCCGATKVIRRIDTEPLTFEVTYGGSHNCD

>M7YD70_TRIUA/902-962

SDGFNWRKFGQKTIHDSNFRRDYYRCAHYRSHKCPVIKHMQRTDADPLLFQVVYRHEHTC

T

>C5X9Q6_SORBI/1354-1412

NDGYTWRKYGSKQILGSNYPRDYYKCTQRRGCPARKHMQRRDGEPILYDVCYFGEHSCD

>M4ECF8_BRARP/121-181

DDGFSWRKYGQKDILGAKFPRGYHRCTYRKSQGCEATKQVQRSNEDPMLFEIIYRGIHSC

S

>M0T4G9_MUSAM/121-181

DDGHSWRKYGQKEILGAKYPRSYFRCTHRNTVGCFAMKQVQRSDDDPSVFDITYRGEHTC

P

>M0THF1_MUSAM/136-195

EDGYSWRKYGQKEILGARHPRGYYRCSHRNSVGCLATKQVQRSDQDPCVFDITYRGEHTC

>I1KPJ1_SOYBN/130-190

EDGYNWRKYGQKDILGAKYPRSYYRCTFRSTQGCWATKQVQRSDEDPTMFDITYRGNHTC

S

>A0A3Q7HL08_SOLLC/133-193

DDGYSWRKYGQKDILRAKYPRSYYRCTYRHMQNCWATKHVQRSDDDPTVFDITYRGSHNC

H

>D7M911_ARALL/158-218

DDVFSWRKYGQKDILGAKFPRSYYRCTHRSTQNCWATKQVQRSDSDATVFEVTYRGTHTC

S

>M1BRE7_SOLTU/99-159

DDGYSWRKYGQKNILGAIHPRAYYRCTHRNTQSCLATKQVQKSEQDPLVFEVTYKGMHSC

K

>M0SP79_MUSAM/129-188

DDGYSWRKYGNKNILGSKHSRSYYRCRDRNTKSCFATKQVQRSDEDPQAFDVIYQGTHTC

>W1PMH6_AMBTC/119-178

GDRYTWRKYGQKDILGAKYPRGYYRCTHRNSQGCPATKQVQRSDDDRSIFDITYRGKHTC

>B9T8B8_RICCO/123-182

GDGYSWRKYGQKDILGAKFPRGYYRCTHRHSQGCLAIKQVQRSDENPSIFEVTYRRKHTC

>E0CUJ8_VITVI/134-194

DDGFSWRKYGQKDILGAKYPRSYYKCTHRNAQGCLATKQVQRSDDDPTIFEITYRGRHTC

T

>B9RDW9_RICCO/125-184

DDGYNWRKYGQKDILGANFPRSYYRCTHRHSQGCLATKQVQRSDQDPTIFEVNYSGKHKC

>K3ZVA0_SETIT/121-180

DDGHSWRKYGQKEILGAKYPRSYYRCTHRHSQGCAATKQVQRADEDPTLFDVIYLGAHTC

>M7YP80_TRIUA/106-165

DDGRSWRKYGQKEILGAQHPRAYYRCTYQKTQGCAATKQVQRADDDPALFDVIYHGEHTC

>C5X9Q6_SORBI/1487-1548

DDGYSWTKYGQKNILGAKHPISYYRCAHWIAQGCTATKRLHRKEDADTLGFDAIYYGQHT

CD

>M0SHM4_MUSAM/124-183

DDGYSWRKYGQKDILGSKHPRGYYRCTHRIMQGCPATKQVQRSDEDPLLFHVTYHGAHTC

>A0A3B6KLB4_WHEAT/1333-1392

EDGFSWRKYGQKDIIGSMHPRAYFRCTHRHVKGCPVTKQVQRTSTDPLLFDVVYHGEHTC

>J3NA98_ORYBR/1018-1075

DDGFSWRKYGQKPIEGAMHPRNYYRCASQGCRAAKHVQATDDNPLIVDVMYHGEHTCT

>D8R168_SELML/1-61

DDGYTWRKYGQKDILGSRHPKSYYRCTHKRESGCPAIKYVQRSDSNPSSFQITYRGEHTC

N

>D8QTC4_SELML/4-64

DDGFTWRKYGQKDILNSKFPRSYYRCTHQKELGCQATKYVQKCEDEPSMYQVTYIGEHSC

Q

>W1P9B9_AMBTC/6-65

EDSFTWRKYGQKEILNSKFPKSYYRCAHRTTGCEAKKYVQRLDNDPQIIEVTYLGRHICQ

>K7KV11_SOYBN/172-232

EDGFTWRKYGQKEILGSKFPRSYYRCTHQKLYECQAKKQVQRLDQNPNIFEVTYRGDHTC

H

>M4DKE6_BRARP/150-210

DDNHTWRKYGQKEILGSKFPRAYYRCTHQKLYNCPAKKQVQRLNDDPFTFRVTYRGSHTC

Q

>M0TC40_MUSAM/153-210

DDGYTWRKYGQKDILNSRSYYRCTHRSYYGCEAKKKVQRLDDDPNTFEVIYCGTHTCQ

>M0TXJ2_MUSAM/6-66

NDGYKWRKYGQKKILNSSFPRSYYHCNHKSYYRCDAKKMVQRSDDDPYTFQVTYCGSHTC

H

>C5X581_SORBI/15-73

HDSYTWRKYGQKEILGARFPRSYYKCGRRPGCPAKKHVQQCDADPSKLEVTYLEAHTCD

>B9S4F2_RICCO/133-192

DAHTWRKYGQKEILNAKYPRSYFRCIHKYDRGCKATKQVQKVEEDPQMYCTTYIGHHTCS

>M4DKE5_BRARP/141-201

EDIYAWRKYGQKEILNSKFPRSYFRCTHKPTQGCKATKQVQKLEQNPEMFQITYIGNHTC

T

>M4CSQ1_BRARP/127-187

DDAFSWRKYGQKEILNAIFPRSYFRCTHKFTQGCKATKQVQKLEPESKMFNITYIGNHTC

N

>K7M7W5_SOYBN/127-187

DDNHAWRKYGQKRILNSEFPRSYFRCSHKYDQGCRAIKQVQVDQENPNMLQTTYIGIHTC

N

>B0LUS3_SOYBN/136-196

DDNHAWRKYGQKEILNSQFPRSYFRCTRKFEQGCRATKQVQRIQENPDMYTITYIGFHTC

K

>K7M257_SOYBN/138-198

EDGYTWRKYGQKMTSQSKYLRSYYRCTHKNDQGCQAIKQVQRIQDNPPLYRTTYYSHHTC

K

>D7M6E1_ARALL/110-170

HDGFLWRKYGQKQIKESEYQRSYYKCAYTKDQNCEAKKQVQKIQHNPPLYSTTYFGQHTC

Q

>M4CDZ0_BRARP/106-165

DGFLWRKYGQKSIKNSKYERSYYRCSYNIDHDCGARKHEQQIKENPPVYRTTYFGHHICK

>WRK64_ARATH/103-163

DDGFTWRKYGQKTIKTSPYQRCYYRCTYAKDQNCNARKRVQMIQDNPPVYRTTYLGKHVC

K

>M4CW62_BRARP/100-160

NDGFSWRKYGQKKIKTSSHQRCYYRCAYAKDRNCNATKRVQQIQNSPSVYRTTYVGKHIC

E

>WRK66_ARATH/86-145

DGFIWRKYGQKTIKTSPHQRWYYRCAYAKDQNCDATKRVQKIQDNPPVYRNTYVGQHACE

>F6GXW4_VITVI/171-231

DDGYAWRKYGQKVILNAKHQRSYYRCTHKHDQGCMATKQVQMTEEEPPMYKTTYHGQHTC

K

>K7M259_SOYBN/132-192

EDGYAWRKYGQKITLNAKYLRSYYRCTHKYDQGCPATKQVQRTQEDPPLYRTTYYGHHNC

K

>B9SRT4_RICCO/128-188

DDGHAWRKYGQKVILNTKFPRNYFRCTHKYDQGCQATKQVQKIEEDPPKYRTTYYGNHTC

K

>M1CUN0_SOLTU/22-82

DDGHAWRKYGQKQILNSTYPRHYFRCTHKYDQKCQASKQVQKIQDNPQRFRTTYYGHHTC

K

>K7MJ24_SOYBN/105-164

DGHQWRKYGQKEILNAKYSRNYYRCTHKYDQNCQAIKQVQRIQEDPPLYKTTYLGHHTCN

>I1L6Z7_SOYBN/121-181

DDGHHWRKYGQKEILNAKFPRNYYRCTHKFDQGCQATKQVQRVQEEPILFKTTYYGHHTC

K

>M1CMZ7_SOLTU/94-155

EDGHAWRKYGQKVILNSKYPRCYYRCSHKYDQECYATKQVERIKEEEPIIYRTTYFGHHV

CK

>WRK70_SOLLC/116-177

NDGCAWRKYGQKKILNSKYPRCYYRCTHKYDQECRATKQVQIIQENPIIMYHTTYFGNHT

CN

>F6HC33_VITVI/111-171

EDGHAWRKYGQKEILNAKFPRSYYRCTRKHEQSCRATKQVQRMKENPIMYHTTYIGHHTC

R

>W1PB05_AMBTC/141-201

DDGYAWRKYGQKDILNANHPRSYFRCTHKNDQGCAALKQVQRMDSNQSFFEVTYIGYHTC

R

>M0SWH8_MUSAM/162-222

DDGQTWRKYGQKEIQSAKHPRSYFRCTHKYDQGCMAHRQAQLSEDDPTDFVITYIGEHTC

R

>I1HPN1_BRADI/101-161

EDGQSWRKYGQKDIQNSEHPKSYFRCTHKYDQKCAALRQVQRCDQDPESFVVTYIGQHTC

Q

>K3Z832_SETIT/111-171

EDGHAWRKYGQKEIQNSKHPKAYFRCTHKYDQQCAAQRQVQRCDDDPDAFRVTYIGVHTC

R

>J3MP57_ORYBR/126-186

EDGKQWRKYGQKHIQDSPNPRSYYRCTHKPDQGCMATKQVQASETNPSEFVISYYGEHTC

R

>M8A8S6_TRIUA/118-178

TDGKSWRKYGQKQINDSTNPRSYYRCTHLPDQGCKAKRHVHVSEANPSEYTIDYYGQHTC

R

>C5X5M4_SORBI/126-186

DDGKAWRKYGQKRIHESPNPRSYYRCTHRPEQRCMATRQVQASDANPSEFIISYYGQHTC

Q

>K4ACF8_SETIT/124-184

EDGHVWRKYGQKEIQNSSYPRSYYRCTHRSDQGCNAKRQVQVCEADPSKFVVTYYGDHTC

R

>J3LNI8_ORYBR/48-108

DDGHVWRKYGQKDIQNSPHPRSYYRCTHKVDQGCTATRHIQRCENDPSNYVITYYGEHTC

R

>I1BSM4_RHIO9/209-264

MDGYFWKNNGNTVQKKTGNKSVYYKCSNSNKGCPVNKTVTWKGNGEYLIKYRGEHL

>C1EBM1_MICCC/10-63

EDGYVWHKYGAKNVRGRKVGYFKCAHRGCEARKKVWRQANGDEAVEREGTHTHA

>M0SJA2_MUSAM/36-92

DGYAWRKYGQKFILKIRKNRSYFKCREEGCKAKKRVEWPPSDPSNVKVTYDGVHHHP

>U5GSI9_POPTR/36-91

EDGFEWKKYGQKFIKNIGKFRGYFRCQKRNCMAKKRAEWSNPENLRIVYEGSHSHA

>W1NEF1_AMBTC/8-65

EDGFVWRKYGQKFIRNIRKNRSYFKCQKKSCGARKRVEWCNSDPQNLRVIYDGSHSHP

>C1MHM4_MICPC/67-120

DDGYRWRKYGQKLIKGSPFPRSYYKCTSENSSMQKHVEQSADNPKLYVVTYHSD

>C1N120_MICPC/5-60

DDGYRWRKYGQKIIKGAPFPRSYYRCTSANCPARKHVEGDPSLLSSLTYEGEHNHE

>J3LCQ1_ORYBR/41-98

ADGYSWRKYGQKPIKGSPYPRGYYKCSTAHGCPARKHVERDPGEPAMLIVTYDGDHRH

>W1NXJ2_AMBTC/234-290

SDNYSWKKYSERTVKGSPYLRLFYKCATAEGCPAKKQVEWCKDNINMVTITYEGFHM

>M0T0B0_MUSAM/207-264

DDFSWRKYGQKPIKGSPHPRSYYKCSSMRECPARKHVERCLDDANMLIVTYEGYHSHP

>B9T4U8_RICCO/34-91

DDYSWRKYGQKPIKGSPHPRGYYKCSSVRGCPARKHVERASDDPSMLVVTYEGEHNHT

>D7M935_ARALL/134-192

SDAWGWRKYGQKPIKGSPYPRSYYRCSSSKGCLARKQVERNPQNPEKFTITYTNEHNHE

>M1DN19_SOLTU/86-144

DDKWAWRKYGQKSIKGSPFPRNYFKCSTSGSCKATKIIEKSPKNENYFLVSYSDEHNHD

>M1DBE7_SOLTU/170-228

NDIWTWRKYGQKHIKGSPFPRNYYKCSTSKHCEAKKQIEKSPKDENIFLVSYSGEHNHD

>D7MUR9_ARALL/1173-1233

ADLWTWRKYGQKYILGSNFPRSYYRCTYRFTQGCLATKQVQRSDTNSNMFAITYISEHNH

P

>M1DVX5_SOLTU/167-225

ADQWRWRKYGMKRTGDSTFLKSYYRCNEANDCPARRHIQKSSTDPNKVIVTYRGQHNHP

>F4QAR0_CAVFA/125-181

DEYHWLLNTIKFVNNSKFLCQNYTCFEKECKATKNVQKLSPNNNNFRVTYINTHNHP

>F4Q0C8_CAVFA/619-677

YSWNRMGSTKADGINFTKYQYNCSVTDCECIKLVQHYPINHDNNRYIELIIYQGVHNHP

>M0RX59_MUSAM/130-188

SDLWAWRKYGQKPIKGSPYPRSYYRCSTSKGCQARKQVEQSRADPGMLLITYTAEHNHP

>B9RND6_RICCO/52-110

SDFWSWRKYGQKPIKGSPYPRGYYRCSTSKGCSAKKQVERCRTDASVLIITYTSNHNHP

>M0TU82_MUSAM/44-102

SDSWAWRKYGQKPIKGSPHPRGYYRCSSWKGCPARKQVERSRLDPATLVVSYSFEHNHP

>A0A1D6LTJ8_MAIZE/134-191

DPWAWRKYGQKPIKGSPYPRGYYRCSTDKDCRARKQVERCRTDPSTVIVSYTGEHSHP

>I1MKR8_SOYBN/158-216

SDIWAWRKYGQKPIKGSPYPRGYYRCSSSKGCLARKQVERNRSDPAMFIVTYTGEHNHP

>I1LDL1_SOYBN/180-238

ADLWAWRKYGQKPIKGSPYPRNYYRCSSSKGCMARKQVERSNTEPDMFVVTYTGDHSHP

>D7MKX1_ARALL/1146-1204

ADQWVWRKYGQKPIKGSPYPRNYYKCTSSKGCSARKQVERSRTDPNMLVITYISEHNHP

>E0CUS7_VITVI/11-69

RDSWSWRKYGQKPIKGSPYPRNYYRCSTLKACSARKQVELSQDNPEEYIVSYIGDHIHA

>I1HG21_BRADI/167-225

DDLWAWRKYGQKPIKGSPYPRGYYKCSSLKACAARKLVERSPDKPEVLIVTYIADHCHA

>A0A3N7FWR6_POPTR/157-215

SDMWAWRKYGQKPIKGSPYPRSYYRCSSLKGCLARKQVERSSTDPSIFIITYTAEHSHA

>M4CLX3_BRARP/328-385

EDGFKWRKYGQKAVGGNAYPRSYYRCTSVNCRARKRVERANDDPKAFITTYEGKHNHH

>D3BE88_POLPP/1056-1111

DDGFNWRKYGQKAVKGSPYPKSYFKCAEHGCNVKKQVIQQGDKKFVNTYNGRHTHD

>WRKY1_DICDI/1111-1165

DDGFFWRKYGQKSVKGSPFPKSYFKCAELTCPVKKQVIQQDSKYINTYRGKHNHD

>F4PLI3_CAVFA/941-996

DDGFNWRKYGQKAVKGTHFPKSYFKCAEPGCSVKKQVLQQSENSFLSTYNGNHNHD

>M0THP3_MUSAM/86-142

DDGYKWRKYGQKSIKNSPNPRSYYRCTNPRCNAKKQVERSLEDPETLIVTYEGLHLH

>D7KEM5_ARALL/135-192

EDGYRWRKYGQKAVKNSPYPRSYYRCTTQKCNVKKRVERSFQDPSIVITTYEGKHNHP

>WRK68_ARATH/118-175

DDGYKWRKYGQKPVKDSPFPRNYYRCTTTWCDVKKRVERSFSDPSSVITTYEGQHTHP

>M0TPC7_MUSAM/150-207

DDGYRWRKYGQKAVKNSRFPRSYYRCTSATCGVKKRVERSSDDPAVVVTTYEGQHNHP

>K3XLT9_SETIT/160-217

DDGYRWRKYGKKLVKNSPNPRNYYRCSSAGCGVKKRVERARDDESFVITTYDGVHNHP

>WRK50_ARATH/113-170

DDGFKWRKYGKKMVKNSPHPRNYYKCSVDGCPVKKRVERDRDDPSFVITTYEGSHNHS

>I1HL63_BRADI/95-152

EDGFKWRKYGKKAVKNSPNPRNYYRCSAERCGVKKRVERDRDDPRFVVTTYDGVHNHA

>M0TK84_MUSAM/89-146

DDGYSWRKYGKKMMKNSQNPRNYYHCSSEGCNVKKRVERESEDSRFVITTYEGIHNHR

>M0SMA5_MUSAM/88-145

DDGYKWRKYGRKKMKNSPYPRNYYRCSTVGCNVKKTVEREREDSRFVLTTYEGTHNHH

>F6H4B4_VITVI/93-150

DDGFKWRKYGKKSVKNSPNPRNYYKCASGGCNVKKRVERDREDSSYVITTYEGVHNHE

>M4F9N3_BRARP/76-133

DDGYKWRKYGKKSVKNNINKRNYYKCSSEGCMVKKRIERDGKDAAYVITTYEGVHNHE

>M1D2T0_SOLTU/151-208

DDGYKWRKYGKKKVKTNIYLRNYYKCSSGDCKVKKKVERDGNDSRYLMTTYEGKHNHE

>M1D6V7_SOLTU/22-80

DDGYKWRKYGKKTVKSNTNYLRNYYKCSIRGCEVKKRVERDGHDSSNLITTYEGKHNHE

>W1P843_AMBTC/109-166

DDGYRWRKYGKKSVKNSPNPRNYYRCSNGGCPVKKKVERDAKDSAFVITSYEGRHNHT

>B9SM87_RICCO/108-165

DDGYRWRKYGKKAVKNSRNPRNYFKCLKAGCNVKKTVQRDTEDPDYVTTTYEGMHNHE

>I1KBC5_SOYBN/112-169

DDGYKWRKYGKKTVKSSPNPRNYYKCSGEGCDVKKRVERDRDDSNYVLTTYDGVHNHQ

>Q6IES4_ORYSJ/135-192

DDGYKWRKYGKKSVKNSPNPRNYYRCSTEGCNVKKRVERDKDDPSYVVTTYEGTHNHV

>WRK59_ARATH/109-166

DDGYKWRKYGKKPITGSPFPRHYHKCSSPDCNVKKKIERDTNNPDYILTTYEGRHNHP

>M4CXB1_BRARP/69-126

DDGYRWRKYGQKAVKNNKFPRSYYRCTHGGCNVKKQVQRLTSDQEVVVTTYEGVHSHP

>B9RAC9_RICCO/80-137

DDGYRWRKYGQKTVKNSKFPRSYYKCTHNGCSVKKQVQRKSEEEEVVVTTYEGKHTHS

>WRK45_ARATH/65-122

DDGYRWRKYGQKAVKNNPFPRSYYKCTEEGCRVKKQVQRQWGDEGVVVTTYQGVHTHA

>K7KVJ1_SOYBN/74-131

DDGYQWRKYGKKIVKNNKFPRSYYRCSHQDCNVKKQIQRHSRDEQIVVTTYEGTHTHP

>M0RX59_MUSAM/291-348

DDGYRWRKYGQKAVKNSVHPRSYYRCTHHTCNVKKQVQRLSKDTSIVVTTYEGIHNHP

>B9T726_RICCO/27-84

DDGYKWRKYGQKVVKNTLHPRSYYRCTQDNCRVKKRVERLAEDPRMVITTYEGRHAHS

>D8R033_SELML/2-60

NDGCQWRKYGQKMAKGNPCPRAYYRCTMSPGCPVRKQVQRCAEDTSILVTTYEGTHNHP

>K7LCG4_SOYBN/201-259

GDGCQWRKYGQKTAKGNPCPRAYYRCSMGTACPVRKHVQRCFKDETILITTYEGNHNHP

>M4CUI9_BRARP/208-267

NDGCQWRKYGQKTAKTNPLPRAYYRCSMSSNCPVRKQVQRCGEDDTSAYMTTYEGNHDHP

>M1A9L5_SOLTU/207-265

NDGCQWRKYGQKISRGNPCPRSYYRCSVAPLCPVRKQVQRCLEDMSILITTYEGTHNHS

>U5D8Z1_AMBTC/112-170

RDGCQWRKYGQKVTRDNPSPRAYYRCSMAPGCPVKKKVQRRADDSSILVAAYEGVHTHP

>I1MX45_SOYBN/109-166

KDGYQWRKYGQKVTKDNASPRAYFRCYMAPICPAKKKVQRCLHDKSILVAIYDGEHSH

>F6H3I5_VITVI/96-154

KDGFQWRKYGQKITKDNPSPRAYFRCSMAPQCPVKKKVQRCMEDSSILVATYEGAHNHE

>C5XPF8_SORBI/150-208

NDGCQWRKYGQKVAKGNPCPRAYYRCTGAPDCPVRKKVQRCAHDAAVLVTTYDGAHNHP

>M0S0M4_MUSAM/331-387

DGYHWRKYGQKVVKGNTFPRSYYRCTNPKCPVRKYVERSSEDSAHLVTTYEGRHNHE

>I1IVB4_BRADI/275-329

EDAFRWRKYGQKAVNGNLFPRSYYRCSTARCNARKFVERSSDNSLVTTYEGRHNH
