# Supplementary material for: A systematical genome-wide analysis and screening of WRKY transcription factor family engaged in abiotic stress response in sweetpotato
Source: BMC Plant Biol. 2022 Dec 28;22:616. doi: 10.1186/s12870-022-03970-6 (PMC9795774; doi:10.1186/s12870-022-03970-6)
Supplement: Supplementary file 13 — Additional file 13. [file 12870_2022_3970_MOESM13_ESM.docx]

**Additional file 12**. The nucleotide and amino acid sequences of 84 IbWRKYs identified in sweetpotato genome.

Nucleotide sequences

>IbWRKY1

ATGGCTGGTTTTGGTGACCATATGCCAATAATGGGAGATTGGGTGCCTCCTAGTCCAAGCCCGAGGGCATTTTTCTCTTCGGTTTTGGGCAATGATATTGAGTCAAGATCAAATGCCAGTCAGCCCCTCCTTCCAGATCCTAAAGAGTATGCTTCATCAGGGAATTCGGATAGTAAAAATGTGGCGCAAGGCAGTGATCAAACGCCTAAATTAAGTTCACTGTCTGAGCGGAATATGAACTCTCATGGAGGTCTGTTGGAAAGAATGGCAGCTAGAGCTGGATTCAGTGCCCCGAAGCTGAAAACGGATAGCATTCGACCTCCTGCTCTTGTTCAGAACCAAGAACTTAGGTCTCCTTACTTCACAATTCCACCGGGTCTTAGTCCAACAACCCTGCTAGATTCCCCCGTTTTCCTCTCGAATTCTCTGGTCCAATCATCTCCAACAACTGGAAAATTTGCATTTCCCTCAATTGGTGACAGTCGAAACTCAGCATTGTTTATGGGGGCTTCTGATAACAACAAAGAGACTTCTTTTAACAACAATGACGCCTCATCCTTTGCTTTCAAGCCAGTTATAGAAACTGGTCCTTCCCTTTTTCCCGAGACAATTAGCAAAGTACCTCCATCCAATCTATCTTGGCAATCCGTTCCAGGTATTGAGGTTTCAGTCCATTCTGAGAACCCTCGTGTGCATCAACATGCTGAGCCTACTCTGGTTCACACTCAGAGTGGAACACTTGAACAATCCGTGTTCTCGAGATCATATACTGAAGGGGTTAGTAATATCATATCAGAGCCGAGGACCTTTCAAGCAGTTGCTGGCAGTATGGAGCACTCTCCCCCACCCGATGAACAGCAGGATGAGGAAATAGATCAACGAGGAGGCGGTGATCCCAATGCTGTTGGTGCTCCTGCTGATGATGGCTATAACTGGAGAAAATACGGGCAGAAACAGGTCAAAGGAAGTGAGTATCCACGGAGTTACTACAAATGCACACATCCAAATTGCCAAGTTAAGAAGAAAGTGGAGCGGTCCCATGAGGGTCATATTACAGAGATTATCTACAAGGGAACTCACAGCCACCAAAAACCTCCACCTAATCGCAGGGCAGCATTTGGATCTGAAAATGCAGAACAAGATGGAACTATTGGCATTGGTGATCCTATTTGGGAAAATGTGCAAAATGGATCTGGTGCTGGAGGTCCTGATTGGGGGAATGACAATCTTGAGGTAACATCTTCAGGCACTCAGTTTGAACCTAGGGATCCCGTGGAGGGGTCTTCTCCTTTTTCAAATGAAGAAGATGAAGATGATCGTGGAACACATGGCAGCGTATCACTCCCCGAGGGTGAAGGAGATGAGTCTGACTCAAAGAGAAGGAAGATTGAAACTTATGCAGCTGACATGAGTGGGGCCACGAGAGCCATTAGGGAACCCAGGGTGGTGGTGCAGACTACCAGTGAGGTTGACATACTTGACGATGGGAGTTATTACAAGTGCACAAGTGCTGGCTGCACTGTTAGGAAGCACGTGGAGAGAGCTTCACATGATCTGAAATCGGTGATTACCACCTATGAAGGGAAGCACAACCATGATGTTCCTGCAGCTCGCAACAGCAGTCATCCTAATTCAGGGGCTTCCAACTGTCTGTCCTCACAAACGACTGCTACTCAAGGTCACGTGCATAGGCCCGAGCCTTCTCAGCTTCAGAACACCATGTCACAGTTTGCTAGGCCTCCTTCACTGGGCTCATTTGGTTTTCCTGGTGGGCCACAGCTCGGCCACACCCTAGGCTTTGGTTTTGGGATGAACCAGCAAGGCCTTGCCAATCTGGCAATGGCTGGATTAGGACCTAACCAAGGCAAATTTCCAGTTCCTCCGGTTCATTCTTATCTTGGACAACAACACCCAATGAATGACATGCGACCCAAAGCAGAACCCAAGATGGAGCCTAGCTCAGATCCTGGCCTGAATCTCTCTAATGACTCCTCAGTCTATCAACAATTCACCAGTAGACTGCCTCTTGGACCTCAAATGTAA

>IbWRKY2

ATGGACGCTCATCACCTTCTTCATCATCCGACAGTGATTCTCAACTCCCTCGACGATCCTACGTTGACGGCGCCGGCGCCGGCGTTCCGGCCCGAGAAAAGGGCCGTTAACGAACTAGATTTCTTCAAGAGAGAGAATTCTGATCTTGATTCCGCCATGGACGAAGCTCTGGTTAGTAAAGGGAATGGCCGCCGTGTCGGAGATGAAGCTGTGAACCATCCCCCAGTACTTGATACTGGCTTAGATCTTCTGGGTTCAAGCAAGAAGTCCATGGTCTTCCATGGCGCTTCGCCACCAGCAACAATGGAACATAAAGCAACGGTTGAAGAAGAAAGAAGTTTATATCTTACAGCTTTGAGGGAAGAGCTGGAAAGGATGAACTCGGAAAATCAACGTTTGAAATCAATGCTTAATCAAGTCCACGAAAAATATAACGCTTTGAAGATGCACTATGCATACATCTTAGAACATCAACACACCCTAAAACCTGAAATCCCGGAAGACAATAAGATGAACGATGGATTCGCAGAAGGAAATGAGAGGAAAAGAAAAGTGACAGATGATATGAAGGAAGAGCATTCACATTCGTCGCCGGAAGGCGCCGGCGCGTCACCGCCATGTCCGGAAGATAACACCAGAGAAGAAAGCCCCGACAAGGTACAACAAAAGCTTGTCAGATCCAACGGTGAACATTCTGATCATCTTCTTCCAGCTGCCGCCGATCAACACGCGCCGGCGGCGAAGAAGGCACGCGTCTCCGTCCGCATACCTTGCGACACACCTTATTGTTCTGATGGATGCCAATGGAGAAAATACGGTCAAAAGATGTCCAAAGGAAACCCGTGCCCTCGAGCTTATTATCGTTGCACCATGACCTCTACTTGTCCCGTCCGCAAGCAGATTCAAAGGTGTGCCGAAGACCGTTCGGTTATGATAGTTACCTACGAAGGTGAGCACAACCACCCACTGCCGCCGGCCGCCAGGCCAATGGCATCCACTACGTCGGCGGCGGCCACAATGCTACTCTCCGGCGCCGCTCGGAGTGCGGACGGCGCGGGACGTCCGGCGAATCTCGACGCCCTCCCAGCAAATTTCCTCCCAACAATATCAACTTTCGCACCAGTCCCTACCATCACCCTGGACCTCACCAACCCCATGGCTACACAGCCGCAAACGCCTCCGCCGTTCCATTCCCCAAATCCTCCGCTCCCTCCCGGCATCGTAAGCGCCGCCGCGGCTGCCCTGACGAGAAATCCTAGTTTTACGGCGGCTTTGGTTTCCGCCATCGCCTCCATCATTGGCGGCAACAATATTGCAGCTCAGCCGCCGCCGCCGCCTAACAGTGCTGAGCTCAGTGACCAAGACGTTAAGCCGTCTCCCAGCATTGAAAGCAACGTGCAAGCAAAAGTTTTATAG

>IbWRKY3

ATGGATGGTGAAAAGAATGATTGGGATCTTGGTGCAGTGGTGAGAGGCTGCAAAAACCTCAATGGCAATTCAAGTAGCCAAGATGTTAATGAACATTTCAATGGTGGTTTTGCTAGCCAAACCTATGTCTCAATCCCTTTACTCCCTCAACCAAGCCATTATTATAGTAGTGTTTCTCCTAGCATAGGGACCGAAAGGCGCTATTTCGGGTTAGAAGAGGTTATTGATAGGTTTACTAATGGAAAGGTGAGAGAACTAGCCCTAGATCTTCAGGCTACCATCAACCCCACTTGTCTTGACAAGGCTGATGGCTCGGGAGGAATGGGGAAGGACACCCCAGCCCTTCAAAGTCCCGACCCTTATTTCGGACTAGAAGAGGTTATTGATAGGTTTACTGGTGGAAAGATGAGAGAACCAATCTTAGATCTTAAGGCTACCATCACTCGCCCTACTAGTCTTGACAAGGCTGGTGGCTCGGGCTTGGGGGGTATCGGGAAGGACACCCGAGCTCCTCAAAGTCCCAATCCTTCCCCTACTTTGGAGCCTCAACAGCCTCTTCCAAGTTCCCAAACTAGTAGTCCCGAAAAAGATGGTGGCTTGGAGGATGAGAATCTTCCCCACCAGGTGGAGGAAGTGAATGAGGTTGAAATGTCGGTTGTGAAGGTTAAAGTGCCGGTTGAGAAGGTTCAAGTGCCGGCTGAGAAGGTGGACGAATGGGATGGATGGGAATGGAGGAAGTATGGGACGAAGATGATGAATGATTCACCACACTCAAAGGGCTATTACAGATGCAACCATGAGGAGAAAAAGTGCCCGGCAAAGAAGCATGTTCAGCTAAGCTACATGGATGAAAGCACGTATATCATTACGTACAAGGGTAATCACAATCACCCTCCCCCCGTTCAGACCACTACATCCAAACGTAAGAAGAGGAGCTGGGCTCGGGCTCGACCTCCCGCTCCTCCTGCTTCCAAAGGGGAAGGATCATTTTCTACTCCTAGTACTAATACCGCTACCTGA

>IbWRKY4

ATGGACAATATCGGCGGCGACCGGGAATTTTTGCAGAGCCTGATAAGTGAGCTGGCGAATGGGAGGGACGCTGCTACGCATCTCCAGATGATCCTTAATGCGCCATCATCGTTTTCTCCGGAGACTCGCGAGTTGTTGGTTCATAATGTCCTGGCTTCCTACGACAGGGCGCTCGGCATGCTCAACTATTCGCCGGAGAGCGCTGCGGTCCAGCCGCCGGCGGCTGGACCGGCATTGGGAATTGAATCTCCGTCGTCTTTTACCGGCAGTCCTCATAGCGAGGATTCTGACCGCGAGTATGATGGGTCTCGGAGGAGAAATGCACCGCGTTGGACGCAAAAGGTTCAGGTTTGTCCCGGGTCGGGGCTTGAAGGGCATCTTGATGATGGGTATAGTTGGAGAAAGTATGGGCAGAAAGACATTCTTGGAGCAAGATATCCCAGAGGCTATTATAGATGCACTCATAGGCATGCCCAGGGCTGTTTGGCTACCAAACAGGTTCAAAGATCCGACGAGGATCCGACCATTTTCGACATCACTTACCGTGGAAGGCACACCTGCAACCAGGCCGGCGGCAACCCAAACCCGCCGCAAAATCAAGAACCAAACATGCCGGAGAGCCAGCGGAATATTATCCCGTTCACCCAACAACAAGGCCAGCCATCGGAAACTCTCTTAAGTTTCCGCAGACAACTCAGAGTTGAAACCAGCGATTTAGACAACACCCACCAAGACAATCATCAGCAGTTCACTTATTTATACACCTTCCCATCAAGCTCCGACCAGAGCTACACTTTCCGGCCGCCGTCGGCGCCGGACGCCAGCAACTTTTCATGGAGCATTTCGCCTTCCTTCGGCTCCCCTGCTTCCACCACCGCGTCCAGCGACTTCGGCCGTCGTCCCCGGCCGACAACGGAAGCTCCGCCGACAAGCACTACCAACTCCGGCGGGATTATTCCGGCTGTTTCCGCCGGAAATTCCCCCGCCGTTGACCCGGATTCGCTTTTGGCACTATGGGTTTTGACTCAAACTTCACCTTTGATAGCCATGGATTCTTTCCAAACCCCTGAAAATTGA

>IbWRKY5

ATGGCAGTGGACCTTATGATGGATTACCGACACGGCGGAAACGGCAGGAGTGTTGATCTCACCTTCGCGAAGAAGCTGGAGGAGAGTGCGGTGGTGCAGGAGGCGGCGTCGGGGCTTGAAAGCGTTCATACGTTCATCAGATTGTTGTCGCAGCAGAAGCATAAGGCGGCGGAGAGCCGGGGGAAATCGACGGTGGAGATTGAAATGGTGGCTGATGTGGCGGTTAATAAGTTTCAGAAGGTGATTAATCTTCTCGGTCGGACCCGGACGGGTCACGCCCGGTTCCGGCGAGCCCCCGTCGTTTCTTCTTTGCCGGTTCCGGCGAAGGTCGACACCAAGGTTTATAACCCTACGCCGATTCAGCAAGTTCCGCCTCCGGTTTCCGCCGCCGCCGCCGGGAAGACGATTAGTTTCTCGTACTCGCCGGAGGTTTCGCGGGCTAATTCTTTCAATATATCGTCGTTAACCGGGGAGACGGAGAGCAAACAGGCGTCTTCTTCCTCTGCTTTCCAAATTACCAATCTGTCCCTGGCCTCCTCCGGCGGAAAACCGCCTCTTTCTTCGTCTTCGTTGAAGAGAAAGTGTAGCTCCTCCGAGAATAACCTCTCCGGCAAGTGCAGCGGCGGCTCCTCCGGGCGATGCCACTGTTCCAAGAGAAAGAAGTTAAGACAGAAGACGGTGACCAGAGTTCCGGCAATAAGCATGAAGATGGCCGATATTCCACCTGACGATTTCTCATGGAGGAAATATGGACAGAAACCCATTAAAGGCTCTCCACACCCTAGGGGATATTACAAGTGCAGCAGCGTGAGAGGGTGTCCGGCACGTAAACATGTGGAGAGGGCTGTGGATGATCCGGCGATGCTGATCGTCACATATGAAGGAGAGCACAATCATTCCCTCTCTGTAGCTGAAACAAACAGTTTAATCTTAGAGTCTTCTTAG

>IbWRKY6

ATGAGCGAAATCACAGAGAGAGGTTCAATCGTTGCTGGGCCTTCGCGCCCTACTGCGCTTGCAATTGCACCGCGACCGCCATTGGAGAGTTTCTTCAACGATGGGTTCATACCCGGGTTCAGCCCGGGTCCGATGACCCTTGTGTCCGGTTTCTTCGCCGATTCCGATGGGTGCTCTTTCTCCCAGTTGCTGGCCGGAGCTATGGCTTCCCCACTGGCGAAACCTAGTGTTTTGGAGGATAGCTCAGCGAAGAAGGGGAGTTCTGGGGCGGGGAGTGAGAAGCAATCCGGGTATAAGCAGAATCGGCCGGTGAGCTTGGCGGTGGCTGCTCTGTCGCCGTTGTTAGTTGTCCCGCCGGGGTTAAGTCCTTCCGGGTTGCTCAATTCGCCCGGGTTCCTCTCTCCGATTCAGTGGCCTGTTGTCCTTCTTGGTTTGAGTAGGTCATGGGTCAAGGCGATTTACCTCCTTGTCCTTATGCTCTTTTGCTGGGGTGGGGTTTACCGGTATACACTCTCGGATAGTGCTCCTTTGCTGGGGTGGGGTTTATTGGTAACATCCTCGTACGGTGACTATGGGTTTCCCTCGTCACTCAAGAAAAATGCAGGCTGTAAGCATGTGTTCTTCATTTGCTATAGCCCTTTTGGGATGTCTCACCAACAAGCACTGGCGCATGTTACAGCTCAGGCAGCGATAAACCAGTCTTACAGGCAAATGCAAACCGAATATCAGCAGCAATCCTCACCTGCAGATGCGTTTGAGCATGAATCATCTTTGATGCCAAATGATTCGTTCCAATTTCAAGTTGATGACATGTTAGACGTGGAGAGTTTGAAGAGTGAGCCAGTAGAGGTCTCGCAATCGCTGAGAAAACCTGCTCCCGGTGTCCTGGAGAGGCCTGCTAGAGATGGTTATAACTGGAGGAAATATGGACAGAAGCTGGTTAAGGGAAGTGACTGTCCGCGAAGTTACTATAGATGCACGCACCTCAAATGTCCTGTCAAAAAGAAAGTTGAACGATCCGTTGGTGGGCACATAACTGAGATCACATATAAAGGGCAGCACAACCACGAACTTCCTAACCCTAATAAAAGAAGGAAAGATGAGTGTGATCTGGACGGTGGAGAGAACATCCAAGTGAATTCTGAAATTGCTTCTCATAGTTGGACTGAAATGAACACATCAAACGAGGCTGAGTTTTCTGAGTCTGCCCAGTTACCCACCAAATTGCCATCTGAACAACTCGATGTGGGATGCGACCTCGATGAAATGGAGGAGACTGCAATGGCATTAGATGAGGATGATGGACAGAATCCTAAGAAAAGGAGCTTAGAGGTTGTGTCATCTGTTATGCCTTCATCTCATAAAACAGTCACTGAACCCAGAATCATAGTGCAGACGAGAAGTGATGTTGACCTTTTGGATGATGGGTACAAGTGGCGAAAATATGGGCAGAAAGTAGTAAAGGGGAATGCTAATCCAAGGAGTTATTATAGATGTACATACAGTGGATGCAGTGTTCGCAAACACGTCGAAAGGGCTTCAACAGATCCCAAAGCAGTTATAACTACATATGAAGGCAAACACAATCACGATATCCCTAATGGGCGAAACAGCAACCGCAGCCAAACTAACGCTAATGTTCTACAGCTGAAGCAACAAAGCACGCTCGCAGTTAACAGCTGA

>IbWRKY7

ATGTCCGACATGAACCCTAATTTCCTAGGACCAAACATGATGTCCAACCCTCCATCATATACAAACCCTAATAGTAATAATCTCCATTTTTACCCTGAAATGCTCCAAGATTATGACTATGATTATGCCCAAGATTTCGACCTTTCCTACGTTAACACCCTCCTCAATGATGATCATCACTACTCCAATGTTGTTAGTAACAACCCCTTTGCTTCTTCTTCTACTTCTTCTTCTTCATCTCTTTTCTCGCACCCACCACCTCCCCCCACCATTGTGCAAGAGAAGAGTAGCACTACTTCCGCAAGCTCTGGCAGCTCTGGCTTTGATGGAATGATGCCCACAACTTATCCCATGCAAGAAGTGAACTCCATGAGGCAGATGGTGAATACAACAAAGGCCAAGGAGAGGCATTCAATTGCTTTTAGGACGAAGACTGAGCTTGAGATATTGGATGATGGATACAAGTGGAGGAAGTATGGGAAGAAAAAAGTTAAAAGTAACTCCAATCCAAGGTTCGATGTCTTCTCCAAACACAATCTCATCAATATTTCCAAAGAAAGCAGTTTGCAGTTGAAGCTGATTTCCATGACAAGTAGCTACCCCTCTGCTTTTTTGTGA

>IbWRKY8

ATGTTCAATAACATGAACCCTAATTTCCTAGGACCAAACATGATGTCCACCCCTCCATCATATACAAACCCTAATAGTAATAATCTCCATTTCTACCCTGAAATGCTCCAAGATTATGACTATGATTATGCCAAGATTTCGACCTTTCCTACGTTAACAACCTCCTCAATGATGATGATGATCATCATCTATATCATTACTCCAATGTTGTTAATAGCCCTTTGCTCCTCCTCTTCTTCTTCTTCTTCTTCTTCATCTCTTTTCTCACAGCCACTACTACCTCCCCCAACCATTGTGCAAGACAATAGTAGCACTACTTCTGCAAGCTCTGGCTTTGATGGAATGCTGCCCACAACTACTTATCCCATGCAAGAAGTGAAGTCCATGAGGCAGATGGTGGTGAATAGAACAAAGACCAAGGAGAGGCATTCAATTGCTTTTAGGACGAAGACTGAGCTTGAGATGTTGGATGATGGATACAAGTGGAGGAAGTATGGGAAGAAAAAAGTTAAAAGTAACTCCAATCCAAGAAATAACCCGGTAATGACCCAAAAGCAAGACCCAAAATGCCCCAAGCCAGAGAGCCGAGGAGGCGAGGAGCCAGGTAATCAAGGTCAAGGCTAA

>IbWRKY9

ATGTCCGACATGAACCCTAATTTCCTAGGACCAAACATGATGTCCAACCCTCCATCATATACAAACCCTAATAGTAATAATCTCCATTTTTACCCTGAAATGCTCCAAGATTATGATTATGACTATGATTATGCCCAAGATTTCGACCTTTCCTATGTTAACACCCTCCTCAATGATGATCATCATCATCACTACTCCAATGTTGTCAATAACCCCTTTGCTTCTTCTTCTACTTCTTCTTCTTCATCTCTTTTCTCGCACCCACCACCTCCCCCAACCATTGTGCAAGACAAGAGTAGCACTACTTCCGCAAGCTCTGGTAGCTCTGGCTTTGATGGAATGCTGCCCACAACTACTTATCCCATGCAAGAAGTGAACTCCATGAGGCAGATGGTGAATACAACAAAGGCCAAGGAGAGGCATTCAATTGCTTTTAGGACGAAGACTGAGCTTGAGATGTTGGATGATGGATATAAGTGGAGGAAGTATGGGAAGAAAAAAGTTAAAAGTAACTCCAATCCAAGAAAGCAGTTTGCAGTTGAAGCTGATTTCCATGACAAGTGCTACCCTCTGCTTTTGTGA

>IbWRKY10

ATGCTCCAAGATTATGACTATGATTATGCCCAAGATTTCGATCTTTCCTACGTTAACACCCTCCTCAATGATGATGATCATCATCACAACTCCAATGTTGTTAATAACCCCTTTGCTTCATCCTCTTCTTCTTCTTCTTCTTCATCTCTTTTCTCACAGCCACTACTACCTCCCCCAACCATTGTGCAAGACAAGAGTAGCACTACTTCCGCAAGCTCTGGCTTTGATGGAATGCTGCCCACAAATACTTATCCCATGCAAGAAGTGAAGTCCATGAGGCAGATGGTGGTGAATAGAACAAAGACCAAGAAGAGGCATTCAATTGCTTTTAGGACGAAGACTGAGCTTGAGATATTGGATGATGGATACAAGTGGAGGAAGTATGGGAAGAAAAAAGTTAAAAGCAACTCCAATCCAAGGAATTACTACAAGTGCTCACATGAAGGGTGCATAGTGAAGAAGAGGGTGGAAAGAGATGGAGAGGATTCGAAGTTTTTGATAACAGAATACGAAGGAATACACAACCACGAGAGTCCATATGTTATATATTACTACTAA

>IbWRKY11

ATGGCTGCTTCTTCAGGGACTGTATACTCCACCGCCTTCACCGAGCTTTTGGCCGGAGATGTCTACCCAGCCGGCGTTAAGTCTTTGTCGCCGCCTTCTCTGCCTCTCTCTCCTTCCACCCAGCTACTGGACTCGCCGCTTCTCCTCTCCGCTTCCAATCTTTTGCCTTCTCCGACCACCGGAACTTTTCCGGCTCAGCCTTTCAGCTGGAATAGCACTGCCAACGTAACCCGGGAAGCTCTGAAGCAGGAAAACAATGGCGGCTTTTCTGATTTCTCTTTCCTTACCAACCCTGCTTCTGCGGGAGAACTGAACTGGGCTTATCAAGAAGGAAATGAAGTAGAAGATGCAGCATCATCAGAAAAGGCCATTGGAGAGCTGAACACTGTGGTGAACTCTCAAAGCAGAAATTGTTCAGATTACAATCACCACAAAAACCAGGCATTAAAGAGGTCAGATGATGGGTACAACTGGAGAAAATATGGGCAGAAACAGGTGAAGAGAAGCGAGAATCCAACAAGCTATTACAAGTGCACACACCCAAACTGTCCCACCAAGAAGAAAGTTGAGACAACCTTAGAAGGTGAGATCACTGAGATTGTCTACAAAGGTTCTCACAACCATCCTATGCCTCAAACCACCCGGAGATCCGCCGCATCTCCGCCTTCCTCCTATGTCTCTAATGGCACTGGGCAGCTGGATAATTCTGTTGCAACACCAGAGAATTCTTCAGTGTCATATGGGGATGATGATTTTGAGCAAAGCTCCCGGAAGAGAGAGTTAGGGGAGGGGGAGTTTCATGAATATGATTCAAATGCAAAGAGATGGAAAGCTGAAACTGGAAATGAAAATGAAGGAATTTCTGCACTGGGAAGTAGGACTGTGAGAGAACCCAGAGTTGTAGTTCAAACCAGGAGTGATATTGATATTCTTGATGATGGGTATAGATGGAGAAAGTATGGTCAGAAAGTAGTAAAGGGCAACCCAAATCCAAGGAGCTATTACAAATGCACTACTCTGGGCTGCCCAGTGAGGAAACATGTGGAAAGAGCACCGCAAGATACGAGGTCTGTGATTACAACATACGAAGGGAAACACAACCACGATGTTCCGGCTGCAAGAGGCGGCCACTCCCTGAACAGACCAATTCCCAGCAATAGCAACAACAGTCCAGCAATTGCCATTAGGCCTTCTGCAATGTCTCATCAATCCAGCCTTCTTGCAGCCATTCTCCCAACACAGGGCTCTGGGAATTTCGCGAATCAGGGAACCCTGTTCTCTGGATCCAGGGAAGACCGGCTCAGAGATGACATGTTGCTGCAGATGTTACTGCCCTGA

>IbWRKY12

ATGGATAACACTAAGTCCCCAGCATCAGAGTTGAAGAATTCAAGAACACTGAGACAAGCATCGTCGTCTAAGAAAAGGAGCATGGCGCAAAAGGTTGTGGTAAGAGTGAAACTTGGAGAAGGTGATGGCAGAAAGCGGAAGAGCGAGGGGCCTCCTTCAGATAGTTGGTCCTGGAGAAAATATGGCCAAAAACCCATTAAAGGATCTCCTCATCCCAGGGGTTATTACAGATGCAGCACCTCAAAGGGTTGTTCAGCTAAGAAACAAGTAGAGAGGAGCAAAACGGATGCCTCTGTGCTCATCATCACTTACACGTCCACTCATAACCATCCAGGTCCGCCAGAAGATAATCCCACAACTCCAGTGCCAGAAGAAGAAGGAGAAGAAGCTCAAATGGAGCAGCACAGAACCCGCCTGAAGGACAATGGTTATGAGGAGGATATTTTCCACTACTCCCAATCTCCATTCAACACCTCTGAACATATCATTATAAACCGCCCAGACGACAACTACCTTTTGGGAACCTCCAGTAGTCTTCTGCTAGACGGAGAGCCCTTGTCTTGCCGGTACCCTCATTTCATGGACTTCTCCACCCCCAAATCACAAGAAAACTACGACTTCTACGATGAGCTGGAAGAGCTTCCCCACACTTCATCCTTCATCACCTACACTTTCTTCAAGGACACAATCTCTGTGAATTCCATTTAA

>IbWRKY13

ATGGATCATCCACCGGGGGGCTGTTCTCAGACCGCAGTCATAGCTGCCATTAGACCGAAGACAGTGAGATTAAGGTCAGCGGGGAACCAAACTTTAGGTGGAAAGGTCGATATGCCTGGCGTTTCAGTTTGGTCTCCTGCTAACGTGTTGAAATCCGATGACAAACCAACCATCGTATACAAACCCATGGCAAAGCTACTCTCCAAGACAACTTTTCCTCAAAATTTGAACATGAGAAGTTCTACATCGAGTCAGCAAAACGAAGCAGCTGAGGAGACGAACCAGGTCAAACCATCTGACGTCAGATTAGAAGCTCATCAAAGTTCATCATTGAAGTCAGGAACAGAGAAAAAACCGGTTGAAAACTCGAAAATGGCATTGCAGAACACAGAAGACGATGAGCGTTCTCTGTTTCAGGCCAGTGGCGTGGACTGTCTTTCGAGCGATGGATATAATTGGAGGAAATATGGACAGAAGCAAGTTAAGGGAAGCGAGTACCCGAGAAGTTACTACAAGTGTACACATCCAAAATGCCCCGTGAAAAAGAAGGTCGAGAGATCATCACTAGATGATCAGATTGCAGAAATTGGGGTTCTCGTCTCTGAGGATACTTGTAATGAAACCAATAACCCCGTGAGGAGCGAGCAACTTACTCTGCAAAACGAACCTTGTGGACCTAGCACCGAGCATAAAAACAACACCATGTTGTCCACACGCTCAACTTATTCTAGCGGAGCCCCTCCACCGTGCTATCCCGTTACATCTGCTGCAGCGTTCCATGGTGCTGTATCAACTCCCGAAAACTCTTGCACTCCAAGTGGAATACATCGAGAAGGATTGGAGGCAGAAGGCGATGAACTAAAAGGAAAAAGAAGGCAATGCGGGAGCCAAACCAACAATGGAGCGACACTCGGGAATGGTGCAATGGAGACACAGACTGTTGTCGGGAGTACAACTGATTCTGAAACCACGGGGGATGGATTCCGCTGGCGAAAGTATGGGCAGAAGGTAGTTAAAGGAAATACGCACCCCAGGAGCTATTACAGATGCACCAGTCCTAAGTGCAATGTGAGGAAGTATGTGGAGAGAGCCCCCGATGATCCAAAATCCTTCATAACGACCTATGAGGGAAAACACAACCACGACATTCCAACAAGAACCCCAAATCCCGAAGCATCGAGATCAAGCACAAGGGCTGCAGCCACAAAGGAAAAATCATAG

>IbWRKY14

ATGGAAGGTGATGAAGGAAGATCAGGGCTACCCAATTATGGATTACAAGTCTCTTTCTCCACTACTCCCCACCATCATCATCATCATCCTCATGCGGCTATGCATCACGAAATGGGGTTTGTACACTTCGAGGATCACAACCAGGCCGTTATGAGCTTCTTAACCCCTCTCTCCTCCTCCTCTCAGCCGCTCGACGGTGGCGCCGGCAGCAGCTGCAGCAACGCCGCCTCCACCACCGCGGCCGCCGCCAAATCCTCCAGCCACGCCGCCACGGCTTCTCTAGGGTTTAGCCACTCCGAACCGCAGCTTTCTAACAGACCTTCATGGAATAATAACGACCAGGTTGGAACAATGGATCCAAAGGGGGCAAACGATGAAAATTGCAGTGGAAATGCAGCTGAGGGTAACAATTCATGGTGGAGGAGCTCCTCGTCATCGTCGCTGGTTGATAAGGGGAAAGTGAAGGTGAGGAGAAAGCTGAGAGAGCCCAGATTTTGCTTCCAAACAAGGAGCGATGTTGATGTTCTTGATGATGGGTATAAATGGAGGAAATATGGGCAAAAAGTTGTCAAGAACAGCCTTCATCCCAGGTTAATCATAACTGACCAGCTTAAACGAAGAAGGTCATCAATAGCTATTCTAGCTTGA

>IbWRKY15

ATGGAGCCCGATTGGGATCTACATGCAGTGGTCAGAGGTTGCGCCGTCACCTCCACCGCCGCCGCCGCCACCACCACTTCTCCTTTCTGCAGCTTCAACCCAAGGCAACAAGACGAAAACCTGGACTTCTCTCATGAGCCGTTCGATTTCAGCTCATCAACGGTCAATAACACTACCTGGTTTAACGAAGAGTTGCATGACCTTTACCTGCCTTTCCTCCACCGCCGGGAACCGCCGTCACTTCCGCCGCAAAGTCCGCCGCCGCCGTTCCCTGTTCTCCGAGGATTAGAAGGTTCAGTACTCCACAATCAACTTAACATCACGGCCACAAGGATACAGGCAAACGGCCAATCTCTCTCTTCTAATGCTTCTTCAACTACTTCCAGTAATTCTCGTCCCCAGACTCCGGCGGGTAAAAGAAGGAAGAACCAGATGAAGAGGCTGTGTCAAGTTCCGGCTGAAGATTTGGCTTCTGATATGTGGTCTTGGAGAAAATATGGGCAAAAACCTATTAAAGGCTCCCCATATCCAAGAGGTTATTACAGATGTAGCACGTCAAAGGGATGTTTGGCGAGGAAACAAGTGGAGAGGAATAGATCCGATCCGAATATGTTCATTGTCACCTACACAGCGGAGCACAACCACCCTATGCCTACGCACCGGAACTCCCTCGCCGGAAGCACGCGCCGGAAGGGTGCCAGCCAGCAAACAACCACGTCGGGGAGTGAAACGAACAGGCCCAGCACTAGCTTGTCGCCGCCGGAGAAGCAAGAAAGCAGCCGTGATGAGAAGGGCGGTGTTTTTGACGATGAATTTGGAGTGTCAAACATGGTAAATGACGGAAACGCCCCGGAGGAGGACGACGATTTCTTTGAGGGAATGGCGGAGCTGGGCGAGTCACTGAAATCGGACGACATCGGAGATTCCTTCTCCGATAACTTTCAGGACGCCATGCAGTTCCAATGTTGGCTTCCGACCACCGCCGGCGGCGGCGGTTGA

>IbWRKY16

ATGGAAAAGGTTGGTGTTTTGGAGGGAAACTCTCTAATCATCAATGAGTTAACTCAGGGGAGAGAGTTGGCAGCCCAGCTGAAAGGGCAGTTCGATTCTTTTACATCACCGGAGATATGTGAGCCACTGGTGGAGAAGATCCTTTCTTCCTATGAAAAAGCACTCACACTCCTCAACTTCAAACTCTTTTTTGGAGGAGACCCTAATGCCATGGATTCCCCTCTTCCTCTCCTGGCTAACAATTCCAATGTTTGTCCCACCGGCGAAGCCTCCGATGGTGATTCCTCCAAGGAGCAATGCCATGTCTTCAAGAAAAGAAAGACATCTCCACAGTGGAGCAAACAAGTCCGGGTTTGCTCTGCCTCAGGTTTAGGAGCCAACCTTGATGATGGCCATAGCTGGAGAAAATATGGGCAGAAAGATATTTTGGGGGCTCATCACCCAAGAGCATATTATAGGTGCACTCATCGCAACACGCAAGGCTGTTTGGCTACAAAGCAAGTTCAGAGGTCTGATGGAGACGCTTCTGTTTTTGAGGTTACATATAAAGGAAGGCACAGTTGCAAAGCCTCTCATCCAGCATTGATTTCTGGCGAAAACGAGAGGCCAAAGCCTCAGTTTCAGGTGCAACAGCCCGAGGGAAAGCAACTGCAAGCACAAAGCTTGGTGCTTGACTATGGACTAAATCAGAAACTTGAAACCACCGAAGAGGATCATGTTCTCCCTCCCTTCTCGTTCCCTCCTACAATCAAATGCGAGGTAGCAGACAAGGAAAATAACTTGCTCAAGTGCTGTTCTCCTTCACCGTTTACCGCTCCATCAACATCGGAGGAGTGTATGTACTTGTCATTTTTGCCTGGCCAAAATGATGATGATGATTTTGGACTGAGCCAAATTCTGGACAGATCAGAATCTGATCTCACTGATCATATGATTTCAACCCCAACTTCTGTCACCAACTCTCCTTTCCAAGATTGGGATTTTCTAGCTGATCAACCCAATCTTGATGCAACCGATATCTCAAAGTACTTCTCCTGA

>IbWRKY17

ATGGAGGAGATTGAAGAAGCTAACAGGGCAGCTGTGGAGAGTTGCCATAGGGTTATTAGTCTGTTGTCTCAGCCTCATGATCAGAGCCAAGTGAGAAAGGTGAAGAAAATCCAGACCCCCTCTCTCCCTCCAAGCATTCTTTTGGAGAACCCAATGTGCAGAGGGGATGATCATCACCCCAAAGCCTTGCAGCTTCTGCCTGCCATTTCTCTTGAGGCCTCAAACCAGGAAAAGGGTTCTTCTGGTGTTATTAAGAGTGGTCTTGCATTGGGGAGCCCTTCGTTCGAGTTAAACTTACATGGTAAAACCCCGGTCCCGCTATCCCACCAGACTCCGATTCCTAGCTATCACTTCCTTCAACAGCAGCAGCAGAGGTATCAGCAGCAGCAGCAGCAGCAGTTGAAGCAACAGGCAGAGATGATTTATCGTCGTAGCAATAGTGGCATTAGTCTGAATTTCGATAGCTCTACTTGCACCCCTACCATGTCCTCCACTCGCTCGTTTATCTCCTCTCTGAGTATCGATGGGAGCGTCGCGAATATGGACGCGAATTCCTTCCATTTAATCGGTGCCTCTCGCTCCGCTGATCTGAGCTCGTATCAGCACAAGAAAAGGTGCTCTGGAAGGGGTGAGGATGGAAGTACGAAATGTGGAAGCAGCAGTCGATGCCACTGCTCCAAGAAGAGGAAACATCGGGTGAAAAGGTCAATTAAAGTTCCCGCTATCAGTAACAAGCTAGCTGATATCCCTCAAGACGAATATTCTTGGAGGAAGTACGGGCAGAAGCCAATCAAAGGTTCTCCTCACCCAAGGGGATACTATAAATGTAGTAGCATGAGAGGCTGCCCTGCGAGGAAGCATGTGGAAAGATGCTTGGAAGACCCTTCAATGTTAATTGTGACTTACGAGGGAGACCATAACCATCCTAGGGTGCCATCACAGTCAGCAAACACATAA

>IbWRKY18

ATGGCTGCTTCTTCAGGGACAATAGACGCCCCCACAGCTTCTTCATCTTTCTCTTTCTCCACCGCCTCTTCATTCATGTCCTCCTCCTTCACTGACCTCCTCGCCTCCGACGCCTATTCCGGCGGCTCTGTGAGCAGAGGGCTGGGTGATCGGATAGCGGAGAGGACTGGGTCGGGTGTGCCCAAGTTTAAGTCTTTGCCGCCGCCGTCGCTGCCGCTATCTTCGCCGGCCGTCTCGCCGTCCTCTTACTTCGCTTTTCCTCCTGGGTTGAGCCCCAGTGAGCTCCTGGATTCCCCTGTTCTTCTATCTTCCTCAAACATTTTGCCGTCTCCAACTACTGGGACTTTTCCTGCTCAGACCTTTAACTGGAAGAATGATTCTAACGCATCCCAGGAAGATGTTAAGCAAGAAGAGAAAGGATACCCAGATTTCTCTTTCCAGACTAACTCTGCTTCAATGATTCAAAGAGGAAAGATGAGCTCAATTCTCTGCAGAGCCTTCCCCCCTGTGACTACTTCAACTCAGATGAGCTCTCAGAACAATGGTGGGAGCTACTCTGAGTATAATAATCAATGCTGCCCGCCCTCCCAGACGTTGAGGGAGCAGAGGCGATCTGATGACGGGTACAATTGGAGGAAATACGGGCAGAAACAGGTGAAGGGGAGCGAAAATCCGAGGAGTTATTACAAGTGCACGCACCCGAATTGCCCCACGAAGAAGAAGGTCGAGAGGGCTTTGGATGGGCAGATTACCGAGATTGTCTACAAAGGAGCTCACAATCACCCGAAGCCTCAGTCCACTAGGAGATCGTCGTCCTCTACAGCTTCTGCTTCAACCTTGGCTGCCCAGTCTTATAATGCGCCTGCCAGTGAAGTCCCGGATCAGTCGTATTGGTCTAATGGTAACGGGCAGATGGATTCTGTTGCCACGCCAGAGAATTCTTCGATCTCCGTGGGGGATGATGAATTCGAGCAGAGCTCTCAGAAGAGGGAGTCCGGGGGAGACGAGTTTGATGAAGACGAACCGGATGCAAAGAGATGGAAAGTGGAAAACGAAAGCGAGGGAGTTTCTGCACAGGGGAGTAGGACAGTAAGGGAACCGAGAGTTGTAGTTCAAACGACGAGTGATATTGATATTCTCGACGATGGTTATAGATGGAGAAAATATGGCCAGAAAGTTGTGAAGGGAAATCCCAATCCAAGGAGCTATTACAAATGCACGAGCCAAGGCTGTCCGGTGAGGAAACACGTGGAAAGGGCTTCACACGATATCCGCTCGGTGATAACAACCTACGAAGGAAAGCACAACCACGACGGCCTTCGACGATGTCTCCAATCTAACTATCCCATCCCAATCCCGAGCACGAGGCCAATGCAGCAGGGAGAAGGCCAAGCGCCTTACGAGATGTTGCAGGGACCAGGCGGTTTTGGGTACTCGGGATTTGGGAACCCGATGAACGCCTACGCGAACCAAATCCAGGACAACGCGTTCTCGAGGGCCAAGGAGGAGCCTAGAGATGACTTGTTCGTGGAGACATTGCTAGCTTGA

>IbWRKY19

ATGGCTAAGAACGACGGAGGATCATCATCATCATCCGCGGCTTCGAGATCGGCTCCGGAGCGGCCGACGATTACTCTGCCGCCGCGGAGTTCGGTGGAGAGTTTGTTTACGGGCGGGTTTATGAGCGGGATAAGCCCGGGGCCTATGACTCTAGTCTCGAATTTTTTCTCGGAGGGAGATTCCTACTCCGAGTGCCCCTCCTTCTCACAGTTGTTAGCTGGCGCCATGGCTTCTCCGGCCGCTTTTGGCGGTGTTCGGCCGCCGCCGCCGCCGCAGCCTGTTGAGGCGAAGGAGGAGTCCGGTGGTGGTGGAAGTGGTGGAGATTCAGATTTTAGGTTGAAGCATAATCGGCCGGCAGGGTTGGCGATAACTCAACAGTCAATGTTTACCATACCGCCTGGTTTGAGCCCAACGACTTTGCTTGATTCACCCGGATTTAGTGCGCTCTTTTCGCCTGGTCAGCCTTTCACACTGTACTGTATTGGTGGACTGGTGGACTGGTGGACTGGTGGTGTTTTAATAAGGAAATCTGCAAGTGTTGCTGTGACAGCACACGCTCTCCTATGTACCAAACTTAAGAATCCGCGTTTGTTCTGTGCAAAGCGCACAGTTTCATTCCTTTCTTGTTTTCTCTCTCTCCAGGGCGCTTTTGGAATGTCTCATCAGCAAGCACTTGCTCAAGTTACAACTCAGGCTGCACAGGCTCAAGTCCAAATGCATATTCAACCTGACTACTCTTCTTCATCAGCAGCTCCTGCAACATCATATTCACAGCTACAGACTATAGCATCAAATGCAACAATAAATCAACAGGTGCCTTCTCAAGCATCTGATCATAATATCATGAAAGAATCATCTGAAGTTTCCCATTCTGATCAGAGAATAGAACCAGCTTCCTTTCCTGTTGACAAACCTGCTGATGATGGTTACAACTGGAGAAAGTATGGGCAGAAGCATGTCAAGGGAAGTGAATTTCCTCGAAGTTACTATAAGTGCACACATCCAAAGTGTCCAGTCAAGAAGAAGGTTGAACGCTCTCTCGAGGGCCAAATAACTGAGATTATATACAAGGGTCAACACAACCACCCACCGCCTCAAAACAGAAAAGGTGCAAAGGATCCTGGAAACTCCAATGGGCCCTATGCCCTTCAAGGTGGCTCTGAACTGAGCTCCGAAGGCCTGACAACAAATTTTAACAAGCCCAAGGATCAAGAATCAAGTCAAGCTACACATGAACATGCGTCTGGATCAAGTGAAAGTGAAGAAGTGGGTGATGCAGAAACTAGAGCCGATGGAGGAGATGATGATGAACGTGAATCAAAGCGGAGGGCCATAGAAATGCAGGTTCCAGATCCAGCTACATCCCATCGGACAGTTACAGAACCAAGGATTATTGTTCAGACTACTAGTGAAGTTGATCTTTTAGATGATGGATACCGGTGGCGAAAATATGGCCAGAAAGTTGTTAAAGGGAATCCCTACCCAAGAAGCTACTATAAATGTACAAGTCCTGGGTGTAATGTACGAAAACACATTGAGAGGGCCTCAAATGATCCTAAAGCTGTCATAACAACATACGAGGGCAAGCATAACCATGATGTTCCTGCTGCTAGGAACAGCAGCCACAACACATCAAACAACAATACCGGGCCACAACTGAGGCCACACAATGTTGCAGGTCAACAGCAGGCTGCACTTAGAACAGACTATTCTAGCAATGAACAGCAAGTAGCTCTCCTACGATTCAAAGAAGAACAGATTACATAA

>IbWRKY20

ATGGAGAACAAGGCAGCTGAGCTGAGCAAACCCGAGAATAATAATAATAATAATAATCCCATGGTTACCCCTTCATTTTCCGACCAGATTCCGGCCACCTTTTCCCTCCAAACCCTCTTCGATATCCCCTCCTCCGATCACAGCAAAAACCCTTCCTCGAGCTTCTTCGATTATCTCTTCCCTTCCCAAGACTTATCCACCGCCGTCTTCGATCTCCTCCAAACGCCGCAGCCGCCGTCGCAGCCGCTTCCCGAGTCGTCGGAGGCGGTGAACACTCCGGTGACCCCCAACTCCTCCTCCATATCCTCCTCGTCCAATGAAGCCGCCATTGACGATCAGCTCCCGAAAACGCCGGCGGAAGAAGTTGAACAGGAATCCGACAGGAGTAAAAAACAGTTGAAGCCTAAAAGGAAGAATCAAAAGAGGGAAAGAGAGCCAAGAGTGGCGTTCATGACAAAGAGTGAAGTGGATCACTTGGACGATGGTTATAGATGGCGAAAGTACGGCCAAAAAGCCGTCAAAAACAGCCCCTTTCCCAGGAGCTACTACCGGTGCACGACGCCGGCGTGCGGCGTGAAGAAGCGGGTGGAGAGGTCGTCGGAGGATCCGTCAACGGTGGTCACCACGTACGAAGGCACGCACAGCCACCCGTGCCCGGTGACGCCACGCGCCACTACCGTAGGGATCATGCCGGAACCCTCCAACTTCGGCAGCGTCAGCGCCGCCCCCGGCGGGACCGGAAGTCCGCCGCCGTCGTCTTTTCTCATCCCTCACCACCACTTTCACTACCCAATGCAGCAGCAACAGCCGTTTTTCGCCATAACCTCGCCGTTGCCGCCTCCTTTGACCTATACCCCATCCACCTGGCAACCTAGAGAGGAGGTTCCCTCCGCCGTCGTCGTCTTCTCCGGCCGCCCGGGACGATGGGCTTCTGCAGGATATGCTGCCATTCCCCTTGAGAAAGGAGCCCCGCCCCGAAGAATAGGCTGCTTAGTGTAA

>IbWRKY21

ATGTTCCGATGCTCCTCCCCGCCGTCGCAGCCGTATCTGTCGCTGATGAATATGATGAATAATAATAACGGTGGTATGGAGAGTGGGTTTTTGGGGATGAAGATGAGTGCGGGTGACGTGGAGGTTCCCTGCAGTGAGGAGATGAAGAGTGAGTCTATTTGCACTGCTGCTGCAACCACTGAGAATAATAGTGGTTTCACGGAGATCGGGGCGGCGAAGTCGCCGTCGTCGTCATCGGCGGGGAAGAAGAAGGGAGAGAAGAAGATGAAGAAGGCTAGATTTGCTTTCCAGACTAGGAGCCAAGTTGATATTCTTGATGATGGTTATAGATGGAGGAAATATGGTCAGAAGGCTGTTAAGAACAATAGATTCCCAAGGAGCTACTATAGGTGCACCCATCAAGGATGCAACGTGAAGAAGCAAGTTCAGAGGCTGTCAAAGGACGAAGGAGTTGTGGTGACAACCTACGAAGGCGTCCATTCTCACCCCATCGAGAAATCCACCGACAACTTTGAGAACATCTTGAGCCAGATGCAAATCTACGCCGCCTTTTGA

>IbWRKY22

ATGGATGGTGAAAATGATTGGGATCTTGCTGCAGTGGTGAGAGGCTGCAACAACCTCAATGGCAATTCAAGTAACCAAGATGTTCACGAACATTTTAATGGTGGTTTTCCTAGCCAAACCTATAACTCAATCCCTTTACTCCCTCAACAAAGCCGCGATCATGGTAGTGATTTTCCTAGCATAGTGACTGAAAGGTACTATTTCGGGCTAGAAGAGGTTATTGATAAGTTTACTAGTAGAAAGATAAGAGAACCGGTCGTAGATCCTCAGAGTACCATCATTCCCCCTACTAGTCTCGACAAGGTTGATGGCTCGGGCTCGGGGGGTATCGAGAAGGACACCCCAGTTCCCCAAAGTCCCGACCCTTCTCCTACTTTGGAACCTCAACAGCCCCTTCCAAGTCCCCAAACTGATAGTCTTGAAAAAGGTGGTGGCTTGGAGGATGAGAAGCTTCAAGTGAATGAGGTTGAAATGTTGGTTGAAAAGGTTCAAGTGGCGGTTGAGAAGGTTGAAGTTCCAGTTGAGAAGGTGGATGAATGGGATGGATGGGTTTGGAGGAAGTATGGGAAGAAGATGGTGAATGATTCACCACACTCAAAGAGCTATTACAAATGCAACCACGAGGGAGAAAAGTGCCCTGCAAAGAAGCATGTTCAGTTAAGCCACATGGATGAAAGCAAGTATATCATTACGTACAGAGGTAATCACAATCACCCTCCCCGTTCAAAACACTACAACCAAACATAG

>IbWRKY23

ATGGATGACACAGGAGAAGCGTCTAAGCCATCTCTGCAGCTCCAAAATACATGCGCCGACATCGGCGGCGGAGGTGGCGGTGGTGATGAGCCTAGTGGAGCGACGACTGGAACTGAGACTTCAGAAGAAGCTCAAGTGGGAGGTTCTGACTCCGAAGAAACCCTAGATACGGTGGACTCGCCTTCCATACAGTTGGATAAGAGTGCTAGCCGACCGGATTCCCTTGCTACCTCCTCCTCGCACGTGCTCTCTGAAGTTCCAATCGAGTATAGCTTGCACCCGTCCGAATTCCTGAAGGAAATCAAGGATGAGGTTGGCATTTCTAACCAGAAAGCCTCAACTGTTCAAGCTCAAAGGCGGAACCAACTGCAGTCTGCTGATGATCCATCTGTGTTGGAATTATCTCCAACTTCTGTTACACAGTCCATATCATCCATTCCCAGCCCAACTCCAGGAGAGCGAAGATTGTCTCCATTAGAGAACAGAAATGGCGCATGCATCCAAGAAGTAGATAACCAGAATTCTTCCAACTCCAAAGCTTTATCTCTTGTTCCTGTCTTAAAGATACAAGCACCTGATGGGTACAATTGGCGGAAATATGGTCAAAAGCAAGTGAAGAGTCCTCAAGGTTCTCGGAGCTATTACAGATGCACATATTCTGACTGCTGTGCAAAAAAGATTGAGTGTTCTGACCACACTAACCGTGTTACAGAGATTGTTTATAGAAGTCCTCACAATCACGAGCCACCCCGAAAAGTAAATACCCCTAAAGTAAACAAGCTTGCAATCTCATCTATGCCTCGTAGTCAGGATAGCAAAGTAGCTCGCCTAAATAGTAATGCTGATGAGACAGTGCCATCCACTTCAAAGAAACATGTTAAAGAAACAATACCAATATCAGAGACAAAGCAGCAGGATTTCTCTGGATTGGATGACAATGCTGAAACTAATGTTAAACGGGAGGATTGTGATGAACCTACACAGAAGAAAAGATTGAAGAAATGTTCCTCAAGTCCTGAGTCTCTTCCTAAACCTGGCAAGAAAGCAAAATTGGTTGTTCACGCTGGTGGTGATGTGGGAATCTCCAGTGATGGCTATAGGTGGCGCAAGTATGGACAAAAAATGGTGAAGGGTAATCCCCATCCCAGGGCTGTAGACAACACGACTGCTGTCATTATAACCTATAAGGGGGTTCATGATCATGGCATGCCAGTACCTAAGAAACGTTATGGCCAACCTAGTGCTCCCCTAGTTGCCGCGACTGCCTCCGCTTCCATGACTGATTCGCAGACTAAGAAATCTGAACCAACCACCCAGTGGTCAGTAGACAAAGAAGGTGCATTAACAGGCGAGACATTGGAGCATGAAGGAGAGAAAACTGTGGAATCAGCTAAAACTCTATTGAGTATTGGATTCGAAATCAAGCCTTGTTGA

>IbWRKY24

ATGGCTGCTCATACTGAGTTTATGTATCCAGAAAGTATGATGGATGCTGAAGAGCTAATACAGGAGCTTCTTGACGACGAATCGCCGTTGTTCTTGGCTCCTCAAGAGACAATAATGGAGTCCAGTGGTTTTGCAGGTGTTTCAAATTACTCACTCCTTAATAGCTTGATTTATGGCCATGCAAACCAACCCCTGCATGATTCCAGAAGTTGTATGTTGGAAAGAGGTTTGATGGTGAGTAGGGATCATCATGAGAGTAAATATACTTTGAGAATCAAGACTAATTGTGGCAATGCAATGGCTGATGATGGTTATAAGTGGAGGAAGTATGGCCAGAAATCTATCAAAAACAGCCCAAATCCCAGGAGCTATTACAAGTGCACAAACCCCAAGTGTGGAGCCAAAAAACAAGTGGAGAGATGCAGTGATGATCCAGACACCCTAATAATCACATACGAAGGACTTCATCTCCATTTTGCATATCCCTTTTTCACGTTGGATAATGAACCCAACAAAACCATTGACTTAGTACCCACAAAGAAACAAAAGAAGACCATTGCAGAAGAAGTAGTGGAGTCCCAGGAACAGGAGCAGACAAACCATGTAGTGTACGAGAATAATCCAGGTAGGGAGGACACAAATCCGACCCCAATTGATGAATGGGATTCACAAGGGTTGCTTGAAGATGTGGTTCCTTTAGTTATTCGCAGACCATCAACTATTTGTAACGCCACGACGTCGTATTCTTCGTCTTCCTCTTTTCTTTCTCCTCCTACTTCACCCTCATCACTCTCATGCTCCACTAATAACTGTTTTCTTTCAGATTTTGATGCTTAA

>IbWRKY25

ATGGCCGAAGATTGGAAAAGGGCCATCGGAGAGTTGATTCGCGGCCAGAAGTTAACCAACCAACTAAGGGACTCGTTAAAGGACCCTAAGGTTGCCGACGATCTACTGCGGCAAATCTTGGGAACTTTCAACAAGACCCTTTGGATTCTTAATAAGTCGTCGATCGACACCGATGAGGTTTCCCAGGGCGCCGGCGACCCCAGCTCGCCGTGCTACGACGGCCGGAGATCGGAGGATTCCAGTGGTAGCTGCAAGGCTTTTGTGAAAGATCGGAGAGGATGCTACAAGAGGAGAAAAACTTGTGAAACACAAATCAAAGAATCCCCAAATTTGGTAGATGATGGTCATGCATGGAGGAAGTATGGACAAAAAGTGATTCTTAATTCCAAATATCCAAGGAATTACTTTAGGTGCACCCACAAATTCGACCAGAATTGCCAAGCAACCAAACAGGTCCAACAAATTGAAGAGGATCCACCGTTGTACCGTACAACATACCTAGGGAAGCACACATGCAGGAATTTCCAGAAATGCCCTCAGATCCTCCTGCAGCCCGAGGACGCCTCTGTCCTACTCTGCTTTGGGCAAAACAGTCAAAGTGACATGTACACTTGTCTTCCCACATTTTCTTCCTCCATCAAACACGAAAGCAAGGAGTACAATCCCCACAGCAGCCGCATCGGATCCTCCACCTCCGACTGTTTCGTGCCGTCCGATGCCGCCGGCCACGTGGCGCCGCTGTCGTCGGCGTCTGATTACGGGGACGTCATCTCCTCCGGCGGCACAATGGATATGACCCAATTCATCGACTCCGACGTCGTCGACATGGACGATTTCCTAATATACTAG

>IbWRKY26

ATGAACAAGGGCTCGAAGGATCAGGAGTACAGAGATATGTCAAAGAAGAGAAAGTTGATGCCAACATGGACAGAGCAAGTGAAAGTTGGCACTGATAATGGACTTGAAGGTCCACCTGAAGATGGATATAGTTGGAGAAAGTATGGGCAGAAAGATATTCTTGGAGCTAAATATCCCAGAAGCTATTACAGATGTACGTACCGTGCAATGCAGAACTGTTGGGCAACCAAACAAGTCCAAAGATCTGATGAAGATCCAACCACATTCGAGATCACGTACAAAGGGGCACATACATGTAGCCAAGCACCGAAATCAGTCCCACCGCTAGCATCACCCAAAAAACAAGACCTGAAACAAAGCATCCACTGCAAAGACAGTCTGTCGATGCAACCAAATCAGATGTTAATGGAATTAAGATCAAACCTGAGAGTCAATACCAGTGACTTGGAAAGGAAGGAAACCACATACCCTTTCTCCTTCCCTCCAACATTCTCTGGATTAACAGATGAAAAGCCGATGTTCCAGATTTCACAGGTTGATGATAATCTCTTGGGGACATACTCACCATCCTTTGTGTCTCCTACCACTCCAGAGTCAAACTACTTCTCTGTGTCACACCAACAGACTAGCAGTTTTGGGGGAGTTCAGAATTTGCACCACTCTGAGTCAGACCTTACTGACATATTCTCTGCCAATACATCATCTACAAACTCTCCAATTGTGGGCTTAGACTACACACTGGACCCCGCAGATTTTGATCCAAATTTCCTATTTGATACCTCAGAGTTTTTCACATGA

>IbWRKY27

ATGTTCTCAACTTCGATGGTTTTGTCGGGTCCACCGCCATGGTTTCCAATGATGCCTTGTTGGAGGAGCTTTACAAGCCGTTTTACCAGCCTGCAGCCGTCCATTTCTTTCGCCGGAGAAGTGAAACAAGCTGAAGGCGCTGTTCATCAGATACTGCAGCAAGACGGGGATGTTAAGAAGGGTGGTTTGGATAAATCTTCGCCGGTTTTGGAGACGCCTAATTATGTGCCCAAGTTCAAAAGAAGGAAAAATGAGCATAAAAGAGTGGTGGTTCAAGTTCCAGCTGAAGAACTGTGTGAGGATAAGTGGGCATGGCGAAAATATGGCCAGAAACCCATTAAAGGCTCTCCCTATCCTAGTAGAGCAAAGCAACACGGCGGCGGGGATGTTCATAGTAACATACACGGCGGAGCACAGCCACAGCCAGCCGACGCGGCGGAACTCCTGGCCGGGACTATCAGAAACAAGTTCCCACCAAGTACGGCCTCAAAAGCCGTGAAAGCTGCCGAGGATTCATCAACTGCTCATCATGTTCCGGAATTGGTGGCGGCGGCTTCAGCTCTGTCGTCCCCGACCGACAACAATTGGAGGGCGTGCAGTGAGGAGGAAACCAAGATCAAAGACGAGGATGATGATGAAGAAGAGTTGGAGATGGGGGAGAAGATTTCTTTGAGTGATCAGATTCAGAGCGGTATGCCGCAGGTGACGATGGATAATAATGAAGATTTCTTTGCAGATCACGCAGCGGTCATCCCACCTAATGAGTTGTTGAATCACTCACGATGA

>IbWRKY28

ATGGATAACGATAATACTATTACGAGGTTAATCCTACATGGGATTAATCTAGCCAAGGAGCTGGAAGCCGATCTTCCTAACCTAGCCAGCCAGCCGCCGGAAGCCGTTTCCGCGAGCTGCGAGGAGATCATTAGGGTTTTTACTAGCGTGAGGGAGAGGCTAGCGCCGCCGCAGCCGCCGCCGTCGAGTTTGCAGCCGTATAGCTTGGCTATGATCCAGGAGGCGCCGCCGCAGCAGCCGAGGCAGCCAGCCGGCGCCGGTCAAGCACTTGACTTGTTTCATCAGGTGTCCGGCGGCGAGCAGGCGGCGGCTGGCGGGAGCGGAGCCGACGTGGCGGAGCCGAGCCGCCGGAGAAGGATGGAAGAAGCAGACAGAACTACTGTATATGTACTTGCTCCTCAAATGGGAAATCTTGACATGCCTCCTGAGGATGGGTATACATGGAGGAAATATGGTCAGAAAGACATTCTGGGTTCTAGATTCCCGAGGGCGTATTACAGATGTACCCATCAAAAACTGTACAACTGTCCAGCCAAGAAACAAGTGCAACGCCTTGACAACAATCCACAAATGTTGAAAGTGACGTATCGGTACCACCACAGGTGTCACATGTCGGCCACGTCGCCCTCGGCGGCGCCACCGCCGCCGCCGACAGCTGGGGACGTAATTCAACCGCCAGGAACAACCACCCATCCGCCGCCGCAGCCGGCGGCAGGTGGCGGAGGCGGCGGCAGCTACTGGCTCTCCATGGATATCAGGCCAATAACATCGGAAGGCGGCGTGCAAATGCAGACTGAATTTGCGAGCACTAGCGGCGGCGCGGGGCCGTCGGGGTCCGGCAGGTACGGGCGGGAGGCGGCGGATTTCGGGGGGCAGGCGGTGGTGGATATGGCGGATGCGATGTTTAACTCCGGGAGTAGCGGTGGTAATAATAGTATGGACTTCATCTTCCATACTATGGATGAAAATTGA

>IbWRKY29

ATGGACGTGCCGGAGAATCTACTTGTTCGCCAGAGAAGAGCGATCACGGTGCTCGTAAACGGCAAAGCCACGACCGTTGAGCTCCAAACCCTACTCCAAAACCCGCCCCCCGACGGCGCGTCCAGCTCACTCCCCGCCGAGCTCGTGCAGCAAATCGTCAGATCTTTCAATCAGGCCATCTTCGAGCTCACTTCCGGCGATGCCGCGGCCCAGATTTGCCAGATCCCGTCCGCCTCCGCCTGCTCCGGCGGCCTCACGTTCGAGGACTCCGGCGAGACTACCGGCAAGAACAAGAAGAAGGGCCGCAGAGGGCGATACAAGAAAAGTAAAAATTCGGAGACATGGAACAAGGTATCTGAAACCCAGGAAGATGGTGGTGCATGGAGGAAATATGGCCAGAAGAATATACTCCACTCAGAACATCCCAGGTGCTACTTTAGGTGCACCCACAAGAGGGACCAAGGCTGCAGAGCGACAAAACAAGTGCAAAGAACCTCGGAGGGGTTGTACCAGACAACATATTTCGGCTACCACACCTGCAAGGATCCGCAGAGGTTTCCACGCCGGAAACCCGCCGCCGATCATGTCTTCTCCGGCGACGACGCTCCAAACGACCATCAAACCGTGCTGAAAGCTGAAAAGCAGATGAATCTGCAGGAAGAAGAAGATGAAGATGGTGGGGCCATTGATACAGTGAAGAAGGAGGAAGAAACAGCACAGAGCGAGATTTCTGTGAGCAAATCACCAGATAATAATAATAATAATGATGATAATAATGATAAGTTTTATGATGATGATAATTTTATATGGGGGGATATTATTGGTGAGTCTAGTAATTATGAATCCAGTTTTTATGCATGCTCTTCAACTAGCTTCAATGATCTTGACATGGGTGGCGTTGCAGATTTCGGTACCTTTTTTCCTCCCCATTGA

>IbWRKY30

ATGAGTTCTTCAGGTGGAAGTTTGAACACTTGTGTGGATAATTCCCATCACCACAACTCCTACTCTTCTTTCTCCTCCTTCTCCTTCACCGATCTTCTCTCCAACAACGAAGAATCCAAGAACCCAGAAAAGGGTTTGGGTTCTTCTTCTTCCTCCTTTAACTGGGGGATTTCTGACACCCACGAAATCCCAAAGTTCAAGTCTTTCCCGCCGGCGACTTTGCCCATTTCACCGTCTCCGGTGTCTCCTTCGTCTTTTCTTAATATCCCCGTCGTTGAGCCCTTCTGTGCTGTTGGACTCTCCTGTCTTCTTCTCCACCTCAAATCATTATTCTTGGTGTTTAGGTTGTGGTTTGTTTTCGAGGGGTTTGAGGTTATGCTGATGTTGTTTTGTGCGCAGGTTCTTCCGTTATCTCCCACCACTGGTGCATTTGCTGGATTAAATAATAACCCTAAGGAAGAAGAAAGGAAGAGCAATGATTTCTCCTTCCAAAGTAGGGCTGCCTCCTCTTCATCCATGTTCCAATCTTCTCTTGGAAGAAATTCAATGGAAGAACAAATGTCAAGGCAGCAACAACAACCAAACATGGGATCTGCAGATTTCTCCACAATGAAGACTGATATAAAACCAGAATTGCCTCAAACCCATAGCTTCTCCCAGGAAAACCCTGCGATGCAGCAGCAGCCTGCAATGGTGCATTACAGCCAGCCATCTCAGTATGCAAGAGCCCAGAAGGCGGAAGACGGGTACAACTGGAGAAAGTATGGGCAGAAGCAGGTTAAAGGGAGCGAAAACCCGAGGAGTTACTACAAATGCACATTCCCAAACTGCCCTACAAAGAAGAAGGTTGAGAGGAACCTGGATGGACACATCACTGAGATTGTCTACAAAGGCAACCACAATCACCCCAAGCCTCAGTCCACCCGAAGATCATCGTCGTCTTCCCAGTCGGTTCAGATCAACCCCGAAAGCTTTAACATTGATGTCGCGAACCAATCAAATATGATGCTTGGAAGCACTCAGCGCGACAGCTTTATCACCCCTGAGAATTCCTCGGCTTCCTTTGGCGATGAGGATCTCGAGCAGGGCTCTCCATCCAGAGATGACGATGAAAACGAGCCTGAAGCTAAGAGATGGAAGGGCGACAATGAAAATGAGGCGATATCATCTGCAAGCAGAACAGTGAGAGAACCAAGAATTGTGGTTCAGACAACAAGTGACATTGATATTCTTGATGATGGTTATAGATGGAGAAAATATGGACAGAAAGTTGTCAAAGGAAACCCAAATCCTAGGAGCTACTACAAATGCACGTTTCTGGGTTGCCCCGTGAGAAAGCACGTGGAGCGAGCCTCCCACGATCTTCGGGCAGTGATCACAACATACGAGGGAAAACACAACCATGATGTTCCTGCAGCACGCGGTAGTGGGGGCTATTCCCTCAATAAACCTCAGCAACCTCAGCCTGCCGGTAACATGGGCAGCAGTGCAGCACCAGTCGCGCTGAGGCCTTCAACGATGCCCAACCACTCGTTGAACTACCAGAACGCCATTTTCAACCCGAGGCCACAGACCACACAGAGCCAACAGCCAATAACCTTGCAGATGTTGCAGAGACCTGCAGGGAATTTAGGGTACTCGAGCTTAGGGAACTCGACGGGATCCTACATGCCCACTGGAAAAGACGAACCTAAAGACGACTTCTTCAGCACTTTCCTAAACTGA

>IbWRKY31

ATGGCACACTCCTCGCCGGAAAACTCATCGGCCAACCGCAAGAGGGCCGTCGACGGCCTGATTCTTGGCCGGAACTTGACGTGCCAACTAAGAGAGGTGCTGAAAAACAGCTCCGACGAACATGGACCGCCGTCTAAGGTGGTTGCGGAGGATTTGGTGGCGAAGATTCTGGAATCTTTTAATGAGGGTATTTCCGTAATTGGATCCATGGATTCCGACGAGGTTTCTCAGCCGCCTTCCGACGGCCGGAAATCGGAGGACTCTAGTGGTAGTTGCAAGACTACTTCTGCACTCAAAGATGGTAGAGGATGCTACAAGAGAAGAAAAACATGTGAGACACTGATTAAAGATTCTCAAACTTTAGTAGATGATGGATATGCTTGGAGAAAATATGGACAAAAAGTTATCCTCAATACCCCATATCCAAGGAATTACTACAGGTGCACTCACAAATTCGATCAGAAATGCCAAGCAACCAAACAGGTGCAAATGATTCGCGAAAACCCTGCTCTGTACCGCACAACATATAATGGCAATCACACATGCTTGAACTTCCAAAAATACCCTCAAATTATCGTGGACTCCACAGCACATGGGGACTCTTCTTTTCTACTTTGCTTTGGGCAAAATGGTCAAACCAACAAGGAAGTTCAGAATCCAACTTTAATAAAGCAAGAAAACAATAAGCAAGAGTTTGCTGAGAATTTGTACCACAGCCACATTCAATCATCAAGCTCCGGTTGTTGTTTGCCGTCGTCCGATGACCGTCCGATGTCGTCGGCCCGGTGGGGGCCGGCGGCATCGTCGGGGTCGGAGTATGGGGACGTGAATTCATCTGGATGCACTCATGATGATTTGGGTATGCAAATGATGGGCAATGTCGATGTTGATGATTTCACATTAGGGTTCTTGGCGGATTTTTGA

>IbWRKY32

ATGCCCAAACCCACCATGGAAACCCCAAACCCTTTTCACACTCACCGGAAAAGAGTCATCTCAGTGCTCCTCAAGGGCAAGAAATCAGCCACTCAGCTCCAAACCCTACTCCGTAATTTTTCCCATGGATCCCAAGAAAAATCCCACCTTGTACTCGAAATCTTGGGATCTTTCTCCGAGGCTGTCTCCCAGCTCAAGAATGGCCTGCCGCCGCCGGATTCCGAGCTCTCCGGTGGCCGGAACTCCGGCGACAACCCGGCCAAGGCCCGCCGAGGATGTAACAACAGAAGAAAGTGTTCAGATACTTGGATCAATGTCTCTAACACCAAGGAAGATGGTGGTGCATGGAGGAAATATGGCCAGAAACAAATCCTCAATTCAAAATATCCTAGGTGTTACTTTAGGTGCACCCACAAGCATAGCCAAGGATGCAAAGCAACAAAACAGGTTGAGAGGATATCCGAGGATGAGTACAAGACCATGTACTTTGGTCAGCACACTTGCCAAGATTCTTTTAGGGCTCCAGTTCTCGTGATCAAATCTATCTCGACTGTGGATTCTACTCAATCGCCGAGCTTTGAATGTATCACACAAGATGGCAATGGTGGCGGTGATCACTATGACGACGTGGTTGTGAATCAAGAAAATCATCGAGAATTTAAGGATCGAATTGTATGA

>IbWRKY33

ATGGAAAACGAATTTGGTGTGGATGGCGAGTTTAATCTGAATGAGCTGATAAGCGAGTTATTACAGGGAAGAGACGCTGCTAACCGCCTCCAGTTGTGCCTGAATAATGCACCATTATTGCCGTCATCGTCGTCGTCTTCCCAAGATTGGAGCTGTGAAGTTCTGGCAAATAAAGTCCAGGCTTCGTTGGATAATGCGCTTTGCATGTTAAACCATGCGGGACGAAATGAATCTTCTGATAGCGACCATGAAGTTAGAGACCCTACTTCTGGCAGAAGGAGAAAAGCCGCGGTTTGGACAGACCAAGTTCAAGTCAGTCCTGGTGAAGGCCTCGAAGAAGCCCATGACGATGGCTATAATTGGAGAAAGTACGGCCAGAAAAAAATTCTCGGAGCCCAATTTCCCAAAGGCTATTACAGATGCAGTCATCTTCATTCCCAAAAATGTTTAGCCAAAAAGGAAATCCAGAAATCGGACGAAGACCCCACCGTTTTTAACATTCAGCAGGAAGAGAATCTTTTGAATTTACAAAGGAATCTCAAAATCAAAACTGACAATATAGAGCCCAGTAGCCATAGCCATGGCAACCCATTTCCTTCTTCTCATGATTTTGCGGCAGCTTCAAGCTCGGGGGTGATTAAGGTTGGTGAGATCCACTGCGGTTTCCCGCAGCCTCTAACAAACAGTTTTGTAGCAAATGTTTCGACTACGACCACCTCAGACCTTGAAATGATTATGGAGATTCCTACGTCAACGTCAGGAACAAATACCCCAATTGTCGGCATGGGATTCCCGTTCGATGCTACAATGGGTTTTGACTTCAATTTCACTTTCGATAATAATAATGATAATAATCCTGCGGGATTCTTTGACTAG

>IbWRKY34

ATGTCGTCTAGCTCTACCACATCCCAAGCCATGCTCAACCAAATCTTATTTCAAGATGTGGCATCTTCATCTTCATCCCTTTTCTGTGTTTCTTCAAACAATAATAATATTACTGGGACTGGGACTGGGACTGCTCTTCCATTTGAGTCTCTTAAAACCCTCATCACAGTACCCATGCCTACCTCTCTTGCATCACTGCTCCCTCCCCTTGTTGAATCATCATCACCTAATTCTACTTCAGCTTTTCATCAAACACAAACACAAACACTACTACAACAACATCAAGACCTCTCTCCCCTCTTTGGACCACCCCATCATCATCAACTCCTCTCTTTGCACAGATCCGCACCAAACTTATGGGCATGGGGAGAAGTGAATGAGTGCATGATGAGAAGCAAGAGAAGTGGATTAGTGGTGGATGATCATCGTCATCATCATCACCTGGGGGGTCTGGGGGTTTCAGCAGTGAAGATGAAGAAGATGAGCAAGTCAAGAAGGAAGGTGAGAGAGCCAAGGTTCAGCTTCAAGACCATGAGTGATGTTGATGTGTTGGATGATGGCTATAAATGGAGAAAATATGGCCAGAAAGTTGTCAAAAACACCCAACATCCCAGGAGCTATTACCGTTGCACGCAAGATAACTGTCGGGTGAAGAAACGTGTGGAAAGGCTAGCGGAGGATCCGAGAATGGTGATAACAACGTACGAAGGCCGACATGTGCACTCCCCATCCCACGACGATGACGATTCACAAGCTTCATCTCAAGCCAACGATCTCCTCTGGTAA

>IbWRKY35

ATGGCGGTAGAGCTAATGATGAGTTACAGGAATGTTGATGTTAATGGCGGTGGGGGTGGGAGGATTGGGTTTGTGAAGAGTTTGGAGGAGAGCAGCGCGGTGGTGAAAGAAGCGGCTTCTGGGTTTGAGAGCGTGGAGGAGTTTATCAGATTGTTGTCGCAGGGGAAAAAGAAGCAGCAGGGCGACCGGGAAAAGGCGGCCATGGAGATTGATGTTGTGGCTGATGTTGCTGTCAATAAGTTTAAGAAGGTGATTGATCTTCTGGGTCGGACCCGGACTGGGCATGCCAGGTTCCGGCGAGGTCCCGTCGCTTCTTCGCCGGTTATGGAAGTGCCGGCCGATAATAAAGTGTATAGCCCCACGCCGATTCAGCAGGTTCCGCCTCCTGCATCTTATGATTACAGCGCCGCCGCCCACCGTTATAGCGCCGCGGCGGCGCCGATGACGATCAGTTTCACGTGCTCGCCGGAGATTTCACGCGCCAATTCCTTCAATATCTCGTCGTTGACCGGGGAGACTGACAGCAAGCCGATGCTTTCTTCTTCCTCCGCTTTCCAGCTCACCAATCTTTCCCAAGTCTCCTCCGCCGGAAAGCCGCCTCTCTCCACGTCTTCCTTGAAGAGAAAGTGCAGCTCATCGGAGAATAACCTCTCCGGCAAGTGCAGCGGCTCCTCCAGCAGGAAACTAAGATTGAAGAGGGTAACAAGAGTTCCAGCCATAAGCATGAAGCTTTCAGACATTCCACCTGATGATTATTCATGGCGGAAATATGGACAGAAGCCCATCAAAGGATCCCCACACCCAAGGGGGTACTATAAATGCAGCAGCGTCCGAGGTTGTCCGGCACGCAAGCATGTGGAGAGGGCGGTGGATGATCCGACCATGTTGATAGTAACATACGAAGGCGAGCATAATCACTCCGTCTCCGTCGCAGAAACAAATGGATTAATCTTAGAGTCCTCGTAA

>IbWRKY36

ATGGAGGCAAAGTTGTTGAGAATCACCTCTACCGCCATTCCAACCCATAGGAAAATCGCCAGAATTCCCAGAAAGCTTTTAGCCCACCGGAGCTATCGTCAAGATTGCACCTTTCAATTTCTCATTCCAAGCTGCAGTGCTATGCACGCCATTCCCTCAGCTCAACCGCTTCCAAATTGGCGGCAAGAACACATGATCGAACCTGGGCTGCCTGGCAGCCGCAGCGTGGAGTTACACTCAATGTGTTGGGCTCCTTTTGGTCACCAGACAGTAGTCCTCCCACCTAATGGGCTGCTAAGGGACCGTGATTTATCCTCCACTATCTTGTCGGGCCGGGGTGTGGAGGGCCTGGTGGCGAGGAAATTTCTCTTTTTAATGGGAATGAAGAGCAGAGTAGATGAGACAGGGAATTACACACTAGAGGGTGTAAGAATATCTTTAGTTCGGTTAGAGGATAGCATTATATCCGGCCTTGTGGAGAGAGCTCAGTATCGTTACAACCCGGATACATATGACCCTAATGCTTTTGTGATGGAAGGCTTCCATGGCTCTTTGGTTGGAAGGTATTCGGACCCTGATGAGCATCCTTTCTTCCCGCTTGAATTGCCCGAGCCACTATTGCCACCCCTTCAACACCCACAGGTTCTACACCCAAATGGTGCTTTGGTCAATATCAATGCCAAAATATGGGATATGTATTTCAAAAAGCTTCTTCCGAGGTTAGTGGAAGAAGGCGATGATGGTAATTGTGGATCCACTGCTGTTTGTGACAGTTTTTGCCTGCAGGTCCTCTCGAAGAGAATTCATTATGGCAAATTTGTTGCAGAAGCAAAATTTCAAGCTTCACCAGACCTCTATAAGGCTGCAATCAGAGCAAAAGACAGAAATAGGCTTATGCAATTGCTGACATGCCCAGAAGTTGAAGAACTGGTAAAGAAGAGAGTAGAGATGAAGGTCAGAGAACACTTTCAAGAGGTTACCATTGACATGGAAGGGGAGAGCAAGAGTGATCCAAAGTACAAAATAAATCCAATCTTTGTTGCTAATCTGTATGGGGATTGGGTCATGCCATTGACAAAGGAAGTTGAGAATTTCCCACAAGGAGAATCATCGGAATGGGGAAAGCAGCAGAGAAGGAGCTTCTGCGGTTTCGGGTCCGTCGCGCCCGACGATTACAATCCCGCCCCGACCCCCTTCGACACTACCCTTTTCTCACTAGGCTCCATACCCGGTTTCAGCCCCGGACCCATGACTCTCATGTCCACTTTCTTCTCCGATTCCGATGCCTGCTCTTTCTCTCAGTTACTCGCCGGAGCCATGGCTTCCCCGCTCGCAAACCCCGCTCTTCTGCCCGATAAAGACGGGGATTCGGGTCCGGGTTGCGAGAAACTTTCCGGGTATAAGCAGAAGCAGCCGATGAGCTTGCTGGTGGCTCAGTCGCCGTTGTTCATGGTCTCGCCGCGGTTTAGTCCTTCCGGTCTGCTTAACTCGCCGGGGTTTCTTTCCCCACTTCAGGCCCTAGCTCATGTTACGGCTCAGGCGGCATTTAACCAGTCTTACAAACAAATGCAAGTTGAATATCAGCATTCTTCGTCTGTGGAAGGTGCAGGGCACCAAACTTCGTCTTCTATGCCAAACCAGGCGGTGCAAGGTGAAGCGGCTAATGTGGCGTTGGACACAGAAAGTTTGAAGGTTGAAACATCGGAGCTCTCTCAAGTAGACAGCAAAGTTAGTTCTGGTGCTATTGAGAAGCCTGCTAGTGATGGGTATAATTGGAGGAAATACGGGCAAAAGCTGGTTAAGGGAAGTGAATGTCCTCGAAGCTACTATAGATGGAAGCACAACCATGAAGTTCCTAAGTCCAACAAGCGAAAGCAAGATGACTGTGATCAAGAGTCTAGGGAGGACAACTCCCGAGAAAAGCCTCAAAGTGCTTCTCATAGATGGACTGAAACAAACAGGTCAAGTTCTCAACCAGAGATGGTATCGACCAAACTGCAGTCTGAACAACTTACTGTCGCATCTAAACGTGATGAAATGGAGGAAACTGCAACAGTATTAGATGAAAAGGATGACGGTGAACGAAATGCAGAGAGACGGATTTCAGAGGCGGGCTCATCTGTTCTGCCTTCATCTCACAAGACAGTCATGGAACCCAAAATCATTGTGCAGACAAGAAGCGAAGTTGACCTTTTGGACGATGGGTACAAATGGCGGAAGTATGGACAAAAAGTGGTGAAGGGGAATGCTAATCCAAGGAGTTACTATCGGTGTACATACAAGGGATGCAATGTCCGAAAACACGTTGAAAGGGCTTCAACAGATCCAAAAGCGGTCATAACCGCATACGAAGGCAAACATAATCACGATACCCCAAATGCACCAAACAACAATCATACCGAAACTAAAAAACAGCAGCATTCAGCAGTTGAAACAACAAAAGTTGGTAGCTGCGTAGCTTGGAACAGATATCTGAAAGAGCAAATCGAAGAAGAGAATCCCATACAGTATCAGTAG

>IbWRKY37

ATGGCGGATTCCTTGGATATCTCCGGCGACGGTGCTGCAAAGCAAAAAGCCGGCGACTCTTTAGCTGGCCGCCAAGAAGGATTCATGACAGCCGTTCTAAAGAAGGATGAAGGTAGAGCGGCGGCGACGGTGAAAGCGGAGATGAAAGAGGTGAAGGAGGAGAACGCAAGGTTGAAGACTCTCCTTGCGAAGATAGAGAAGGATTACAGCTCTCTCCAGATGCGATTCTTCGATGTTTTCTCTAATCAACCAGCAGAAATCGAGAAGAAAAGCTGCAAAATTAGTAGCCCTATGAGCTCTCATCATCACGATGAAGAAACACAGATATCTCTTCGATTAGGACGAAGCCCTAGTCCTGATCGTCGTCAGTCGAGAGTGATTGACGATATTAACGCCGCCGCGGCCGCCAAGAGCACCGATGAAGATGATGATGAACATAATCAAACTCTGAAACTTGGGCTTGACTATGGCGGCGATAATAAGTCAACTGAGCCTAACTTGGAGCTCAGTTCGGGCCGTCAAAGCCCGGACAACAGTGCGTCGGAGACCAAAGAAGAAGACGCAGCGGCTGCCGGAGAAACATGGCCCCCCAGTAAAGCTCTTAAGGCCACGAGAAGTGGAGACGATGAACTGTCGCAGCCCAGCGTGAAGAGAGCTAGGGTTTCTGTTATGGCTCGATGTGACACCCCAACGATGAATGATGGATGCCAATGGAGAAAGTATGGTCAAAAAGTAGCGAAAGGGAATCCATGCCCGCGTGCTTACTACCGCTGCACGGTGGCGCCGTCGTGCCCGGTGAGAAAACAGGTGCAACGATGCGCCGACGACATGTCAGTCCTGATCACGACTTACGAAGGAACGCACAATCATCCCCTCCCGGTTGCAGCCACCGCCATGGCCTCCACCACTTCGGCCGCCGCCTCCATGCTCCTCTCCGGCTCCACCACTTCTCAAACCGCCGGACTCAGGTCGCCGCCATCCCCGGCTACAAACTTCTTCCCCGGCCTGAACTTCAGTCTCCCTGCAGACACTTCAAGAACAACCAGACCTCTCTATTTCCCTAATTCCTCTTCCCCACCTTTCCCAACCATCACCCTTGACCTCACCACTTCATCCAACAACATTTCCAGCATGTTTTCCTCTAATGTCATGAAATCAGCCCCCAGATTCCCTTCAACAAACCTGAGCTTTTCTTCCTCAGAATCCAACATTTCACCTGCAATCTGGAGCACCGGAGGATACACGAATTATAGTACCATCTACAACAGGAACAACAACATCCTAGGCACATCACAACCAGGAAAGTCATCCCAAGAACAACCATTTTACGGCCAGGCAGCGGCTGCTTCCCAGCAGGCACTGACAGAAACCTTAACCAAAGCAATCACATCAGATCCTAGTCTCCGGTCAGTAATAGCCGCTGCAATTACATCAATGGTTGGTAACAATGCGCCAATGCAACACAAACGAGTGAAGGTAAACGATGAAGTTATCGGCACGCATCAAGCTATTAGTTGA

>IbWRKY38

ATGTCTGAAAACCAGTTTTACCATGATCACACCGGGTTTAATCCGTTGTTCATTGGGGACGACGGCGATGATCATCGAAACCCTTCTCTGTATTCATCGAATCTCCCGCACCCAGAATTCGATCCTTCTTCTCCTTTCGTGAACTACAACGTTGTTTCCAGTTCGGGTTTTCCCACGTTGCTCTGGTCCTCTTCGACGAGGTCCGAGGTTGTCGGCCCTCCTTCCTCGGACCACCACGTTCAAGAATGCTCTAGAAAGAGCAGCGGTTCCGTTGGGCAGCCGCCGGCGTCTTCCTCCTCCAGTGAGGCCGCCGGAGGCGGCGGCGGAGGAGAAGAAGATTCTTCAAAGAGCGGCAAGAATCTAGAGGGTGTAGAGTGTGAAGATGGAGAAGACAAGTCCAAGAAAATGTACAAAGGCAAGAAGAAAGAGGAGAAAAAGCAAAGGGAGCCTCGATTTGCCTTCATCACTAAAAGTGGGATTGATAATCTTGAAGATGGATATAGATGGAGAAAATATGGACAAAAGGCAGTCAAGAACAGCCCTTTCCCTAGGAGCTATTACAAATGCACCACTCAAAAATGCCCAGTGAAGAAGCGTGTGGAGAGATCGCACCAAGACCCAACCACCGTCATCACCACCTACGAAGGCCAGCACAACCACCACTGTCCCGCCACTCTCCGGGGAAACGCCGCCGCCATGTTCTCTTCGCCGCCCTCCTTCTTTCCATCCTCAGCGCCTCAGCCGCCGCCCCGGGACCTCTTCGCCAACCAAATGTACCCCGCCGTGGTGCCGCATTCCCCGTCTCCCATAATGTACGATTATCAGCAGAGCCACGGCGGCCTTATCGGCGGCCACGCGCCGCCGCAACCGCAGTTTGATTACGGAATGTTTCAAGAGATGGTGGCGTCGTTGGTCCAAAACAGGAACATAATAATCTTTGAGGGTTTTAAGGATTATTGGTGTTTTTTTTGTTGTGTGTTTAAGTGTTGGTGGATTGGTGGTTGTGTAAGTGTTGATGAGCATGGATTGATGATTACTTTGAGAGCATTGACTTAG

>IbWRKY39

ATGGAAGAGGGCGTAGATGAAGGCTCGCTTGGAAAATTGCAGCCGAAACGGGAGCCTAACACTGGCTCTCTTGAATCAGAGACAGAACACAAAGTAAGTGATAAACTGGTGCCAGCTGAGGGGGTTTCTGGTGAGCTGCAGAAGAGATTGAGCCCTAATGTTAAGGAAGAAGCATCTGAATCAAGTGAAAGTAAAGTTGCAGTTCCTGATAAGTGTGAAACTGTGCCAAGTGATATGCAGCGAAAGCAGGGTGTTGTTAATGGGAGTTCTGCGTCACAGTCGGAGAAAGAAAAAAGTTCTCATTCTGGGGTTCAAGTGAAAGATGGAGAAGAAGTTCAGCAGAAACAGGGCGTAGGGAGTGACACTGATGCTTCACAGTTTAGCCAAGTTTCTATTGTACCAAAGAAGGAGTCTGATGGCACTGGGCATGAACAGAGTAACGATGAAAAAATTCATGGGACAGAAACCCTCGCTCTCGCTGTAATTCCTGAAAAGAATTCAGATAATCCACAGCAATTACAAATTCAGAGCATGGAGGTCCTTGCTACTCATTCTAATCAAGCAAGAGTAACTTATGTCAAACCACATGAAAAAGGTTTAGATAAATTGCAGCCTAGGAGGAACCCTGAAATTGGTGCCCACACACCACAATTTGATCAAAGAAGTCCTCCTTCAAAGGCCCCTGAAAAACCATCAGAAGATGGTTACAACTGGAGAAAGTATGGACAGAAGCTTGTTAGAGGAAATGAATTTATTCGGAGTTATTATAAATGTACTCACACTAATTGCACAGCAAAGAGACAAGTGGAGAGATCTCAAGATGGTCATATCACAGAAATTAACTATATTGGAAACCATGAACATCCTAAACCTCAAAATAGCCCTCAGATAAATGCTCCGACTATTCTCCCTATCCAAATGAGAAGACCAGACTTGCCTATCATGACTCCTTCTGAAGGTACGCAATCTGTTACACTGGGGGAAAAGTGCGAAACTCCTGAGCCAAAACAGATTACATCACCAGTAGGTGTTGTTTCGGCTGATATAGGTGCAAGGGATTCAGTCCTCCAGTCACATAATTTGCGAGATGAGGATGATCACTGTGGTGGTCCAGATTCAAAAAAGCAGAAGAAATGCCTTTCTAGTCCTGATGACAATAAGCCTCATGGTGAGCCAAGGCATGTTGTTCAGACTATGAGTGAGGTGGATATAGTGAATGATGGCTACCGCTGGCGCAAGTATGGCCAGAAATTAGTAAAAGGAAACCCAAACCCCAGGTCTTCCCACTTAGTCGATTGTATTAAGGCATCTAAGAAGTTAAGCATGCAACTACATGCTTATATGCCTGAACCTCTGGACTCTAATTTCATGTGGAGCTATTACAGATGCTCAAATGCAGGGTGTCCAGTGAAGAAACACGTCGAGAGGGCGTCCCATGATCCAAAAGTGGTTATCACAACATACGAAGGGCAGCACGACCACGACATGCCAGCTTCTAGGACTATTACCCAAAATTCAGGAGAGGGCGATGCCACAAGTGGAGAGTCAAGACCCGAATCAGGTGAAAACAAACATGTGGGCCTCGATATGGTTGTTCACATTGGTGCTAATTGA

>IbWRKY40

ATGGAGTTTACTAGTTTGGTTGATACTTCTCTGGGTCTCAATGCTAAGCCTATCAGAGTTGTTAGCGGCAAACCGAAACAAGAAGTTGAGAGCAATTTCATTGGGTTGAGAATGAACATTGGGAACAAAGATGAGAACACAGATAAAGACAACAATCTGGCGGGGGAGCTAATGGAGGAGTTGAATAGAGTGAGTGCTGAGAACAGGAAGCTGAGTGAAATGTTGACAGTGGTTTGTGGGAACTACAATGCGTTGAGGGAGCAAGTGAGGGAGTACATGAACAAGCAGCAGCAGCAAAGTGGGAGCATTAATGATCACAACAGCAGCCAAGTTATTATGGGATCAAGAAAGAGAAAATCCCCCAGCAATAATAATAATAATGCCAATTCGGAAAGCAGCTCCAGTGATGAAGATTCTGCCAAGAAACCCAGGAGGGAATTGGAACATCAACACCACATCAAAGCCAACACCTCCAAGATTTATGTCAAGACTGAAGCCTCAGATACCAGCCTGATTGTGAAGGATGGGTATCAATGGAGGAAATATGGTCAGAAAGTAACGAGAGACAATCCCTGTCCAAGAGCCTATTTCAGGTGCTCTTTTGCTCCTACCTGCCCAGTGAAGAAGAAGGTGCAAAGAAGCGTAGAAGATCAATCAATCTTGGTGGCAACATATGAAGGAGAGCACAACCATGACCTCCCTTCAAAGCTAGAACAACCCTCCGCCACCGCCACCGCCGCCGCCGCCGCCCGTTCTTTGCCGCCAGCTGCCGCCCTCAACGCCCAACCACGTGACCTATCGCCGCCCAAAACAGCACTATCAGTGCCCAATGCAAATGGTGCTAAAACCGCGTCTACCCCGGCGGGAAGCTCATTGCCGGTTGACAGGCCGGATTTTCAACAGTTCTTTATTGAACAAATGGCATCTTCCTTAACAAAAGATCCCACCTTCAAAGCAGCCATCGCAGCCGCCATTTCCGGAAAATTCTCCCCACATAACAACAACAGAGAGAAATGGTAA

>IbWRKY41

ATGGATAATAATAATAATAATATTGATCTATCCTTGAACCTTAATGAATCTCGACGACGACCTCGTTCATCAGAACTAGAATCATCGGAACCTGATAAAATGCTGCAATCAAACGCAGCGGACGGGGAGATATCCACCGGAAGTTCCTTGTCGGAAAACCAGAAAATAGAAGAGTTATCGGTGTTGCAGAGGGAGATGAAGAGGATGAAAGAGGAGAACAAGGCGTTGAGGGATGCTGTGGAGCAAACCATGAAAGATTTCCACGATCTGCACCAGAAATTTTCTTCTATCCAACAGAAGAACAATCATGAAGACAAGGAATTTGCTAAGGATTTTTTGACGCTGAGTGGAACTGATGAGACGAGGAATCATCGAGAGCTGCAAGAGAGGAGTCATCAGATAACCTCGGATCCATCACCGGAGGACGGCGGCGATGAAGACGACGACGGCGACGGTGAATTAGGGTTGTCATTGACGCTGAAAAGTAGCAGCAGTAGTAGTTCATTGATTGGAAGAAGAATGCATGGAGAAGGTGAGGAAAGAGGAGAGAAGAGTAAGGCGGAAGAGATGAATTCAACTACTGGATTTACTCCGACGCCACCGCCACCGCCACCGGCGGCGATGATACAGAACAATCCCCCGCCGGGATTTACTGCCACTTCTCCTCCCAACAAAAAAACTAGGGTTTCCGTCCGGGCTAGATGCGACGCTGCTACAATGAACGATGGATGCCAATGGAGGAAATATGGTCAAAAAATTGCAAAAGGAAATCCATGTCCAAGAGCCTATTATCGTTGCACAGTTGCCCCAGGATGTCCAGTGAGAAAACAGGTACAACGATGTTTAGAAGACAGATCCATATTAATCACAACCTACGAAGGAACACACAACCACCCACTTCCGGTGGGCGCGACGGCGATGGCCTCAACAGCCTCGGCAGCCTCGTTCATGTCGCACCTGGATTCCACCAACCCAATTTCCAACCTAAACCACGCATTTCTCATCCCTAATTACAACCACAACCCACACTTCATCATCAACCCTAACAACCCTTCTTCCCACCTTAACATCCCAAACCTCGTACGAAACAACACCATCATAAACCCTACGGCTTCAAATCCATCATCCTCGTCGTCGCCGCATTTCTGGGGGCCAAAGCTACCTGATCATCATCATCTGGTGGCTGATCATCAAAACATGAGCGCCGCCATTGCTGCTGATCCTAAGTTTAGGGTTGCGGTCGCGGCGGCTCTTTCCTCACTCATCAGTACTAAAGATCAAACCCATGCATCCTCGTGA

>IbWRKY42

ATGATTTTGTACAACCCAATCACGTTGGGGTTGACCCTCGAGAATCCTGATCAGCGCTCTGCTGGGTTCTTCACGAACAAGCCGGTTTTTGGGTTTAATCTTAGCCCGCGCTTGAATCCGATCAACGCCGGCGGTAGTGGCGGTATGATTCCGATCAACGCCGCCGCCGAGAAACGTGGGCCGCCCAACGAAGTTGACTTCTTCTCCGACAAGAAACTGCCGCCGCCGCAGGCGGCGGCGGATATCGTCGTCAAGAAGGAGATAACCTTGCACGGAGAACCCGTCACCAAATCCGATTTGAATGTAAATACTGGACTGCAACTTGTGATTGCTAACGCCGGGAGTGATCAATCCACGGTGGACGATAGTGTTTCATCGGATATGGAGGAGCGAAGAGCCAAGAACGATCTGTCAGTGTTGCAAGTGGAGCTGGAAAAGATGAACGCCGAGAATCAGAGATTGCGTGGGATGCTGTCGCAGGTTTCCACCAACTACAGCGCTTTAAAGCAGCATCTCGAGAATCTAATGCAATCCCAGAATCAGCAAAGTTCCAGAATAGGAAGCACACAAGATCGTGAGGTTGTTGACAGAAAATCCGAGGAAAAGAAGCCTGAGAAAGAAGAAACGACTGTTCCGAGACAGTTCCTGGAGTTGGTTCCCGCCGGCGGCGGTGCGGCAGCGGATGAGCCGTCGCAGTCTCATACTTCGTCGGAGGAGAGAACACTGTCTGCGGGTTCTCCGAGGAACAACACGGAATTGTCGAGGCATAAAGGGATTGCCAGGGAAGACAGTCCAGATTCAGAAAGCTGGGCTCCTAATAAACTCCCAAAGCTGAATTCTTCCAAGCCTGTTGATCAAGCTGCCGAGGCTACCATGAGGAAAGCCCGTGTCTCCGTTCGGGCCCGATCCGAAGCCCCCATGATTAGTGATGGATGTCAATGGCGGAAATATGGACAGAAAATGGCGAAAGGAAACCCGTGTCCCCGAGCTTATTATAGGTGCACCATGGCGGTTGGGTGTCCGGTTCGAAAACAAGTACAAAGGTGTGCGGAGGATAGGACGATTTTGATCACGACTTACGAAGGGACGCACAACCACCCGCTGCCGCCGGCCGCCATGGCGATGGCGTCGACGACCTCGGCGGCGGCGAACATGCTACTTTCCGGCGCCATGCCGAGCGCCGACGGGATGATGAATACTAACTTTCTTGCAAGGGCAATTCTGCCTTGTTCTTCTAGCATGGCTACAATTTCAGCTTCGGCGCCTTTTCCAACCGTTACCCTTGACCTCACTCAAACCCCCAATTCTTTGCCTAATTACCAAAGACCCCCAACTCAATTCCAACCCCCTTTCGCGGGAGCCCCTCAAATTCCCCAAAATTACCCTCAGCTCCCCCAGGTTTTCGGTCAAGGTTTGTACAACCAGTCAAAGTTCTCCGGCCTGCATGTTTCCCATCCGGACATCGGCGCAGCCGCGGCTCAGGCGGCGCAGTTGGCTCAACAGCCGCGCGTGCAGCCACCGCCGCCGCAACACCCTTTGTTCGCCGACACGCTCAGCGCCGCCACGGCCGCCATCACCTCCGACCCCAACTTCACCGCCGCCCTCGCCGCCGCCATCTCCTCCATCATGGGCGGTGGCTCCCAGCCGAATAACGCCACTAATGCCGCCGCCGCCGCCACTAGCAACACCAACAAGACTAGCAGTTTCCCGGGGAACTAA

>IbWRKY43

ATGACTCCGAGAACTCGCCGGAAAACAGCGCCGATTCGCCGCGCTCCGGCATGTTTCACGACACCAAGATGGCCTCCATCAATTCCCCCCAAAAGAAGTAGACGTGCCATTCAGAAAAGAGTGGTGTCAGTGCCGATCAACGACGTTGAAGGATCAAAGCTCAAGGGCGAGAGCAGCTTTCCGCCGTCCGATTCTTGGGCTTGGAGGAAATATGGACAAAAGCCAATCAAAGGCTCCCCTTATCCTAGAGGATATTATAGATGTAGCAGCTCAAAAGGGTGCCCAGCGAAAAAGCAAGTGGAGAGGAGCCGTGTAGACCCAAACATGCTAGTGGTGACTTATTCTTGTGAACACAACCACCCATGGCCCGCCGCCAGGAACAATCACGCCCACCGCAACGCCGTCTCTCTCACCGCCGCCGCCGCCGCCGCGCCAACAACAAGATCATCATCCAAAGCCGCCGCCGCCGACTCCGAGGACGCGCGCGAAGCCGAAGCCGAAGCCAGCGAGTTTTCCGCTCAACCGAAACCCGAAACCTCCGAAAAAATTCGGCGGACTCGGCGATTCGCCGTCGGTGATCTACAGCGACGAGTTCGGGTGGTTTTCGAGCTTCGAGCCGACGACGTCGTCGTCGACGGTGATCGAGAGCACTTCCATTTTGACGGAGGCGCGAGTGACGCACGCCGACATGTCGGTGATATTCTCAATGCGGGAGGAGGAGGAGGGAGAGGAATCGCCGTTCGCCGGCCTCGGAGAGCTGCCGGAATGTTCGAGGGAGTTCGGGATCGGAATGATGGAGAGAGACGAGGCTCGGCGCCGGCATAA

>IbWRKY44

ATGTGCAGCCAGTTTAAGCTGCACGGCATGGAGAATTACCAGGGAGATTTAGCGGATATTGTCCGCGGCAGCGGATTAGCCGGAAACGCCGCCCAGGAAGCCGAGCCGCCGGCGCTTCCCGACACGTGGCAGTATAGCAGCGGCGATAACGATAATATCGCGGCGGCGGTTATGAACTGCTACTCCGATCACCGAGATTTCGGGGATCCGTTTTCGCACATGAGAGATCCGATGATGTTCCAGGACCTAGCCATGCCGCCGCCGCCTCCCTCCGCCGCGTTCTTCGCCTGCTCCGATAATGATCTCGCCGACACCGCCGCTGCCGCGGCGGTGTCGGAAACCGCCGCTAGCTCCGCCGTCTTCGCCCCGCCGAAGCTAATTCTCGACGAAGAAATGAAGAGACCGGCCTGCAACATATTCTCCAGAATGCTCCAGATCTCCCCCACTTCAAACCCGCCGCCGCCGGGCGGCGGCGGCGGAATCGCTTGTGGCGAAAACATAATCTCGGCGGCGTCGTCCTCCGGCAACGCGGCGGCGGGGCTGCAGATCTCATCTCCGCGAAATACGGGCTCTATTAAACGAAGGAAGAGTCAGGCGAAAAAGGTGGTGTGTGTGCCGGCGCCGGCGCCGGCGAATAGCCGGCCAAGCGGGGAGGTGGTGCCGTCTGATCTGTGGGCTTGGAGGAAGTACGGCCAGAAGCCAATCAAAGGCTCCCCTTATCCCCGGGGGTACTATAGATGCAGCAGTTCAAAAGGTTGCTCGGCAAGAAAACAGGTGGAACGTAGCCGGACTGATCCAAACATGTTGGTCATAACTTACACTTCCGAGCACAACCACCCCTGGCCCACCCAGAGAAACGCCCTCGCCGGTTCTACCCGATCACAAGCCGCCAAAAACGCCGCCGCCAAATCCTCCCTTTCCCAAACCCCAACCCCTAATGACACCCAAACGGATCCCCACGACACCCCGCCGGTCAAGGAGGAGACCACCACCGCGCCGGAGGACCGCGCCATGCAAATCAACGACTTGGACGATTTCCCGGCGGCGGCCGGCGGGTTCCCCCAGAGCTACAAACCCGCGCTGCCGTCGTCGGACGACCCGCATCACCACCCGGAAGATTTCTTCGCCGATTTGGGAGAGATAGAATCCACCGACCCTCTAAACCTCATGTTCTCCCAAGCCTTCCCGTCCGGACAAGAAAAGAAAGCCGCCGGAATCGCCATTGATGACGCCTTCAACTTCTACGATTGGACAGATTCAACAACAACAACAACAATAGTATACAAACGCTCGGAAAATCCGATTCCGGTCAGAGGGGATTGTAATAAAACAATCCACGTGGGATCTCCCAGGTAA

>IbWRKY45

ATGGAAAGCGCTTATAACGGGGAATACAAGGCTCTACTCAATGAGTTAATCCAAGGGATGGAATGTGCAAAGCAGCTAAGAGTTCATCTCAACTCTGCAGCTTCTTCTGAAACCCAATACTTTTTTCTGCAGAGGATACTCTCTTCTTATGAGAAAGCCCTGTTGATTCTCAAATGGAGGTTGGTAGGGCAATCCCACCCGGTGGCAACACCTCTGCCGGGTGCACCTGAACCTTCCATCTCTCTCGTTGGGAGTCTCGACATTAACAACAACAACAGTTTTAAGGAGCAGCAGGACTACAATGTATCAAAAAAGAGAAAGGCGATGCCAACATGGACCGAACAAGTAAGAGTTGGCGCTGAGAATGGCCTTGAAGGCCCTACAGAAGATGGATATAGTTGGCGAAAATACGGGCAGAAAGACATTCTTGGAGCTAAATATCCCAGGAGCTACTACAGATGCACGTTTCGCCTAATGCACAACTGCTGGGCAACTAAGCAAGTGCAGAGATCTGATGATGATCCAACAGTATTTGACATCACATACAAGGGGGCACATACATGCAACTTAGCTCCCACCACCTCAGTTCCCCCGCTGAGATCGCCTGAAAATCAAGAACTCAAACAAATCCATCACCAGAACGAGAGTTTCCAGGCAATGCAATCAAACCAGATGCTAATGAACCTGAGAGCAAGCCTGAGAGTGAACACAGATGGATTGGACACAAAGGAAACCGCATTCCCCTTCTCCTTCCCTCCAACATTCTCGGACTTACAGACGAGAATCAACACTTCCAGAGCTCACAGGTCGATGACAATGCAGTGGCATTGGGCACATACTCACCCTCCTTTGTTTCCCCCACCACTCCCGAATCGAACTACTTCTCTGCTTCACAGCAACACACAAATGCCTTCAAGGGAGTTCATATATCCTCAGCCAACACATCATCAACAAACTCTCCAATTGTGGGCTTGGACTTGGACTACTCACTTCACCCTGCAACTTTAG

>IbWRKY46

ATGGAAGGAGACGAGCCGCCGCCGCAGTTGATATCCAACCCCAACGACCTGCCGCCGCAACTCTTCTCCTTTCCGTCCACTTCCTTGCAAGCTCCGCCGCCTTCCTTGTTGAGCCCTTTACTGCCGCCGACGATGCAAAGCTCGGCTCAGCTCGGCCCGGACATCGATTGGGTCGGGCTGCTTTCGGGCTGTACTATGGATCAAACGCAGGCGCAGGTGTTGAGTGAAAACGGGAATAAGAGTAAAGGGAAGAAGTTGGTGCAGAGTGGTAAGAAGAATAACTTTCCGCCCAGAATTGCGTTTCATACGAGGAGCAGTGAGGATATTCTTGACGATGGATTTAAATGGAGAAAATATGGGCAAAAATCTGTCAAAAATAGCGCCCATCCCAGGAGTTATTACCGGTGCACACACCACACATGCAATGTGAAGAAACAAATTCAGAGACTGTCAAAGGATAGAAGCATTGTGGTGACAACTTATGAAGGCATTCACAATCATCCTTGCGAGAAACTTATGGAGACTTTAACTCCACTTCTCAAACAACTTCAGTTCCTTCCCAGATTCTAG

>IbWRKY47

ATGATGATGGCTCAGTCCGTTGGTGGATGCCGCCTTGAAGACGGGGCCTTACAAGCCGTCGTTCATGGATCCTCAACCCGTGATTCCGCCGCCGCCGCTGGTTTCGGAGTTTTCGACGGTCGTGGGTCGCCGGACGAGGATTTCGGGGTGTTTGAGGATGTTGTCGGGACGGAGATGACTAGGATTTGGAACAGTGATGAACTGGAAGAGCTTTACAAGCCATTCTACAACCCAGTAGTCTGTGCTTCTTCGCCCGTTTGTTTCCCTAAAGAAGTCAACGAACAACAGGCGGTGGAACTACAACATGATTATCAGATGCAGCCGCCGGCTGCTCCTCCGGCGACGGCGGTTTATGTGCCTAAATATAAAAGAAGGAAAAGTGAGCATAAAAGAGTGGTGCTTCAGGTACCACTTGAAGAGCTTTCTGATGATAAGTGGGCTTGGCGAAAGTATGGCCAAAAACCTATCAAAGGTTCACCATATCCAAGGAGCTATTACCGGTGTAGCAGCTCAAAAGGTTGTCTGGCGAGGAAACAAGTGGAGAGAAGCTGTAGCGAACCTGGGATGTTCATCGTAACCTACACGGCGGAGCACTGCCACAGCCAGCCGACCCGCCGGAATTCCCTAGCCGGGACTATCAGGAACAAGTTTCCTACGCCGGCGGCCTCAAAAAAACCCCAACATAATTATTCAAGTGAAGACCCTCCGGCATCATTCATGTCGCCGGCGTTTTCGTCGGTTGTTTCTCCGGCGACGGTAAAAGAGGAGGAGAAAATGGTGGATGAAAACGAGTACAATATTAATAATGTCAAATACGACGTGGAGGATGATTTCTTTGCTGGGTTAGATGATCTTGATGGACTTATTTCTCACTTTCTCTTCTGCCTGTGA

>IbWRKY48

ATGTCTGAAGACCTAAGAGACTTGTACTACCACCACCCATTTCAAGAAGATGAGAGATCTCACGCCGGCTTTCTGTTCTCCGGCGCCGCCGCCACCTCACAGATTCACAACACTACTACTACTAATAACCCTCATCACGCTTTTCTTGATCCACCTTCTTCATACATGAGCTTCACTGACCACTTGATCGGGGCGACTGAGTTTGGGAGACCCGGTGGTTTCGGGTTCTCGTCCTCCGCCGACGCGGCGGCGTTTTCTGCCGTGAAAGATGAGCAGAAACCCTCGCCAATGAACGCCGGGGATGGCGGTGGTGGTGGTGGTAATAATAATGCTAATGAGACCCCGGTTACTCCCAACTCCTCCATCTCCTCCTCCTCCACCGAAGCCGCCGGCGACGAGGACTCCAACAAGGCTTCAAAGCGAGATAAGCAAGCCGTGGATGCTTCAGAAGATGGAGATGATAAGAAAGAGACCAAGGGAAAGAAGAAGGCGGAGAAGAAGCAACGGCAGCCGCGATTCGCCTTCATGACAAAGAGTGAAGTGGATCATCTTGAAGATGGATATAGATGGAGAAAATATGGACAAAAAGCTGTCAAGAATAGCCCTTATCCAAGAAGCTATTACAGGTGCACAAGTCAAAAATGTCCGGTGAAGAAACGAGTGGAGAGATCGTACCAAGACCCTTCCATTGTTATCACAACCTACGAGGGGCAACACAACCACCACATTCCCACCAATCTCCGGGGAACCATCGCCGGAATGCTGCCGCCGTCTCTGCTAACTCCCTCGCCGCTGCTAGGCGCCGGACCTCCGCCGCAAATCTCCTTTCCCCCGGAGCTCTTGGCCCAAATGACGCCGCCCCACCACCTCTTCGCCGCCCATGCAAACCCCTTCTCCGGCGCCGGCGCCGCCGCCTTCCACCCACAAAACCTAGCCCAGTTCCAGCTGCCGCCGGACTTCGGACTGCTTCAAGATATGGTTCCGTTCTTCAAGCAAGAGCCATGA

>IbWRKY49

ATGGTTGGTAGGTTCGACCAAACCCACCCTTCCCCTCCGGAGCAAGACGACTCCGACACTTCGCCGGAAAACAGCGCCGACTCCCCGGTTTCCGGCGCCGATCACGACACCAAGATCACCAAGGTTTCATTTCCCAAACGAAGGAAATCTGCTCAGAAAAGAGTGACGTCAGTGACAATTAACGACGGAGAAATCAGCATTCCGCCGGCGGATTCTTGGGCGTGGAGGAAATATGGGCAAAAGCCTATCAAAGGGTCTCCCCATCCTAGAGGGTATTATAGATGTAGCAGTTCAAAAGTGTGTCCGGCGAGGAAACAAGTGGAGAAGAGTAGGGCGGATCCTAACGTGTTACTAGTAACTTATTTTTGCGAGCACAACCACTCTCGGCCGGCCGCCAGAAACGCCGCCGTTATGGCACCAGACGCGGCGGTTTCCGACGACTCGAAGCGGCAAACGGCTACAAGCGACCATTCTAGTCAATCAGAAGGCGATTCCGAGGAAAGGGTCGCCGCTAAATGTCACGAATCTCCCCTCGCCGCCGGCAATGACGCCGATTGGCTCTTGAATTTCGAGCCTGCAACATCGTTCGCCATTCTTGACGAGAGTCCCATGATGACCCAAACTAACGTTACCGACGCCGGCGACGCGCCGCCGGCGGTCTTCCCAGACCGGGTAGAAGACGAATCCCTCTTCGCCGATCTCGGGGAGCTGCCGGAGTTTTCCAGGGGATTCCGGCGAGGCTGGGGCGAGAGATGA

>IbWRKY50

ATGGCTGTGGAATTGATGATGGGTTATGGGAATGATGGATTTGCTGTGAAGATTGAGGAGAGCGCCGTGCAGGAGGCGGCTACCGCCGGGCTAAAGAGCGTTGAGAATCTTATTAGGTTGCTTTCTAATTCGCCGGAGTGTGGCGGTGGATTTTCTGATTCGGTGTCGGAGACGCCGCCGGCGGAGGCGGAGTTCCAGGCGGCGGCGTTGGCTAACGCCGCCGAGAGGGCCGGTTGGGAATCAGGCCGGTTTGGAGGGAAGAAAATAGATCAGGAACCGGAACCGGAACCGGCGGCTCCGGCCGCCGATGCATCGGAAAAGCCGCTTAGTGGGTCGAAAATCTACTGCCCAACTCCAATCCAACGGTTACCGCCGCTTCCCCACCACCACTTCGTGAAAAACGGATCGGCGGCGAATGATCGGAAAGAATCGTCGACCACCATCAGCTTCTCCGGCGCCGCCGCCGCCGCCGCGGCTGCCTCTCCGGCGGGCTCTTTCATCTCATCGCTCACGGGGATACAGACAGCCTTCAGCCTTCTCTCTCGTCGGGCTTCCAGATTACAAACCTCTCGCAAGTTTCCTCCGCCGGCGGCCGGCCGCCGCTCTCCACGTCGTCGCTTAAACGGAAATGCAATTCCATGGATGACGAAATCAAAGGTGAAACGAATTGTAAGAGTCCCTGCAATTAGCTTGAAGATGGCTGATATCCCACCAGATGAATACTCTTGGAGAAAATATGGTCAAAAGCCCATCAAGGGCTCCCCACATCCGAGGGGGTATTACAAGTGCAGTAGTGTAAGGGGATGCCCAGCAAGGAAGCATGTGGAGAGGGCATTGGATGATCCAACGATGCTGATTGTAACCTATGAAGGGGAGCACCACCATTCTCATTCCATTACAGACCCACCGGCGGCGATGATTCTCGAGTCATCTTAG

>IbWRKY51

ATGGAAGGAGAAGAGCCGCCGCCGGCGCCGCCGTCTCTGCCGGCGTTGTTGGCAAACGGCGACATTAATATTCAAGACCTCTTCTCGTTTCCCTCGACTTCAGTAGTGGAGCATAATAATCCTTCAATGATGTTGAGCCATTTGCCCATACAGAGCTCTGTTGTTGGGTCTGATAGTATTGACTGGGTTGGGCTTCTCTCCGGTTGCATGGACCAAATGGCGGCGCCGGCGCCGGCGAGCCGGGGAGGTGAAAACTGCGTTCAGAAGAATAAAGGGAAGAGGAAGAAATATGTTCCGCCCAGGGTTGCGTTTCATACACGGAGCACTGAGGATATTCTTGATGATGGCTATAAATGGAGAAAATATGGCCAGAAATCTGTCAAAAATAGCACTCATCCCAGGAGCTACTATCGTTGCACGCACCATACGTGCAATGTGAAGAAACAGATACAACGACTCTCAAAGGACAAAAGCATTGTGGTGACAACCTACGAAGGCATCCACAACCATCCTTGTGAGAAACTCATGGAGACCTTAACTCCTCTCCTCAAACAACTTCAGTTTCTCTCCAGATTCTAA

>IbWRKY52

ATGGAGAACAAATTTGATGACCTCATCATCAAAAGAGATAGTATGGGAATTCCTGTATTTTCCGATGAGATTCCGAGCACCTCTCCGGCGGCGTTGCAGCAAGCTCTGTTGGGTGAAGCTGACAAGACTACTTATTCTTTGGGCTTCTTAGATGCTCAACATAACAACAACAACTACTACAACACTACACCCATACCTAACACCATATTTGATCTCATCATCCACACTCACACTCCGCCGCCTCAACATCAGTCCATTCCGCCACCGCCATCTCATTCCCCCTCCCCGCTGGCTTCTACATCTCTTCTGGAGTCGTCCGAGGTGGTGAACGCTACTCCCCCTACCCCTAATTCTTCCTCGCTGTCTTCGTCCTCCAATGAAGCTACTCCGGCGGCCAACGATGATCAGCACCAGACAAGTAAAACAGTTGAAGAAGATGACGAAGAAGACAAGACTACTAAGAAACAGGTGAAACCCAAAAAGAAGAAGAAGAATGCTCAGAAGAGGCAAAGAGAAGCAAGATTTGCGTTCATGACAAAGAGTGAAGTGGATCAGTTGGACGATGGGTATAGATGGCGGAAGTACGGCCAAAAAGCTGTCAAGAACACCCACTTTCCCAGGAGCTACTATCGTTGCACGGCAGCTTCATGCGGCGTGAAGAAGAGAGTAGAGAGATGGTGCGAAGATGCGTCCATTGTTGTGACCACATATGAAGGTACCCACACCCATCCCTGTCCCATCAAGCACACCGCGGGATCCCTTGGGATTGGGATTATGCCACCTAATACATCCTCCTTCTTTCCTACTACTACTACTACTGCTGCAGCACAAGAAGGTGGAATTACCAGTAGTAGTAGTAGTTATTGTTTCGAGGAGCTGGACAACAATAATGTAATATTAATACCATCTCGCCCATCCTCTCAGCAACTTCACTATCCCCTGCAACACACCAGCACCTATTTCCAGACGCCCACTTATAGTAATAATATTAGCAATGGAGCTGCTGCACTACTTTGGGGGCGTACTGATTCAATCAGCACCACTACTAATTTATTGCCATTGCCACAGGGACTGCCTCCTTCTTTAATTCCAGACCATGGACTTCTCCAGGATATGGTACCATTTCAGATGGTAAAACAGGAGGAGCGCAGCAGCCCAGCCAAGAAAGATATATGA

>IbWRKY53

ATGGCTGTAGAGTTGTTGTCTGTTACAGGAAATAGCGGTTTCGCCGCTAAGATGGAGGAAAACGCCGTCCAGGAGGCGGCGGCGGCTGGGCTTCAGAGCGTTGAGAAGCTTATCAGATTGCTTTCTCAATCTCAGCCGCAAGTTAGTGGGTTTTTCTTCTGGGTCTTCGCCGCCGCCGCCGGCGACGGCGGCCGGGGAAGGCTCCGCGGATTACCAGGCGGTGGCGGACGTGGCTGTGAGGGCCCGATTTGTAACCCTCCTCAGGCTCCGCAACCGCAGAGGAAAATGGATCAGGAATCGGAGCTCGGGGCGTCGGGTCAAACCCGGGTCGTCGAGAACTCCGAGAAGCCGCACACGGGTGCCTCCAAAATGTATTCTCCGCCGCCGATTCAACGATTGCCGCCGCTCCCGCACAACCACCACCACATGCTGAAGAACGTTCCGGCGCCGCCGGCGCCGGACCGGAAAGAATCGTCGACCACCATCAATTTCTCCGCCTCCCAGGCCACATCCTCGCCGGGCTCTTTCATCTCATCGCTTACAGGGGACACAGAGAGCTTACAACCTTCACTTTCCTCCGGTTTCCAGATAACCAATCTCTCCCAGGTCTCGTCGGCCGGCCGTCCCCCTCTGTCCACGTCATCGTTCAAACGGAAGTGCAATTCAATGGACGATTCGTCCCTAAAGTGCAGTAGTGCTGGTGGGTCAGCCTCAGGACGTTGCCATTGCCCCAAGAAAAGGAAATCAAGAGTCAAAAGAGTGGTTAGAATCCCCGCTATCAGCATGAAGATGGCTGATATTCCCCCAGACGATTTCTCCTGGCGAAAATATGGCCAAAAGCCCATCAAAGGCTCCCCACATCCTAGGGGATATTATAAGTGTAGTAGCGTACGAGGATGTCCGGCGAGGAAGCATGTGGAACGGGCACTGGATGATCCGACGATGTTAACTGTAACCTACGAAGGAGAACACAACCATTCGCAATCAATCGGGGACACTCCGGCTTCCCTAATCCTCGAATCATCTTAA

>IbWRKY54

ATGTCTGCTCCTACTTTTCTTGAACCAAACCCTAATTATCTTAATTACAGTACTGCTCCTTTTTCCTCAAACATAATCCCAGATTATGATTACTCATGTTATCAAGATTTAGATTTCTTCTGTGCTGATAATCATCATCTTTTTTCTGATGATTTTACATCGTCCAATGATAATAATACCTTTGGGTACCCATCATCTGATCTGCCACAAACTCCTCTTGTCTTGCCTGAGAAAAGCAATAATTCCACCACTGGAAGCTCAAGCTGCTCATCTGATGGAATGCCAACTTCTACCAATTATATGCCAGTGAAGTGCAAAAGGGAGACGATGAAAGGGCCAAAGATGAAGGAGAAACGTGCGATTGCCTTTAGAACAAGGACTGATCTTGAGGTATTGGATGATGGATATAAATGGAGGAAATATGGGAAGAAGAAGGTCAAAAGTAACTCTCATCCAAGGAATTACTACAAGTGTTCGAGTGGAGGATGCAAAGTGAAGAAGAAGGTGGAAAGAGACCAAGATGATGCAAAATATCTGATAACGACGTACGAAGGGGTACACGACCACGAAAATTTGTATGTAATATACTATCAAGGAATGCCTGCAACTCTTACAAGCAATGGCCTGTCTCTCCCAGCAGCTTCACAGCCTTATTGA

>IbWRKY55

ATGCTCACTTCACTTGGTGATTGCTCAGCTCCCATGGATGGAAACAACCATCATCACCACTACTACTACACTACTCATCATAATGATGACTCCACTTTTGCTCGCCAAGATTCCGGCTTTGAGCTCTCCGAGTTCTTTGACCTCGATGTCTGGCCGCCTGAGGAAGACCCGGTTTTCGCCGTCGCCGGACATCCCCAGAACCCTGGTCAAGCCACGGCGGTTGACGCCGTCATGATTCCTTCAGGCGCCGGAGGCGTCGTCACCTACGCTGGACCTTCTAGTAGCATCACTGATAGTGGCGGAGGCATGGAGAGGACGGCGGCGGGGGTGACTGAAAAGTTTGCGTTCAAAACAAAATCAGATATTGAAATACTGGATGATGGCTACAAATGGAGGAAGTACGGCAAGAAAAAGGTGAAAAACAGCCCAAATCCAAGGAATTACTATAGGTGCTCAGTGAATGGGTGCCCAGTGAAGAAAAGAGTTGAAAGAGACAAAGAGGATCCAAGTTATGTAATAACCACATACGTGGGGATCCATAACCATCAAGGCCCTTAG

>IbWRKY56

ATGGGCAGTTGCTTGGAACACAATTCTCCTTCTATGGATATAGATCTCAATGCTGACGTCAACGAAATTCCGATGAGGGATGGAGAACATGATACGATGTTGGAGGAAGAGTTGAAGGAGACTAGGGCGGAGAACAAGAAGCTGTCGGCGGCGCTGTCGGCGATGTGCGAAAACTACGGCAGTCTGCAGAGTCAGTTACTGGATTTAATGCAAGAGAGATCGTGGAAGAGGAGGAATTCCGACCGGGACACGACAACCTGCGGGAGCTCCGAAATCGGGTACGATGAATCATCGGTATCCAAGAGGCCTAGGGAAATCAGAACTAACATCTCCAGAGTTCACGTCCAGACCGATCCATCCGACACCAGCCTTATTGTGAAAGACGGGTATCAATGGCGAAAGTATGGTCAGAAAGTAACGAGAGATAACCCATCTCCTAGAGCATACTACAAATGCTCTTTTGCACCTTCTTGTCTTGTGAAGAAAAAGGTGCAGAGAAGTGTGGAAGATAAATCCATCTTGATAGCTATATACGAAGGTGAACACAACCATCCCCACCCATCAGAGACAACCCAAACATTTACCTTAGCATCACAAACATCTAATCTAGGATTAGGATTTCACAATAACATACAACAAAGATCATCATGTGATTCCACAATAATGGACACCAAGGAAATACAACAACTCTTGGTTGAGAAAATGGCTTCTTCCTTGACAAATGATCACAGCTTCACTGAAGCACTGGCAGCAGCCATATCCGAGAGGATATTAGACAACCCCTTAGATTGA

>IbWRKY57

ATGGCTGAAGAGTTGAGAGATTTCTACTACCACCAACCCTTTCAAGATGACCGGCACGGCGGCGGCTTCCTCTACTCCGCCGCCCAAGCTTCATCCATGGCGGATTCTTCTCTGCTTCATCATCATCATCATAACCTTGATCCCACAAGTCCGTATATCAGTTTCACCGACTACTTACAAGGATCCAGCGACTTCGAAGCGTCCGCTGGCCTCGGATTCTCCTCGTCGCCGTCGTTTACCTCTGCCAAAGACGGCGAGCGGAGGTCGGTGAATGTGACGACGACGACGACGGATGTGGGCGGCGGCGGTAGTACTGAAACTCCGGTCGTCATGACTCCTAACTCCTCCATTTCCTCCTCCTCCACCGAGGCAGCCGGCGGCGACAACGATGATTCGAAGCACAAGAGAGAGAAGCTGGCGAAGGAGACCGAAGGAGAAGACGACGGCGAGGATAATAATAGCTCCAAGAAAGAGAATAAGGGGAAGAAGAAGGGAGAGAAGAAACAGCGACAACCGCGATTCGCCTTCATGACAAAGAGTGAAGTTGATCATCTTGAAGATGGTTACAGATGGAGAAAATATGGCCAAAAAGCTGTGAAGAATAGTCCTTATCCAAGAAGCTATTATAGGTGCACAAGTCAAAAATGTCCTGTGAAGAAACGCGTGGAGAGGTCATACCAAGACCCCTCCGTCGTGATCACCACCTACGAAGGCCAACACGACCACCACATCCCCACCAATCTCCGGGGAAGCCTCGCCGGAATGTTGCCGCCTTCCATGCTAGCCACCTCCTCCCTGCTCGGTGGCCCGCCCCCACAGGGCGTCACCCTCCCTCCCGAGCTTTTAATGGCCCAGATCAATCCACTTGCCCACCATTTCTACGGTGGCGGCCATAACGCCGCCTCCATGATGTTTCAGCCCCACCAAAATAATCTAACGCAAATGCAACAACTCCACCCTGACTTTGGCTTGCTTCAAGATATGGTTCCCTCCATGATTTTCAAGCAAGAGCCATGA

>IbWRKY58

ATGGGGCTAACCCTTAAGAGCTCAGAAAGCGTTGGGATTTTCATAGAAAAACGGGCAAAGACTCTAAATGAAGTTGACTTCTTCTCCGAGAAGAGACCGCCGCCGCCTGCGGCGGTGCTGGTGAAGAAGGAGATGCCGGATTTGAACGTAAACACTGGGTTAGAGCTTGTGATGGCTAATAATGGCGGAAAAGAGAATCAATCAACCGCAGATGACTGTGCGGCGCCGGAGATGGAACGACGGCCAGCTAAGAGTGAGGTGGGAAGATTGCAAGTAGAGCTAGAAAGAATGAAGGATGAAAACCAGAGATTGAAAGGGATGCTTTCTCAAGCCACCGACACCTATGGCGCCTTGCACATGTATTTTCTCACTCTGGTTCAGCAGCAACAACAACAACAGACCTCAACAACATACGAGCCATGGAAAATAAAGATGCTAATCGTTGTTGTACGTTCAGGTGGCGGATGGAAAGAGAACAGTCACAGCAAGACAGTTCTTGGACACGGCCGCCGGCGCCGCCGCTGGCGGCGGTCAGACAGACGAGGCATCCCACAATTCTCGTACCTCGTCGGGGAGAGAACGGAGTCTGAATCGCCTGGGAACAAGGTAGAAGCACCGGTGTCGAGGTCGAATGAAGGGAGTATTGGTCGTAGTAGGGAAGAAAGCGCAGATTCAGAAACATGGCGCCCTAACAAAGTTCCCAGATTGAATAATAATCCTTCAAAACCTGTTGGTGTTGATGATGAAGATCAAGCTGCTGCTGCTGCTTCTGCGGCGGCGGCGACGATGAGGAAAGCCCGTGTCTCCGTCCGTGCCCGCTCGGAAGCCTCCATGATTAGTGATGGATGTCAATGGAGAAAATATGGGCAAAAAATGGCGAAAGGAAACCCATGCCCACGAGCCTATTACCGACAACCTACGAAGGCACACACAACCATTCCCCTACCGCCGGCCGCCATGGCGATGGCGTCGACCACCTCTGCCGCCGCGAATATGCTTCTCTCCGGCGCGGTGCCGAGCGGTGATGTGATGATGAACCCTAATTTCTATGGAAGAGCCATTTTTCCCTCTGGTAGCATTGCTACAATTTCTGCTTCTGCACCTTTTCCTACTATCACATTGGATCTTACCCATCAACCCCTCAATTCTTTGCCTAATTACCCAAGACCCCCAATTGCCCAATTCCCTTTCTCTAATGCCCCTCAAAATCCTCAACATTATGTTTCGACCCCTCAAGTTTTTGGCCAGGCAGGTTTGTATAATAACCACTCAAAGTTTTCAGGGCTACAAGTTTCTAATACCCTTCAACACCCTTCGTTTGCTCATGGGGGCGCCACCACGGCCGCCGCCATCACTGCCGATCACAACTTCACCGCCGCCCTCGCGGCCGCCATCTCCTCCATCATCAATGGTTCACAGCACATTCCTGCCCACAACAACCCTTCAAGCAATAGCAACCAGACTAGTAGTTTGCCGGCGAAATAA

>IbWRKY59

ATGATGGAGACAAGGCTTCACCACAACAGCTCCTCGTGTTTTCACAAGCAAGAAGAGCTCTCCGCCGGAACGCCGCCGGAGAATGGGGTTGAGTCGCCGTTGTCCGGCGATGAAGCGGCGGAAGTTAGCACCCCGTCCCCTAGGAAAAGGAGAGGCGCACAGAAGAGGGTGGTTTCCGTGCCGGCCGGCGACGGCGACGGATCACGGAGTAAATCCGAGGTTTATCCCCGCCGGATTCTTGGTCGTGGCGAAAGTACGGCCAAAAACCCATTAAAGGCTCCCCTTATCCCAGCTAGAAAACAAGTTGAACGCAGCCGCCTGGACCCCACCATGCTCCTCATCACCTACTCCTCTGAGCACAACCACCCTCACCCCACCAAAATCCACCACCACTACGCCGCCGGCACCGGAACCGGCACCGGCGCCGCAACTTCTTCCTCTTCCGCCGCCACGACACCGACTGATTCAGCCGCGGACCCCGGCTCGCCGCCCAAGCCGGCTCCCAAGGAGCTGCCCATCTTCGCCGACCCGGACCCGGAGAACGACACCTTCCCGGAGCTCGCCGGCGGCGAGATGGGGTGGTTTTCCGACGTGGGGCCCTGCTGTTTTATGGAGAGCGCCACGGCGGTTGCAGGGCCCACGCTGTGCCACGACACCGACGTGGCGCTCACGTTGCCTATTAGGGAAGAGGACCAGTCGTTGTTCGGCGATTTGGGCGAGTTGCCGGAATGTTCGGTGGTTTTCCGGCGGTGTAGAGTGGAAACGCCGTGCTATGCCGGCACCGGATAA

>IbWRKY60

ATGGCGGAGAATCACGCGGGACGAGCGTCGAAGTCGTCGTCGTCGTTGAGGTTGGCTTCGGCGCGGCCGAGTATTACCTTGCCGCCGCCGAGTCCAATATTCACCGGCGGAGTGAGTCCAGGGCCGATGACTCTCGTCTCGAGTTTGTTCGGCGAGAATGATCAGGAGTCCGAGTGCCGCTCATTCTCTCAGCTGCTAGCCGGTGCCATGGATTCTCCCGGAGCTCGGCCGGCGAGGAGAGTGGATCCTAATTCAAAGGAGGAGGAAGTTTTCAGGCATAACGGCTCGGCAATAACTCAACCGTCAATATTTACTATACCGTCGGGTTTAAGTCCTGCTAGTTTGCTAGATTCTCCTGGCCAGGGCCATTTTGGAATATCTCATCACCAAGCCCTTGCTCAGCTCACAGCTCAACCAGGCATTCAATCTGACCATCCATCTTCATCTTTGCCTCCTGCACCCCAGTTTTTTCAGCTTCAGATGCCTCTTCCTGGTAGAGATCCTAACAGCATTAAAGAATCATCTAATGTTTCACATTCGGATAATGTATCAGAACCTTGTTCTTTTCCTGTTGATAAGCCGGCTGATGACGTGAAGAAGAAGGTTGAGCGTTCTCTTGATGGTCAGATAACCGAGATTATATACAAGGGCAAGCACAACCATCAGCCACCTCAACCCCAAGGGGCGAAGGATACTGGAAATCAAAATGGCCTTCAAGGCAGCTCTGAACTGAATCTCATGGATGGAGTTCCCTTTCAATCATTACCAATGAAGGACCAAGAATCTAGCCTGGCTACTCAAGAACACATGTCTGGATCTAGTGAAAGCGAAAAGGTGGGTGATGCAGAAGCTAGGATGGATAGAAGAGATGATGATGATGAACGAGAATCAAAGCGTAGGGCCACGGAAGGGCAGATCCCCGAGCCAGTAACATCACATCGGACAGTGACAGAACCAAAGATCGTTGTTCAGACTACTAGTGAAGTTGATCTTTTAGAGGACGGATATAGGTGGAGAAAATATGGCCAGAAAGTTGTTAAAGGGAACCCTTTTCCAAGGAGCTACTATAAATGTACCACTGCTGGATGCAATGTGCGAAAGCACGTCGAAAGGCTTGCAAGTGATCCAAAAGCCGTCATAACAACATACGAGGGCAAGCATAACCACGATGTACCAGCAGCTAGGAATAGCAGCCACAACACGGCCAATAACGGCCCCGCGTTACAGCTGAGGCAGCACAACCCTGCTGCAGTTGATAATCAGGCTGCACTCCTGCAATTCAAGGAAGAACAAATTACATGA

>IbWRKY61

ATGGATGATAAGGGGAAGTTCGATCACCACGAGTTCACCGCCGATTCGAGCGGCTGGGCGCTGCGAGGGGACGCCGACGGGGCGTACTTCTTCGGGGCCGGCGGCGTGGATAAGGAGGAGAGCAGCATCCTGAGCGACTTCGGCTGGAATTTTCAACCGCTCGACGGAATTTCCGGCGGAGGCGGAGGAGGAGGAGGAGTTGGAGCGTTCGATCTGATCCACGCGGATTTGGCGGGAAACGGTTGTGTTTCTGGTTCCGGCGGTGCTGATGAGAGCCCTAGCGGCGATGGAGAGGCGACCGCGACGCAAGCCGAGCCGGCGACTTCCAGCTCCTCAGAGGAAGCGGCGGCGGCGGAGAAGCCGTCCTCGTCCTCCGCCTCGCGGCCGCCGCCGCCGCCTGCGGACACAACAAGTAGCAAAGTGAAAAAGAAGGGCCCGAAGCGAATCAAGCAGCCGCGGTTTGCATTCATGACTAAAAGTGAAGTTGATCACCTTGAAGATGGGTACCGGTGGAGGAAATATGGTCAGAAAGCCGTCAAAAACAGCCCCTTTCCCAGGAGCTACTACCGCTGCACCAACAGCAAATGCACGGTGAAGAAGAGAGTTGAGAGATCCTCTGAGGATCCCACGGTGGTGATCACAACGTACGAGGGGCAGCACTGCCACCACGCTGTCGGCTTTAGAGGCGGGTTCATAGGCCACGAGGCCTCGGCATATATGGCCCGGTTGAACCCTTTAGCCGTGCAGTCATATCTCCCCGGGCTCACCGCCCAATCACATACTCTACCCATCCAATCACATCAAAACCTGACACACAACATCGAATCCCATGACCCAAGTCGTCGCCCTACACCACCACAACTCCCTCCAGATGAGGGATTGTTAGGCGACATGGTGCCTCCTGGGATGTGGAACAAGTAA

>IbWRKY62

ATGGAGTTTACTAGCTTGGTTGATACTTCGTTGGATCTAAACATAAAACCTCTCCGGCCGGCCGGCGACGCCGGAGTACCGCCGAAGCAAGAAGTGGAGAGCAATTTCATTGGGTTGGGCATCAACATGGCAATCAAAAATGAGGCGGATGGTCTAGTGGAGGAGTTGAATAGAGTGAGTGCGGAGAACAAGAGACTGACGGAAATGCTGACAATCATGTGTGAGAAATACAATGATTTGAGGGAGAAATTGAAGGGGTACATGATGAAGAACAATGGATGTGAGGACAACAGTAGCCCTGTGGGTGTTCTGGGATCAAGAAAGAGGAAATCGGAGAGCAACAACGTTAATAATGGAGGACAACGCTCGGAGAGCAGCTCCAGTGATGAAGATTCTGTCAAGAAACCCAGGGAGGAACAACAACAACAACCACAGCACATCAAATCTAAGACCTCCAAGGTTTATGTCCGGACGGAATCATCTGATACCGGCCTGATTGTGAAGGATGGGTATCAATGGAGGAAGTATGGGCAGAAAGTAACTAGAGATAATCCATCGCCCAGAGCTTACTTCAAATGCTCTTTTGCTCCCACCTGCCCAGTCAAGAAAAAGGTTCAGAGAAGTGTGGAAGATCAGTCGGTTTTGGTGGCAACCTATGAGGGAGAGCACAACCACCCACACCCCTCAAAGATGGATCAATCATCCACTCCGCCTGCCCGATCTGCTCCGGCCCCTAGCACCACCTCAGCCCTCACCACTCTCAACACCTCAGCAGGACCAACCATCACCCTTGACTTAACAGACCCCAAACCCAAACCATCATTACCCATCACCGCCGCCGCCGCCAGAGTATTGCCGGCGCCGGCGGACAGACCGGACTTCCACCAATTCTTGATAGAACAGATGGCTTCATCCTTGACAAAAGATCCCAGCTTCAAAGCAGCTCTTGCAGCCGCCATTTCCGGAAAATTAATCATTCCTCATAATCAGACAGAAAAATGGTAA

>IbWRKY63

ATGGAGGAAGACAAGAAGAAAGCTGCAGATTCAAGTGGTGATGATGAAGGTTATTGCACTCATGAGATAGGAGATGGAAATAAAGAGAACGAGCGTGACACTTTGAAGGCATCTTCATCACCCAACCACAAAACTCTCAGTTCAGACAAAGAGGTATTGAAAACGAGCTACTCGAGTCAGCAAAGGCTGAAATGGGGGGAAGTGATGGAAGAGAATCAAAGGCTGAAGATGTATTTGGAGCGTATCATGAAGGATTATCGGACGCTTCAGATGCAATTCCAAGGCATGGTTGAAAAGGAAGGAGAAAAAGCAGCAAAGAGCGATGATAATACTCCCCAAATAACCGAAGAATCAGAGCTAGTGTCCCTTAGCCTGGGAAGGGCTTCAGCTGAGATGAAAAGGGAAGAGCAAAACAGACCAGTAATAGTGTGTGCTGGGAAAGATAAGGTGGATAATGAAGATAATGATCAGAAAGAGGGCTTAACTCTTGGCTTGGACTGCAAATTCAAATCTTTGCAGCCCAATAATCCAAGCACAGATAACAGTTCAGATGAAGTGAAAGAAGAAAATGGGGAGACTTGGCCTCCAAGTAAGGCCTTGAAAACTATGAGAAGTGGAGAGGATGAAGTTTCACAACAAAACCCTGCTAAAAGAGCTAGGGTTTCTGTTAGGGTCCGATGTGATGCCCCAACGATGAATGATGGGTGCCAATGGAGGAAATATGGACAGAAGATTGCGAAAGGGAATCCGTGCCCTAGGGCATACTATCGCTGCACTGTGGCTCCATCCTGCCCAGTAAGGAAACAGGTTCAAAGATGTGCGGATGACATGTCAATTTTGATCACAACATACGAAGGAACACACAACCATCCACTTCCACTTTCAGCCACAGCAATGGCCTCCACCACTTCCGCCGCCGCATCCATGCTAATGTCCGGTTCATCAACCTCCGCTTCCACCTCAATGCCGCCCGGAACCACCACCACAACCGCCACCTCCACCTCCACCACCAATCTCAACGGACTAAATTTCTACCTTTCCGATACCTCAAAACCAAAACCCTTCTATATCCCAACCTCATCAATCACCCCAACCCTAGGACACCCCACAATAGTCCTTGATTTAACCTCAACCGCTCCCTCTTCCTCATCAAACCTAAGTAGAATAGGAAGCTTAGCAAATTTCCCTCCAAGATTTTCCTCTACAAATCTCAACTTTAGTTCTTTGGAATCAAACCCTCTCCCCATTTCTTGGAACCTTGGAACTACTCAACCTTATAACAAACCCCACATAACCCAATCTCTTACCTTTGCAAGACAACAACAACAACAACCTCAAGACAATCAAATTCTTTTCCAATCCTATTTGTCCAAAAACAACCTTAACAATAACAACAACAATCCTTCACAATCTCTCCCACAAGAAACCATTGCAGCTGCAACAAAGGCGATCACTTCCGACCCAAACTTCCAATCCGCATTAGCAGCTGCCCTTTCATCAATCATGGGTACTAACAATGGAAACAATGGAGCATCCTTAAACCTCGGAATCCACGGATTAGCGGAGAAATTAAGCCACAACTTGAAGGCGGCGGCCGAGCCTTTTCCGATCCTTTCTAGCTTTTCTCCATCTCCAAATCCAAATAAATGCTCTTCGAGTTTGTTGAGTAGGCCGACAACGACATCCTCGGCAAATCCTCATCCAGGGAATTTGATGTATCATCTCCAACAAACCTCAAAGAGTAAGTCTTCCTCTCCTGGTGATAGCAGAGATCAAATAATTTGA

>IbWRKY64

ATGGAAGATTCTCAGTCCCACTCCCACTCTCACTCTCAGTCTCACGCGCAGCACCACTCCGGTGAAGACCTCGATGCTCTCCAGAGCAGTCGTGTGATTCACGCCGCTGATGGAAGCTCCGATGCCTCCGACGCCGTGTTCTCTCGCGGCGTCGCCGGATCCAACTGCGGCGCTAGGTACAAGCTCATGTCTCCGGCGAAGCTCCCGATCGCGAGGTCCGCCGGTATTACCATCCCACCTGGCTTGAGTCCGACTTCGTTCCTCGAATCTCCTGTTCTTCTCTCTAACATCAAACTTCAGATTCGGATTCGGAAGAAAACTTCTTTATGGTTTCCTCTTCAGAGGGAAGGAAGAGTTAAGGCAAAAGGGGCAGAGCCTTCTCCCACCACAGGATCTTTCTCAAATCTTCATTTGATGCAAGGCTCTGGTGGCTCTGCTGCATTTTCATTTCCGGATAGCAGAGCTTTTAGTCAAAGAAAGTCGAGCAACTTTGAGTTTAAGTTTCCCATTGGATCAAGTTCTACATCTGCATCAGCATCAATAGAACCTATGACATCTGCAGGCTTGAACCAGCAGCAAAGTGAAACTCAGAGACAAGTTCAAAATCAATTCATTTCTCAGTCATTGGCCACTTCATCTGTGGTTGAATCTTCAATTCCTACATCAAATGAATTGAACTTATCTTCAGCTGTTGCACTGCATACTTCGTCTGTTGGTACTGATGCAATTGAGTCTGATGACCTAAATCAGAGGGGGAAAGCAGTGAATGCTGATCAGTCTTCTGTCACAGCTGAAAGATCATCAGATGATGAGTATAACTGGCGAAAGTATGGCCAGAAACTTGTAAAAGGAAGTGAATTTCCACGGAGCTATTACAAGTGTACATATCCAAATTGTGAAGTGAAAAAGATATTTGAACGCTCTCCTGATGGAAAAATAACAGAGATTGTATACAAGGGTTCACATGATCATCCTAAACCCCAACCCAGTCGTAGATTTACTCCTGGTGCAATGATGCCCATCCAAGAAGATAAATTTGAGAAAGAATCATTTTTCAATGGTCAAGAAGATAAGTTCAACTCCATTGCCCAGACTGGTCGTCCAGAACCAAGTGGGATCCCTATTCTTTCACCTCAACGAGTAGGTGATGATATCCATGAAGGTGCAACTTTACTACTGCAGGGTACTAATGATGACACTGATGAGGATGATCCATTTTCAAAAAAGAAGATGGATGGCTGTGTTGATATCACACCAGTGGTTAAGCCTATCCGTGAACCACGTGTTGTTGTTCAAACTGTGAGTGAGGTTGATATATTGGATGATGGCTATCGGTGGCGTAAATATGGACAAAAAGTTGTCAGGGGGAATCCAAATCCCAGGAGCTATTACAAGTGCACTAATGCTGGATGCCCAGTCAGAAAACATGTTGAAAGGGCTTCCCATGATCCCAAAGCTGTTATAACCACATATGAGGGAAAGCACAATCATGACTTACCAGCAGCCAGGACTAGCAGTCATGAAATGGCAAGCACAGCACCCGAAAGTGGAATATCCAGAGTCAGGCCAGAAGAGAATGACGTGATTAGCCTTGATCTAGGGGTTGGTATTGGCTATGGCACTGAGAACAGGACCAATGACCGGCTTCATTCACTGGCTCCAGAAACTGTCCCAACTCAAGTTCTAGCTTCAGGTGGTGGGATGATGGCAGTTCAAGCACCAGCTATTGTGCGCTATGGTATTGTAAATGGTGGTATCAACCGATTCGGGGTTCTGACTGTCATATCTGAAAATGGTTTTCTAGCTGATTTGCCATTGCCAGAACATGAAAAGCCGAGGCTGGCAGATGTAATTTGCCACCTACCACAACCAGCTAATTGCAAAGGCAGCCACTTTCACAAGTCGGAAACTGGGCTCACTCCGCTCACTCCTCAACGAATCCTGTGTGCCAAGCAATATCTTATATCTACTATGCAGAAGCAAAAACGGTTTCATGGTAACTATCCTACACCTCTGCCCTTGTTTTCAAAACTGGAGGCTGGAAACCCATCGAGGAACATAATTGAGATGATCTTTCGAGCTGCTTCTAAAAATCCATCAAAGCATCCATGGTTAATAAAGAGAGTCATAAAAGTGAAGAACACAATAGATGTCTTGGATAGATTTGAGAAGTACAGAGAGGCGGTGAAGAGCAGGCTAATTCAATCTGGTTTCAACACTTCATACAACAACAAAAATGGGATCCGGCTCAGTACAAACAGCGATACCCTTAGTGAGGATACAACCGTAATCTCTAACAGGAACAAAGCCAAGAGGGCAGTGATAGTTTGCCGCACCATTGCTGGCATAGTAGAGAAGGATCAAGACTTGCTCGAAGAAGAGCACGATTCAGTTGGACATGGAGTGGATACAAAGCTAGAATATTTAACTGTAAAAGACCCTAGTGCTGTACTCCCGTGTTTTGTTATAGTTTTCTGCTGA

>IbWRKY65

ATGTCTGATAACCCATTCTATTTCCATAATCACATGGGAAGTGGAAGGATCAATACATTCCCTTTCTTCGGCGATGATAACTCCGACCACAACCCTTCTTCTATCTACTCTTCCTCCGATCATCACCCGCCGCCGTCTGCTCCGACACAGAATCTCCTCCACCAAGAATTCCTCCCTTCTCCGTTCATGAGCTTCACCGAGAGCCTACAAGGCTCCATGGATTACCACACTCTCTCCAACGCTTTTGGGATGTCTTGCTCCTCGTCCGAGGTTGTCTGCACTCCGACAGATCATCATCATCATCAGCAACAGCAGCAGCAGAACCAGGAATCTTCTAGAAAGAGCAGTGTTTCTGCGGGAGAGGCGGCGGGGGAGAATATTCCGTTTGTGGCGGCGAATTCATCGGTATCTTCCTCGTCCAGTGAGGCTGCAGTGGGAGACGGAGAAGAAGATTCCTCAAAGAGCAACAAAGATCTGTTACTGCCCAAAGGGTGCGAGGACGGAGACGACAAGTCCACAAAAATAAACAAAGGTGCAGCGAAGAAGAAAGGGGAGAAGAAGCAAAGGGAACCAAGATTCGCCTTCATGACAAAAAGTGAGATTGATAATCTTGAAGATGGATATCGCTGGAGAAAATACGGACAGAAAGCTGTCAAGAACAGCCCCTTTCCCAGGAGTTATTACAGGTGCACGAGCCAAAAGTGCACGGTGAAGAAGCGTGTGGAGAGATCATACGAAGACCCAACAATCGTGGTGACCACCTACGAAGGGCAGCACAACCACCACTGTCCGGCGACTCTACGCGGCAATGCGGTGGCGCTGCTGTCCCCAGCGTCATTCTTGTCACCATCTCCGGCCGCACTCATGCCCAACTTCCATCAAGACCTCTTGCTCAATCCAATGCTCTCCGGCGCCCCAAATTTCCAGCCTTCTTCCATGTACGGCGGCTATCATCATCACCATCTTGGCCTCAACCCTCACCACTATGATCATCAGATCACCCAATCGCCAGTCGATCAGTACACTTTATTCCAGGACATGGTTGTCTCATCATTGGGCCATAAACAGGAGCATCCATGA

>IbWRKY66

ATGGCCTCACCTAGTGGACTCTCCTTTGATCCAGATCCCATCAGGCCTCCCCACCCCACAAACCCTCACCTTCTTCTGAACCCACCCAAAACCACCGCCATGAACCCCCACCGCCACAAGTTCATAAAGCTCGAGCCTTTTATCCATGGAGTCTCCGGTCAACAACAGATCGCCTCCCCACTATCCAATTCCCGGGCCTCCGCCGTTGATGACGACGTTAAAGATTTGCGCACCTCCACTGAATTGGATTTCAGTATTAACACGGGTTTGCATCTTCTCACGGCGAACACCAATAGTGATCAGTCAATAGTGGAAGATGAGTTGTCACCGAATTCAGAAGACAAAAGAGCTAAAAGCGAGCTGGCGGTTGTGCAAGCTGAACTGGAGAGGATGAACGGCGAGAATCGACGGTTGAGGGACACGTTGAATCAGGTGACCAACAATTACACTTCTCTCCAGATGCATGTGATGACGTTAATGCAACAACAACAACAGCAAAGCCATGGGAAAACGGAGGAGTCTAAGCAGACCCGCCAAAATAATGGAGGCCAAATGTTGCCCAGGCAATTCATGGATCTCGGTCTTGCTGCCGGAGGAGGAGGAGGGCCCACCGAGGCCGATGAGGCGTCGCTGTCATCGTCGGAGGGGCGGAGCGGCCGGGAAGGGTCGCAGTCGCCGACCAACAATTTAGACGAGGCGTCCCGAGCGGACAGCCCGGAAAAAGGGTCCGGTTGGAGGTCTAATAAGGTCGCTAGATCTGGGCATGCTTCTAAAAGCGGTAACATTGATCAAGCCACCGAGGCCACCATGCGAAAGGCTCGTGTCTCCGTTCGAGCTCGATCCGAGGCTCCCATGATTACAGATGGTTGTCAATGGAGGAAATATGGCCAGAAAATGGCCAAGGGAAACCCGTGTCCTAGAGCTTATTACCGGTGCACCATGGCCGCTGGTTGCCCAGTTCGAAAACAAGTTCAAAGATGCGCTGAGGATAGGACAATCTTGATCACAACTTACGAAGGGAACCACAACCACCCACTGCCGCCGGCGGCCATGGCAATGGCATCCACGACCTCGTCGGCGGCGAAGATGCTGTTGTCGGGATCCATGCCGAGCGCCGATGGGCTGATGAACTCAAATTTCCTAGCGAGAACCCTCCTCCCTTGCTCTTCAAGCATGGCCACAATCTCAGCCTCCGCTCCATTCCCCACTGTTACATTGGATCTCACCCAAAACCAAAACCCAAACCAATCCCAATTCCCCAGACCCCCAAACCCCTTCCAATTCCCATTCCCAAACCCGTCCCACAACCCCGCCGCCGCCCTTTTGCCTCAGATCTTCGGCCAGGCGTTATACAACCAATCCAAATTCTCCGGCCTTCAATTATCTCAGGATTTGGAAAACATCCACGCGCCTCCTTCCTCAATGCCACCTCACCAATCCTCCCAGCAGAACCCGCTGGCGGACACCGTGAACGCCCTCGCCAGCGACCCCAACTTCACGGCGGCGCTGGCCGCCGCCATTACTCCCTCATCGGAAACCCCTCCCATTCCGGCAACGCTTCTAACGCCTCCAACGCCAATAATAACGGCAGCGTTATTACCAGCAGCAATGGCAATGACAAAGTCAACAGTTGAAATTCTATCTCCAAACCCAAACTGCTATACATATTAA

>IbWRKY67

ATGGAGTCTCCGGTCAACAACAGATCGCCTCCCCCCACTATCCAATTCCCGGTCAACCGTAACTGCTCCGACCATCGTCGCCATGCCCATGACCAAGAAGACCACGATCATAAAACGACCGTCTTAGGTGAGATGGACTTCTTTGCATACACCAAGGATAATAATAATGGAGATTCCAGGGCCTCCGCCGTTGATGACGACGTTAAAGATTTGCGCACCTCCACTGAATTGGATTTCAGTATTAACACGGGTTTGCATCTTCTCACGGCGAACACGAATAGTGATCAGTCAATAGTGGAAGATGAGTTGTCACCGAATTCAGAAGACAAAAGAGCTAAAAGCGAGCTGGCGGTTGTGCAAGCTGAACTGGAGAGGATGAACGGCGAGAATCGACGGTTGAGGGACACGTTGAATCAGGTGACCAACAATTACACTTCTCTCCAGATGCATGTGATGACGTTAATGCAGCAACAACAACAACAACAGCAAAGCCATGGGAAAACGGAGGAGTCTAAGCAGAATCCGCGCCAAAATAACGGAGGCCAAATGTTGCCCAGGCAATTCATGGATCTCGGTCTTGCTGCCGGAGGAGGAGGAGGGCCCACCGAGGCCGATGATGCGTCGCTGTCATCGTCGGAGGGGCGGAGCGGCCGGGAAGGGTCGCAATCGCCGACCAACAATTTAGACGAGGCGTCCCGAGCGGACAGCCCGGAAAAAGGGTCCGGTTGGAGGTCTAATAAGGTCGCTAGATCTGGGCATGCTTCCAAAAGCGGTAACATTGATCAAGCCACCGAGGCCACCATGCGAAAGGCTCGTGTCTCCGTTCGAGCTCGATCCGAGGCTCCCATGATTACAGATGGTTGTCAATGGAGGAAATATGGCCAGAAAATGGCCAAGGGAAACCCGTGTCCTAGAGCTTATTACCGGTGCACCATGGCCGCTGGTTGCCCAGTTCGAAAACAAGACAATCTTGATCACAACTTACGAAGGGAACCACAACCACCCACTGCCGCCGGCGGCCATGGCAATGGCATCCACGACATCCTCGGCGGCGAAGATGCTGTTGTCGGGATCCATGCCGAGCGCCGACGGGCTGATGAACTCAAATTTCCTAGCGAGAACCCTCCTCCCTTGCTCTTCAAGCATGGCCACCATCTCAGCCTCCGCTCCATTCCCCACTGTTACATTGGATCTCACCCAAAACCAAAACCCAAACCAATCCCAATTCCCCAGACCCCCAAACCCCTTCCACTCCCATTCCCAAACCCGTCCCACAACCCCGCCGCCGCACTTTTGCCTCAGATCTTCGGGCAGGCCTTATACAACCAATCCAAATTCTCCGGCCTTCAATTATCTCAGGATTTGGAAAACATCCACGCGCCTCCTTCCTCAATGCCACCTCACCAATCCTCCCAGCAGAACCCGCTGGCGGACACCGTGAACGCCCTCGCCAGCGACCCCAACTTCACGGCGGCGCTGGCCGCCGCCATTACTTCCCTCATCGGAAACCCCTCCCATTCCGGCAACGCTTCTAACGCCTCCAACGCCAATAATAACGGCAGCGTTATTACCAGCAGCAATGGCAATGACAAAGTCAACAGTTGA

>IbWRKY68

ATGCAATTTCCGGCGGCGATGGAGGATGAAAATTTTGTGTTTAACTTAGTGATAGACGATGATAATAGTCCAGATGAGATGAAGTCCAGCATAGGTGAATCATTTCCTTCAACCTCCGCAAATGAAACGCATTCTTCTTCGCCAATGCAGATCATTCCTTTCGATTTCCCGATGGAATCGTTGGATTGCGGAGTGGACATGTCATCGGCCGTCGACACTAACCCTAATTCCAATTCCAATCTTATGTTACAGTCGCCGGTTAATGGAGAATGCATCAATAATAATGTTAATGGAGAAACCTGCAGCCCTTTTAATAATCCGGAACCGGAGTTGGATCACCGGAGAAGCGCCGCCGCGGCTCCGGCAGCTAAATCCCTAGCTAATAATAATAATAATTCTGCCGGCAGTTCGTCCGACGACGGATACACGTGGCGGAAATACGGGCAAAAGCACGTGAAAGGGAGCGAGTATCCGAGAAGCTATTACAAATGTACGCATCCGAAATGCACGATGAAGAAAAAGGTGGAGCGATCGCCGGACGGGCAGATCACGGAGATCGTGTATAAGGGCGCGCATAATCACCCGAAAGCTCCGGCGACCAGTCTCCGGCGATCGCCGCCGTCGTTAGGAGCGGAAAGCTCTTCGTCGGAGATGATGAGCCAAGGGAGCGGATCTTGTTTCAGATCTCAGGCTCCGATTTGGGCAAACATTCACCACTATGGCAGCATGCCGGAAAGATCTGCGTTGGCTTCGAGCAGTGATCTGACGGCTGAGATTTGCGATCCGCTGTCGTCGTTGACGACGAGATCTGCGGCGGCGATGAGTGGATTTGAATCGGCAACAACCCCTGAACCTTCATCTACACTTGCTAGTCAAGATTGTGATGATAATGAAGATGCAGTTACTCAGGGGATCTCCCCTTCCCAGTTTGGTGAAGATGGAGAATCCGAGCCCAAACGAAGGAGAAAAGATGGGTGGTCAATTGAGGCAAATTTGTCAACAAGATCAATCCGGGAACCCAGAGTAGTGCTCCAGATTGAGAGTGAAATTGATATTCTTGACGATGGATATCGCTGGAGAAAATATGGCCAAAAAGTTGTCAAAGGAAATCCAAACCCAAGGAGCTATTACAAGTGCACAAGTCCCGGATGCCCGGTGAGGAAGCACGTGGAGAGAGCTTCCGACGATTTGAAATCCGTAATCACTACTTACGAGGGGAAGCACAACCACGAGGTGCCACCAAACAAGGCGGCCGTTGTAAACTACAACAGCTACAGCGCCACCTCGGGCACCACGGCCAGCTCAGCCATGCCGAGAGCTCCGGCTCTCGGCGGAGTGGGCGTGCAAGATCACCACCCATCGTTCCCATTTGAACGGAAACCCATGATTGCAGGCGCCGGAGGGGACGAGCTACTAAGGCCGGAAATGCTCGACTGCTATGCCGCCGGCGACTTCAGGTTCGTGCCATCCTCCATTTACCCGCTCAAATTCCCTCCTCCGCCGTTGCAGGGCCCGCTGACAGCGGCCGCCGCCACCTTCAACTACAGCCGGCCTCCGGGCATGGTGCTGCCGGAATTCCCCATGCCATTACTCCCAATGAGCCTGCCTCCATTCCATGAACTCACTAATTTACCTCCTCTTGCTGATTTTCTGCACTTCAATGACCCTAGCACAAAAGAAGAGCACAAGGAGAATGATCCTCACACATCATTACTATATGAATGA

>IbWRKY69

ATGAACACCTGCAGTAACAACAACAACAACGAAAGCCCTCACGACCACATGGATATCGATTTGTCCTTGAAGCTCAACGAATCTCCTCAAGATTCCCCGCAGCCCTGTCGGAGAATCATCATCACAACCGAACAAAGGAGATTCCCCATCGTCGTCCAAAAATTCCAAGACAGAAGAGTGCTGCAAACGGAAATGAAACGCATGAAAGAGGAGAACAAGGTATTGAGGGAGGCAGTGGAGCACACCATGAAAGATTTCCTCGATCTGCAGACCAAACTTGCACTATTTGTCCAACAAAACGATCACAAAAAGGACGTTATTAGCAATTTTCTTTGGGTGAATGGGGAAGAAGAGAAGATTAGTCAAGAGCTGAACAGGACATCATCATCGTCATCGCCGACCGCACAGCTGGAACATGATAAAAATAATAATAATAATAGTATTAGTGATACTGAATTAGGTTGTCGCTCACGCTGCAAACCGCCGCCGCCGCAGATGATCATGAGAAAGGAAGAGATCAGAAGAGTCCGCCATCGCCGCCGCCGTCAATACACGGTAATATTCACTCATGGAAGCAGCTTTGCTGCAGGCTTATGCCAAGAATCCCCCATTTCTCAGCACAACGTCAGAAAACCTAGGGTTTCGGTCCGGGCTAGGTGTGAATCTGCCACCATGAATGATGGTTGTCAATGGAGAAAATATGGGCAAAAGATTGCTAAAGGAAATCCTTGTCCAAGAGCCTATTATCGGTGCACGGTAGCTCCGGGATGCCCCGTAAGGAAGCAGGTTCAAAGATGCATAGAAGACATGTCTATACTAATCACAACCTACGAAGGAACACACAACCATCCCCTTCCCGTGGGTGCAACCGCCATGGCAGCCTCAACAGCTTCAACAGCAGCCTCCTTCATGTTTCTGGATTCTTCCAACCCCATTTCCAACAACAACCTCGGAATTCCCCAAAACCAACCGTTTCTCAACTCCCAAAACTACCACCACATGATCCCAACCCTAACCCGAAACCTAAACCCTAATTCCGCCATGGCAACCCCTTACCACCATGATCCCGCAGCTTCTTCCAAACTAGGGCTCGTTCTTGACCTAACCAAAGATGGCAATTTTGATGGCCATCTTCAACCTGGGAACACCGCAGAGAATGATAAACAGAAATTAGTTGAAAATGTGAGCGCCATTGCCGCTGATCCTAAGTTTAGGGTTGCTGTTGCCGCTGCCATTTCATCATTCATTAATAAAGATAAGCCGCCATCCTAG

>IbWRKY70

ATGTCGTCTAGCTCTACCACATCCCAAGCCATGCTCAACCAAATCTTATTTCAAGATGTGGCATCTTCATCTTCATCCCTTTTCTGTGTTTCTTCAAACAATAATAATATTACTGGGACTGGGACTGGGACTGCTCTTCCATTTGAGTCTCTTAAAACCCTCATCACAGTACCCATGCCTACCTCTCTTGCATCACTGCTCCCTCCCCTTGTTGAATCATCATCACCTAATTCTACTTCAGCTTTTCATCAAACACAAACACAAACACTACTACAACAACATCAAGACCTCTCTCCCCTCTTTGGACCACCCCATCATCATCAACTCCTCTCTTTGCACAGATCCGCACCAAACTTATGGGCATGGGGAGAAGTGAATGAGTGCATGATGAGAAGCAAGAGAAGTGGATTAGTGGTGGATGATCATCGTCATCATCATCACCTGGGGGGTCTGGGGGTTTCAGCAGTGAAGATGAAGAAGATGAGCAAGTCAAGAAGGAAGGTGAGAGAGCCAAGGTTCAGCTTCAAGACCATGAGTGATGTTGATGTGTTGGATGATGGCTATAAATGGAGAAAATATGGCCAGAAAGTTGTCAAAAACACCCAACATCCCAGGAGCTATTACCGTTGCACGCAAGATAACTGTCGGGTGAAGAAACGTGTGGAAAGGCTAGCGGAGGATCCGAGAATGGTGATAACAACGTACGAAGGCCGACATGTGCACTCCCCATCCCACGACGATGACGATTCACAAGCTTCATCTCAAGCCAACGATCTCCTCTGGTAA

>IbWRKY71

ATGGAGGATGATTGGGATCTACATGCGGTGGTCAGAGGCTGCGCCGCCGCTGCCTCCTCGTCCGCAGCCACGGCCACCGCCACCACCGCTTCTCCTCTTGGCAGTGCCTTCCAGCCAAGGCAAGACCAAGACTTGCTCTGCTTACAAGATCTGCTTGAATTTGGGGCGAAGAGAAATGAGAGCAGACGGCGCTTTAACGACGACTTGCATGATCTGTACAGGCCTTTCTTTCGGCCGCCGCCACCGCCATCTCAACCTCCGCAGCCGTCTCTGCAACGAACGCAGGCAGCGCCGCCTCTCTCCCCGCAGAACACCCCAATCTCACCCCTCTCTGTTCTCGGAGGATTACCAGATCTGTCTCCCTCCCACCAAATCCTCAAACCACACATTTCTCCCGCTGCGACAATCATTCACCCCAAGAAACAACCTTTCCCCGTCAATATTTCTTCTGCAACTACTCCTACTACTCCTCATACTCAAAGCCCAAGAAGCAAAAGAAGAAAGAACCAGTTGAAGAGGGTGTGCCAAGTTCCTGCTGAGGCTTTATCTTCTGATGTGTGGTCTTGGAGAAAATATGGGCAAAAACCCATCAAAGGCTCCCCATACCCAAGGGGTTACTACAGATGTAGCACCTCCAAGGGGTGTTTGGCCAGAAAACAAGTGGAGCGGAATAGATCTGACCCGTCTATGTTCATCGTCACCTACACAGCAGAGCACAACCACCCTATGCCTACCCACCGGAACTCGCTCGCGGGCAGCACCCGCCAAAAGCCGGCGGCGGCCCACCAACCGGAAACCTCCGGAGATACCATGAAGTCCGGCGCCTCGCCGCCGGCACCGGAAAAGCAAGAAAGCAGCAGGGACGAGAGAGAGGATATGTTTGAGGACGAAGACGACGAATTCGGCGTGGCGAACATGGCAATAGATAGCTTGGGCCCCGACGACGACTTTTTCGAGGGCTTGGAAGACCTGGGCGGTGATTGTTTCCCCGATAGTTTACCCGGCACGACCTTGCAGTTCCCTTGGCTGACGACAACAACGGCGGCCGGCGGTGGTTGA

>IbWRKY72

ATGGCCAACAAATCTGGTGGTGGACTCTCCTTTGATCCAGATCCCATCTCTACAGATAATAACCACCATTTTTTCCGTCAACCTCACTCCTTCTTAACCCTCCCACTTCCCTCGCAAATGGACTCTCCGGTCAACAACTCGCCCCCTACTCTCCAGTTCCCCGTCCACGTCCCCTGCGCCGCCGATCATCATAATGATCGCAAGACAATTCGCGTCTCCGGTGAAGTGGACTTCTTTGCTCACAAGGACGATCGCGCCTCCGCTGCTGTGGCTACCGCCGCCGACGACTTGGATTTTGATGTGAACATTGGTTTGCATCTTCATACCACAAATGCTACCGGTGGCCGGTCAATAGTTGAGGATGCCCTATCACCTAACTCTCAAGACAATAGAGCTAAGAATGAGGTAGGAGTTATTAAAGCTGAATTGGACAGAATGAACGCAGAAAATCAACATTTAAGGGACATGCTACATCAAGCCACCAACAACTATAGTGCTCTCCAAACACATTTGACTTCTCTAATGCAACAACAACAACAACAAAATGGAGGTGGCGGTGGCCTAACTGCGCCACCACCGCCTCTGAGGCCGTTTATGGGTCTCGGGGCTGAGGCGGCCGAGAATGCTTCGCAGTCATCTTCTGAGGGGAAGAGCGGTGGTGAACCGCCACGTCACGGTTCAAAAAGTAGTAGTGGTGATAATGTTGATCAAGCTACTGAGGCTACAATAAAAAAGGCTCGTGTCTCCGTCAGGGCTCGATCCGAGGCTGCAATGATCACAGATGGTTGTCAATGGCGGAAGTATGGGCAGAAAATGGCGAAGGGAAACCCGTGTCCCCGCGGCTACTACCGGTGCACCATGGCAACTGGCTGCCCAGTTCGGAAGCAAACAGCTATGGACAACTTAGATAATGTAGTGGTTACCAATTTTCTGATCACTCACGAGGCAGAAGATAGGACAATTTTGATCACAACCTACGAGGGCAATCATAACCACCCGCTGCCGCCGGCAGCGATGGCGATGGCGTCCACGACCTCCTCCGCCGCGAGAATGCTCCTTTCAGGATCAATGCCAAGCGCCGACGGGGCATCGATGGCGGCGCTAATGAACAACTCCAACTTCCTCGCCAGAGCCTTCCTCCCAGCAGGCATGGCTACAATCTCAGCCTCAGCACCTTTCCCTACCCCTTTGTTCAACCAAACCAAATTCTCCGGCCTTCATCTCTCACACGACTTGGAAAATCAAGCAATGCATCACCAGCAGAACCCGGCCGAGTTGGCCGACACCGTGAACGCCCTGACGTCGGATCCTAACTTTCCGGCGGCTCTTGCGGCGGCCATCGCTTCCATTCTCGGTGGTTCGTCGGTTCCGGACAACACTGCTGCTAGCAACGCCGCCACCCTCGGGAACGACAATGCCGGCAGCGTTACCATGGAAACTACCAAGTAA

>IbWRKY73

ATGGCCAACAAATCTGGTGGTGGACTCTCCTTTGATCCAGATCCCATCTCTACAGATAATAACCACCATTTTTTCCGTCAACCTCACTCCTTCTTAACCCTCCCACTTCCCTCGCAAATGGACTCTCCGGTCAACAACTCGCCCCCTACTCTCCAGTTCCCCGTCCACGTCCCCTGCGCCGCCGATCATCATAATGATCGCAAGACAATTCGCGTCTCCGGTGAAGTGGACTTCTTTGCTCACAAGGACGATCGCGCCTCCGCTGCTGTGGCTACCGCCGCCGACGACTTGGATTTTGATGTGAACATTGGTTTGCATCTTCATACCACAAATGCTACCGGTGGCCGGTCAATAGTTGAGGATGCCCTATCACCTAACTCTCAAGACAATAGAGCTAAGAATGAGGTAGGAGTTATTAAAGCTGAATTGGACAGAATGAACGCAGAAAATCAACATTTAAGGGACATGCTACATCAAGCCACCAACAACTATAGTGCTCTCCAAACACATTTGACTTCTCTAATGCAACAACAACAACAACAAAATGGAGGTGGCGGTGGCCTAACTGCGCCACCACCGCCTCTGAGGCCGTTTATGGGTCTCGGGGCTGAGGCGGCCGAGAATGCTTCGCAGTCATCTTCTGAGGGGAAGAGCGGTGGTGAACCGCCACGTTCGTCTCCGGTGAATAATGTGGAGAGCGTGTCGGGAGAGAATATGCATAGCTTGGAAAAAGGGGGAAGTAGTAATGTAAAGGTTGGTAGGACAGGTCACGGTTCAAAAAGTAGTAGTGGTGATAATGTTGATCAAGCTACTGAGGCTACAATAAAAAAGGCTCGTGTCTCCGTCAGGGCTCGATCCGAGGCTGCAATGATCACAGATGGTTGTCAATGGCGGAAGTATGGGCAGAAAATGGCGAAGGGAAACCCGTGTCCCCGCGGCTACTACCGGTGCACCATGGCAACTGGCTGCCCAGTTCGGAAGCAAGTCCAAAGATGTGCAGAAGATAGGACAATTTTGATCACAACCTACGAAGGCAATCATAACCACCCGCTGCCGCCGGCAGCGATGGCGATGGCGTCCACGACCTCCTCCGCCGCGAGAATGCTCCTTTCAGGATCAATGCCAAGCGCCGACGGGGCATCGATGGCGGCGCTAATGAACAACTCCAACTTCCTCGCCAGAGCCTTCCTCCCAGCAGGCATGGCTACAATCTCAGCCTCAGCTCCTTTCCCTACCGTCACATTAGACCTAACCCAAACCCAAACCCCCAATCCCCTCCACTTTCCAAGAATACCCCCCAACCATTCCCACAATAATAATATCAATAATCAAGCCGCCTTTTCGCCTCTTTTTGGGCAGCCTTTGTTCAACCAAACCAAATTCTCCGGCCTTCATCTCTCACACGATTTGGAAAATCAAGCAATGCATCATCAGCAGAACCCGCCGGCCGAGTTGGCCGACACCGTGAACGCCCTGACCTCGGATCCTAACTTTCCGGCAGCCCTTGCGGCCGCCATCGCTTCCATTCTCGGTGGTTCCTCGGTTCCGGACAACACTGCTGCTAGCAACGCGGCCACCCTCGGGAACGACAATGCCGGCAGCGTTACCATGGAAACTACCAAGTAA

>IbWRKY74

ATGGAAAATGGATGGGGGCTAACTCTTGAAAATTCCATCTTTAAGAACGGTTTGGGCTCCAGTCGTCCGAGATTTGATACTGCTAACATGTTTCCGGTGAAGAAAGACGACGGCGAGAGGCGGCAGCAGGCCGTTCTAAATGAAGTTGATTTCTTCTCTGAGAAGAAGAAGCCGGTTGACAGCGGCTTTGTTGTGAAGAAGGAAACCTCAAATGATGAACCTCCTATAAGGACTGATTTGAATATAAACACTGGTCTGCAACTTGTGACTGCTAACACCGGAAGTGATCAGTCAACTATAGATGACGGTGTTTCATCGGGCATGGAAGAGCGACGAGTAAAAGATGAGGTTGCAGGATTGCAAGCTGAGCTTGAAAGAATGAATGGCGAAAACCAAAGACTGAAAGGGATGCTTACTCAAGTCACCAACAATTACACTGCTTTGGAGATGCATCTCGCGGCACTGATGCAGCAGCAGAAGAAAAATTCAATGGCAGAAAGTGCACATGAGGTTGTGGACAGGAAATCCGAGGAAAAGAAATATGAGAAAGAAGGAAGCACGGTTCCTAGACAATTCTTGGACCTGGGCCCCAGTGGTCTTACTGGCGCTGAGCAAACAGACGAGCCGACTCACTCTCCGACCACATCATCGGAAGAAAAAACAATCTCCGCTTCGCCGAGGAACAACGTGGATTCATCAAAGCACAAACGGTCGGCGAGGGAAGAAAGCCCAGATTTGGAAAGCTGGAATCCTAACAAAGCTCCCAAATCCATAATTTCTTCTTCTTCTTCAAGGCCAGTTGATGATCAGCAAGCTTCTACTGATGCTACCATGAGGAAAGCCCGTGTTTCTGTCCGTGCCCGATCTGAAGCCCCCATGATTAGTGATGGGTGTCAATGGAGGAAATATGGACAGAAAATGGCTAAAGGAAACCCATGTCCACGGGCTTATTATAGATGCACCATGGCTGTTGCTTGCCCAGTTCGCAAACAAGTGCAAAGATGTGCTGAGGATCGGACCATTTTGACCACCACATATGAAGGCACACACAACCATCCTCTCCCGCCGGCAGCCATGGCCATGGCGTCAACCACCTCCGCCGCCGCGAATATGCTGCTTTCCGGCTCCATGCCCAGCGCTGATATGATGATGAATCCAAATTTCCTAGCGAGGGCAATTCTCCCATGTTCTTCTAGCGTGGCCACAATTTCCGCTTCCGCACCATTCCCCACTGTCACGCTGGACCTCACCCAAACCCCCACCTCCCTTCCTAATTACCCTAGACTCCCTCCCTCCCAATTCCCCGGCGCCGGCGCCCCTCATAGCCTTCCAGGTTTCGCCGTAACGCCGCCGCAAGTGTTTGGGCAGGGCTTATATTCTCAGTCCAAGTTTTCTGGGCTTCAAGTTTCTCATGAAGCGCAACACCCACTCCTCCCGCCTGCACACCCTTCTTTATCCGACACCTTGAGCGCCGCCACTGCCGCTATTACCGCCGATCCCAACTTCACCGCCGCTCTCGCCGCCGCCATCTCTTCCATCCTCAACGGTTCACAGCCAAACATTGTTAATAGCACCAATAATAATACCACCGCGACGGCCAATGCACCCAATAGTAACCCTACAAACAATTGCAACAAAACGCACTAG

>IbWRKY75

ATGAACACCTGCAGTAACAACAACAACAACGAAAGCCCTCACGACCATATGGATATCGATTTGTCCTTGAAGCTCAACGAATCTCCTCAAGATTCCCCGCAGCCTGTCGGAGAATCATCATCTCAACCAAACAAAGGAGATTCCCCATCGTCGTCCAAAAATTCCAAGACAGAAGAGTTTATTAGATGGGATGATCCACATCACTACGAGGCCAGGGCTCAATCCGGCCTGCATAACAGTGCTGCAAACGGAAATGAAACGCATGAAAGAGGAGAACAAGGTGTTGAGGGAGGCAGTGGAGCACACCATGAAAGATTTTCTCGATCTGCAGACCAAACTTGCACTATTTGTCCAGCAAAACGATCACAAAAGGACGTTATTAGCAATTTTCTTTGGGTGAATGGGGAAGAAGGGAAGATTAGTCAAGAGCTGAACAGGACATCATCATCGTCATCGCCGACCGCACAGCTGGAACATGATAATAATAATAATAATAATAGTATTAGTGATACTGAATTAGGGTTGTCGCTCACGCTGCAAACCGCCGCCGCCGCAGATGATCATGAGAAAGGAAGAGATCAGAAGAGTCCGCCATCGCCGCCGCCGTCAATACACGGTAATATTCACCATGGAAGCAGCTTTGCTGCAGGGCTTAGTGCCAAGAATCCCCCCATTTCTCAGCACAACGTCAGAAAACCTAGGGTTTCGGTCCGGGCTCGGTGTGAATCTGCCACTATGAATGATGGTTGTCAATGGAGAAAATATGGCCAAAAGATTGCTAAAGGAAATCCTTGTCCAAGAGCCTATTATCGGTGCACGGTAGCTCCGGGATGCCCCGTAAGGAAGCAGGTTCAAAGATGCATAGAAGACATGTCTATATTAATCACAACCTACGAAGGAACACACAACCATCCCCTTCCGTGGGTGCAACCGCCATGGCAGCCTCAACAGCTTCAACAGCAGCCTCCTTCATGTTTCTGGATTCTTCCAACCCCATTTCCAACAACAACCTCGGAATTCCCCAAAACCAACCCTTCTTCCAAACTAGGGCTCGTTCTTGACCTAACCAAAGATGGGGTTTCCTCTTCTGCTAGCTCTTCAACCTCTTTGCCCAAACTGCAGGGACAAATGGGTCAATATTCCTCTTGGATGATGCAAAGACTAGCAGGCAATTTTGATGGCCATCTTCAACCTGGGAACACCGCAGAGAATGATAAACAGAAATTAGTTGAAAATGTGAGCGCCATTGCCGCTGATCCTAAGTTTAGGGTTGCTGTTGCCGCTGCCATTTCATCATTCATTAATAAAGATAAGCCGCCATCCTAG

>IbWRKY76

ATGATGTCCGGCTCAGATTTTCTCCAAGCAAACATTAATCCTCCATCAAAAGATCACCCCACCTTCAATCATGAATCTTTTGATAATCTTCCTAGTATAATTGAAGATTATCATCGTCATTATTATCCAATTTCCGAGGTTTCGAGCTATATTTCGTACGTTAATCACTTCCTCAACGACGAATCTCCGGCGCCGGCGCCGCCTAATAGTACCGGTCACAGCTTTGGGTCGCCGCCGTCTGCCGCCGTAGTTACGCAGGAAAGGAGTTGCACCACGACCACGACCACCGGAAGCTCGTGCAGTTCCTTTGATGGAATGCCGCCGACGAGCAGCCCTCACTCGCAAATGATAGGGTATAGCAGTAATGAGATGAGAAGGAAGTTAAAGGTTAATAAGGAGGAACAGACGATTGCTTTCCGAACAAAGACAGAGCTTCCGGTGTTGGATGATGGATATAAATGGAGGAAGTATGGGAAGAAGATGGTCAAGAGTAACACCAATCCCAGGTAA

>IbWRKY77

ATGGCGGTGGAATTGATGCGTGGTTTCAGAAGCGAGAGTTTGGGCGGGAAGATGGAGGAAAACGCCGTCCAGGAGGCGGCGACCGCCGGCCTTCAGAGCGTCGAGAAGCTTATCAGATTGATTTCTCAGTCTCAGCCGCAAAACAGTGGATTTTCTGCTGCGCCGCCGTTGCCGGCGTTTTCCGCCGATTACCAGGCGGAGGCGGGCGCCGCCGTGACGAAATTTAAGAAGTTTATTTCTTTGTTGGACCGATCCCGGACCGGACACGCCCGGTTCAGGAGAGGCCCGGTTGTGAATCAGAAGAGGGAAGTAGATCCTCCTCCGGTTAATCAGAACTCGAGTAGCCGGATCCGGGTCAGCGAAGAACAACCCGAGAAGAAAATCTATCACCCGAAGCCGATTCAGTGTTTACCGCCGCTCCCGCACCACCACCAACCGGCGAAGAATGCGTCGGCGGCGAATACGATTGACCGGAAAGAGCCGTCGACCACAATCAGTTTCGCCGCAATGGCGGCCCCTTCTCCGGCCGGTTCTTTCATCTCATCTCTCACTGGCGACACAGACAGCGTTCAGCCGTCTCTCTCCTCCGGCTTCCAGATCACCAATCTCTCCCAAGTCTCCTCCGCCGGCAAACCGCCGCTCTCATCCTCGTCCTTCAAACGCAAGTCCAGTTCCATCGACGACGCCGCCGTTAAGTGCCACAGCACCGGCGGTTCCGCCTCCGGCCGCTGCCATTGCCCCAAGAAAAGAAAAACAAGGGTGAAACGAGTGGTTAGAGTTCCGGCAATCAGCCTGAAGATGGCCGATATTCCCCCGGATGATTATTCATGGAGAAAATACGGCCAGAAGCCAATCAAAGGCTCTCCACATCCTAGGGGATATTACAAGTGCAGTAGTATAAGAGGATGCCCAGCGAGAAAACATGTGGAGAGAGCATTGGACGATCCAACGATGTTGATTGTAACCTACGAGGGAGATCACAACCATTCACGCTCCATCACGGAGGCACCATCTGCCCTAATCCTGGAGTCATCTTAG

>IbWRKY78

ATGCAAGTATTTGGCAGCACTGGTGGATTTTCATTTCCCAGAAGCAACATTTATATTGAAAGTAAATCCAACAACTTTGAGTTTGAATTCCCTGTTGGTTCGTACTCTACATCAGAATCGTCATCAATAGGTCCCAAGCAAAATGAACCTCTGAAGCAAGCTCAAGATCAATGCCTTCCTCAGTCATTAGCACCATCATCTTTGCTTGAATCTTCAATAATTCCTACATCGAAAGAACTGAGGATATCTGCACCTGTTGCTGTGCATACTTCATCAGTTAGTACTGCTCCAATTGAGTCTGATGAACTAAATCAGAGAGGTCAGTCTAATCCTGGTATTCAAACATTAAATGGTGATCAGCCGGCTGCTGGAGCTGAAAGATCATCAGAAGATGGATATAACTGGCGAAAGTATGGGCAGAAACTTGTTAAAGGAAGTGAATTTCCACGAAGTTATTACAAATGTACATATCCAAACTGTGAAGTGAAAAAGATATTTGAACGCTCTCCTGATGGACAGATAACAGAGATTGTATACAAGGGTTCACATGATCATCCTAAACCCCAACCCAGTCGTAGATTTACACCAGGTTCTCTTACATCAATTCAAGAAGATAAATTTGAAAAGGAATCATCTTTCAATGTGAAAGAAGACAAGCTCAATTCCAATGATTTCAATGGATATCCCGTCTTATCACCTGGACGAGTAGATGATGATGGCCATGAAGGTGCAGCCTCACAGCTGCTAGTTACTAATGACGACACTGATGATGATGATCCATTTTCCAAAAGAAGGAAGTTAGATGGTTGTGTTGATATTACACCAGTGGTTAAGCCAATCCGTGAACCACGCGTTGTTGTTCAAACTGTGAGTGAGGTTGATATATTGGATGATGGATATCGCTGGCGTAAATACGGGCAAAAAGTTGTCCGGGGAATCCAAATCCCAGGGTGTTCCGTCAGAAAACACGTTGAAAGGGCTTCTCATGATCCCAAAGCTGTAATAACGACCTATGAGGGAAAACACAATCATGATGTACCAACTGCCAGGACTAGTGCCAATCATGAAACAGGAGCAACACCTTTAGGTGGAGCATCAAGGGTCAGGCCAGAAGAGAATGATGCAATCAGCCTTGATCTAGGCGTTGGAATTGGCTATGGTACTGAGCACAGGCCTAATGGCCAACTTCATTCACTGGCCCCAGAAACCCTCCAAGGTCAGGTTCATGTTTCAAGTTCTGGTATGATGACAGTTCAACCACCACCAATGGTGTGCTATGGACCTATACATGGCGGTATCAACCGATTTGGATCTACTAGGCAAAATATGGTTCAGGCCCCTGGCTTTGATACACTGCCTCTACAACCAGCCAACCAATGCCCACAGACCCTTGGCCGAATACTTTTAGGCCCATAA

>IbWRKY79

ATGGGTGGATTTGATGACCATGTGGCCATCATGGGAGATTGGATGCCTCCTAGTCCAAGTCCAAGGGCGTTTTTCTCATCAATCCTGGGTGATGATCTCGGGACAAGGTCAGCTGCTGAGCCTACGAGGGAGAACAAAACCGGGACCCTTGTTTCAGAGCCTGAAGGGTATGCTTCGTCGAGAAATAGTGATGGGAAAGGTGGTGATCAAGCGGGGAGCATGAGTTCACTCTCTGAGCAGAAAATAGGTTCTCGTGGTGGTGGTCTCTTAGAAAGAATGGCAGCTAGGGCTGGATTCAATGCCCCACGGCTAAACACAGAGGGCATTCGAACTGCTGCTGATCTTTCGCAGAACCAAGAAGTTCGGTCTCCTTACTTGACGATTCCTCCTGGTCTCAGTCCAACAACTCTGCTTGATTCTCCTGTTTTCCTCTCGAATTCACTGGCACAGCCGTCTCCAACAACTGGAAAATTTCCATTTCCTTCATCTGGTGATTTTCAGAACTCAACGATGTTTATGGAGGCTTCTCGTAAAAGCAAAGAGAGTTCTTTCGACAACAATGATTCGTCGTCCTTTGCTTTCCAGCCAGTTATTCAAACCGGCCCTTCCACCTTTCCCGGCACATTTGGCAAAGTACCTCCATCCAATCTAGCACGGCAATCCTTTCCCAGTGTAGAGCCTACTCAAGTCTATGCACAGAATGGAACAATTCAACGGCCTGATTTCTCTAGATCTTCTACTGAGAAGGATAACGGGAGTGGTAATATCACATCAGAGCATTCTCCACCTCCCGATGAGCTGCCAGATGAGGAAACTGATCAAAGAGGAAGCGGAGATCCCAACTTTCTCGGGGCCCCTGCTGATGATGGTTATAATTGGAGGAAATATGGGCAGAAACAAGTGAAAGGAAGTGAGTATCCCCGGAGTTACTACAAGTGCACACATCTAAATTGCCCCGTCAAGAAGAAAGTGGAGCGTTCTCAAGAGGGTCATATTACAGAGATAATCTACAAGGGAGCCCACAATCACCCAAAACCTACACCTAATCGCAGATCAGCACTCGGATCCGCAAATCCACTTGGTGACATGCAAATAGAAAATACTGAACAAGCTGGAACCGGAGGGGATGGCGATCCAATTTGGGCTAATATGCAAAAGGGATCAGGTGCTGGAGATCCCGATTGGAGGAATGACAATCTTGAGGTAACAACTTCTGCACCTTTGGGCTCAGAATATTGCAACGGGTCTTCCTTGCAGGCCCAAAATGTCACTCAGTTTGAATCAGGGGATGCAGTGGATCGGTCTTGTACTTTTTCAAACGACGAAGATGAAGATGATCGTGGAACACATGGAAGTGTGTCGCTAGGTTATGATGGTGAAGGAGACGAGTCCGAGTCAAAAAGAAGGAAGATTGAAACTTATGCAGCAGACATGAGTGGTGCTACCAGAGCCATTAGGGAGCCCAGGGTAGTGGTGCAGACTACCAGTGAGGTTGACATACTTGATGACGGGTATCGCTGGCGTAAGTATGGACAAAAAGTTGTCAAAGGGAATCCAAATCCAAGAGTCAATATTGCCTCCACTGAGGCTCAATCTCATGACCCCCCATATAGGAGAGTCACTACATGGCATCTGAGCACAAGAAGTTACTACAAGTGCACGAGTGCTGGCTGCACTGTCAGGAAACACGTGGAGAGGGCTTCTCACGATCTGAAATCGGTGATTACCACCTATGAAGGGAAGCACAACCACGATGTTCCTGCAGCCCGCAATAGTAGTCACGTTAATTCTGGAGTTTCCAACAGCGGGCCCTCCCAAGCAACCGTGCCAAATCAAACCCATTTGCATAGGCCCGAGCCTTCACAGCTTCAGAACACCATGGCAAGGTTTGAAGGGCCTCTTTCACTCGGCTTAACCAATCTGGCGATGGCTGGACTGGGCGCTAACCCGGGCAAATTGCCCGTTCTTCCAGTTCATCCGTATCTTGGACAACAGCGCCCGGTCAATCACATGAGCTTCATGATGTCCAAGGGAGAGCCGAAGATGGAGCCCGTGTCAGACCCCGGGTTGAACATCTCCAATAGCTCATCGGTTTATCAACAGGTCATGAGTAGGTTGCCTCTTGGACCTCATATGTAA

>IbWRKY80

ATGAAGCTTCTCCCGTCTTCCCACGAAACTTACTACGAAGGATCAGATGATTCAGACCAGACAACATATTTATACTTCCACCACTACTCATTCTTCTTCACTGCACTACCAACTCGGATCGATCCCATGGATGGGAATTACCAGAATACGGGCTCGCCTTTTGGCAGCCCCCATCATCAGCCTGTATTTGAGCCCTCTGAGTTTCTCGAGCTGAGCGACTGGGCTGAGGAGGAGCCGGCGGCGATGCACGTCTCCGGCGGTCATTATTATCCGCTTCTTAATCCGCCGCACCACCAGGTTCCTCCGCCTCCGGAGGGAGTTCACGGCGGCTACCTGCAGGGAGGACCTAGAAACAATGGTGGGAGCTACGGCGGCGGCAGAGAAAAGTTCGCGTTCAAAACAAAGTCTGAGGTTGAAATACTGGATGATGGGTACAAATGGAGGAAATACGGCAAGAAGATGGTGAAGAACAGCCCAAATCCGAGGTAA

>IbWRKY81

ATGAAGCTTCTTCCGTCTTCCCACGAAACTTACTACGAAGGATCAGATGATTCAGACCAGACAACATATTTATACTTCCACCACTACTCATTCTTCTTCACTGCACTACCAACTCGGATCGATCCCATGGATGGGAATTACCAGAATACGGGCTCGCCTTTTGGTAGCCCCCATCATCAGCCTGTATTTGAGCCCTCTGAGTTTCTCGAGCTGAGCGACTGGGCTGAGGAGGAGCCGGCGGCGATGCACGTCTCCGGCGGTCATTATTATCCGCTTCTTAATCCGCCGCACCACCAGGTTGCTCCGCCTCCGGAGGGAGTTCACGGCGGCTACCTGCAGGGAGGACCTAGAAACAATGGTGGGAGCTACGGCGGCGGCAGAGAAAAGTTCGCGTTTAAAACGAAGTCTGAGGTTGAAATACTGGATGATGGGTACAAATGGAGGAAATACGGCAAGAAGATGGTGAAGAACAGCCCAAATCCGAGTCCTCCTCTTACCCTTGTAATTCTTTTCCCACTTAATTCAAAGAAATAA

>IbWRKY82

ATGGGCTACTACAGTTCTAGCTTGGGAGATAAATCTCTTGCCATTGACCTCAATACTTCCATTAATATGGATTGTGAAGCAGCTGATACTTGTGGCAATAGCAACTCATCTGTGGGAGATCAGAAGCATGGTTTGTTGATGGAAGAGTTGAAAGTGACGAAAATGGAGAACAAGAAGTTGGCGGCAAAGCTTACAGAGGTGTGTGAAAACTACTGCACCTTGCAGAACCACTTCTTAGGGCTTCTGAAGACCCACGGCGCCGACGACGACTTCCTCGGGAAGAGAAAGTCCGCCGACGACGACGGCGACTGCTATGGCGCGGCGGCAGCGTCTTCCCCGAAGAGGCCCAGGGAAACCAGGACACCTGTCTCTAGGGTTCGTGTGAAAACAGACCCCTCCGATATGAGCTTGGTGGTGAAAGATGGGTACCAATGGCGGAAGTATGGGCAAAAAGTGACAAGAGATAATCCTTCTCCAAGAGCCTACTACAAATGCTCTTTTGCACCTTCTTGCCCAGTCAAAAAGAAGGTACAAAGAAGTGTTGAGGATCCATCAATTCTAATAGCCATATATGAAGGAGAGCACAATCACCCACACCCGACCCAGCCCGAAGTATTATCCGTTCCATTACCACAAGGCTTCACACCACAATCAATCTGCAGCCCGGTTTCAGATGTAGAGAATTCTTCGAGCCCTGCCCGATTGGATATTCGGGCCAAACTTCAGAGATCTCTTACTTCAATTGATTCAGTGGAACTCCAACACTTTTTGGCTGAGAAAATGGCTTCTTCTTTGACCAAAAACCGGAGCTTCACGGATGCGCTAGCGGCGGCGATTTCCGACAGAATTCTCCTCGACCACGCGCTCGCAGATTCATGCTCATGA

>IbWRKY83

ATGGGCTACTACAGTTCTAGCTTGGGAGATAAATCTCTTGCCATTGACCTCAATACTTCCATTAATATGGATTGTGAAGCAGCTGATACTTGTGGCAATAGCAACTCATCTGTGGGAGATCAGAAGCATGGTTTGTTGATGGAAGAGTTGAAAGTGACGAAAATGGAGAACAAGAAGTTGGCGGCAAAGCTTACAGAGGTGTGTGAAAACTACTGCACCTTGCAGAACCACTTCTTAGGGCTTCTGAAGACCCACGGCGCCGACGACGACCTCCTCGGGAAGAGAAAGTCCGCCGACGACGACGGCGACTGCTACGGCGCGGCGGCAGCGTCTTCCCCGAAGAGGCCCAGGGAAACCAGGACACCTGTCTCTAGGGTTCGTGTGAAAACAGACCCCTCCGATATGAGCTTGGTGGTGAAAGATGGGTACCAATGGCGGAAGTATGGGCAAAAAGTGACAAGAGATAATCCTTCTCCAAGAGCCTACTACAAATGCTCTTTTGCACCTTCTTGCCCAGTCAAAAAGAAGCACAATCACCCACACCCGACCCAGCCCGAAGTATTATCCGTTCCATTACCACAAGGCTTCACACCACAATCAATCTGCAGCCCGGTTTCAGATGTAGACAATTCTTCGAGCCCTGCCCGATTGGATATTCGGGCCAAACTTCAGAGATCTCTTACTTCAATTGATTCAGTGGAACTCCAACACTTTTTGGCTGAGAAAATGGCTTCTTCTTTGACCAAAAACCGGAGCTTCACGGATGCGCTATCGGCGGCGATTTCCGACAGAATTCTCCTCGACCATGCGCTCGCAGATTCATGCTCATGA

>IbWRKY84

ATGAAGAATCAAACGGCGCTGTTCCTCGGTTTTACGCCGCCGTTAGCAGCTTCATCGGAGCACGCCAAGAAGGGAAGCGGCGGCACGAAGGTTGTCGGTTACCCGACAGCAGAAGAAGCGAATGATGTAACCAACATTAATAATAGTGATGAAGAAATATCATTATTATCCCAGAAGGAAAGTAGTGGAGGAGTGGTGAGTAAGAAGAAGGGGGAGAAAAAGACACGCATGCCCAGATTTGCGTTTCAAACTAGAAGCCAAGTTGATATTCTAGACGATGGATATCGGTGGAGAAAGTATGGTCAAAAAGCTGTCAAGAACAACAAATTCCCAAGGTTAGTTTCAACCTCTACCTTACCTTAG

Amino acid sequences

>IbWRKY1

MAGFGDHMPIMGDWVPPSPSPRAFFSSVLGNDIESRSNASQPLLPDPKEYASSGNSDSKNVAQGSDQTPKLSSLSERNMNSHGGLLERMAARAGFSAPKLKTDSIRPPALVQNQELRSPYFTIPPGLSPTTLLDSPVFLSNSLVQSSPTTGKFAFPSIGDSRNSALFMGASDNNKETSFNNNDASSFAFKPVIETGPSLFPETISKVPPSNLSWQSVPGIEVSVHSENPRVHQHAEPTLVHTQSGTLEQSVFSRSYTEGVSNIISEPRTFQAVAGSMEHSPPPDEQQDEEIDQRGGGDPNAVGAPADDGYNWRKYGQKQVKGSEYPRSYYKCTHPNCQVKKKVERSHEGHITEIIYKGTHSHQKPPPNRRAAFGSENAEQDGTIGIGDPIWENVQNGSGAGGPDWGNDNLEVTSSGTQFEPRDPVEGSSPFSNEEDEDDRGTHGSVSLPEGEGDESDSKRRKIETYAADMSGATRAIREPRVVVQTTSEVDILDDGSYYKCTSAGCTVRKHVERASHDLKSVITTYEGKHNHDVPAARNSSHPNSGASNCLSSQTTATQGHVHRPEPSQLQNTMSQFARPPSLGSFGFPGGPQLGHTLGFGFGMNQQGLANLAMAGLGPNQGKFPVPPVHSYLGQQHPMNDMRPKAEPKMEPSSDPGLNLSNDSSVYQQFTSRLPLGPQM

>IbWRKY2

MDAHHLLHHPTVILNSLDDPTLTAPAPAFRPEKRAVNELDFFKRENSDLDSAMDEALVSKGNGRRVGDEAVNHPPVLDTGLDLLGSSKKSMVFHGASPPATMEHKATVEEERSLYLTALREELERMNSENQRLKSMLNQVHEKYNALKMHYAYILEHQHTLKPEIPEDNKMNDGFAEGNERKRKVTDDMKEEHSHSSPEGAGASPPCPEDNTREESPDKVQQKLVRSNGEHSDHLLPAAADQHAPAAKKARVSVRIPCDTPYCSDGCQWRKYGQKMSKGNPCPRAYYRCTMTSTCPVRKQIQRCAEDRSVMIVTYEGEHNHPLPPAARPMASTTSAAATMLLSGAARSADGAGRPANLDALPANFLPTISTFAPVPTITLDLTNPMATQPQTPPPFHSPNPPLPPGIVSAAAAALTRNPSFTAALVSAIASIIGGNNIAAQPPPPPNSAELSDQDVKPSPSIESNVQAKVL

>IbWRKY3

MDGEKNDWDLGAVVRGCKNLNGNSSSQDVNEHFNGGFASQTYVSIPLLPQPSHYYSSVSPSIGTERRYFGLEEVIDRFTNGKVRELALDLQATINPTCLDKADGSGGMGKDTPALQSPDPYFGLEEVIDRFTGGKMREPILDLKATITRPTSLDKAGGSGLGGIGKDTRAPQSPNPSPTLEPQQPLPSSQTSSPEKDGGLEDENLPHQVEEVNEVEMSVVKVKVPVEKVQVPAEKVDEWDGWEWRKYGTKMMNDSPHSKGYYRCNHEEKKCPAKKHVQLSYMDESTYIITYKGNHNHPPPVQTTTSKRKKRSWARARPPAPPASKGEGSFSTPSTNTAT

>IbWRKY4

MDNIGGDREFLQSLISELANGRDAATHLQMILNAPSSFSPETRELLVHNVLASYDRALGMLNYSPESAAVQPPAAGPALGIESPSSFTGSPHSEDSDREYDGSRRRNAPRWTQKVQVCPGSGLEGHLDDGYSWRKYGQKDILGARYPRGYYRCTHRHAQGCLATKQVQRSDEDPTIFDITYRGRHTCNQAGGNPNPPQNQEPNMPESQRNIIPFTQQQGQPSETLLSFRRQLRVETSDLDNTHQDNHQQFTYLYTFPSSSDQSYTFRPPSAPDASNFSWSISPSFGSPASTTASSDFGRRPRPTTEAPPTSTTNSGGIIPAVSAGNSPAVDPDSLLALWVLTQTSPLIAMDSFQTPEN

>IbWRKY5

MAVDLMMDYRHGGNGRSVDLTFAKKLEESAVVQEAASGLESVHTFIRLLSQQKHKAAESRGKSTVEIEMVADVAVNKFQKVINLLGRTRTGHARFRRAPVVSSLPVPAKVDTKVYNPTPIQQVPPPVSAAAAGKTISFSYSPEVSRANSFNISSLTGETESKQASSSSAFQITNLSLASSGGKPPLSSSSLKRKCSSSENNLSGKCSGGSSGRCHCSKRKKLRQKTVTRVPAISMKMADIPPDDFSWRKYGQKPIKGSPHPRGYYKCSSVRGCPARKHVERAVDDPAMLIVTYEGEHNHSLSVAETNSLILESS

>IbWRKY6

MSEITERGSIVAGPSRPTALAIAPRPPLESFFNDGFIPGFSPGPMTLVSGFFADSDGCSFSQLLAGAMASPLAKPSVLEDSSAKKGSSGAGSEKQSGYKQNRPVSLAVAALSPLLVVPPGLSPSGLLNSPGFLSPIQWPVVLLGLSRSWVKAIYLLVLMLFCWGGVYRYTLSDSAPLLGWGLLVTSSYGDYGFPSSLKKNAGCKHVFFICYSPFGMSHQQALAHVTAQAAINQSYRQMQTEYQQQSSPADAFEHESSLMPNDSFQFQVDDMLDVESLKSEPVEVSQSLRKPAPGVLERPARDGYNWRKYGQKLVKGSDCPRSYYRCTHLKCPVKKKVERSVGGHITEITYKGQHNHELPNPNKRRKDECDLDGGENIQVNSEIASHSWTEMNTSNEAEFSESAQLPTKLPSEQLDVGCDLDEMEETAMALDEDDGQNPKKRSLEVVSSVMPSSHKTVTEPRIIVQTRSDVDLLDDGYKWRKYGQKVVKGNANPRSYYRCTYSGCSVRKHVERASTDPKAVITTYEGKHNHDIPNGRNSNRSQTNANVLQLKQQSTLAVNS

>IbWRKY7

MSDMNPNFLGPNMMSNPPSYTNPNSNNLHFYPEMLQDYDYDYAQDFDLSYVNTLLNDDHHYSNVVSNNPFASSSTSSSSSLFSHPPPPPTIVQEKSSTTSASSGSSGFDGMMPTTYPMQEVNSMRQMVNTTKAKERHSIAFRTKTELEILDDGYKWRKYGKKKVKSNSNPRFDVFSKHNLINISKESSLQLKLISMTSSYPSAFL

>IbWRKY8

MFNNMNPNFLGPNMMSTPPSYTNPNSNNLHFYPEMLQDYDYDYAKISTFPTLTTSSMMMMIIIYIITPMLLIALCSSSSSSSSSSSLFSQPLLPPPTIVQDNSSTTSASSGFDGMLPTTTYPMQEVKSMRQMVVNRTKTKERHSIAFRTKTELEMLDDGYKWRKYGKKKVKSNSNPRNNPVMTQKQDPKCPKPESRGGEEPGNQGQG

>IbWRKY9

MSDMNPNFLGPNMMSNPPSYTNPNSNNLHFYPEMLQDYDYDYDYAQDFDLSYVNTLLNDDHHHHYSNVVNNPFASSSTSSSSSLFSHPPPPPTIVQDKSSTTSASSGSSGFDGMLPTTTYPMQEVNSMRQMVNTTKAKERHSIAFRTKTELEMLDDGYKWRKYGKKKVKSNSNPRKQFAVEADFHDKCYPLLL

>IbWRKY10

MLQDYDYDYAQDFDLSYVNTLLNDDDHHHNSNVVNNPFASSSSSSSSSSLFSQPLLPPPTIVQDKSSTTSASSGFDGMLPTNTYPMQEVKSMRQMVVNRTKTKKRHSIAFRTKTELEILDDGYKWRKYGKKKVKSNSNPRNYYKCSHEGCIVKKRVERDGEDSKFLITEYEGIHNHESPYVIYYY

>IbWRKY11

MAASSGTVYSTAFTELLAGDVYPAGVKSLSPPSLPLSPSTQLLDSPLLLSASNLLPSPTTGTFPAQPFSWNSTANVTREALKQENNGGFSDFSFLTNPASAGELNWAYQEGNEVEDAASSEKAIGELNTVVNSQSRNCSDYNHHKNQALKRSDDGYNWRKYGQKQVKRSENPTSYYKCTHPNCPTKKKVETTLEGEITEIVYKGSHNHPMPQTTRRSAASPPSSYVSNGTGQLDNSVATPENSSVSYGDDDFEQSSRKRELGEGEFHEYDSNAKRWKAETGNENEGISALGSRTVREPRVVVQTRSDIDILDDGYRWRKYGQKVVKGNPNPRSYYKCTTLGCPVRKHVERAPQDTRSVITTYEGKHNHDVPAARGGHSLNRPIPSNSNNSPAIAIRPSAMSHQSSLLAAILPTQGSGNFANQGTLFSGSREDRLRDDMLLQMLLP

>IbWRKY12

MDNTKSPASELKNSRTLRQASSSKKRSMAQKVVVRVKLGEGDGRKRKSEGPPSDSWSWRKYGQKPIKGSPHPRGYYRCSTSKGCSAKKQVERSKTDASVLIITYTSTHNHPGPPEDNPTTPVPEEEGEEAQMEQHRTRLKDNGYEEDIFHYSQSPFNTSEHIIINRPDDNYLLGTSSSLLLDGEPLSCRYPHFMDFSTPKSQENYDFYDELEELPHTSSFITYTFFKDTISVNSI

>IbWRKY13

MDHPPGGCSQTAVIAAIRPKTVRLRSAGNQTLGGKVDMPGVSVWSPANVLKSDDKPTIVYKPMAKLLSKTTFPQNLNMRSSTSSQQNEAAEETNQVKPSDVRLEAHQSSSLKSGTEKKPVENSKMALQNTEDDERSLFQASGVDCLSSDGYNWRKYGQKQVKGSEYPRSYYKCTHPKCPVKKKVERSSLDDQIAEIGVLVSEDTCNETNNPVRSEQLTLQNEPCGPSTEHKNNTMLSTRSTYSSGAPPPCYPVTSAAAFHGAVSTPENSCTPSGIHREGLEAEGDELKGKRRQCGSQTNNGATLGNGAMETQTVVGSTTDSETTGDGFRWRKYGQKVVKGNTHPRSYYRCTSPKCNVRKYVERAPDDPKSFITTYEGKHNHDIPTRTPNPEASRSSTRAAATKEKS

>IbWRKY14

MEGDEGRSGLPNYGLQVSFSTTPHHHHHHPHAAMHHEMGFVHFEDHNQAVMSFLTPLSSSSQPLDGGAGSSCSNAASTTAAAAKSSSHAATASLGFSHSEPQLSNRPSWNNNDQVGTMDPKGANDENCSGNAAEGNNSWWRSSSSSSLVDKGKVKVRRKLREPRFCFQTRSDVDVLDDGYKWRKYGQKVVKNSLHPRLIITDQLKRRRSSIAILA

>IbWRKY15

MEPDWDLHAVVRGCAVTSTAAAATTTSPFCSFNPRQQDENLDFSHEPFDFSSSTVNNTTWFNEELHDLYLPFLHRREPPSLPPQSPPPPFPVLRGLEGSVLHNQLNITATRIQANGQSLSSNASSTTSSNSRPQTPAGKRRKNQMKRLCQVPAEDLASDMWSWRKYGQKPIKGSPYPRGYYRCSTSKGCLARKQVERNRSDPNMFIVTYTAEHNHPMPTHRNSLAGSTRRKGASQQTTTSGSETNRPSTSLSPPEKQESSRDEKGGVFDDEFGVSNMVNDGNAPEEDDDFFEGMAELGESLKSDDIGDSFSDNFQDAMQFQCWLPTTAGGGG

>IbWRKY16

MEKVGVLEGNSLIINELTQGRELAAQLKGQFDSFTSPEICEPLVEKILSSYEKALTLLNFKLFFGGDPNAMDSPLPLLANNSNVCPTGEASDGDSSKEQCHVFKKRKTSPQWSKQVRVCSASGLGANLDDGHSWRKYGQKDILGAHHPRAYYRCTHRNTQGCLATKQVQRSDGDASVFEVTYKGRHSCKASHPALISGENERPKPQFQVQQPEGKQLQAQSLVLDYGLNQKLETTEEDHVLPPFSFPPTIKCEVADKENNLLKCCSPSPFTAPSTSEECMYLSFLPGQNDDDDFGLSQILDRSESDLTDHMISTPTSVTNSPFQDWDFLADQPNLDATDISKYFS

>IbWRKY17

MEEIEEANRAAVESCHRVISLLSQPHDQSQVRKVKKIQTPSLPPSILLENPMCRGDDHHPKALQLLPAISLEASNQEKGSSGVIKSGLALGSPSFELNLHGKTPVPLSHQTPIPSYHFLQQQQQRYQQQQQQQLKQQAEMIYRRSNSGISLNFDSSTCTPTMSSTRSFISSLSIDGSVANMDANSFHLIGASRSADLSSYQHKKRCSGRGEDGSTKCGSSSRCHCSKKRKHRVKRSIKVPAISNKLADIPQDEYSWRKYGQKPIKGSPHPRGYYKCSSMRGCPARKHVERCLEDPSMLIVTYEGDHNHPRVPSQSANT

>IbWRKY18

MAASSGTIDAPTASSSFSFSTASSFMSSSFTDLLASDAYSGGSVSRGLGDRIAERTGSGVPKFKSLPPPSLPLSSPAVSPSSYFAFPPGLSPSELLDSPVLLSSSNILPSPTTGTFPAQTFNWKNDSNASQEDVKQEEKGYPDFSFQTNSASMIQRGKMSSILCRAFPPVTTSTQMSSQNNGGSYSEYNNQCCPPSQTLREQRRSDDGYNWRKYGQKQVKGSENPRSYYKCTHPNCPTKKKVERALDGQITEIVYKGAHNHPKPQSTRRSSSSTASASTLAAQSYNAPASEVPDQSYWSNGNGQMDSVATPENSSISVGDDEFEQSSQKRESGGDEFDEDEPDAKRWKVENESEGVSAQGSRTVREPRVVVQTTSDIDILDDGYRWRKYGQKVVKGNPNPRSYYKCTSQGCPVRKHVERASHDIRSVITTYEGKHNHDGLRRCLQSNYPIPIPSTRPMQQGEGQAPYEMLQGPGGFGYSGFGNPMNAYANQIQDNAFSRAKEEPRDDLFVETLLA

>IbWRKY19

MAKNDGGSSSSSAASRSAPERPTITLPPRSSVESLFTGGFMSGISPGPMTLVSNFFSEGDSYSECPSFSQLLAGAMASPAAFGGVRPPPPPQPVEAKEESGGGGSGGDSDFRLKHNRPAGLAITQQSMFTIPPGLSPTTLLDSPGFSALFSPGQPFTLYCIGGLVDWWTGGVLIRKSASVAVTAHALLCTKLKNPRLFCAKRTVSFLSCFLSLQGAFGMSHQQALAQVTTQAAQAQVQMHIQPDYSSSSAAPATSYSQLQTIASNATINQQVPSQASDHNIMKESSEVSHSDQRIEPASFPVDKPADDGYNWRKYGQKHVKGSEFPRSYYKCTHPKCPVKKKVERSLEGQITEIIYKGQHNHPPPQNRKGAKDPGNSNGPYALQGGSELSSEGLTTNFNKPKDQESSQATHEHASGSSESEEVGDAETRADGGDDDERESKRRAIEMQVPDPATSHRTVTEPRIIVQTTSEVDLLDDGYRWRKYGQKVVKGNPYPRSYYKCTSPGCNVRKHIERASNDPKAVITTYEGKHNHDVPAARNSSHNTSNNNTGPQLRPHNVAGQQQAALRTDYSSNEQQVALLRFKEEQIT

>IbWRKY20

MENKAAELSKPENNNNNNNPMVTPSFSDQIPATFSLQTLFDIPSSDHSKNPSSSFFDYLFPSQDLSTAVFDLLQTPQPPSQPLPESSEAVNTPVTPNSSSISSSSNEAAIDDQLPKTPAEEVEQESDRSKKQLKPKRKNQKREREPRVAFMTKSEVDHLDDGYRWRKYGQKAVKNSPFPRSYYRCTTPACGVKKRVERSSEDPSTVVTTYEGTHSHPCPVTPRATTVGIMPEPSNFGSVSAAPGGTGSPPPSSFLIPHHHFHYPMQQQQPFFAITSPLPPPLTYTPSTWQPREEVPSAVVVFSGRPGRWASAGYAAIPLEKGAPPRRIGCLV

>IbWRKY21

MFRCSSPPSQPYLSLMNMMNNNNGGMESGFLGMKMSAGDVEVPCSEEMKSESICTAAATTENNSGFTEIGAAKSPSSSSAGKKKGEKKMKKARFAFQTRSQVDILDDGYRWRKYGQKAVKNNRFPRSYYRCTHQGCNVKKQVQRLSKDEGVVVTTYEGVHSHPIEKSTDNFENILSQMQIYAAF

>IbWRKY22

MDGENDWDLAAVVRGCNNLNGNSSNQDVHEHFNGGFPSQTYNSIPLLPQQSRDHGSDFPSIVTERYYFGLEEVIDKFTSRKIREPVVDPQSTIIPPTSLDKVDGSGSGGIEKDTPVPQSPDPSPTLEPQQPLPSPQTDSLEKGGGLEDEKLQVNEVEMLVEKVQVAVEKVEVPVEKVDEWDGWVWRKYGKKMVNDSPHSKSYYKCNHEGEKCPAKKHVQLSHMDESKYIITYRGNHNHPPRSKHYNQT

>IbWRKY23

MDDTGEASKPSLQLQNTCADIGGGGGGGDEPSGATTGTETSEEAQVGGSDSEETLDTVDSPSIQLDKSASRPDSLATSSSHVLSEVPIEYSLHPSEFLKEIKDEVGISNQKASTVQAQRRNQLQSADDPSVLELSPTSVTQSISSIPSPTPGERRLSPLENRNGACIQEVDNQNSSNSKALSLVPVLKIQAPDGYNWRKYGQKQVKSPQGSRSYYRCTYSDCCAKKIECSDHTNRVTEIVYRSPHNHEPPRKVNTPKVNKLAISSMPRSQDSKVARLNSNADETVPSTSKKHVKETIPISETKQQDFSGLDDNAETNVKREDCDEPTQKKRLKKCSSSPESLPKPGKKAKLVVHAGGDVGISSDGYRWRKYGQKMVKGNPHPRAVDNTTAVIITYKGVHDHGMPVPKKRYGQPSAPLVAATASASMTDSQTKKSEPTTQWSVDKEGALTGETLEHEGEKTVESAKTLLSIGFEIKPC

>IbWRKY24

MAAHTEFMYPESMMDAEELIQELLDDESPLFLAPQETIMESSGFAGVSNYSLLNSLIYGHANQPLHDSRSCMLERGLMVSRDHHESKYTLRIKTNCGNAMADDGYKWRKYGQKSIKNSPNPRSYYKCTNPKCGAKKQVERCSDDPDTLIITYEGLHLHFAYPFFTLDNEPNKTIDLVPTKKQKKTIAEEVVESQEQEQTNHVVYENNPGREDTNPTPIDEWDSQGLLEDVVPLVIRRPSTICNATTSYSSSSSFLSPPTSPSSLSCSTNNCFLSDFDA

>IbWRKY25

MAEDWKRAIGELIRGQKLTNQLRDSLKDPKVADDLLRQILGTFNKTLWILNKSSIDTDEVSQGAGDPSSPCYDGRRSEDSSGSCKAFVKDRRGCYKRRKTCETQIKESPNLVDDGHAWRKYGQKVILNSKYPRNYFRCTHKFDQNCQATKQVQQIEEDPPLYRTTYLGKHTCRNFQKCPQILLQPEDASVLLCFGQNSQSDMYTCLPTFSSSIKHESKEYNPHSSRIGSSTSDCFVPSDAAGHVAPLSSASDYGDVISSGGTMDMTQFIDSDVVDMDDFLIY

>IbWRKY26

MNKGSKDQEYRDMSKKRKLMPTWTEQVKVGTDNGLEGPPEDGYSWRKYGQKDILGAKYPRSYYRCTYRAMQNCWATKQVQRSDEDPTTFEITYKGAHTCSQAPKSVPPLASPKKQDLKQSIHCKDSLSMQPNQMLMELRSNLRVNTSDLERKETTYPFSFPPTFSGLTDEKPMFQISQVDDNLLGTYSPSFVSPTTPESNYFSVSHQQTSSFGGVQNLHHSESDLTDIFSANTSSTNSPIVGLDYTLDPADFDPNFLFDTSEFFT

>IbWRKY27

MFSTSMVLSGPPPWFPMMPCWRSFTSRFTSLQPSISFAGEVKQAEGAVHQILQQDGDVKKGGLDKSSPVLETPNYVPKFKRRKNEHKRVVVQVPAEELCEDKWAWRKYGQKPIKGSPYPSRAKQHGGGDVHSNIHGGAQPQPADAAELLAGTIRNKFPPSTASKAVKAAEDSSTAHHVPELVAAASALSSPTDNNWRACSEEETKIKDEDDDEEELEMGEKISLSDQIQSGMPQVTMDNNEDFFADHAAVIPPNELLNHSR

>IbWRKY28

MDNDNTITRLILHGINLAKELEADLPNLASQPPEAVSASCEEIIRVFTSVRERLAPPQPPPSSLQPYSLAMIQEAPPQQPRQPAGAGQALDLFHQVSGGEQAAAGGSGADVAEPSRRRRMEEADRTTVYVLAPQMGNLDMPPEDGYTWRKYGQKDILGSRFPRAYYRCTHQKLYNCPAKKQVQRLDNNPQMLKVTYRYHHRCHMSATSPSAAPPPPPTAGDVIQPPGTTTHPPPQPAAGGGGGGSYWLSMDIRPITSEGGVQMQTEFASTSGGAGPSGSGRYGREAADFGGQAVVDMADAMFNSGSSGGNNSMDFIFHTMDEN

>IbWRKY29

MDVPENLLVRQRRAITVLVNGKATTVELQTLLQNPPPDGASSSLPAELVQQIVRSFNQAIFELTSGDAAAQICQIPSASACSGGLTFEDSGETTGKNKKKGRRGRYKKSKNSETWNKVSETQEDGGAWRKYGQKNILHSEHPRCYFRCTHKRDQGCRATKQVQRTSEGLYQTTYFGYHTCKDPQRFPRRKPAADHVFSGDDAPNDHQTVLKAEKQMNLQEEEDEDGGAIDTVKKEEETAQSEISVSKSPDNNNNNDDNNDKFYDDDNFIWGDIIGESSNYESSFYACSSTSFNDLDMGGVADFGTFFPPH

>IbWRKY30

MSSSGGSLNTCVDNSHHHNSYSSFSSFSFTDLLSNNEESKNPEKGLGSSSSSFNWGISDTHEIPKFKSFPPATLPISPSPVSPSSFLNIPVVEPFCAVGLSCLLLHLKSLFLVFRLWFVFEGFEVMLMLFCAQVLPLSPTTGAFAGLNNNPKEEERKSNDFSFQSRAASSSSMFQSSLGRNSMEEQMSRQQQQPNMGSADFSTMKTDIKPELPQTHSFSQENPAMQQQPAMVHYSQPSQYARAQKAEDGYNWRKYGQKQVKGSENPRSYYKCTFPNCPTKKKVERNLDGHITEIVYKGNHNHPKPQSTRRSSSSSQSVQINPESFNIDVANQSNMMLGSTQRDSFITPENSSASFGDEDLEQGSPSRDDDENEPEAKRWKGDNENEAISSASRTVREPRIVVQTTSDIDILDDGYRWRKYGQKVVKGNPNPRSYYKCTFLGCPVRKHVERASHDLRAVITTYEGKHNHDVPAARGSGGYSLNKPQQPQPAGNMGSSAAPVALRPSTMPNHSLNYQNAIFNPRPQTTQSQQPITLQMLQRPAGNLGYSSLGNSTGSYMPTGKDEPKDDFFSTFLN

>IbWRKY31

MAHSSPENSSANRKRAVDGLILGRNLTCQLREVLKNSSDEHGPPSKVVAEDLVAKILESFNEGISVIGSMDSDEVSQPPSDGRKSEDSSGSCKTTSALKDGRGCYKRRKTCETLIKDSQTLVDDGYAWRKYGQKVILNTPYPRNYYRCTHKFDQKCQATKQVQMIRENPALYRTTYNGNHTCLNFQKYPQIIVDSTAHGDSSFLLCFGQNGQTNKEVQNPTLIKQENNKQEFAENLYHSHIQSSSSGCCLPSSDDRPMSSARWGPAASSGSEYGDVNSSGCTHDDLGMQMMGNVDVDDFTLGFLADF

>IbWRKY32

MPKPTMETPNPFHTHRKRVISVLLKGKKSATQLQTLLRNFSHGSQEKSHLVLEILGSFSEAVSQLKNGLPPPDSELSGGRNSGDNPAKARRGCNNRRKCSDTWINVSNTKEDGGAWRKYGQKQILNSKYPRCYFRCTHKHSQGCKATKQVERISEDEYKTMYFGQHTCQDSFRAPVLVIKSISTVDSTQSPSFECITQDGNGGGDHYDDVVVNQENHREFKDRIV

>IbWRKY33

MENEFGVDGEFNLNELISELLQGRDAANRLQLCLNNAPLLPSSSSSSQDWSCEVLANKVQASLDNALCMLNHAGRNESSDSDHEVRDPTSGRRRKAAVWTDQVQVSPGEGLEEAHDDGYNWRKYGQKKILGAQFPKGYYRCSHLHSQKCLAKKEIQKSDEDPTVFNIQQEENLLNLQRNLKIKTDNIEPSSHSHGNPFPSSHDFAAASSSGVIKVGEIHCGFPQPLTNSFVANVSTTTTSDLEMIMEIPTSTSGTNTPIVGMGFPFDATMGFDFNFTFDNNNDNNPAGFFD

>IbWRKY34

MSSSSTTSQAMLNQILFQDVASSSSSLFCVSSNNNNITGTGTGTALPFESLKTLITVPMPTSLASLLPPLVESSSPNSTSAFHQTQTQTLLQQHQDLSPLFGPPHHHQLLSLHRSAPNLWAWGEVNECMMRSKRSGLVVDDHRHHHHLGGLGVSAVKMKKMSKSRRKVREPRFSFKTMSDVDVLDDGYKWRKYGQKVVKNTQHPRSYYRCTQDNCRVKKRVERLAEDPRMVITTYEGRHVHSPSHDDDDSQASSQANDLLW

>IbWRKY35

MAVELMMSYRNVDVNGGGGGRIGFVKSLEESSAVVKEAASGFESVEEFIRLLSQGKKKQQGDREKAAMEIDVVADVAVNKFKKVIDLLGRTRTGHARFRRGPVASSPVMEVPADNKVYSPTPIQQVPPPASYDYSAAAHRYSAAAAPMTISFTCSPEISRANSFNISSLTGETDSKPMLSSSSAFQLTNLSQVSSAGKPPLSTSSLKRKCSSSENNLSGKCSGSSSRKLRLKRVTRVPAISMKLSDIPPDDYSWRKYGQKPIKGSPHPRGYYKCSSVRGCPARKHVERAVDDPTMLIVTYEGEHNHSVSVAETNGLILESS

>IbWRKY36

MEAKLLRITSTAIPTHRKIARIPRKLLAHRSYRQDCTFQFLIPSCSAMHAIPSAQPLPNWRQEHMIEPGLPGSRSVELHSMCWAPFGHQTVVLPPNGLLRDRDLSSTILSGRGVEGLVARKFLFLMGMKSRVDETGNYTLEGVRISLVRLEDSIISGLVERAQYRYNPDTYDPNAFVMEGFHGSLVGRYSDPDEHPFFPLELPEPLLPPLQHPQVLHPNGALVNINAKIWDMYFKKLLPRLVEEGDDGNCGSTAVCDSFCLQVLSKRIHYGKFVAEAKFQASPDLYKAAIRAKDRNRLMQLLTCPEVEELVKKRVEMKVREHFQEVTIDMEGESKSDPKYKINPIFVANLYGDWVMPLTKEVENFPQGESSEWGKQQRRSFCGFGSVAPDDYNPAPTPFDTTLFSLGSIPGFSPGPMTLMSTFFSDSDACSFSQLLAGAMASPLANPALLPDKDGDSGPGCEKLSGYKQKQPMSLLVAQSPLFMVSPRFSPSGLLNSPGFLSPLQALAHVTAQAAFNQSYKQMQVEYQHSSSVEGAGHQTSSSMPNQAVQGEAANVALDTESLKVETSELSQVDSKVSSGAIEKPASDGYNWRKYGQKLVKGSECPRSYYRWKHNHEVPKSNKRKQDDCDQESREDNSREKPQSASHRWTETNRSSSQPEMVSTKLQSEQLTVASKRDEMEETATVLDEKDDGERNAERRISEAGSSVLPSSHKTVMEPKIIVQTRSEVDLLDDGYKWRKYGQKVVKGNANPRSYYRCTYKGCNVRKHVERASTDPKAVITAYEGKHNHDTPNAPNNNHTETKKQQHSAVETTKVGSCVAWNRYLKEQIEEENPIQYQ

>IbWRKY37

MADSLDISGDGAAKQKAGDSLAGRQEGFMTAVLKKDEGRAAATVKAEMKEVKEENARLKTLLAKIEKDYSSLQMRFFDVFSNQPAEIEKKSCKISSPMSSHHHDEETQISLRLGRSPSPDRRQSRVIDDINAAAAAKSTDEDDDEHNQTLKLGLDYGGDNKSTEPNLELSSGRQSPDNSASETKEEDAAAAGETWPPSKALKATRSGDDELSQPSVKRARVSVMARCDTPTMNDGCQWRKYGQKVAKGNPCPRAYYRCTVAPSCPVRKQVQRCADDMSVLITTYEGTHNHPLPVAATAMASTTSAAASMLLSGSTTSQTAGLRSPPSPATNFFPGLNFSLPADTSRTTRPLYFPNSSSPPFPTITLDLTTSSNNISSMFSSNVMKSAPRFPSTNLSFSSSESNISPAIWSTGGYTNYSTIYNRNNNILGTSQPGKSSQEQPFYGQAAAASQQALTETLTKAITSDPSLRSVIAAAITSMVGNNAPMQHKRVKVNDEVIGTHQAIS

>IbWRKY38

MSENQFYHDHTGFNPLFIGDDGDDHRNPSLYSSNLPHPEFDPSSPFVNYNVVSSSGFPTLLWSSSTRSEVVGPPSSDHHVQECSRKSSGSVGQPPASSSSSEAAGGGGGGEEDSSKSGKNLEGVECEDGEDKSKKMYKGKKKEEKKQREPRFAFITKSGIDNLEDGYRWRKYGQKAVKNSPFPRSYYKCTTQKCPVKKRVERSHQDPTTVITTYEGQHNHHCPATLRGNAAAMFSSPPSFFPSSAPQPPPRDLFANQMYPAVVPHSPSPIMYDYQQSHGGLIGGHAPPQPQFDYGMFQEMVASLVQNRNIIIFEGFKDYWCFFCCVFKCWWIGGCVSVDEHGLMITLRALT

>IbWRKY39

MEEGVDEGSLGKLQPKREPNTGSLESETEHKVSDKLVPAEGVSGELQKRLSPNVKEEASESSESKVAVPDKCETVPSDMQRKQGVVNGSSASQSEKEKSSHSGVQVKDGEEVQQKQGVGSDTDASQFSQVSIVPKKESDGTGHEQSNDEKIHGTETLALAVIPEKNSDNPQQLQIQSMEVLATHSNQARVTYVKPHEKGLDKLQPRRNPEIGAHTPQFDQRSPPSKAPEKPSEDGYNWRKYGQKLVRGNEFIRSYYKCTHTNCTAKRQVERSQDGHITEINYIGNHEHPKPQNSPQINAPTILPIQMRRPDLPIMTPSEGTQSVTLGEKCETPEPKQITSPVGVVSADIGARDSVLQSHNLRDEDDHCGGPDSKKQKKCLSSPDDNKPHGEPRHVVQTMSEVDIVNDGYRWRKYGQKLVKGNPNPRSSHLVDCIKASKKLSMQLHAYMPEPLDSNFMWSYYRCSNAGCPVKKHVERASHDPKVVITTYEGQHDHDMPASRTITQNSGEGDATSGESRPESGENKHVGLDMVVHIGAN

>IbWRKY40

MEFTSLVDTSLGLNAKPIRVVSGKPKQEVESNFIGLRMNIGNKDENTDKDNNLAGELMEELNRVSAENRKLSEMLTVVCGNYNALREQVREYMNKQQQQSGSINDHNSSQVIMGSRKRKSPSNNNNNANSESSSSDEDSAKKPRRELEHQHHIKANTSKIYVKTEASDTSLIVKDGYQWRKYGQKVTRDNPCPRAYFRCSFAPTCPVKKKVQRSVEDQSILVATYEGEHNHDLPSKLEQPSATATAAAAARSLPPAAALNAQPRDLSPPKTALSVPNANGAKTASTPAGSSLPVDRPDFQQFFIEQMASSLTKDPTFKAAIAAAISGKFSPHNNNREKW

>IbWRKY41

MDNNNNNIDLSLNLNESRRRPRSSELESSEPDKMLQSNAADGEISTGSSLSENQKIEELSVLQREMKRMKEENKALRDAVEQTMKDFHDLHQKFSSIQQKNNHEDKEFAKDFLTLSGTDETRNHRELQERSHQITSDPSPEDGGDEDDDGDGELGLSLTLKSSSSSSSLIGRRMHGEGEERGEKSKAEEMNSTTGFTPTPPPPPPAAMIQNNPPPGFTATSPPNKKTRVSVRARCDAATMNDGCQWRKYGQKIAKGNPCPRAYYRCTVAPGCPVRKQVQRCLEDRSILITTYEGTHNHPLPVGATAMASTASAASFMSHLDSTNPISNLNHAFLIPNYNHNPHFIINPNNPSSHLNIPNLVRNNTIINPTASNPSSSSSPHFWGPKLPDHHHLVADHQNMSAAIAADPKFRVAVAAALSSLISTKDQTHASS

>IbWRKY42

MILYNPITLGLTLENPDQRSAGFFTNKPVFGFNLSPRLNPINAGGSGGMIPINAAAEKRGPPNEVDFFSDKKLPPPQAAADIVVKKEITLHGEPVTKSDLNVNTGLQLVIANAGSDQSTVDDSVSSDMEERRAKNDLSVLQVELEKMNAENQRLRGMLSQVSTNYSALKQHLENLMQSQNQQSSRIGSTQDREVVDRKSEEKKPEKEETTVPRQFLELVPAGGGAAADEPSQSHTSSEERTLSAGSPRNNTELSRHKGIAREDSPDSESWAPNKLPKLNSSKPVDQAAEATMRKARVSVRARSEAPMISDGCQWRKYGQKMAKGNPCPRAYYRCTMAVGCPVRKQVQRCAEDRTILITTYEGTHNHPLPPAAMAMASTTSAAANMLLSGAMPSADGMMNTNFLARAILPCSSSMATISASAPFPTVTLDLTQTPNSLPNYQRPPTQFQPPFAGAPQIPQNYPQLPQVFGQGLYNQSKFSGLHVSHPDIGAAAAQAAQLAQQPRVQPPPPQHPLFADTLSAATAAITSDPNFTAALAAAISSIMGGGSQPNNATNAAAAATSNTNKTSSFPGN

>IbWRKY43

MTPRTRRKTAPIRRAPACFTTPRWPPSIPPKRSRRAIQKRVVSVPINDVEGSKLKGESSFPPSDSWAWRKYGQKPIKGSPYPRGYYRCSSSKGCPAKKQVERSRVDPNMLVVTYSCEHNHPWPAARNNHAHRNAVSLTAAAAAAPTTRSSSKAAAADSEDAREAEAEASEFSAQPKPETSEKIRRTRRFAVGDLQRRVRVVFELRADDVVVDGDREHFHFDGGASDARRHVGDILNAGGGGGRGIAVRRPRRAAGMFEGVRDRNDGERRGSAPA

>IbWRKY44

MCSQFKLHGMENYQGDLADIVRGSGLAGNAAQEAEPPALPDTWQYSSGDNDNIAAAVMNCYSDHRDFGDPFSHMRDPMMFQDLAMPPPPPSAAFFACSDNDLADTAAAAAVSETAASSAVFAPPKLILDEEMKRPACNIFSRMLQISPTSNPPPPGGGGGIACGENIISAASSSGNAAAGLQISSPRNTGSIKRRKSQAKKVVCVPAPAPANSRPSGEVVPSDLWAWRKYGQKPIKGSPYPRGYYRCSSSKGCSARKQVERSRTDPNMLVITYTSEHNHPWPTQRNALAGSTRSQAAKNAAAKSSLSQTPTPNDTQTDPHDTPPVKEETTTAPEDRAMQINDLDDFPAAAGGFPQSYKPALPSSDDPHHHPEDFFADLGEIESTDPLNLMFSQAFPSGQEKKAAGIAIDDAFNFYDWTDSTTTTTIVYKRSENPIPVRGDCNKTIHVGSPR

>IbWRKY45

MESAYNGEYKALLNELIQGMECAKQLRVHLNSAASSETQYFFLQRILSSYEKALLILKWRLVGQSHPVATPLPGAPEPSISLVGSLDINNNNSFKEQQDYNVSKKRKAMPTWTEQVRVGAENGLEGPTEDGYSWRKYGQKDILGAKYPRSYYRCTFRLMHNCWATKQVQRSDDDPTVFDITYKGAHTCNLAPTTSVPPLRSPENQELKQIHHQNESFQAMQSNQMLMNLRASLRVNTDGLDTKETAFPFSFPPTFSDLQTRINTSRAHRSMTMQWHWAHTHPPLFPPPLPNRTTSLLHSNTQMPSREFIYPQPTHHQQTLQLWAWTWTTHFTLQL

>IbWRKY46

MEGDEPPPQLISNPNDLPPQLFSFPSTSLQAPPPSLLSPLLPPTMQSSAQLGPDIDWVGLLSGCTMDQTQAQVLSENGNKSKGKKLVQSGKKNNFPPRIAFHTRSSEDILDDGFKWRKYGQKSVKNSAHPRSYYRCTHHTCNVKKQIQRLSKDRSIVVTTYEGIHNHPCEKLMETLTPLLKQLQFLPRF

>IbWRKY47

MMMAQSVGGCRLEDGALQAVVHGSSTRDSAAAAGFGVFDGRGSPDEDFGVFEDVVGTEMTRIWNSDELEELYKPFYNPVVCASSPVCFPKEVNEQQAVELQHDYQMQPPAAPPATAVYVPKYKRRKSEHKRVVLQVPLEELSDDKWAWRKYGQKPIKGSPYPRSYYRCSSSKGCLARKQVERSCSEPGMFIVTYTAEHCHSQPTRRNSLAGTIRNKFPTPAASKKPQHNYSSEDPPASFMSPAFSSVVSPATVKEEEKMVDENEYNINNVKYDVEDDFFAGLDDLDGLISHFLFCL

>IbWRKY48

MSEDLRDLYYHHPFQEDERSHAGFLFSGAAATSQIHNTTTTNNPHHAFLDPPSSYMSFTDHLIGATEFGRPGGFGFSSSADAAAFSAVKDEQKPSPMNAGDGGGGGGNNNANETPVTPNSSISSSSTEAAGDEDSNKASKRDKQAVDASEDGDDKKETKGKKKAEKKQRQPRFAFMTKSEVDHLEDGYRWRKYGQKAVKNSPYPRSYYRCTSQKCPVKKRVERSYQDPSIVITTYEGQHNHHIPTNLRGTIAGMLPPSLLTPSPLLGAGPPPQISFPPELLAQMTPPHHLFAAHANPFSGAGAAAFHPQNLAQFQLPPDFGLLQDMVPFFKQEP

>IbWRKY49

MVGRFDQTHPSPPEQDDSDTSPENSADSPVSGADHDTKITKVSFPKRRKSAQKRVTSVTINDGEISIPPADSWAWRKYGQKPIKGSPHPRGYYRCSSSKVCPARKQVEKSRADPNVLLVTYFCEHNHSRPAARNAAVMAPDAAVSDDSKRQTATSDHSSQSEGDSEERVAAKCHESPLAAGNDADWLLNFEPATSFAILDESPMMTQTNVTDAGDAPPAVFPDRVEDESLFADLGELPEFSRGFRRGWGER

>IbWRKY50

MAVELMMGYGNDGFAVKIEESAVQEAATAGLKSVENLIRLLSNSPECGGGFSDSVSETPPAEAEFQAAALANAAERAGWESGRFGGKKIDQEPEPEPAAPAADASEKPLSGSKIYCPTPIQRLPPLPHHHFVKNGSAANDRKESSTTISFSGAAAAAAAASPAGSFISSLTGIQTAFSLLSRRASRLQTSRKFPPPAAGRRSPRRRLNGNAIPWMTKSKVKRIVRVPAISLKMADIPPDEYSWRKYGQKPIKGSPHPRGYYKCSSVRGCPARKHVERALDDPTMLIVTYEGEHHHSHSITDPPAAMILESS

>IbWRKY51

MEGEEPPPAPPSLPALLANGDINIQDLFSFPSTSVVEHNNPSMMLSHLPIQSSVVGSDSIDWVGLLSGCMDQMAAPAPASRGGENCVQKNKGKRKKYVPPRVAFHTRSTEDILDDGYKWRKYGQKSVKNSTHPRSYYRCTHHTCNVKKQIQRLSKDKSIVVTTYEGIHNHPCEKLMETLTPLLKQLQFLSRF

>IbWRKY52

MENKFDDLIIKRDSMGIPVFSDEIPSTSPAALQQALLGEADKTTYSLGFLDAQHNNNNYYNTTPIPNTIFDLIIHTHTPPPQHQSIPPPPSHSPSPLASTSLLESSEVVNATPPTPNSSSLSSSSNEATPAANDDQHQTSKTVEEDDEEDKTTKKQVKPKKKKKNAQKRQREARFAFMTKSEVDQLDDGYRWRKYGQKAVKNTHFPRSYYRCTAASCGVKKRVERWCEDASIVVTTYEGTHTHPCPIKHTAGSLGIGIMPPNTSSFFPTTTTTAAAQEGGITSSSSSYCFEELDNNNVILIPSRPSSQQLHYPLQHTSTYFQTPTYSNNISNGAAALLWGRTDSISTTTNLLPLPQGLPPSLIPDHGLLQDMVPFQMVKQEERSSPAKKDI

>IbWRKY53

MAVELLSVTGNSGFAAKMEENAVQEAAAAGLQSVEKLIRLLSQSQPQVSGFFFWVFAAAAGDGGRGRLRGLPGGGGRGCEGPICNPPQAPQPQRKMDQESELGASGQTRVVENSEKPHTGASKMYSPPPIQRLPPLPHNHHHMLKNVPAPPAPDRKESSTTINFSASQATSSPGSFISSLTGDTESLQPSLSSGFQITNLSQVSSAGRPPLSTSSFKRKCNSMDDSSLKCSSAGGSASGRCHCPKKRKSRVKRVVRIPAISMKMADIPPDDFSWRKYGQKPIKGSPHPRGYYKCSSVRGCPARKHVERALDDPTMLTVTYEGEHNHSQSIGDTPASLILESS

>IbWRKY54

MSAPTFLEPNPNYLNYSTAPFSSNIIPDYDYSCYQDLDFFCADNHHLFSDDFTSSNDNNTFGYPSSDLPQTPLVLPEKSNNSTTGSSSCSSDGMPTSTNYMPVKCKRETMKGPKMKEKRAIAFRTRTDLEVLDDGYKWRKYGKKKVKSNSHPRNYYKCSSGGCKVKKKVERDQDDAKYLITTYEGVHDHENLYVIYYQGMPATLTSNGLSLPAASQPY

>IbWRKY55

MLTSLGDCSAPMDGNNHHHHYYYTTHHNDDSTFARQDSGFELSEFFDLDVWPPEEDPVFAVAGHPQNPGQATAVDAVMIPSGAGGVVTYAGPSSSITDSGGGMERTAAGVTEKFAFKTKSDIEILDDGYKWRKYGKKKVKNSPNPRNYYRCSVNGCPVKKRVERDKEDPSYVITTYVGIHNHQGP

>IbWRKY56

MGSCLEHNSPSMDIDLNADVNEIPMRDGEHDTMLEEELKETRAENKKLSAALSAMCENYGSLQSQLLDLMQERSWKRRNSDRDTTTCGSSEIGYDESSVSKRPREIRTNISRVHVQTDPSDTSLIVKDGYQWRKYGQKVTRDNPSPRAYYKCSFAPSCLVKKKVQRSVEDKSILIAIYEGEHNHPHPSETTQTFTLASQTSNLGLGFHNNIQQRSSCDSTIMDTKEIQQLLVEKMASSLTNDHSFTEALAAAISERILDNPLD

>IbWRKY57

MAEELRDFYYHQPFQDDRHGGGFLYSAAQASSMADSSLLHHHHHNLDPTSPYISFTDYLQGSSDFEASAGLGFSSSPSFTSAKDGERRSVNVTTTTTDVGGGGSTETPVVMTPNSSISSSSTEAAGGDNDDSKHKREKLAKETEGEDDGEDNNSSKKENKGKKKGEKKQRQPRFAFMTKSEVDHLEDGYRWRKYGQKAVKNSPYPRSYYRCTSQKCPVKKRVERSYQDPSVVITTYEGQHDHHIPTNLRGSLAGMLPPSMLATSSLLGGPPPQGVTLPPELLMAQINPLAHHFYGGGHNAASMMFQPHQNNLTQMQQLHPDFGLLQDMVPSMIFKQEP

>IbWRKY58

MGLTLKSSESVGIFIEKRAKTLNEVDFFSEKRPPPPAAVLVKKEMPDLNVNTGLELVMANNGGKENQSTADDCAAPEMERRPAKSEVGRLQVELERMKDENQRLKGMLSQATDTYGALHMYFLTLVQQQQQQQTSTTYEPWKIKMLIVVVRSGGGWKENSHSKTVLGHGRRRRRWRRSDRRGIPQFSYLVGERTESESPGNKVEAPVSRSNEGSIGRSREESADSETWRPNKVPRLNNNPSKPVGVDDEDQAAAAASAAAATMRKARVSVRARSEASMISDGCQWRKYGQKMAKGNPCPRAYYRQPTKAHTTIPLPPAAMAMASTTSAAANMLLSGAVPSGDVMMNPNFYGRAIFPSGSIATISASAPFPTITLDLTHQPLNSLPNYPRPPIAQFPFSNAPQNPQHYVSTPQVFGQAGLYNNHSKFSGLQVSNTLQHPSFAHGGATTAAAITADHNFTAALAAAISSIINGSQHIPAHNNPSSNSNQTSSLPAK

>IbWRKY59

MMETRLHHNSSSCFHKQEELSAGTPPENGVESPLSGDEAAEVSTPSPRKRRGAQKRVVSVPAGDGDGSRSKSEVYPRRILGRGESTAKNPLKAPLIPARKQVERSRLDPTMLLITYSSEHNHPHPTKIHHHYAAGTGTGTGAATSSSSAATTPTDSAADPGSPPKPAPKELPIFADPDPENDTFPELAGGEMGWFSDVGPCCFMESATAVAGPTLCHDTDVALTLPIREEDQSLFGDLGELPECSVVFRRCRVETPCYAGTG

>IbWRKY60

MAENHAGRASKSSSSLRLASARPSITLPPPSPIFTGGVSPGPMTLVSSLFGENDQESECRSFSQLLAGAMDSPGARPARRVDPNSKEEEVFRHNGSAITQPSIFTIPSGLSPASLLDSPGQGHFGISHHQALAQLTAQPGIQSDHPSSSLPPAPQFFQLQMPLPGRDPNSIKESSNVSHSDNVSEPCSFPVDKPADDVKKKVERSLDGQITEIIYKGKHNHQPPQPQGAKDTGNQNGLQGSSELNLMDGVPFQSLPMKDQESSLATQEHMSGSSESEKVGDAEARMDRRDDDDERESKRRATEGQIPEPVTSHRTVTEPKIVVQTTSEVDLLEDGYRWRKYGQKVVKGNPFPRSYYKCTTAGCNVRKHVERLASDPKAVITTYEGKHNHDVPAARNSSHNTANNGPALQLRQHNPAAVDNQAALLQFKEEQIT

>IbWRKY61

MDDKGKFDHHEFTADSSGWALRGDADGAYFFGAGGVDKEESSILSDFGWNFQPLDGISGGGGGGGGVGAFDLIHADLAGNGCVSGSGGADESPSGDGEATATQAEPATSSSSEEAAAAEKPSSSSASRPPPPPADTTSSKVKKKGPKRIKQPRFAFMTKSEVDHLEDGYRWRKYGQKAVKNSPFPRSYYRCTNSKCTVKKRVERSSEDPTVVITTYEGQHCHHAVGFRGGFIGHEASAYMARLNPLAVQSYLPGLTAQSHTLPIQSHQNLTHNIESHDPSRRPTPPQLPPDEGLLGDMVPPGMWNK

>IbWRKY62

MEFTSLVDTSLDLNIKPLRPAGDAGVPPKQEVESNFIGLGINMAIKNEADGLVEELNRVSAENKRLTEMLTIMCEKYNDLREKLKGYMMKNNGCEDNSSPVGVLGSRKRKSESNNVNNGGQRSESSSSDEDSVKKPREEQQQQPQHIKSKTSKVYVRTESSDTGLIVKDGYQWRKYGQKVTRDNPSPRAYFKCSFAPTCPVKKKVQRSVEDQSVLVATYEGEHNHPHPSKMDQSSTPPARSAPAPSTTSALTTLNTSAGPTITLDLTDPKPKPSLPITAAAARVLPAPADRPDFHQFLIEQMASSLTKDPSFKAALAAAISGKLIIPHNQTEKW

>IbWRKY63

MEEDKKKAADSSGDDEGYCTHEIGDGNKENERDTLKASSSPNHKTLSSDKEVLKTSYSSQQRLKWGEVMEENQRLKMYLERIMKDYRTLQMQFQGMVEKEGEKAAKSDDNTPQITEESELVSLSLGRASAEMKREEQNRPVIVCAGKDKVDNEDNDQKEGLTLGLDCKFKSLQPNNPSTDNSSDEVKEENGETWPPSKALKTMRSGEDEVSQQNPAKRARVSVRVRCDAPTMNDGCQWRKYGQKIAKGNPCPRAYYRCTVAPSCPVRKQVQRCADDMSILITTYEGTHNHPLPLSATAMASTTSAAASMLMSGSSTSASTSMPPGTTTTTATSTSTTNLNGLNFYLSDTSKPKPFYIPTSSITPTLGHPTIVLDLTSTAPSSSSNLSRIGSLANFPPRFSSTNLNFSSLESNPLPISWNLGTTQPYNKPHITQSLTFARQQQQQPQDNQILFQSYLSKNNLNNNNNNPSQSLPQETIAAATKAITSDPNFQSALAAALSSIMGTNNGNNGASLNLGIHGLAEKLSHNLKAAAEPFPILSSFSPSPNPNKCSSSLLSRPTTTSSANPHPGNLMYHLQQTSKSKSSSPGDSRDQII

>IbWRKY64

MEDSQSHSHSHSQSHAQHHSGEDLDALQSSRVIHAADGSSDASDAVFSRGVAGSNCGARYKLMSPAKLPIARSAGITIPPGLSPTSFLESPVLLSNIKLQIRIRKKTSLWFPLQREGRVKAKGAEPSPTTGSFSNLHLMQGSGGSAAFSFPDSRAFSQRKSSNFEFKFPIGSSSTSASASIEPMTSAGLNQQQSETQRQVQNQFISQSLATSSVVESSIPTSNELNLSSAVALHTSSVGTDAIESDDLNQRGKAVNADQSSVTAERSSDDEYNWRKYGQKLVKGSEFPRSYYKCTYPNCEVKKIFERSPDGKITEIVYKGSHDHPKPQPSRRFTPGAMMPIQEDKFEKESFFNGQEDKFNSIAQTGRPEPSGIPILSPQRVGDDIHEGATLLLQGTNDDTDEDDPFSKKKMDGCVDITPVVKPIREPRVVVQTVSEVDILDDGYRWRKYGQKVVRGNPNPRSYYKCTNAGCPVRKHVERASHDPKAVITTYEGKHNHDLPAARTSSHEMASTAPESGISRVRPEENDVISLDLGVGIGYGTENRTNDRLHSLAPETVPTQVLASGGGMMAVQAPAIVRYGIVNGGINRFGVLTVISENGFLADLPLPEHEKPRLADVICHLPQPANCKGSHFHKSETGLTPLTPQRILCAKQYLISTMQKQKRFHGNYPTPLPLFSKLEAGNPSRNIIEMIFRAASKNPSKHPWLIKRVIKVKNTIDVLDRFEKYREAVKSRLIQSGFNTSYNNKNGIRLSTNSDTLSEDTTVISNRNKAKRAVIVCRTIAGIVEKDQDLLEEEHDSVGHGVDTKLEYLTVKDPSAVLPCFVIVFC

>IbWRKY65

MSDNPFYFHNHMGSGRINTFPFFGDDNSDHNPSSIYSSSDHHPPPSAPTQNLLHQEFLPSPFMSFTESLQGSMDYHTLSNAFGMSCSSSEVVCTPTDHHHHQQQQQQNQESSRKSSVSAGEAAGENIPFVAANSSVSSSSSEAAVGDGEEDSSKSNKDLLLPKGCEDGDDKSTKINKGAAKKKGEKKQREPRFAFMTKSEIDNLEDGYRWRKYGQKAVKNSPFPRSYYRCTSQKCTVKKRVERSYEDPTIVVTTYEGQHNHHCPATLRGNAVALLSPASFLSPSPAALMPNFHQDLLLNPMLSGAPNFQPSSMYGGYHHHHLGLNPHHYDHQITQSPVDQYTLFQDMVVSSLGHKQEHP

>IbWRKY66

MASPSGLSFDPDPIRPPHPTNPHLLLNPPKTTAMNPHRHKFIKLEPFIHGVSGQQQIASPLSNSRASAVDDDVKDLRTSTELDFSINTGLHLLTANTNSDQSIVEDELSPNSEDKRAKSELAVVQAELERMNGENRRLRDTLNQVTNNYTSLQMHVMTLMQQQQQQSHGKTEESKQTRQNNGGQMLPRQFMDLGLAAGGGGGPTEADEASLSSSEGRSGREGSQSPTNNLDEASRADSPEKGSGWRSNKVARSGHASKSGNIDQATEATMRKARVSVRARSEAPMITDGCQWRKYGQKMAKGNPCPRAYYRCTMAAGCPVRKQVQRCAEDRTILITTYEGNHNHPLPPAAMAMASTTSSAAKMLLSGSMPSADGLMNSNFLARTLLPCSSSMATISASAPFPTVTLDLTQNQNPNQSQFPRPPNPFQFPFPNPSHNPAAALLPQIFGQALYNQSKFSGLQLSQDLENIHAPPSSMPPHQSSQQNPLADTVNALASDPNFTAALAAAITPSSETPPIPATLLTPPTPIITAALLPAAMAMTKSTVEILSPNPNCYTY

>IbWRKY67

MESPVNNRSPPPTIQFPVNRNCSDHRRHAHDQEDHDHKTTVLGEMDFFAYTKDNNNGDSRASAVDDDVKDLRTSTELDFSINTGLHLLTANTNSDQSIVEDELSPNSEDKRAKSELAVVQAELERMNGENRRLRDTLNQVTNNYTSLQMHVMTLMQQQQQQQQSHGKTEESKQNPRQNNGGQMLPRQFMDLGLAAGGGGGPTEADDASLSSSEGRSGREGSQSPTNNLDEASRADSPEKGSGWRSNKVARSGHASKSGNIDQATEATMRKARVSVRARSEAPMITDGCQWRKYGQKMAKGNPCPRAYYRCTMAAGCPVRKQDNLDHNLRREPQPPTAAGGHGNGIHDILGGEDAVVGIHAERRRADELKFPSENPPPLLFKHGHHLSLRSIPHCYIGSHPKPKPKPIPIPQTPKPLPLPFPNPSHNPAAALLPQIFGQALYNQSKFSGLQLSQDLENIHAPPSSMPPHQSSQQNPLADTVNALASDPNFTAALAAAITSLIGNPSHSGNASNASNANNNGSVITSSNGNDKVNS

>IbWRKY68

MQFPAAMEDENFVFNLVIDDDNSPDEMKSSIGESFPSTSANETHSSSPMQIIPFDFPMESLDCGVDMSSAVDTNPNSNSNLMLQSPVNGECINNNVNGETCSPFNNPEPELDHRRSAAAAPAAKSLANNNNNSAGSSSDDGYTWRKYGQKHVKGSEYPRSYYKCTHPKCTMKKKVERSPDGQITEIVYKGAHNHPKAPATSLRRSPPSLGAESSSSEMMSQGSGSCFRSQAPIWANIHHYGSMPERSALASSSDLTAEICDPLSSLTTRSAAAMSGFESATTPEPSSTLASQDCDDNEDAVTQGISPSQFGEDGESEPKRRRKDGWSIEANLSTRSIREPRVVLQIESEIDILDDGYRWRKYGQKVVKGNPNPRSYYKCTSPGCPVRKHVERASDDLKSVITTYEGKHNHEVPPNKAAVVNYNSYSATSGTTASSAMPRAPALGGVGVQDHHPSFPFERKPMIAGAGGDELLRPEMLDCYAAGDFRFVPSSIYPLKFPPPPLQGPLTAAAATFNYSRPPGMVLPEFPMPLLPMSLPPFHELTNLPPLADFLHFNDPSTKEEHKENDPHTSLLYE

>IbWRKY69

MNTCSNNNNNESPHDHMDIDLSLKLNESPQDSPQPCRRIIITTEQRRFPIVVQKFQDRRVLQTEMKRMKEENKVLREAVEHTMKDFLDLQTKLALFVQQNDHKKDVISNFLWVNGEEEKISQELNRTSSSSSPTAQLEHDKNNNNNSISDTELGCRSRCKPPPPQMIMRKEEIRRVRHRRRRQYTVIFTHGSSFAAGLCQESPISQHNVRKPRVSVRARCESATMNDGCQWRKYGQKIAKGNPCPRAYYRCTVAPGCPVRKQVQRCIEDMSILITTYEGTHNHPLPVGATAMAASTASTAASFMFLDSSNPISNNNLGIPQNQPFLNSQNYHHMIPTLTRNLNPNSAMATPYHHDPAASSKLGLVLDLTKDGNFDGHLQPGNTAENDKQKLVENVSAIAADPKFRVAVAAAISSFINKDKPPS

>IbWRKY70

MSSSSTTSQAMLNQILFQDVASSSSSLFCVSSNNNNITGTGTGTALPFESLKTLITVPMPTSLASLLPPLVESSSPNSTSAFHQTQTQTLLQQHQDLSPLFGPPHHHQLLSLHRSAPNLWAWGEVNECMMRSKRSGLVVDDHRHHHHLGGLGVSAVKMKKMSKSRRKVREPRFSFKTMSDVDVLDDGYKWRKYGQKVVKNTQHPRSYYRCTQDNCRVKKRVERLAEDPRMVITTYEGRHVHSPSHDDDDSQASSQANDLLW

>IbWRKY71

MEDDWDLHAVVRGCAAAASSSAATATATTASPLGSAFQPRQDQDLLCLQDLLEFGAKRNESRRRFNDDLHDLYRPFFRPPPPPSQPPQPSLQRTQAAPPLSPQNTPISPLSVLGGLPDLSPSHQILKPHISPAATIIHPKKQPFPVNISSATTPTTPHTQSPRSKRRKNQLKRVCQVPAEALSSDVWSWRKYGQKPIKGSPYPRGYYRCSTSKGCLARKQVERNRSDPSMFIVTYTAEHNHPMPTHRNSLAGSTRQKPAAAHQPETSGDTMKSGASPPAPEKQESSRDEREDMFEDEDDEFGVANMAIDSLGPDDDFFEGLEDLGGDCFPDSLPGTTLQFPWLTTTTAAGGG

>IbWRKY72

MANKSGGGLSFDPDPISTDNNHHFFRQPHSFLTLPLPSQMDSPVNNSPPTLQFPVHVPCAADHHNDRKTIRVSGEVDFFAHKDDRASAAVATAADDLDFDVNIGLHLHTTNATGGRSIVEDALSPNSQDNRAKNEVGVIKAELDRMNAENQHLRDMLHQATNNYSALQTHLTSLMQQQQQQNGGGGGLTAPPPPLRPFMGLGAEAAENASQSSSEGKSGGEPPRHGSKSSSGDNVDQATEATIKKARVSVRARSEAAMITDGCQWRKYGQKMAKGNPCPRGYYRCTMATGCPVRKQTAMDNLDNVVVTNFLITHEAEDRTILITTYEGNHNHPLPPAAMAMASTTSSAARMLLSGSMPSADGASMAALMNNSNFLARAFLPAGMATISASAPFPTPLFNQTKFSGLHLSHDLENQAMHHQQNPAELADTVNALTSDPNFPAALAAAIASILGGSSVPDNTAASNAATLGNDNAGSVTMETTK

>IbWRKY73

MANKSGGGLSFDPDPISTDNNHHFFRQPHSFLTLPLPSQMDSPVNNSPPTLQFPVHVPCAADHHNDRKTIRVSGEVDFFAHKDDRASAAVATAADDLDFDVNIGLHLHTTNATGGRSIVEDALSPNSQDNRAKNEVGVIKAELDRMNAENQHLRDMLHQATNNYSALQTHLTSLMQQQQQQNGGGGGLTAPPPPLRPFMGLGAEAAENASQSSSEGKSGGEPPRSSPVNNVESVSGENMHSLEKGGSSNVKVGRTGHGSKSSSGDNVDQATEATIKKARVSVRARSEAAMITDGCQWRKYGQKMAKGNPCPRGYYRCTMATGCPVRKQVQRCAEDRTILITTYEGNHNHPLPPAAMAMASTTSSAARMLLSGSMPSADGASMAALMNNSNFLARAFLPAGMATISASAPFPTVTLDLTQTQTPNPLHFPRIPPNHSHNNNINNQAAFSPLFGQPLFNQTKFSGLHLSHDLENQAMHHQQNPPAELADTVNALTSDPNFPAALAAAIASILGGSSVPDNTAASNAATLGNDNAGSVTMETTK

>IbWRKY74

MENGWGLTLENSIFKNGLGSSRPRFDTANMFPVKKDDGERRQQAVLNEVDFFSEKKKPVDSGFVVKKETSNDEPPIRTDLNINTGLQLVTANTGSDQSTIDDGVSSGMEERRVKDEVAGLQAELERMNGENQRLKGMLTQVTNNYTALEMHLAALMQQQKKNSMAESAHEVVDRKSEEKKYEKEGSTVPRQFLDLGPSGLTGAEQTDEPTHSPTTSSEEKTISASPRNNVDSSKHKRSAREESPDLESWNPNKAPKSIISSSSSRPVDDQQASTDATMRKARVSVRARSEAPMISDGCQWRKYGQKMAKGNPCPRAYYRCTMAVACPVRKQVQRCAEDRTILTTTYEGTHNHPLPPAAMAMASTTSAAANMLLSGSMPSADMMMNPNFLARAILPCSSSVATISASAPFPTVTLDLTQTPTSLPNYPRLPPSQFPGAGAPHSLPGFAVTPPQVFGQGLYSQSKFSGLQVSHEAQHPLLPPAHPSLSDTLSAATAAITADPNFTAALAAAISSILNGSQPNIVNSTNNNTTATANAPNSNPTNNCNKTH

>IbWRKY75

MNTCSNNNNNESPHDHMDIDLSLKLNESPQDSPQPVGESSSQPNKGDSPSSSKNSKTEEFIRWDDPHHYEARAQSGLHNSAANGNETHERGEQGVEGGSGAHHERFSRSADQTCTICPAKRSQKDVISNFLWVNGEEGKISQELNRTSSSSSPTAQLEHDNNNNNNSISDTELGLSLTLQTAAAADDHEKGRDQKSPPSPPPSIHGNIHHGSSFAAGLSAKNPPISQHNVRKPRVSVRARCESATMNDGCQWRKYGQKIAKGNPCPRAYYRCTVAPGCPVRKQVQRCIEDMSILITTYEGTHNHPLPWVQPPWQPQQLQQQPPSCFWILPTPFPTTTSEFPKTNPSSKLGLVLDLTKDGVSSSASSSTSLPKLQGQMGQYSSWMMQRLAGNFDGHLQPGNTAENDKQKLVENVSAIAADPKFRVAVAAAISSFINKDKPPS

>IbWRKY76

MMSGSDFLQANINPPSKDHPTFNHESFDNLPSIIEDYHRHYYPISEVSSYISYVNHFLNDESPAPAPPNSTGHSFGSPPSAAVVTQERSCTTTTTTGSSCSSFDGMPPTSSPHSQMIGYSSNEMRRKLKVNKEEQTIAFRTKTELPVLDDGYKWRKYGKKMVKSNTNPR

>IbWRKY77

MAVELMRGFRSESLGGKMEENAVQEAATAGLQSVEKLIRLISQSQPQNSGFSAAPPLPAFSADYQAEAGAAVTKFKKFISLLDRSRTGHARFRRGPVVNQKREVDPPPVNQNSSSRIRVSEEQPEKKIYHPKPIQCLPPLPHHHQPAKNASAANTIDRKEPSTTISFAAMAAPSPAGSFISSLTGDTDSVQPSLSSGFQITNLSQVSSAGKPPLSSSSFKRKSSSIDDAAVKCHSTGGSASGRCHCPKKRKTRVKRVVRVPAISLKMADIPPDDYSWRKYGQKPIKGSPHPRGYYKCSSIRGCPARKHVERALDDPTMLIVTYEGDHNHSRSITEAPSALILESS

>IbWRKY78

MQVFGSTGGFSFPRSNIYIESKSNNFEFEFPVGSYSTSESSSIGPKQNEPLKQAQDQCLPQSLAPSSLLESSIIPTSKELRISAPVAVHTSSVSTAPIESDELNQRGQSNPGIQTLNGDQPAAGAERSSEDGYNWRKYGQKLVKGSEFPRSYYKCTYPNCEVKKIFERSPDGQITEIVYKGSHDHPKPQPSRRFTPGSLTSIQEDKFEKESSFNVKEDKLNSNDFNGYPVLSPGRVDDDGHEGAASQLLVTNDDTDDDDPFSKRRKLDGCVDITPVVKPIREPRVVVQTVSEVDILDDGYRWRKYGQKVVRGIQIPGCSVRKHVERASHDPKAVITTYEGKHNHDVPTARTSANHETGATPLGGASRVRPEENDAISLDLGVGIGYGTEHRPNGQLHSLAPETLQGQVHVSSSGMMTVQPPPMVCYGPIHGGINRFGSTRQNMVQAPGFDTLPLQPANQCPQTLGRILLGP

>IbWRKY79

MGGFDDHVAIMGDWMPPSPSPRAFFSSILGDDLGTRSAAEPTRENKTGTLVSEPEGYASSRNSDGKGGDQAGSMSSLSEQKIGSRGGGLLERMAARAGFNAPRLNTEGIRTAADLSQNQEVRSPYLTIPPGLSPTTLLDSPVFLSNSLAQPSPTTGKFPFPSSGDFQNSTMFMEASRKSKESSFDNNDSSSFAFQPVIQTGPSTFPGTFGKVPPSNLARQSFPSVEPTQVYAQNGTIQRPDFSRSSTEKDNGSGNITSEHSPPPDELPDEETDQRGSGDPNFLGAPADDGYNWRKYGQKQVKGSEYPRSYYKCTHLNCPVKKKVERSQEGHITEIIYKGAHNHPKPTPNRRSALGSANPLGDMQIENTEQAGTGGDGDPIWANMQKGSGAGDPDWRNDNLEVTTSAPLGSEYCNGSSLQAQNVTQFESGDAVDRSCTFSNDEDEDDRGTHGSVSLGYDGEGDESESKRRKIETYAADMSGATRAIREPRVVVQTTSEVDILDDGYRWRKYGQKVVKGNPNPRVNIASTEAQSHDPPYRRVTTWHLSTRSYYKCTSAGCTVRKHVERASHDLKSVITTYEGKHNHDVPAARNSSHVNSGVSNSGPSQATVPNQTHLHRPEPSQLQNTMARFEGPLSLGLTNLAMAGLGANPGKLPVLPVHPYLGQQRPVNHMSFMMSKGEPKMEPVSDPGLNISNSSSVYQQVMSRLPLGPHM

>IbWRKY80

MKLLPSSHETYYEGSDDSDQTTYLYFHHYSFFFTALPTRIDPMDGNYQNTGSPFGSPHHQPVFEPSEFLELSDWAEEEPAAMHVSGGHYYPLLNPPHHQVPPPPEGVHGGYLQGGPRNNGGSYGGGREKFAFKTKSEVEILDDGYKWRKYGKKMVKNSPNPR

>IbWRKY81

MKLLPSSHETYYEGSDDSDQTTYLYFHHYSFFFTALPTRIDPMDGNYQNTGSPFGSPHHQPVFEPSEFLELSDWAEEEPAAMHVSGGHYYPLLNPPHHQVAPPPEGVHGGYLQGGPRNNGGSYGGGREKFAFKTKSEVEILDDGYKWRKYGKKMVKNSPNPSPPLTLVILFPLNSKK

>IbWRKY82

MGYYSSSLGDKSLAIDLNTSINMDCEAADTCGNSNSSVGDQKHGLLMEELKVTKMENKKLAAKLTEVCENYCTLQNHFLGLLKTHGADDDFLGKRKSADDDGDCYGAAAASSPKRPRETRTPVSRVRVKTDPSDMSLVVKDGYQWRKYGQKVTRDNPSPRAYYKCSFAPSCPVKKKVQRSVEDPSILIAIYEGEHNHPHPTQPEVLSVPLPQGFTPQSICSPVSDVENSSSPARLDIRAKLQRSLTSIDSVELQHFLAEKMASSLTKNRSFTDALAAAISDRILLDHALADSCS

>IbWRKY83

MGYYSSSLGDKSLAIDLNTSINMDCEAADTCGNSNSSVGDQKHGLLMEELKVTKMENKKLAAKLTEVCENYCTLQNHFLGLLKTHGADDDLLGKRKSADDDGDCYGAAAASSPKRPRETRTPVSRVRVKTDPSDMSLVVKDGYQWRKYGQKVTRDNPSPRAYYKCSFAPSCPVKKKHNHPHPTQPEVLSVPLPQGFTPQSICSPVSDVDNSSSPARLDIRAKLQRSLTSIDSVELQHFLAEKMASSLTKNRSFTDALSAAISDRILLDHALADSCS

>IbWRKY84

MKNQTALFLGFTPPLAASSEHAKKGSGGTKVVGYPTAEEANDVTNINNSDEEISLLSQKESSGGVVSKKKGEKKTRMPRFAFQTRSQVDILDDGYRWRKYGQKAVKNNKFPRLVSTSTLP
